# Hesperetin promotes bladder cancer cells death via the PI3K/AKT pathway by network pharmacology and molecular docking

**DOI:** 10.1038/s41598-023-50476-8

**Published:** 2024-01-10

**Authors:** Yue Lv, Zhonghao Liu, Leihong Deng, Shunyao Xia, Qingchun Mu, Bang Xiao, Youcheng Xiu, Zan Liu

**Affiliations:** 1 Department of Urology, The First Affiliated Hospital of Harbin Medical University, 23 Postal Street, Harbin, 150000 Heilongjiang China; 2 Key Laboratory of Hepatosplenic Surgery, Ministry of Education, The First Affiliated Hospital of Harbin Medical University, Harbin, 150000 Heilongjiang China; 3 Department of Ultrasound Medicine, The First Affiliated Hospital of Nanchang University, Nanchang, 330006 Jiangxi China; 4 Department of Neurosurgery, Hainan General Hospital, Hainan Affiliated Hospital of Hainan Medical University, Haikou, 570311 China

**Keywords:** Cancer, Cell biology, Molecular biology

## Abstract

Patients with bladder cancer (BLCA) still show high recurrence after surgery and chemotherapy. Hesperetin (HE), as a natural compound, has attracted researchers’ attention due to its low toxicity and easy access. However, the inhibitory effect of HE on BLCA remains unknown. The hub genes and enrichment pathways regulated by HE in the treatment of BLCA were predicted by network pharmacology. The molecular docking of HE and hub proteins was visualized. Colony and CCK8 assays were used to test cell proliferation, and BLCA migration was confirmed by transwell and wound healing assays. In addition, the occurrence of apoptosis and ferroptosis was demonstrated by Hoechst staining, transmission electron microscopy (TEM) and ROS (reactive oxygen species) assay. Western Blotting was performed to validate the hub proteins, target functions and pathways. SRC, PIK3R1 and MAPK1 were identified as hub targets for HE in BLCA, involving the PI3k/AKT pathway. Furthermore, HE inhibited the proliferation and migration of BLCA cells. The MMP2/MMP9 proteins were significantly inhibited by HE. The increased expression of Bax and cleaved caspase-3 indicated that HE could promote BLCA cell apoptosis. In addition, Hoechst staining revealed concentrated and illuminated apoptotic nuclei. The activation of ROS and the decline of GPX4 expression suggested that HE might induce ferroptosis as an anti-BLCA process. Shrunk mitochondria and apoptotic bodies were observed in BLCA cells treated with HE, with reduced or absent mitochondrial cristae. We propose for the first time that HE could inhibit the proliferation and migration of BLCA cells and promote apoptosis and ferroptosis. HE may act by targeting proteins such as SRC, PIK3R1 and MAPK1 and the PI3K/AKT pathway.

## Introduction

As the common type of urinary tract tumor, bladder cancer (BLCA) is the tenth most common cancer in the world. BLCA has an annual incidence of about 549,000 cases and 200,000 deaths^1^. Smoking has long been recognized as the most common risk factor for BLCA, accounting for about half of all factors^2^. Based on the degree of tumor invasion, non-muscle invasive BLCA (NMIBC) accounts for 70–75% of BLCA cases, while muscle invasive BLCA (MIBC) accounts for 25–30% of cases. Clinical malignancy, probability of metastasis and recurrence, and poor prognosis were significantly correlated with the invasive BLCA^3^. Therefore, early discovery and diagnosis of BLCA are essential. Transurethral resection of bladder tumor (TURBT) remains the primary treatment for NMIBC. In addition to surgical resection, platinum-based chemotherapy and even immunotherapy are crucial for advanced patients,^3,4^ but the risk of recurrence remains high for patients with distant metastases^5^. The development of resistance to cisplatin has also become the mainobstacle in the treatment of patients with advanced BLCA^6^. Therefore, the exploration of new treatment methods is still the main direction of BLCA research.

Flavonoids are the main medicinal components of many plants, and they exert a variety of biological activities^7^. Flavonoids have been found to have great anti-cancer potential. A decreased incidence of cancer appears to be associated with a high intake of vegetables and fruits that are rich in flavonoids^8^. Hesperetin (HE) is a natural flavonoid found mainly in citrus fruits such as tangerines, oranges and grapefruit. It has recently received attention for its anti-tumor properties against many types of cancer^9^. HE was found to significantly reduce gastric cancer cell proliferation, invasion and migration. Mechanistically, it could inhibit DOT1L protein abundance and histone H3K79 methylation^10^. In addition, HE was shown to enhance the cisplatin-induced apoptosis of gastric cancer cells^11^. It also induced apoptosis in glioblastoma and lung cancer H522 cells via the P38 pathway and p53-independent pathway^12,13^. Quercetin, a flavonoid, has been shown to induce ROS-dependent ferroptosis and promote cancer cell death^14^. This suggests that flavonoids could promote apoptosis and ferroptosis as an anti-cancer mechanism. However, the anti-BLCA effects of HE are still unknown.

**Figure 1:**
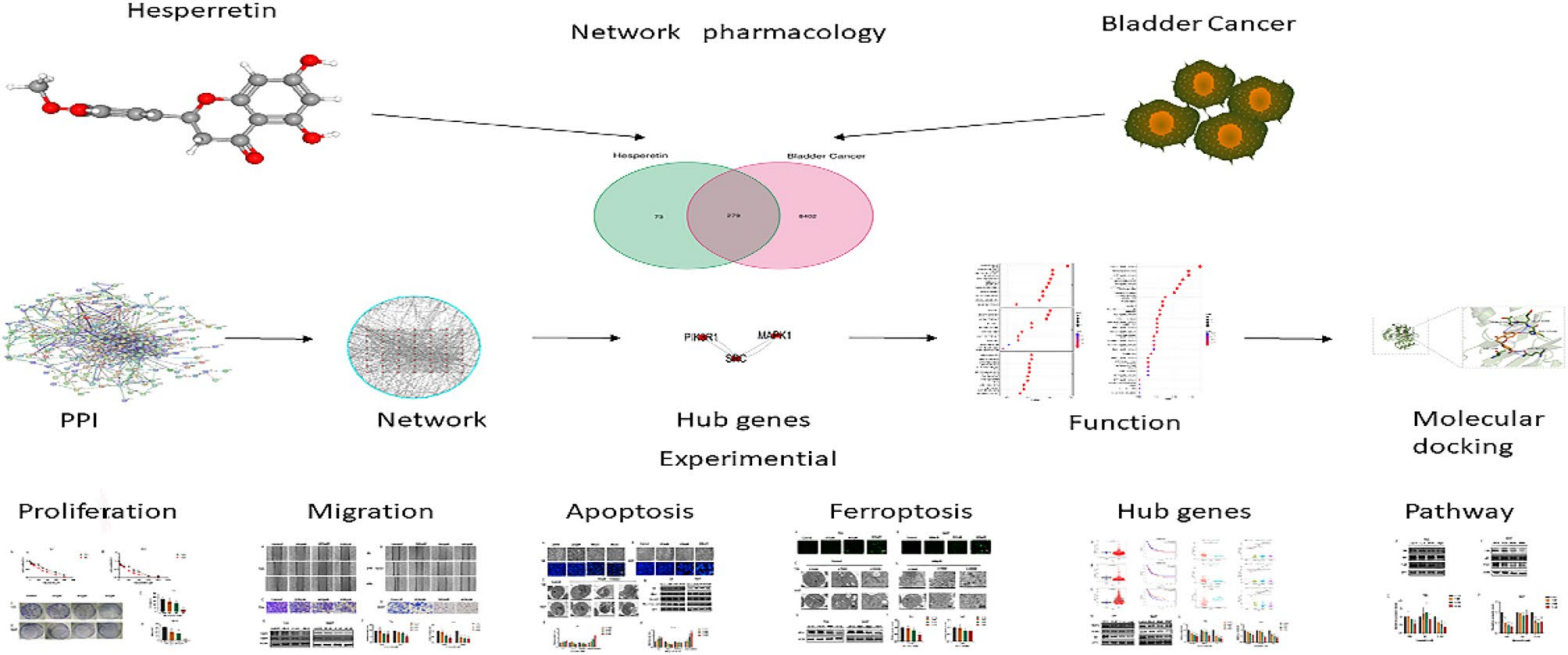
The workflow of the study.

Network pharmacology can predict the interaction targets between drug and disease and construct the corresponding proteins interaction network to predict the molecular mechanism^15^. Therefore, the potential targets between BLCA and HE were identified and validated using network pharmacology. In addition, GO and KEGG analyses were performed to predict the anti-BLCA basic pathway. SRC, PIK3R1 and MAPK1 were identified as hub targets for HE in BLCA, involving the PI3k/AKT pathway. Increased expression of MMP2/9 can lead to the degradation of extracellular matrix and induce tumor cells to migrate to other organs^16^. We found that HE significantly inhibited these two proteins. The anti-apoptotic protein BCL-2, the pro-apoptotic protein Bax, and the splicing of caspase-3 are classic intrinsic mitochondrial apoptotic pathways^17^. GPX4 is an important regulator of ferroptosis. The XCT system can inhibit the synthesis of GSH to reduce the expression of GPX4, thus reducing the cells lipid peroxides and promoting cellular ferroptosis^18^. Apoptosis and ferroptosis were further confirmed by validating these proteins. The workflow of the study is shown in Fig. 1.

## Methods and materials

### Pharmacology network of HE

#### Targets prediction

The corresponding 2D and 3D structures of HE (Compound CID: 72,281) were downloaded from the Pubchem database (https://pubchem.ncbi.nlm.nih.gov/)^19^. The PharmMapper database (http://www.lilab-ecust.cn/pharmmapper/submitfile.html) was used to obtain the predicted gene targets of HE. The gene symbol was then recognized by the Uniprot (http://www.uniprot.org/) database^20–22^. In addition, the HE Canonical SMILES (COC1=C(C=C(C=C1)C2CC(=O)C3=C(C=C(C=C3O2)O)O)O) was downloaded from the Pubchem database and entered into the SwissTargetPrediction database (https://labworm.com/tool/swisstargetprediction) for target prediction (probability > 0)^23^. Furthermore, the BLCA gene targets were predicted through five databases, including GeneCards (https://www.genecards.org)^24^, OMIM (https://www.omim.org)^25^, PharmGkb (https://www.pharmgkb.org)^26^, TTD (http://db.idrblab.net/ttd/)^27^ and DrugBank (https://www.drugbank.ca/)^28^. In these databases, the “bladder cancer” keyword was input to obtain the corresponding BLCA targets.

Two database targets of HE and five database targets of BLCA were collected. Duplicate genes were deleted, and a corresponding Venn diagram was drawn. Finally, the intersection genes of BLCA and HE were obtained (http://bioinformatics.psb.ugent.be/webtools/Venn/)^29^.

#### Protein–protein interaction and hub genes

The intersection genes of HE and BLCA were analyzed through the online STRING (https://string-db.org/) website to obtain a protein–protein interaction (PPI). The species was limited to humans, the confidence score was set to greater than 0.9, and the disconnected nodes were deleted. The downloaded TSV files were imported into Cytoscape3.8.0 for analysis and construction of the visual network of PPI. The hub genes were screened from the gene network by various parameters, including Betweenness, Closeness, Degree, Eigenvector, LAC, and Network. The median value of each parameter was used as the standard in each screening.

#### GO and KEGG

The GO (URL:geneontology.org) and KEGG (URL: www.kegg.jp/feedback/copyright.html)^30^ pathways were analyzed for intersection genes. The ID annotation of the targets was obtained by using the "org.Hs.eg.db" R package. Then the biology functions and enrichment pathways of the targets were predicted by using the "clusterProfiler", "enrichplot", "ggplot2" and other R packages, with the filter condition *P <* 0.05. The GO analysis was divided into three parts: biological process (BP), cellular component (CC) and molecular functions (MF). The first 30 enrichment pathways were obtained and visualized.

#### Molecular docking

The SDF file of HE 2D structure downloaded from the Pubchem database was imported into chem3D software to obtain the 3D form of free energy minimization. The protein crystal structures of the hub genes were downloaded from the RCSB protein database (https://www.rcsb.org/). PyMOL 2.4.0 was used to remove water molecules and ligands from the protein structures. In AutoDock Tools 1.5.6, the protein structures were added to nonpolar hydrogen and converted to PDBQT format. In addition, the small molecule ligand (HE) was also transformed into the PDBQT format to be used for docking^31^. The structure of the protein receptor only displayed secondary structure and undisplayed lines. The location of the active pocket was then determined by defining the spacing (Angstrom) as 1. Subsequently, docking was performed with the Autodock Vina software^32^, with lower binding energy indicating a more stable structure. The protein–ligand interaction profiler (PLIP) website (https://plip-tool.biotec.tu-dresden.de/plip-web/plip/index) was used to visualize the interaction between ligand and receptor, including π-stacking (parallel and perpendicular), π-cation interactions, hydrogen bonds, water bridges, salt bridges and so on.^33^

### Verification in Vitro

#### Cell culture

T24(HTB-4) and 5637(HTB-9) Human BLCA cell lines were obtained from the American Type Culture Collection (ATCC, USA). The 5637 cells and T24 cells were cultured in Roswell Park Memorial Institute (RPMI)-1640 medium (Gibco, USA) containing 10% fetal bovine serum (FBS, BI, Ausbian) and 1% penicillin and streptomycin (Beyotime, Shanghai) in a moist incubator with 5% CO2 at a constant temperature of 37 °C.

#### Reagents

Hesperetin (B20184-20 mg,  >  = 98%) was purchased from Yuanye, Shanghai. The concentration of HE was 600 mmol/L in dimethyl sulfoxide (DMSO, Beyotime, Shanghai) solution. After further HE dilution, the concentration of DMSO was ensured not to exceed 0.1%. The western blotting assay was performed with the following antibodies: anti-ACTIN (23660-1-AP, Proteintech, 1: 10,000), anti-BCL-2 (12789-1-AP, Proteintech, 1:1000), anti-BAX (505,992–2-lg, Proteintech, 1:1000), anti-Caspase-3/Cleaved-caspase-3 (196,771–1-AP, Proteintech, 1:1000), anti-MMP9 (10,375–2-AP, Proteintech, 1:1000), anti-GPX4 (67,763–1-lg, Proteintech, 1: 1000), anti-AKT (60203-2-lg, Proteintech, 1:1000), anti-P-AKT (66,444–1-lg, Proteintech, 1:1000), anti-PI3K(P110) (20584-1-AP, Proteintech, 1:1000), anti-SRC (11097-1-AP, Proteintech, 1: 1000), anti-PIK3R1(P85) (60,225–1-lg, Proteintech, 1:1000), anti-MMP2 (A19080, ABclonal, 1:1000), anti-MAPK1 (A19630, ABclonal, 1:1000).

#### Cell counting Kit 8 (CCK8)

BLCA (T24/5637) cell suspension was prepared by DMEM or PMI-1640 and then seeded into 96-well plates with 5000 cells per well for 24 h at 37 °C. HE of different concentrations (0, 25, 50,100, 200, 300, 400, 600, 800 **µM)** was added to each column for 24 h/48 h at 37 °C. The culture fluid was then removed. 10 μl CCK-8 (APEXBIO, USA) solution was added to each well and incubated at 37 °C for 1–4 h. The absorbance at 450 nm for each HE concentration was measured using the microplate reader. The IC50 for each sample was calculated by Graphpad Prism8.4.3.

#### Cell colony assay

The BLCA cells (800/well) were cultured into the dishes with a diameter of 6 cm. After 24 h of culture, the liquid was replaced by different HE concentrations. The BLCA cells were cultured for 24 h and then replaced with fresh medium for 10 days. The cells were then washed with PBS, fixed with methanol for 15 min, and stained with crystal violet for 20 min. The images were processed and analyzed with ImageJ software.

#### Wound healing assay

5637 and T24 cells were cultured in a six-well plate until the cells were completely filled. A 200**µ** pipette was used to make vertical scratches in each well. Different concentrations of HE were prepared in an FBS-free medium. The degree of cell wound healing at 0, 24 and 48 h wasobserved under a microscope.

#### Transwell assay

The transwell chambers were placed in a 12-well plate, and the lower chamber was immersed in a complete medium containing FBS. The upper chamber was filled with different concentrations of the drug from the FBS-free culture medium. The cell density was about 1 × 10^5^ cells per well, which were cultured for 48 h at 37 °C. After fixation with methanol for 15 min, the samples were dyed with crystal violet for 15 min. A cotton swab was used to wipe off the upper chamber cells. The stained cells were photographed and counted under a microscope.

#### Hoechst 33,258 staining

The induction of BLCA cell apoptosis with different concentrations of HE was assessed by analyzing Hoechst 33,258 (Beyotime, Shanghai) staining of apoptotic cell nuclei. The cells were cultured in a 24-well plate. Hoechst 33,258 solution was added to the cells for 48 h after HE intervention, and the cells were fixed with 4% paraformaldehyde (Biosharp, Guangzhou). The samples were washed twice with PBS and then observed under white light and blue light, respectively, to observe the morphology of the cell nucleus.

#### ROS assay

After cells were treated with HE for 48 h, the DCFH-DA fluorescence probe (Beyotime, Shanghai) was diluted with a 1:1000 FBS-free medium. After 20 min of culture at 37 °C, the cells were washed three times with an FBS-free medium. The green fluorescence was observed directly under a fluorescence microscope.

#### Western Blotting Analysis

The cells were cultured on a six-well plate. After HE treatment for 48 h, PBS was washed twice. The cells were then placed on ice with RIPA lysis buffer (Biosharp, Guangzhou) for 30 min. The solution was centrifuged at 12,000×*g* at 4 °C for 15 min, and the supernatant was collected. Electrophoresis was performed according to the concentration of proteins. The nitrocellulose (NC) membrane (0.45 um, merckmillipore, USA) was sealed with 5% TBST milk for 1 h, and proteins were transferred to the membrane at low temperatures. The membrane was then cut according to the desired protein molecular weight. The primary antibodies were diluted with bovine serum albumin (BSA, Biosharp, Guangzhou) and were incubated with the membrane overnight. TBST was washed three times and incubated at room temperature for 1 h under the fluorescent secondary antibody (Sigma-Aldrich, USA). TBST was used for washing three times. Finally, the gray ratio was analyzed by using ImageJ software (version: ij153 URL: imagej.nih.gov/ij/index.html).

#### Transmission electron microscopy

After HE treatment for 48 h, BLCA cells (T24 and 5637) were trypsinized, centrifuged, and stored at 4 °C in 2.5% glutaraldehyde. After being fixed in 1% osmic acid, BLCA cells were dehydrated with gradients of ethanol. After drying, the samples were embedded in epoxy resin and sliced into approximately50 nm sections for observation. The cell morphology and internal organelles of sections were observed using a transmission electron microscope (TEM) (JEM-1400Plus, Tokyo, Japan) with lead citrate and uranyl acetate.

### Statistical analyses

The bioinformatics of this study was analyzed by R Studio 4.1.3 software. Statistical analysis of the subsequent experiments was performed using GraphPad Prism 8.4 0.3 (California, USA). The difference between groups was analyzed using a t-test (unpaired). All data were collected from at least three independent experiments. *P <* 0.05 was considered statistically significant.

## Results

### Target Prediction

**Figure 2:**
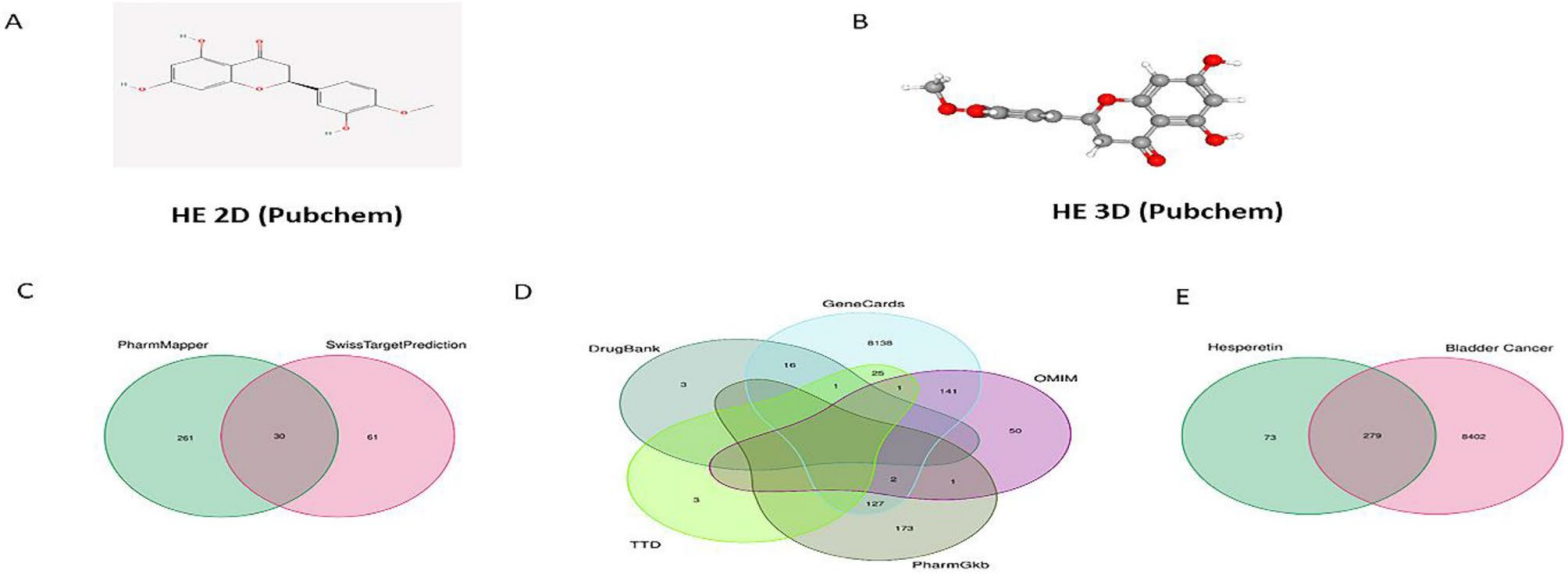
(**A**, **B**) The 2D and 3D structures of HE. (**C**) The venn diagramHE- related gene set. (**D**) The venn diagram of targets related to BLCA. (**E**) The intersection of HE and BLCA gene sets.

The 2D and 3D structures of HE are shown in Fig. 2A,B. In total, 91 gene targets from the SwissTargetPredicition site and 291 gene targets from the pharmapper site were found to be associated with HE. The HE-related gene sets were combined, and duplicate genes from two databases were deleted (Fig. 2C). We collected 8,681 targets related to BLCA. The number of targets in each database and the intersecting genes are shown in the Venn diagram (Fig. 2D). Finally, the intersection of HE and BLCA gene sets was analyzed, revealing 279 gene targets that might be involved in the mechanism of HE on BLCA. (Fig. 2E).

### Screening hub genes

**Figure 3:**
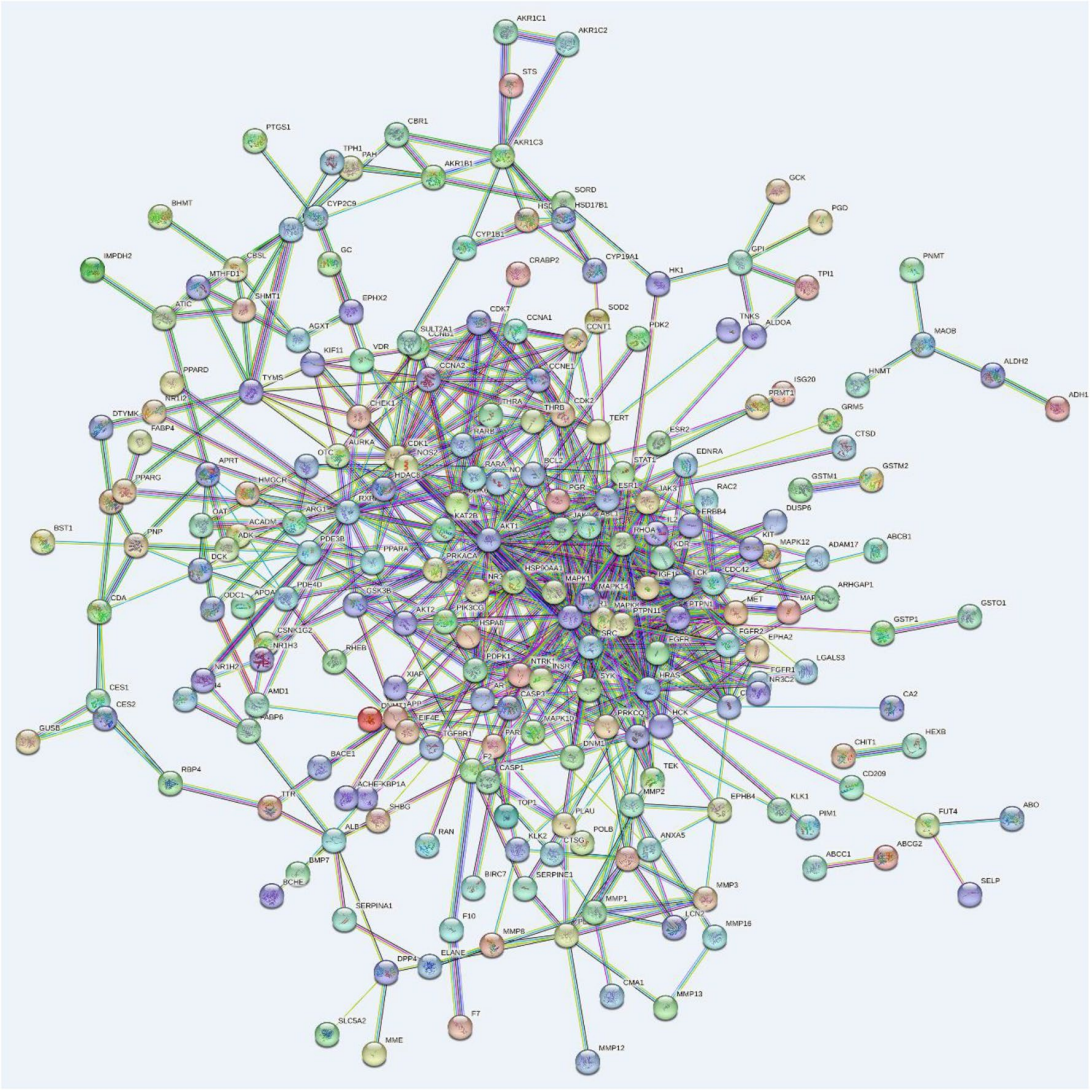
The PPI network.

**Figure 4:**
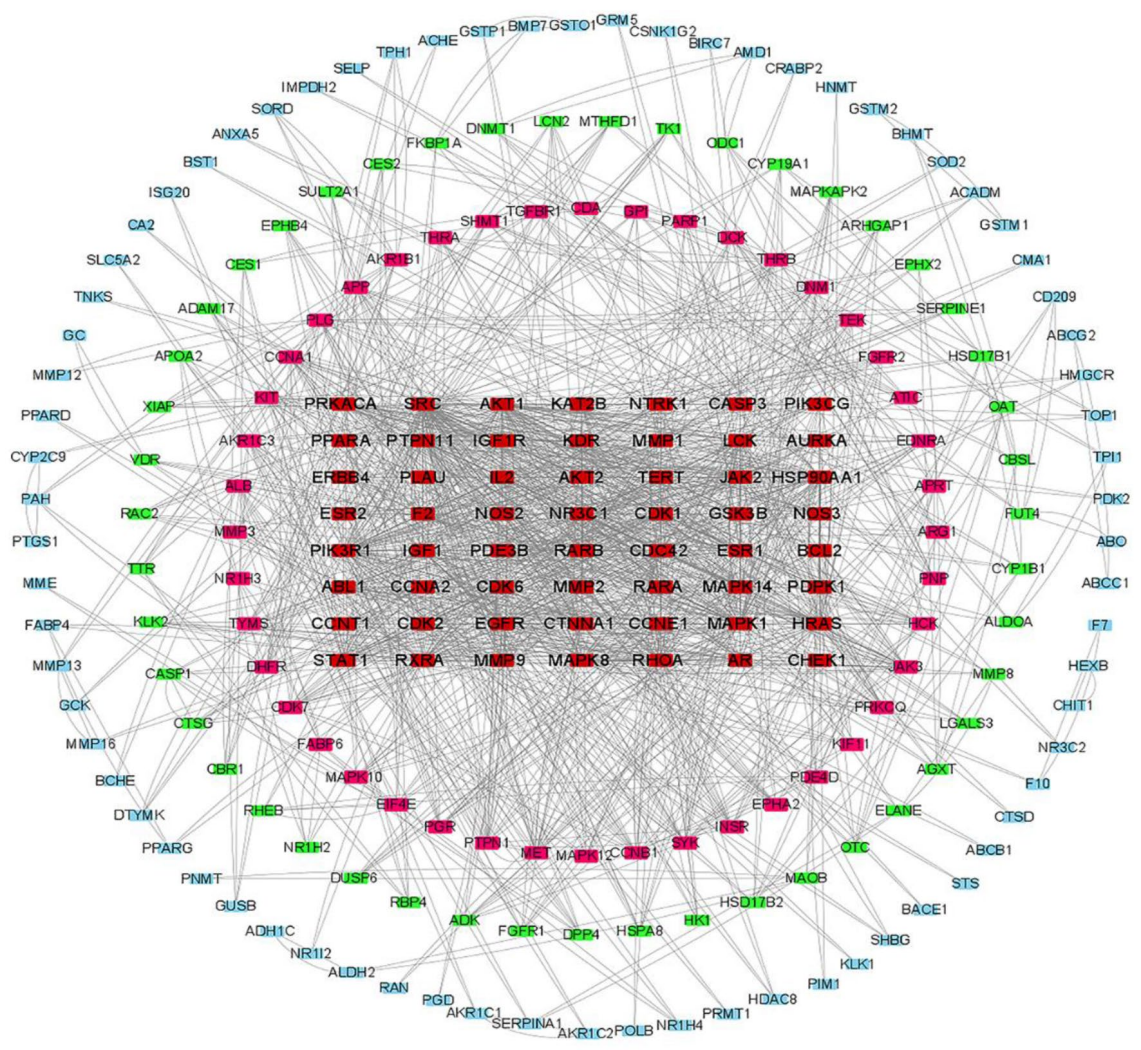
The network was constructed using Cytoscape3.8.0.

**Figure 5:**
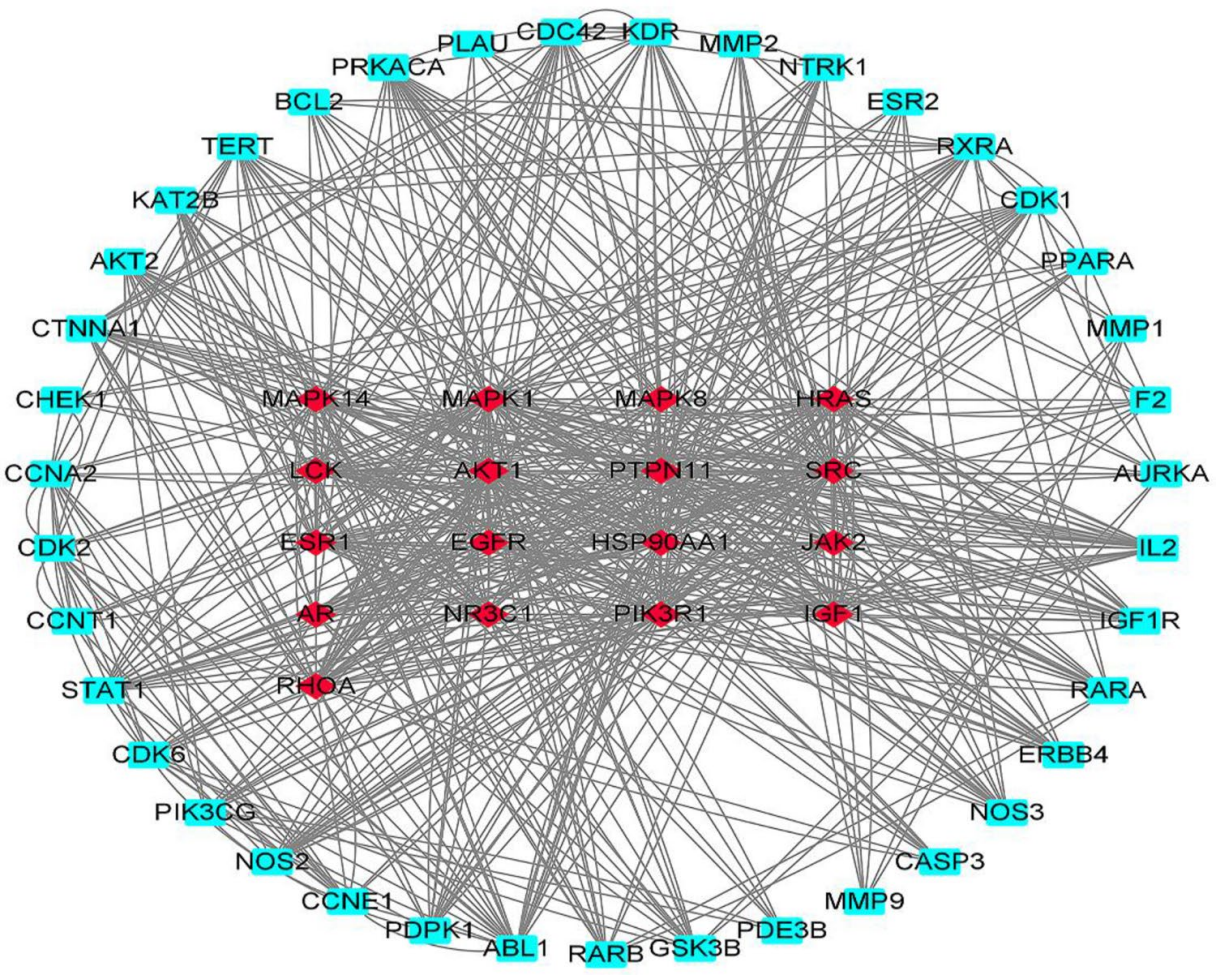
Hub genes were screened for the first time.

**Figure 6:**
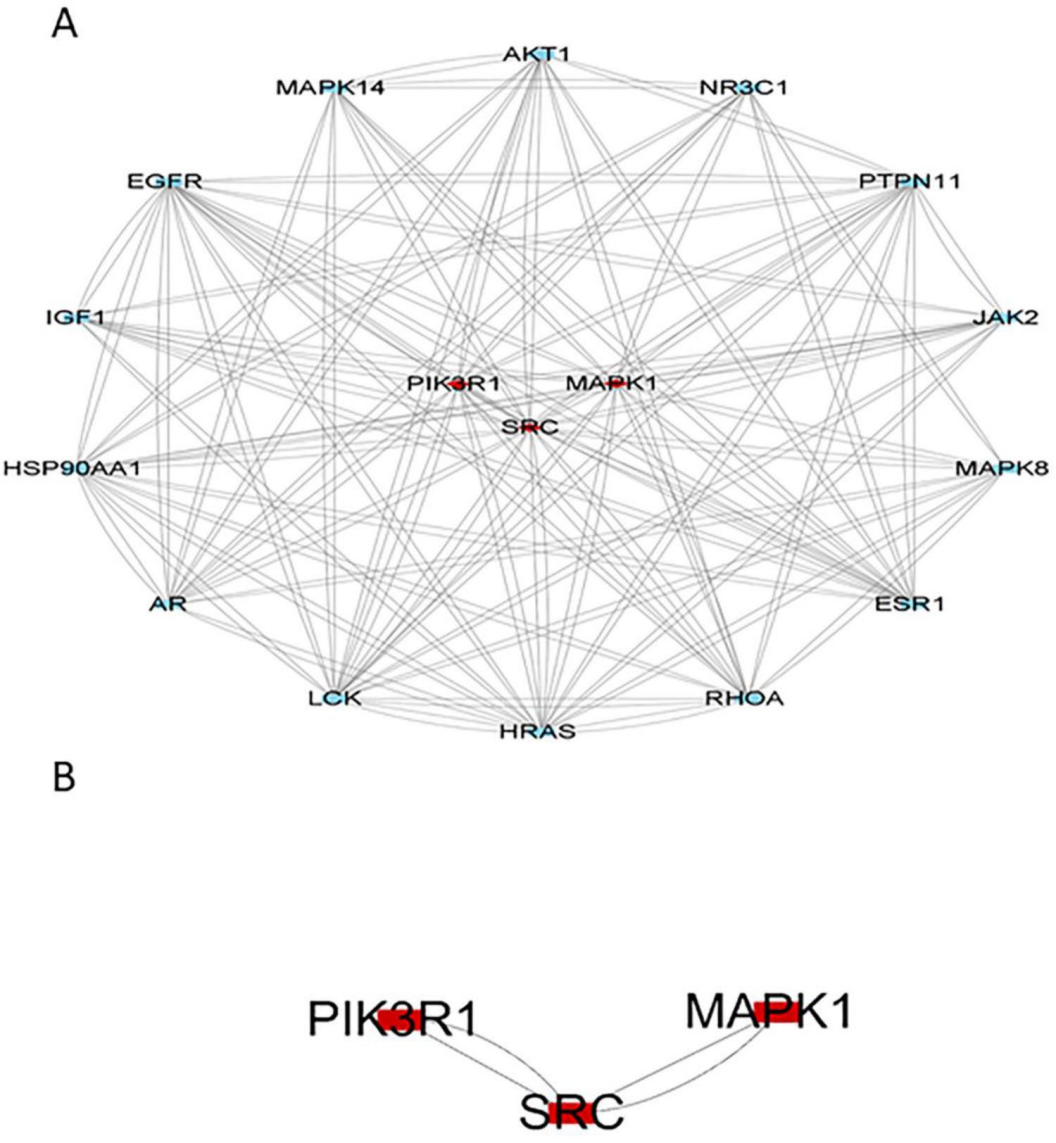
(**A**, **B**) Hub genes were screened, with SRC, PIK3R1 and MAPK1 showing the greatest correlation.

In total, 279 related central genes were imported into the STRING online database to obtain the PPI network. (Fig. 3) The network was then constructed using Cytoscape3.8.0, showing 1,446 edges and 221 nodes (Fig. 4). The hub genes were gradually screened through the median of each condition, including Betweenness, Closeness, Degree, Eigenvector, LAC, and Network (Fig. 5). Finally, the three hub genes (SRC, PIK3R1 and MAPK1) with the greatest correlation were identified (Fig. 6A,B) (Table 1).

**Table 1:** Hub Gene and its conditions.

Name	Betweenness	Closeness	Degree	Eigenvector	LAC	Network
MAPK1	316.145997	0.654761905	56	0.262427032	14.71428571	34.1395167
PIK3R1	156.879384	0.639534884	50	0.259732068	16	31.30592693
SRC	435.7180769	0.6875	60	0.293277085	17.33333333	40.36234958

### Functional analysis and molecular docking

GO analysis predicted the biological functions involved in the 279 overlapping genes. Among biological processes, the response to xenobiotic stimulus and regulation of inflammatory response was significantly involved in the treatment of BLCA with HE. In the CC, both,the vesicle lumen and cytoplasmic vesicle lumen were involved. In addition, significant concentrations of carboxylic acid binding and serine hydrolase activity were observed in MF. The significantly enriched biological functions were visualized by a circle diagram (Appendix Fig. 1 and Appendix Fig. 2). KEGG results revealed that the PI3K/AKT signaling pathway had the highest proportion of genes. Lipid and atherosclerosis, MAPK signaling pathway and chemical carcinogenesis—reactive oxygen species were also significantly associated with HE in BLCA (Appendix Fig. 3).

**Figure 7:**
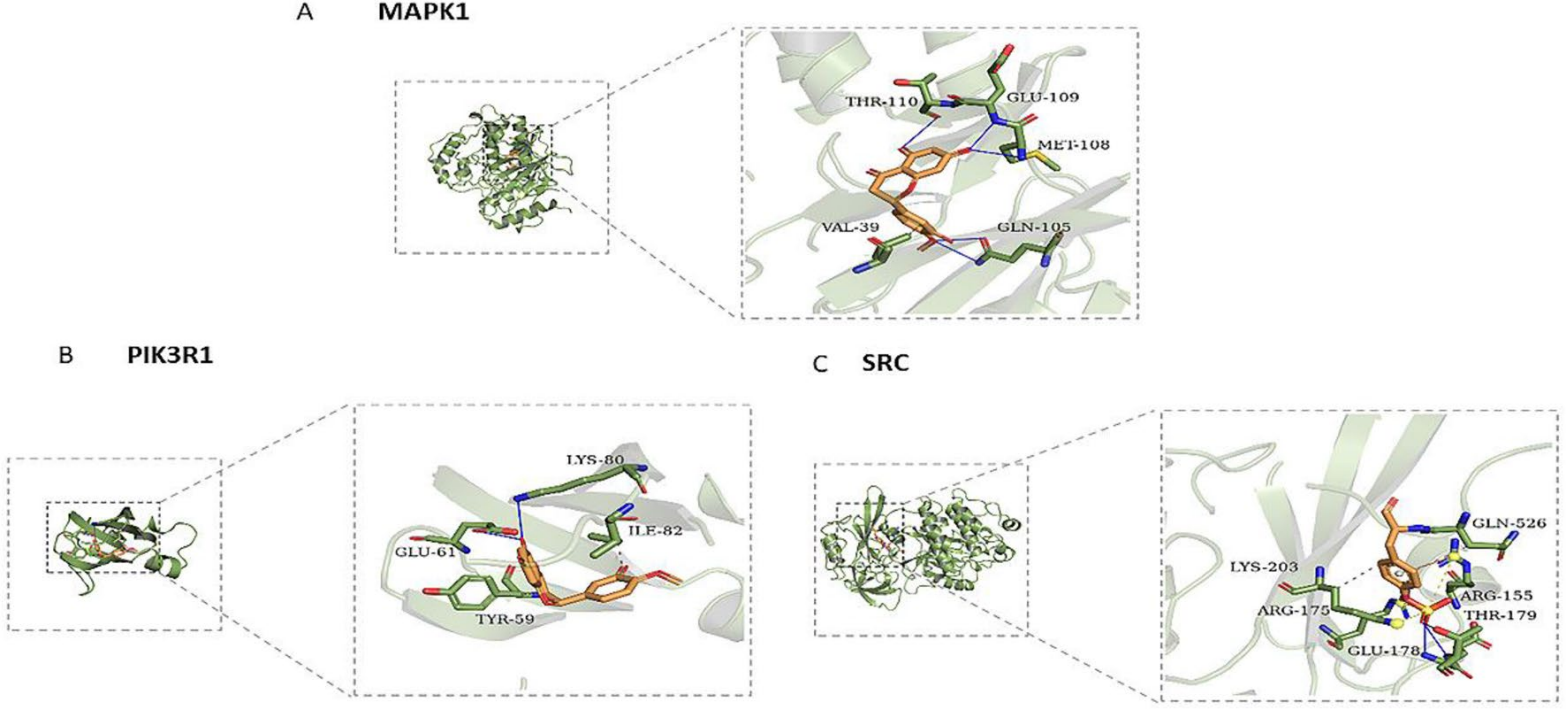
(**A**–**C**) The results of molecular docking of SRC, MAPK1 and PIK3R1.

The results of molecular docking showed three Hub proteins (SRC, MAPK1 and PIK3R1) with a strong interaction with HE. Their minimum binding energies were less than − 5 kcal/mol, indicating effective binding to HE (Table 2). The MAPK1 protein was found to interact with HE through amino acid residues, including THR-110, GLU-109, VAL-39 and GLN-105. The interaction between PIK3R1 and HE depended on LYS-80, GLU-61, TYR-59 and ILE-82. The interaction between SRC and HE required residues such as GLN-526, LYS-203, ARG-175, GLU-178, THR-179 and ARG155. (Fig. 7A–C).

**Table 2:** The molecular docking of SRC, MAPK1 and PIK3R1.

	Proteins	Affinity (kcal/mol)
Hesperetin	SRC	− 7.6
	MAPK1	− 7.3
	PIK3R1	− 6.3

### Effects of HE on BLCA cell proliferation

**Figure 8:**
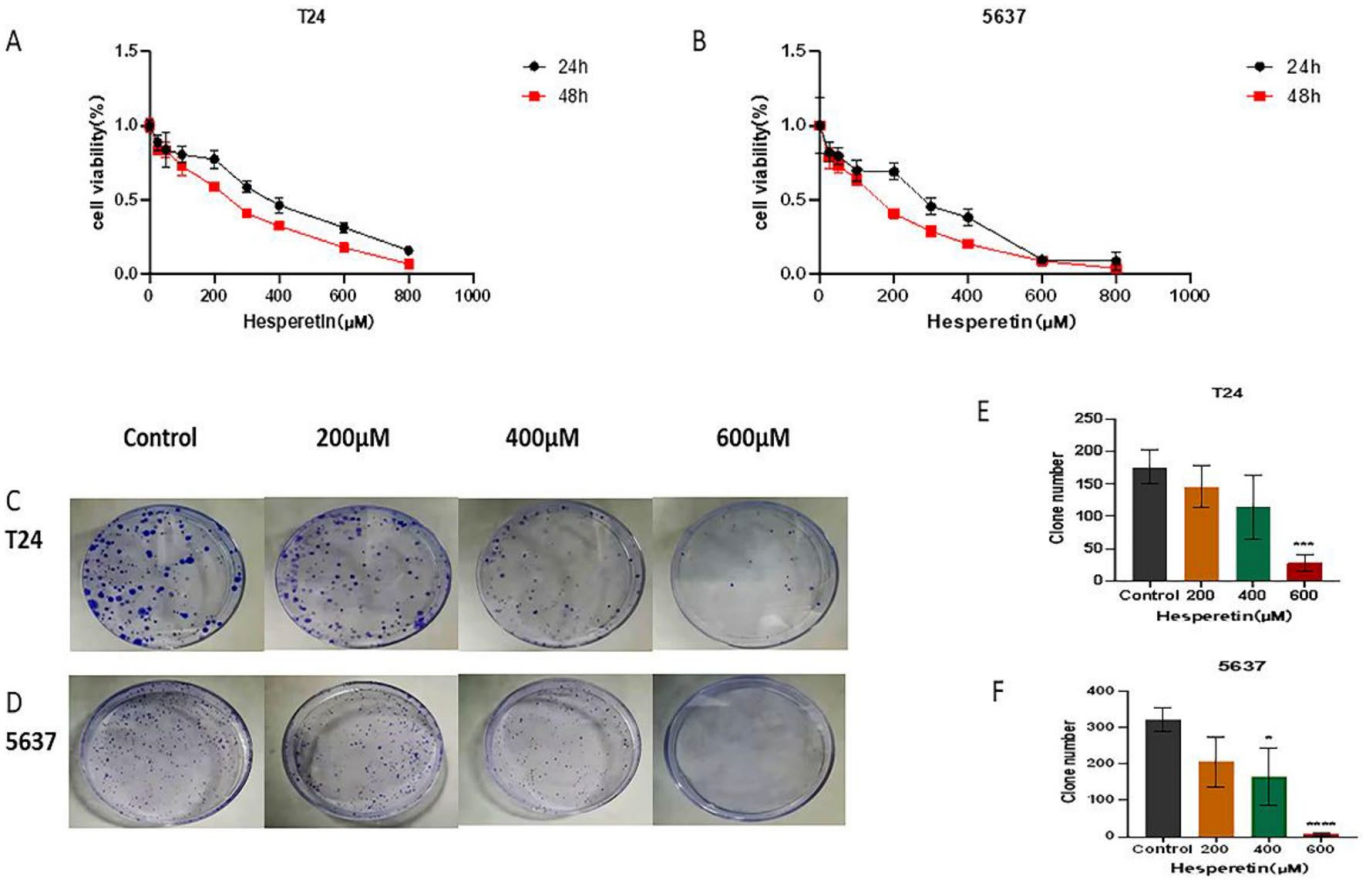
(**A**, **B**) The inhibition curve of T24 and 5637 cells under HE treatment was constructed by CCK8. (**C**, **D**) The results of colony-forming experiments on T24 and 5637 cells are shown. (**E**, **F**) The clone numbers of T24 and 5637 cells were significantly inhibited by HE.*p < 0,05; ** *p <* 0,01; ***p < 0,001; ****p < 0,0001.

To investigate the inhibitory effect of HE on the proliferation of BLCA cells, the CCK8 experiment was performed using eight concentration gradients (0, 25, 50, 100, 200, 300, 400, 600, 800 µM). The effect of HE intervention at 48 h was more pronounced than at 24 h (Fig. 8A,B). The IC50 of the T24 cells was472.0 µM at 24 h and 376.5 µM at 48 h. In contrast, the IC50 of the 5637cells was 370.4 µM at 24 h and 235.4 µM at 48 h. Table 3 displays the results of the repeated experiments. Most concentrations yielded IC50 between 200 and 600 µM. Therefore, the subsequent experiments were divided into four groups (control, 200 µM, 400 µM, and 600 µM). The colony-forming ability of T24 and 5637 cells showed a progressive decrease under different HE concentrations. (Fig. 8C,D). A significant decrease in the clone numbers of T24 cells was observed in the 600 µM HE treatment (*p <* 0.001). Similarly, the 5637 cells showed significant differences at 400 µM and 600 µM concentrations. (*p <* 0.05, *p <* 0.0001, respectively) (Fig. 8E,F) Therefore, HE inhibited the proliferation of BLCA cells.

**Table 3:** IC50 of HE on BLCA cells.

IC50 (µM)				
T24	24 h	449.9	472.0	492.8
	48 h	376.5	381.7	396.2
5637	24 h	328.1	369.9	370.4
	48 h	235.4	185.6	241.0

### HE inhibits BLCA cell migration

**Figure 9:**
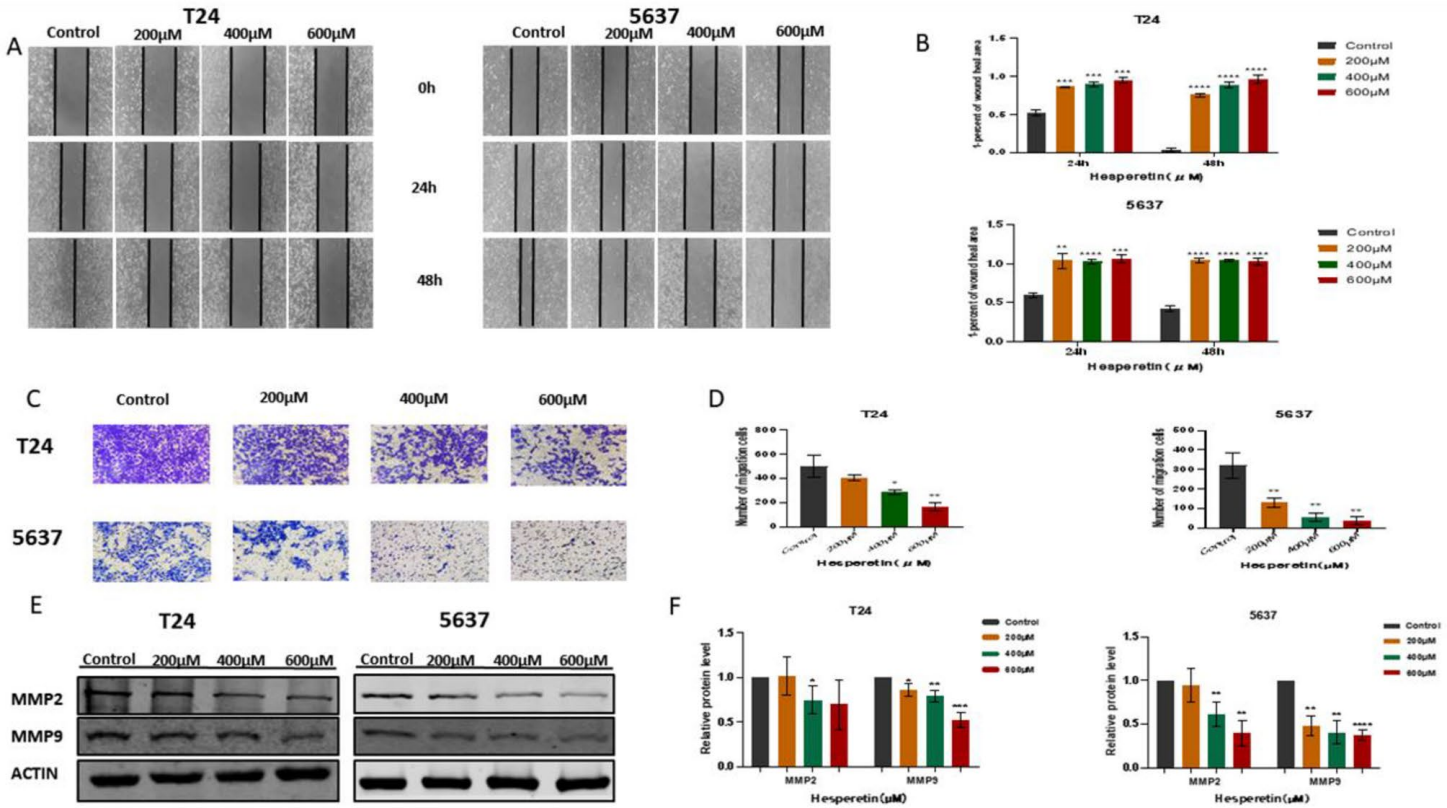
(**A**, **B**) The wound healing assay indicated that BLCA cell migration was inhibited by HE at 24 h and 48 h. (**C**, **D**) The transwell experiment also showed that HE inhibited the migration of BLCA cells. (**E**, **F**) MMP2/9 were significantly inhibited by HE. The protein gray ratio was quantified by histograms. *p < 0,05; ** *p <* 0,01; ***p < 0,001; ****p < 0,0001.

The wound healing assay showed that HE inhibited the migration of BLCA cells. As the HE concentration increased, stronger inhibition of BLCA cell migration was observed (Fig. 9A,B). In addition, similar results were obtained by the transwell test. The migration of BLCA cells was significantly reduced under 400 and 600 µM HE treatment (Fig. 9C,D). The protein expressions of MMP2 and MMP9 were also inhibited by HE (Fig. 9E,F). These results suggest that MMPs are involved in the pathway of tumor migration on which HE exerts its inhibitory effects.

### HE promotes BLCA cell apoptosis

**Figure 10:**
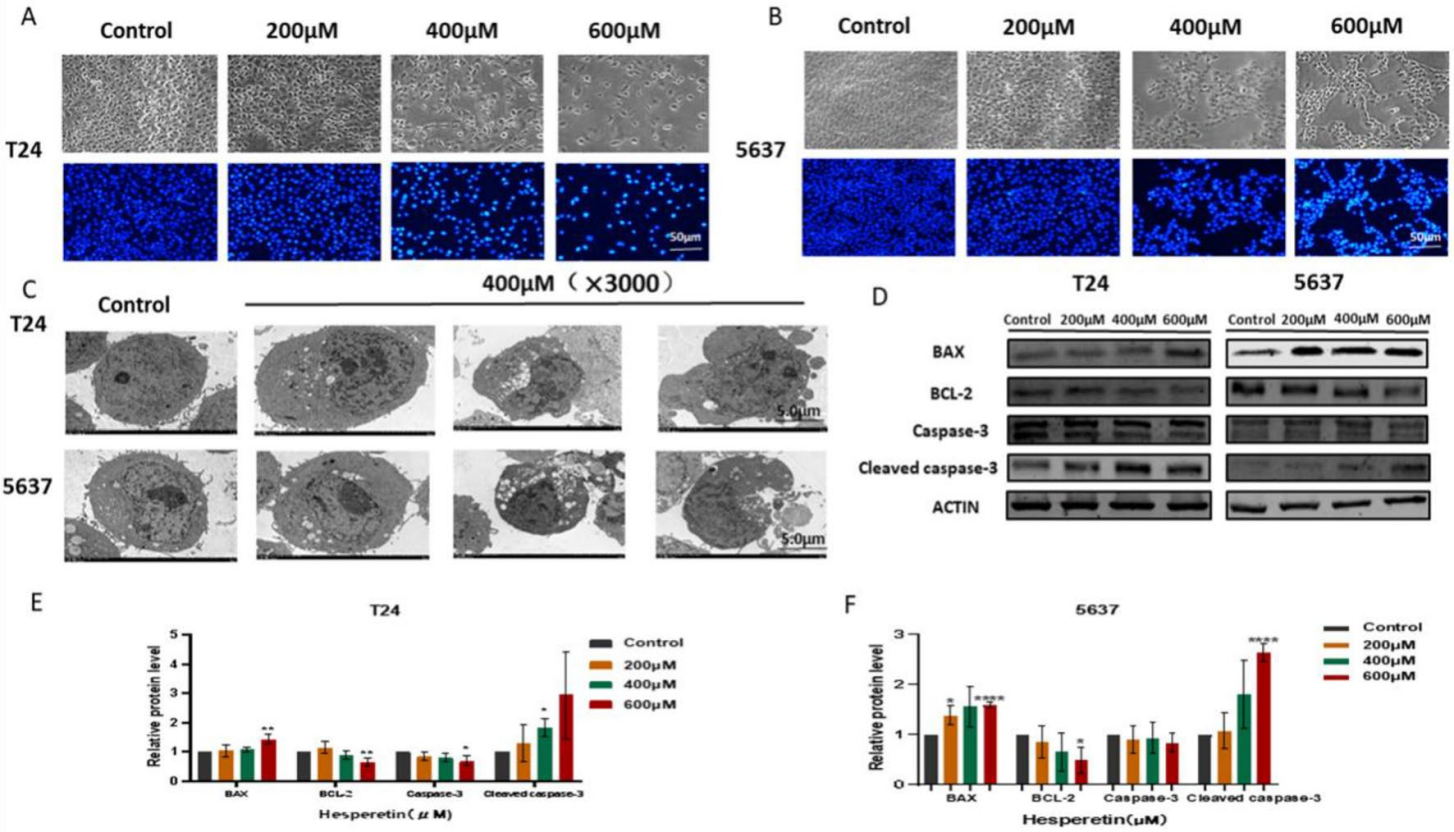
(**A**, **B**) HE induced the condensation of chromatin and shine by Hoechst staining. (**C**) TEM results showed that HE could induce chromatin condensation, nuclear fragmentation and the formation of apoptotic bodies in BLCA cells. (**D**) The expression of Bax, BCL-2 and Caspase-3 were detected by Weston Blot. (**E**, **F**) The protein gray ratio was quantified by histograms. *p < 0,05; ** *p <* 0,01; ***p < 0,001; ****p < 0,0001.

To investigate the effect of HE on BLA cell apoptosis, changes in cellular chromatin after 48 h under 200, 400, and 600 µM HE treatment were analyzed. The nuclei were stained by Hoechst staining, revealing that HE induced the condensation of chromatin and shine (Fig. 10A,B). This indicated that HE could lead to cell apoptosis. In addition, the cells were also observed under TEM (×3000), demonstrating not only the condensation of chromatin but also the formation of apoptotic bodies (Fig. 10C). Furthermore, the expression of apoptosis-related proteins such as Bax, BCL-2 and Caspase-3 were measured. The expression of Bax and cleaved caspase-3 were significantly up-regulated by HE in a dose-dependent way. In contrast, the expression of BCL-2 and caspase-3 was significantly inhibited by HE (Fig. 10 D). The differences were statistically significant (Fig. 10E,F).

### HE promotes BLCA cell ferroptosis

**Figure 11:**
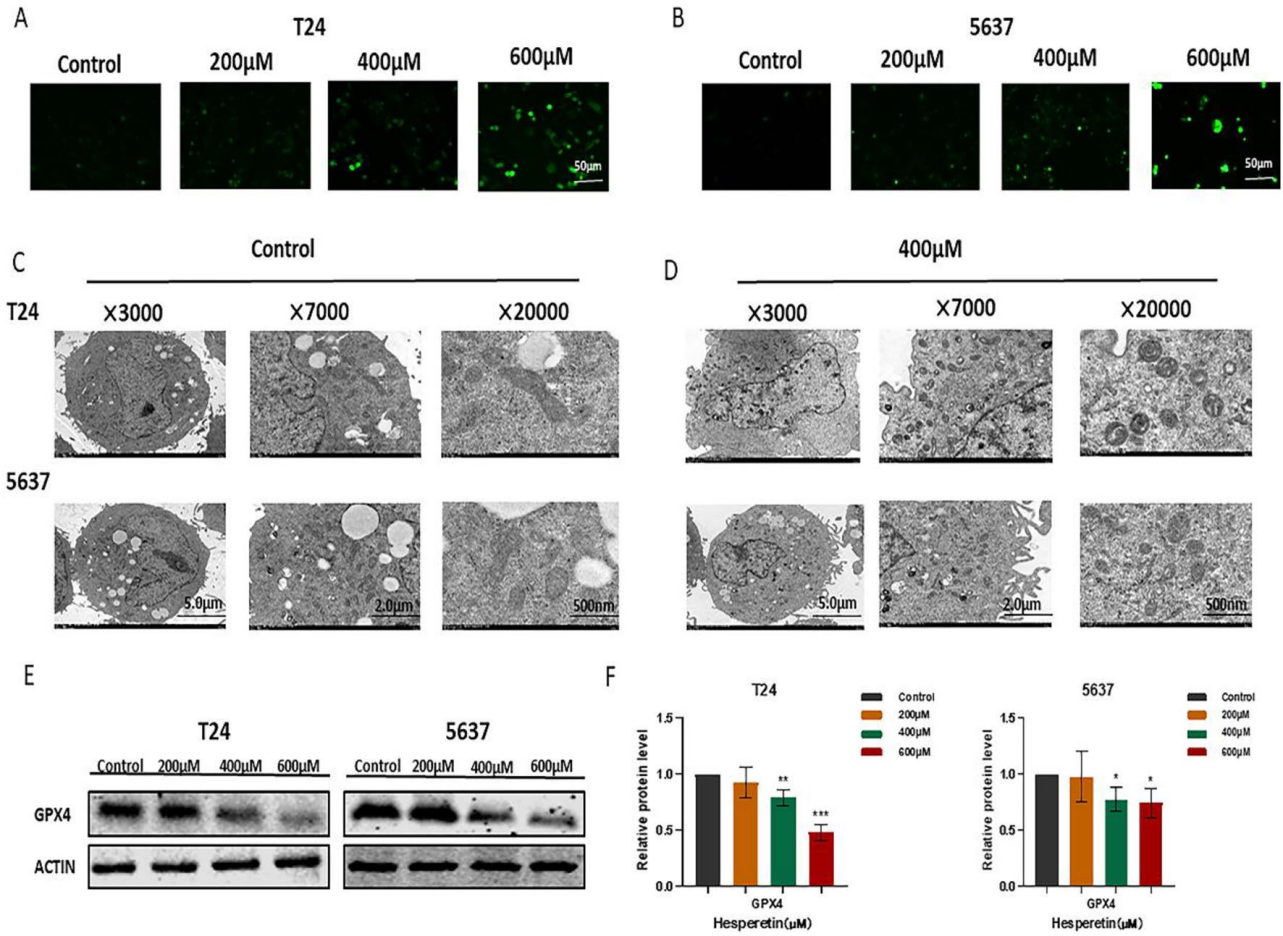
(**A**, **B**) The fluorescence intensity of ROS was detected under different concentrations of HE. (**C**, **D**) The morphology of BLCA cell mitochondria was observed by TEM (×3000, ×7000, ×20,000). (**E**, **F**) GPX4 expression was significantly inhibited by HE. *p < 0,05; ** *p <* 0,01; ***p < 0,001; ****p < 0,0001.

HE was found to increase ROS levels in BLCA cells (T24 and 5637). The fluorescence intensity increased gradually as the HE concentration was increased (Fig. 11A,B). In addition, TEM showed that HE resulted in decreased mitochondrial volume and increased membrane density in T24 and 5637 cells (Fig. 11C,D). GPX4 is an important regulator of ferroptosis in cancer cells^34^. HE was found to inhibit the expression of GPX4 in BLCA cells. In particular, GPX4 was significantly inhibited under the intervention of 600 µM HE (Fig. 11E,F).

### Hub genes and PI3K/AKT pathway analysis

**Figure 12:**
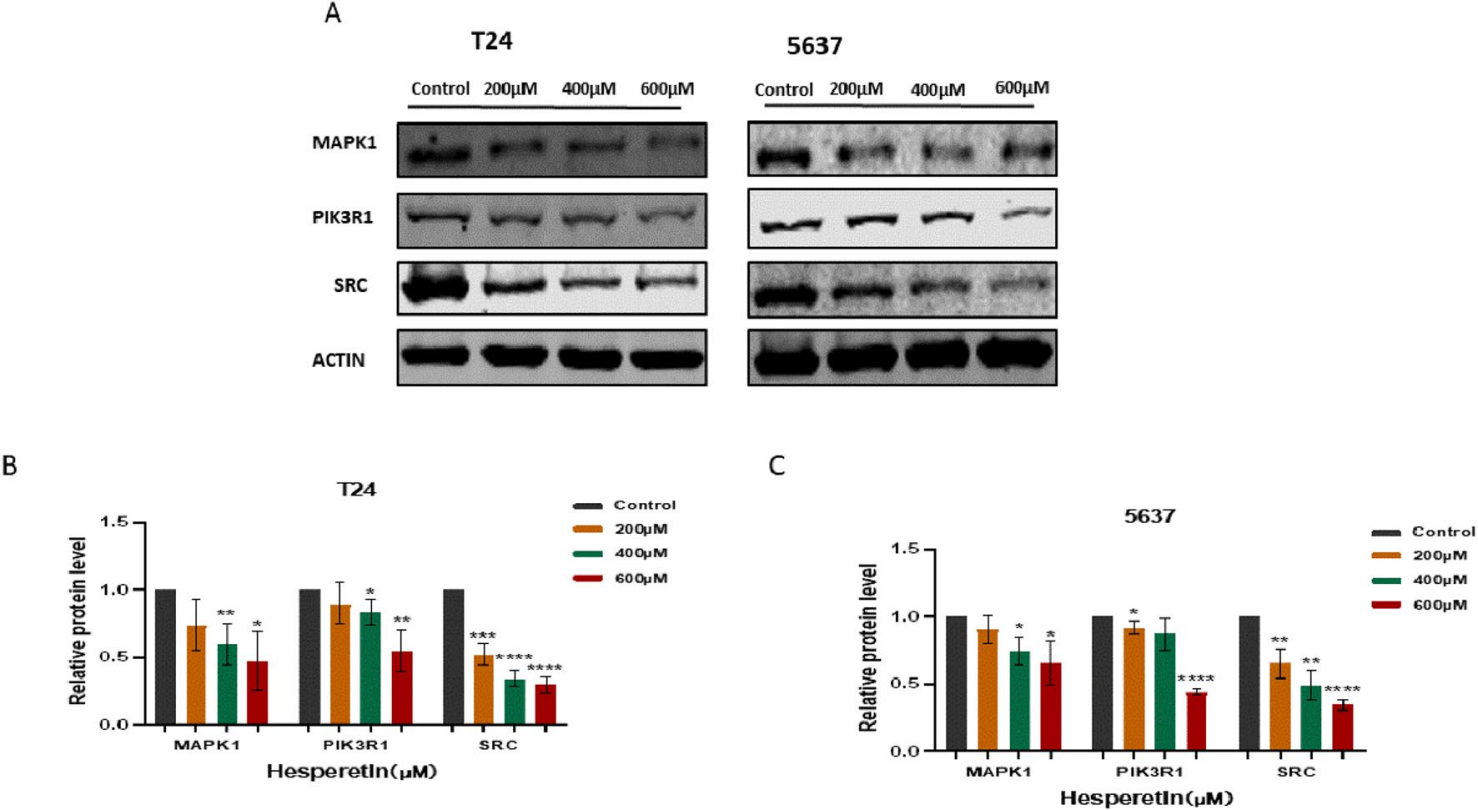
(**A**–**C**) The expression of MAPK1, SRC and PIK3R1 were significantly inhibited by HE. The protein gray ratio was quantified by histograms. *p < 0,05;** *p <* 0,01;***p < 0,001; ****p < 0,0001.

**Figure 13:**
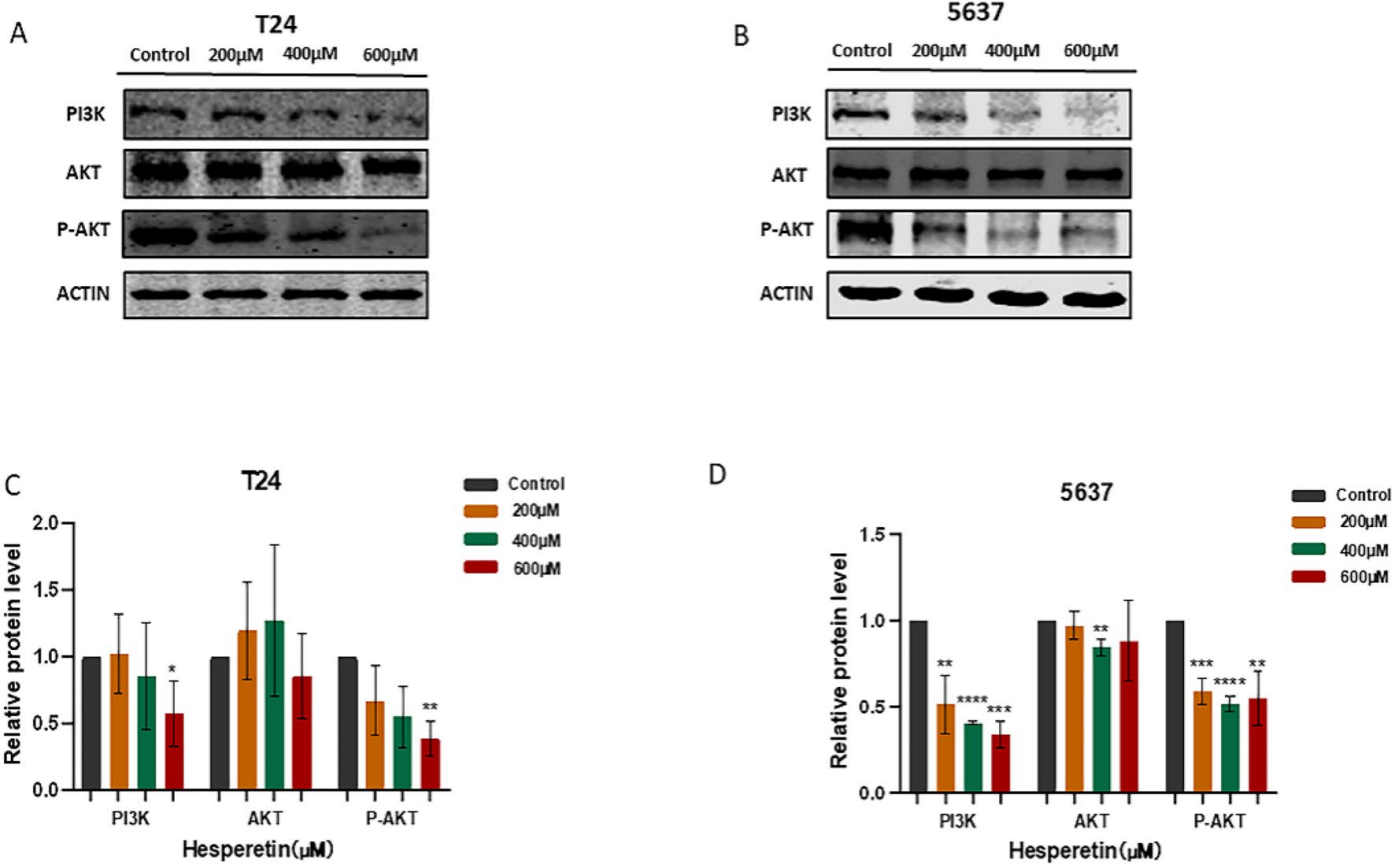
(**A**–**D**) The expression of PI3K AKT and p-AKT was detected by Western blotting. *p < 0,05, ** *p <* 0,01; ***p < 0,001; ****p < 0,0001.

The effect of HE on the hub genes was analyzed by Western blotting. The expression of MAPK1, SRC and PIK3R1 were significantly inhibited by HE in a dose-dependent manner (Fig. 12A–C). To further demonstrate the involvement of the PI3K/AKT pathway in HE treatment of BLCA, the pathway-related proteins were validated by Western blotting. The expression of AKT was not affected by HE. However, P-AKT and PI3K were inhibited by HE, demonstrating that the PI3K/AKT pathway was affected by HE in 5637 and T24 cells. (Fig. 13A–D).

## Discussion

The recurrence of BLCA is still high under the traditional treatment pleural, and many new treatments, including immunotherapy, are being developed^35–37^. Compounds derived from plants have recently gained popularity in the treatment of BLCA, and the development of new drugs based on natural compounds appears to be a recenttrend^38^. Hesperidin and Hesperetin, flavonoids derived from citrus fruits, were found to inhibit tumor cell proliferation in a variety of cancer models^39^. In addition, Hesperetin can inhibit the invasion and migration of breast cancer cells (MDA-MB-231) by regulating the FYN/PAXILLIN/RHOA axis^40^. Hesperetin also promotes apoptosis in prostate cancer cells by activating BAX and BAD expression^41^. For the first time, this study predicted the mechanism of HE against BLCA in terms of network pharmacology. Moreover, the molecular docking model was used to demonstrate the interaction between drugs and proteins. HE not only inhibited the proliferation of BLCA cells, but also promoted apoptosis and ferroptosis.

SRC, PIK3R1 and MAPK1 were identified as hub genes in the mechanism and played important biological functions. SRC expression was higher in BLCA samples. However, it was higher in lower grade BLCA than in higher grade lesions^42^. Patients with high SRC expression had poorer survival when treated with immune checkpoint inhibitors^43^. SRC is a tyrosine kinase that plays a role in a variety of biological signals, including PI3K/AKT, MAPK, epithelial-mesenchymal transition (EMT) and other signaling pathways. It has also been shown to be overexpressed in many cancers and is thought to be associated with tumor invasion and metastasis^44,45^. SRC expression was inhibited in BLCA cells, promoting cell apoptosis and decreasing cell proliferation^46^. Similarly, HE was found to suppress SRC expression in our study. The specific mechanisms and biological functions of SRC in BLCA should be further studied. MAPK1 (ERK2) and MAPK3 (ERK1) are key nodes of biochemical signals in biology, especially the RAF/MEK/ERK axis, which has an important effect on malignant transformation and drug resistance of tumor cells^47^. HE inhibitedMAPK1 expression, further suggesting that HE was involved in the ERK1/2 signal pathway in anti-BLCA. PI3K is composed of the P110 catalytic subunit and the P85 regulatory subunit, which are involved in cell growth and proliferation^48^. HE was found to inhibit the expression of PIK3R1 in a dose-dependent manner.

In addition, KEGG analysis showed that the PI3K/AKT pathway was involved in the anti-BLCA mechanism of HE, which was demonstrated experimentally. The PI3K/AKT pathway not only plays an essential role in the proliferation, differentiation and metastasis of cancer cells but is also involved in cell apoptosis and ferroptosis^49,50^. Intrinsic apoptosis, also known as the mitochondrial apoptosis pathway, activates pro-apoptotic proteins such as Bax, Bak, etc. They damage the mitochondrial membrane and finally activate apoptosis by caspase3/6/7^51^. Apoptosis has been known for years and is a major mechanism used by anticancer drugs such as cisplatin^52^. HE exerted a similar effect on BLCA cells by promoting apoptosis with proteins such as Baxand cleaved-caspase-3. ROS caused lipid peroxidation to attack the cell membrane, leading to many types of cell death, including apoptosis and ferroptosis^53^. The loss of GPX4 activity isthe key to cell ferroptosis^54^. HE inhibited the expression of GPX4 protein. At the same time, ROS was significantly activated, and mitochondrial atrophy was observed by transmission electron microscopy suggesting that ferroptosis was involved in anti-BLCA. In thisstudy, the effect of HE was not verified in vivo studies with an animal model. We will verify it in the future.

## In conclusion

HE was found to inhibit the proliferation and migration of BLCA cells and promote cell apoptosis and ferroptosis. As a natural compound, HE is expected to be a new approach to BLCA treatment.

### Supplementary Information

Supplementary Information 1.

Supplementary Information 2.

Supplementary Legends.

Supplementary Figure 1.

Supplementary Figure 2.

Supplementary Figure 3.

## Data Availability

The data supported the study could be available at online databases including STRING (https://string-db.org/), Uniprot (http://www.uniprot.org/), SwissTargetPrediction database (http://www.swisstargetprediction.ch/), GeneCards (https://www.genecards.org), OMIM (https://www.omim.org), PharmGkb (https://www.pharmgkb.org), TTD (http://db.idrblab.net/ttd/), DrugBank (https://www.drugbank.ca/), The protein–ligand interaction profiler (PLIP) website (https://plip-tool.biotec.tu-dresden.de/plip-web/plip/index). The 2D/3D structures of the compounds in Fig. (5.6.7 and 8) were obtained from the Pubchem database (https://pubchem.ncbi.nlm.nih.gov/).

## Abbreviations

BLCA: Bladder cancer
ROS: Reactive oxygen species
HE: Hesperetin
TEM: Transmission electron microscope
NMIBC: Non-muscle invasive BLCA
MIBC: Muscle invasive BLCA
TURBT: Transurethral resection of bladder tumor
PPI: Protein–protein interaction
BP: Biological process
CC: Cellular component
MF: Molecular functions
PLIP: Protein–ligand interaction profiler
ATCC: American type culture collection
CCK8: Cell counting Kit 8
ICT: Immune checkpoint
EMT: Epithelial-mesenchymal transition
