# Supplementary figures and images for: Hesperetin promotes bladder cancer cells death via the PI3K/AKT pathway by network pharmacology and molecular docking (part 2 of 2)
Source: Sci Rep. 2024 Jan 10;14:1009. doi: 10.1038/s41598-023-50476-8 (PMC10781778; doi:10.1038/s41598-023-50476-8)

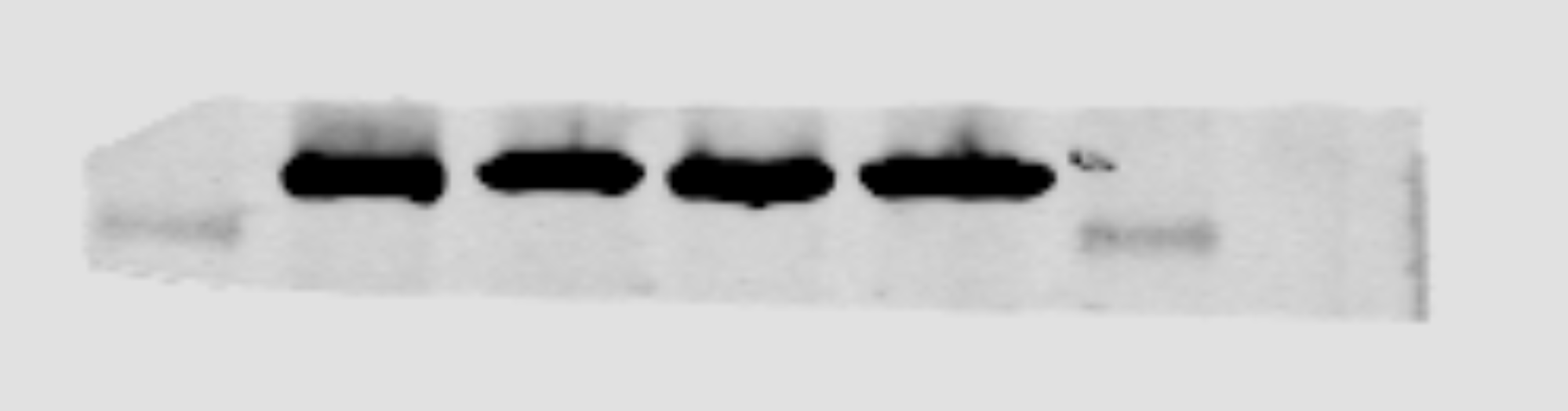

Supplement: Supplementary file 2 — Supplementary Information 2. [file 41598_2023_50476_MOESM2_ESM.zip › protein/2 repeat/4.targets/T24/ACTIN.tif]

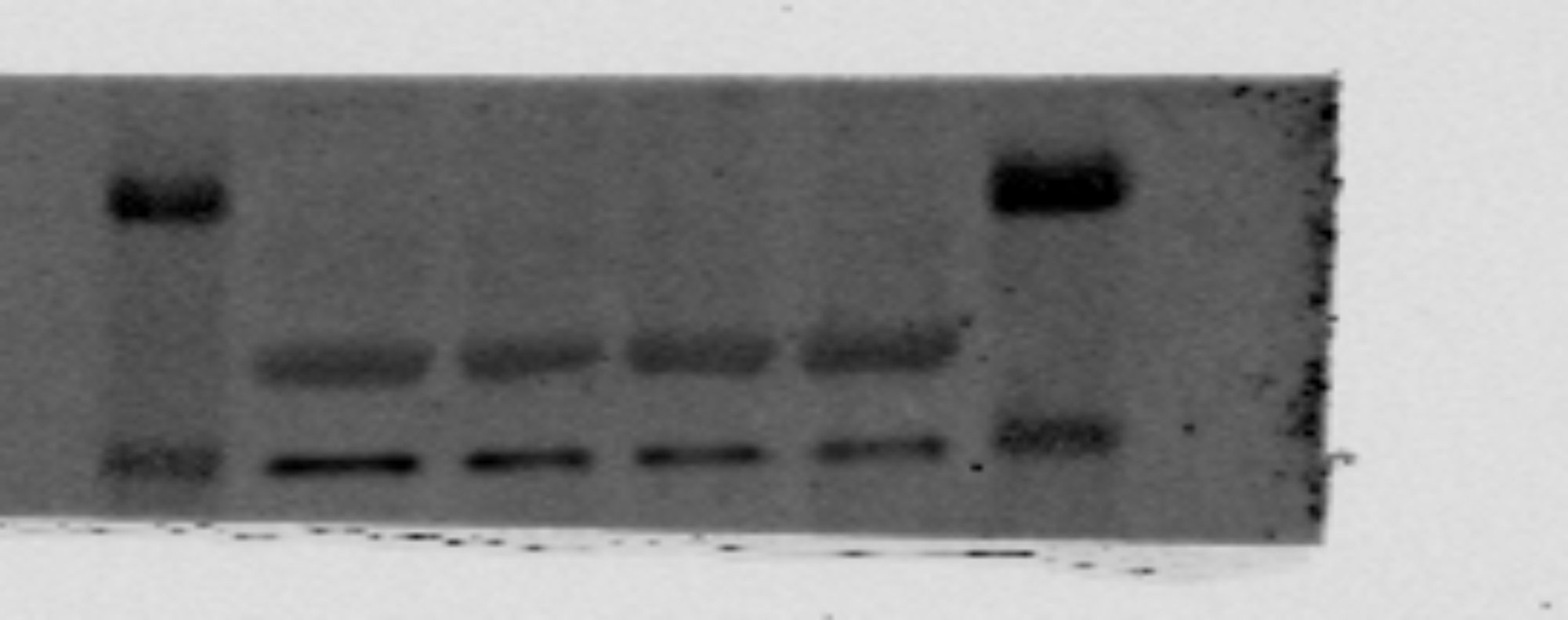

Supplement: Supplementary file 2 — Supplementary Information 2. [file 41598_2023_50476_MOESM2_ESM.zip › protein/2 repeat/4.targets/T24/MAPK1.tif]

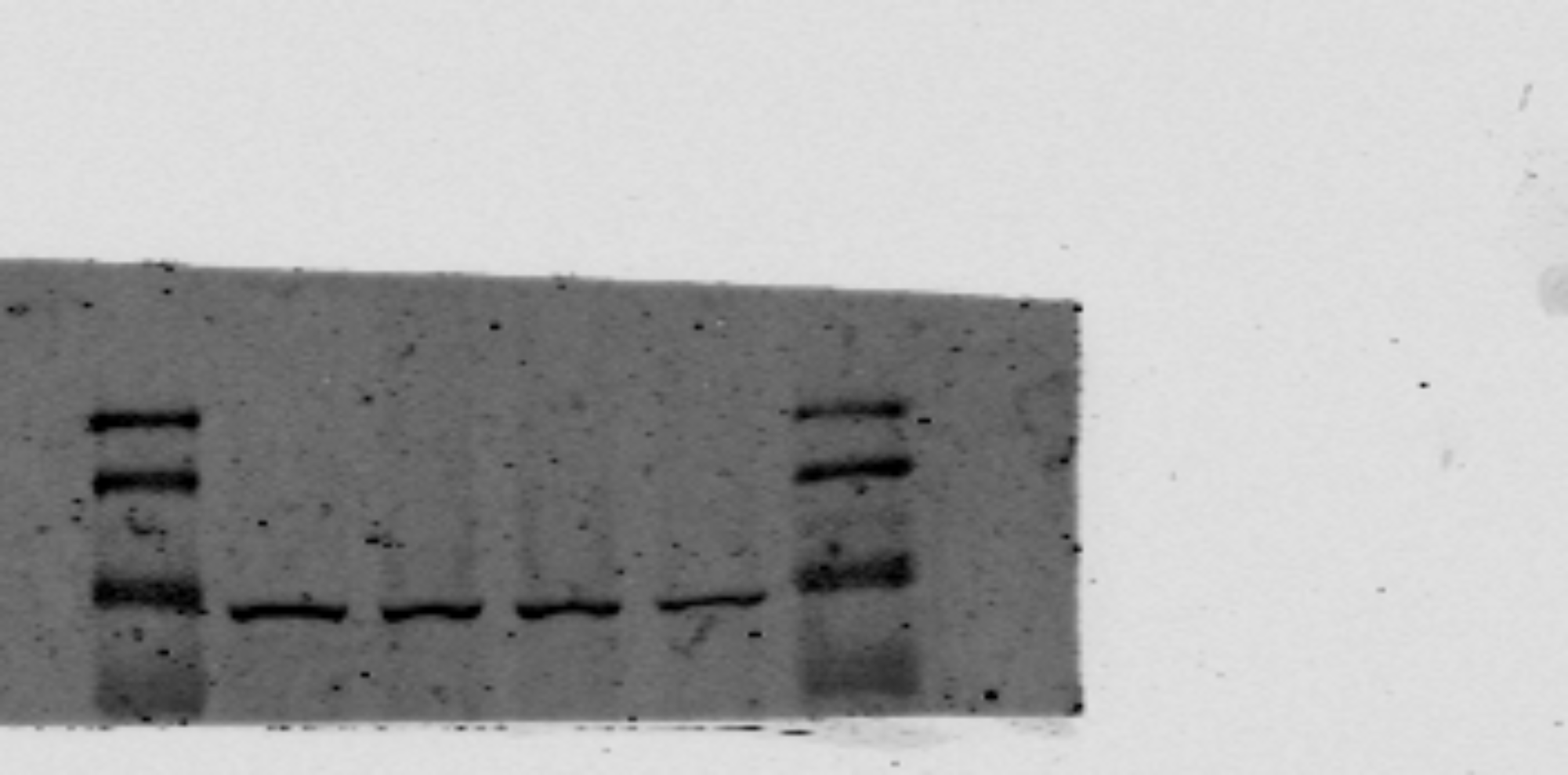

Supplement: Supplementary file 2 — Supplementary Information 2. [file 41598_2023_50476_MOESM2_ESM.zip › protein/2 repeat/4.targets/T24/P85.tif]

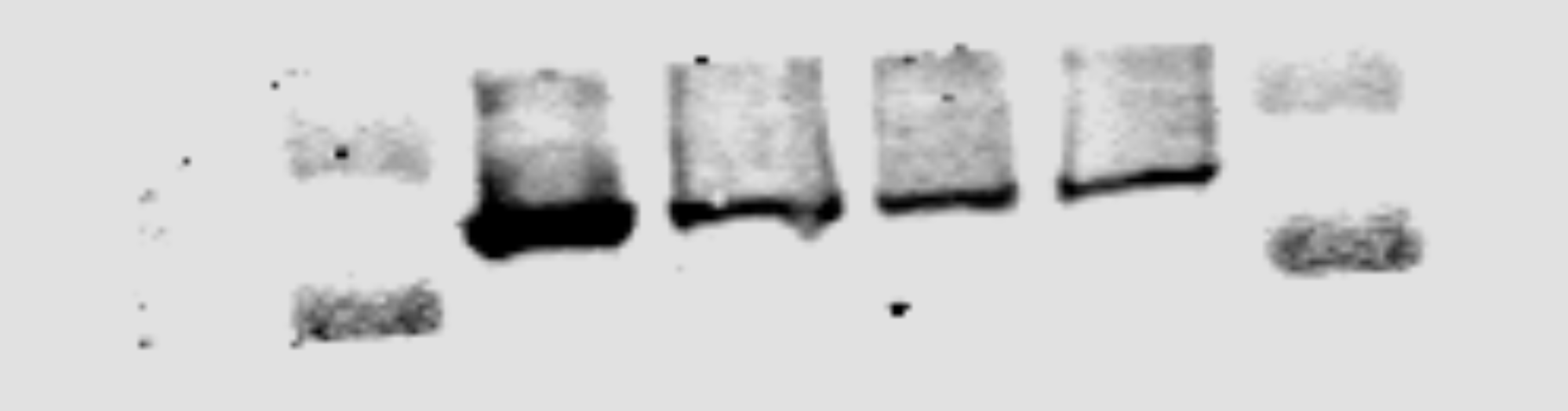

Supplement: Supplementary file 2 — Supplementary Information 2. [file 41598_2023_50476_MOESM2_ESM.zip › protein/2 repeat/4.targets/T24/SRC.tif]

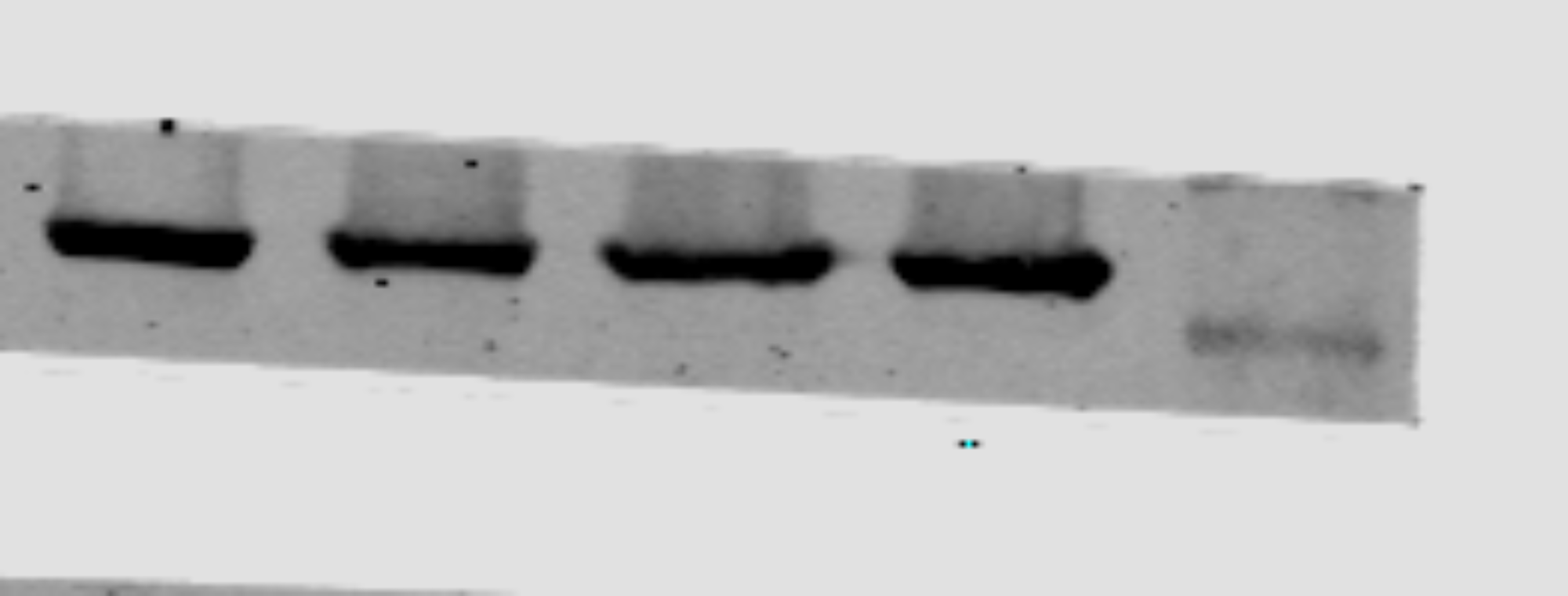

Supplement: Supplementary file 2 — Supplementary Information 2. [file 41598_2023_50476_MOESM2_ESM.zip › protein/2 repeat/5.pathway/5637/ACTIN.tif]

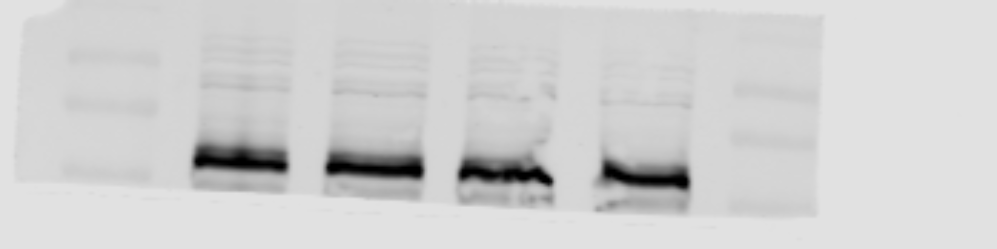

Supplement: Supplementary file 2 — Supplementary Information 2. [file 41598_2023_50476_MOESM2_ESM.zip › protein/2 repeat/5.pathway/5637/AKT.png]

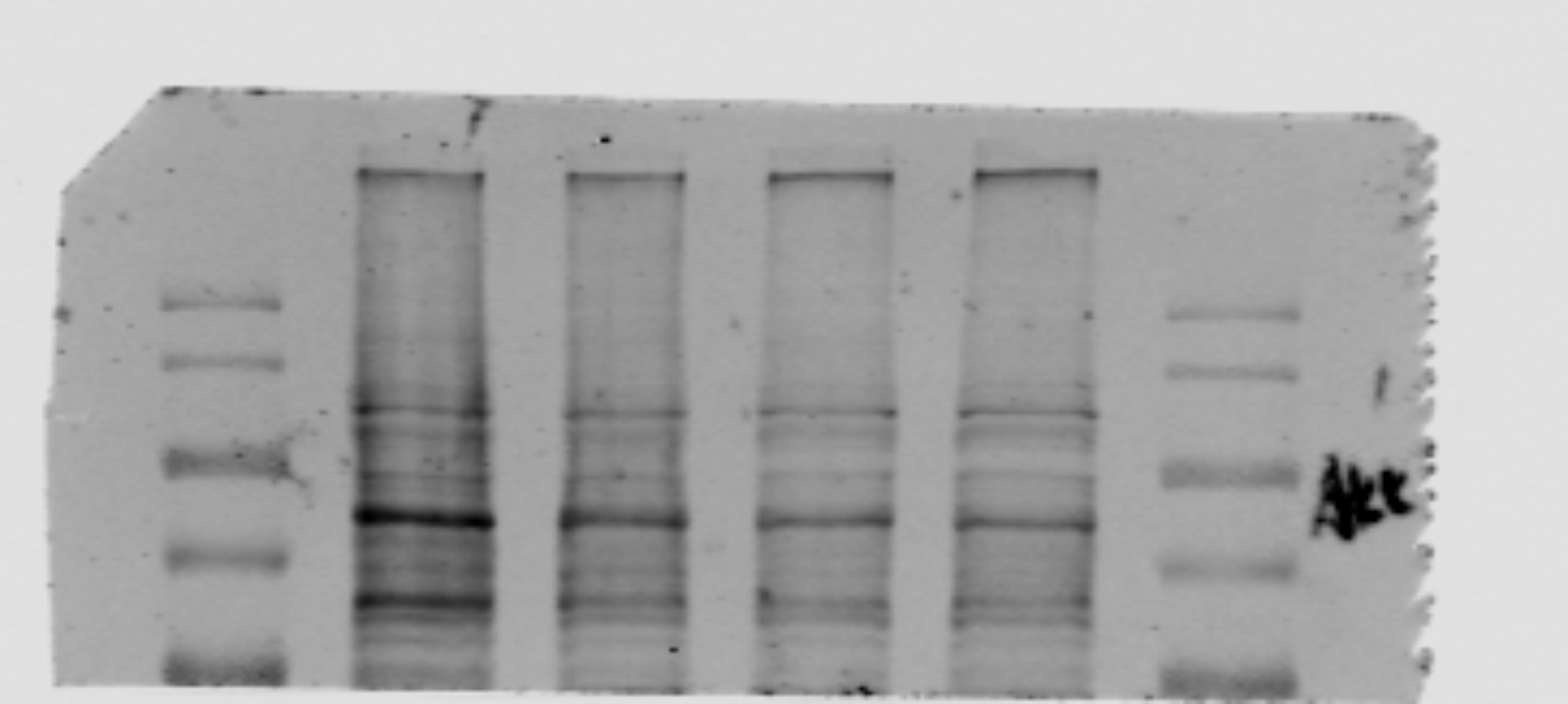

Supplement: Supplementary file 2 — Supplementary Information 2. [file 41598_2023_50476_MOESM2_ESM.zip › protein/2 repeat/5.pathway/5637/PAKT.png]

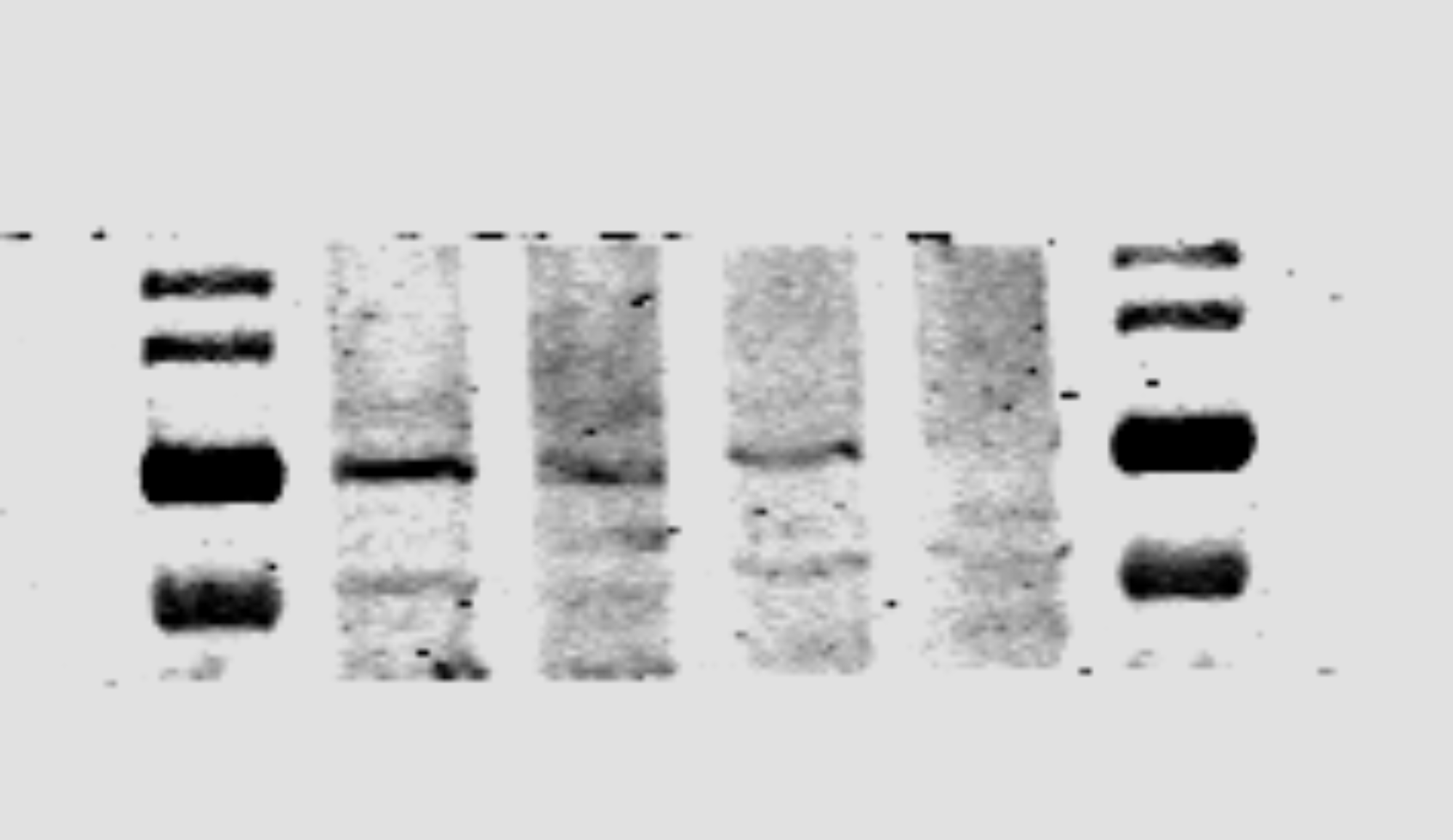

Supplement: Supplementary file 2 — Supplementary Information 2. [file 41598_2023_50476_MOESM2_ESM.zip › protein/2 repeat/5.pathway/5637/PI3K.tif]

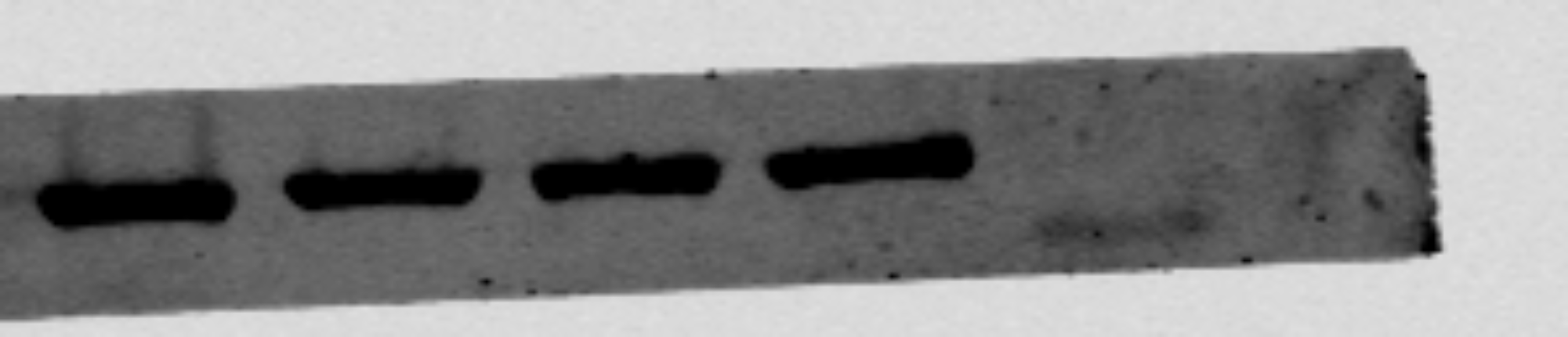

Supplement: Supplementary file 2 — Supplementary Information 2. [file 41598_2023_50476_MOESM2_ESM.zip › protein/2 repeat/5.pathway/T24/ACTIN.tif]

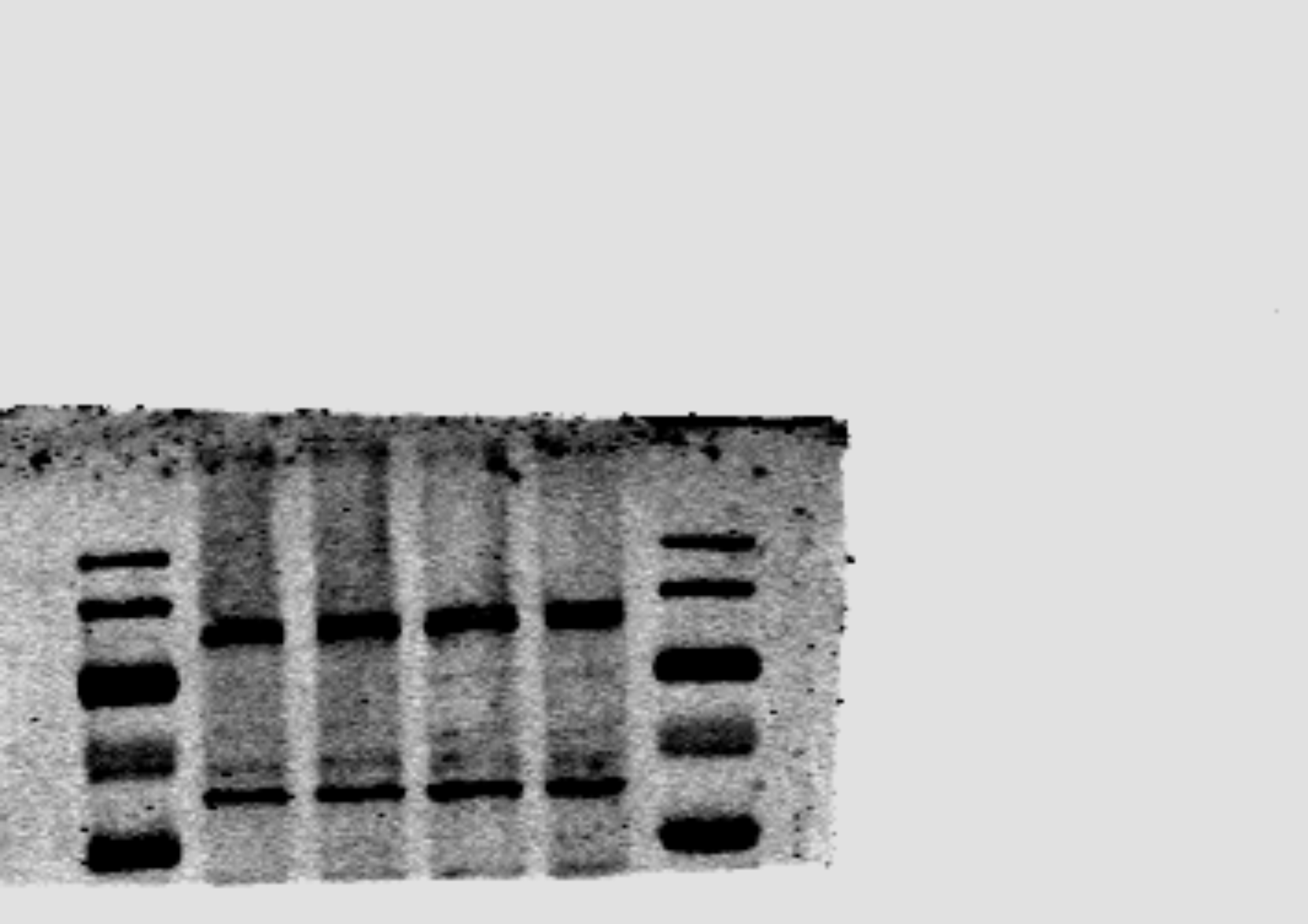

Supplement: Supplementary file 2 — Supplementary Information 2. [file 41598_2023_50476_MOESM2_ESM.zip › protein/2 repeat/5.pathway/T24/AKT.tif]

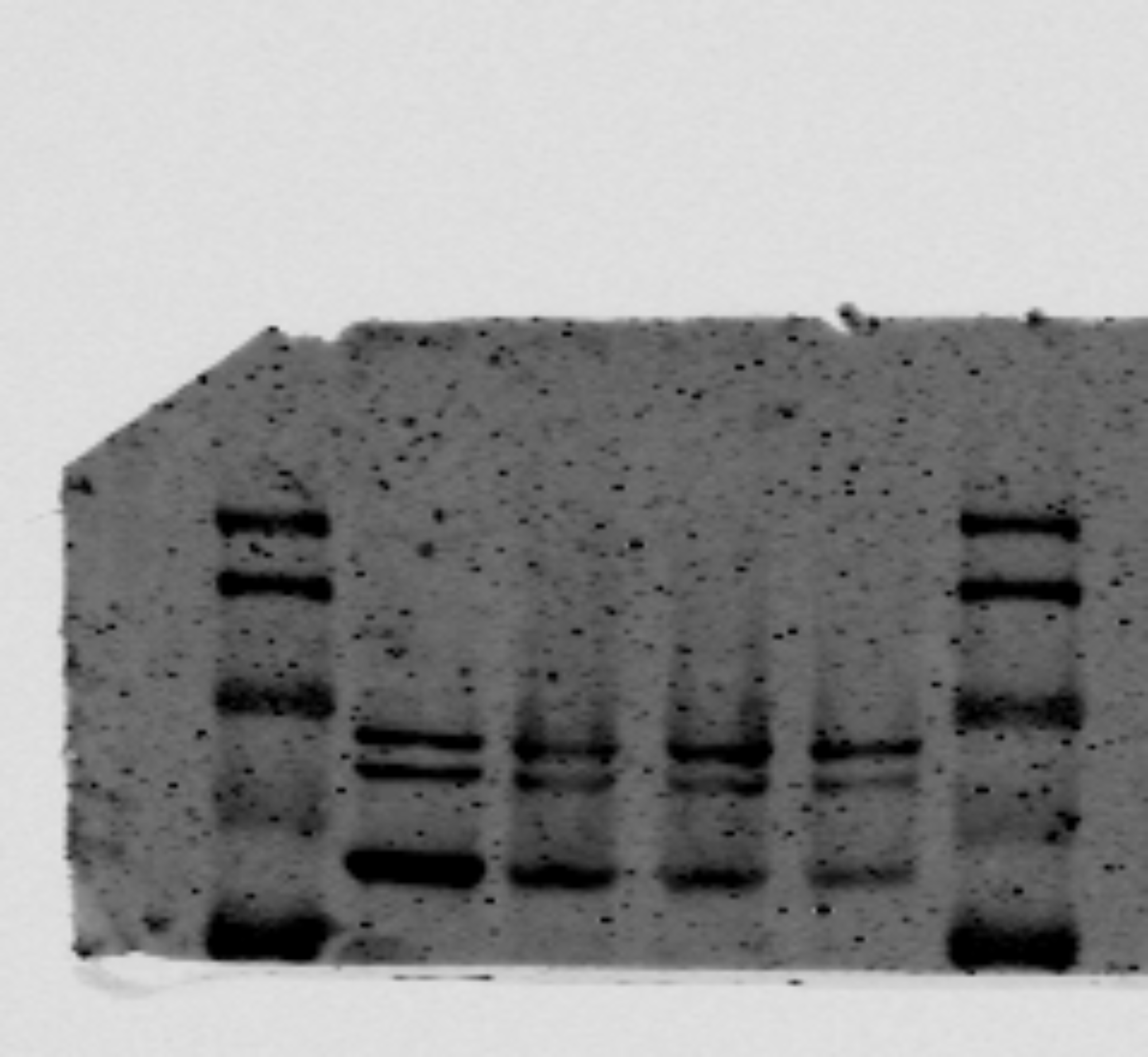

Supplement: Supplementary file 2 — Supplementary Information 2. [file 41598_2023_50476_MOESM2_ESM.zip › protein/2 repeat/5.pathway/T24/PAKT.tif]

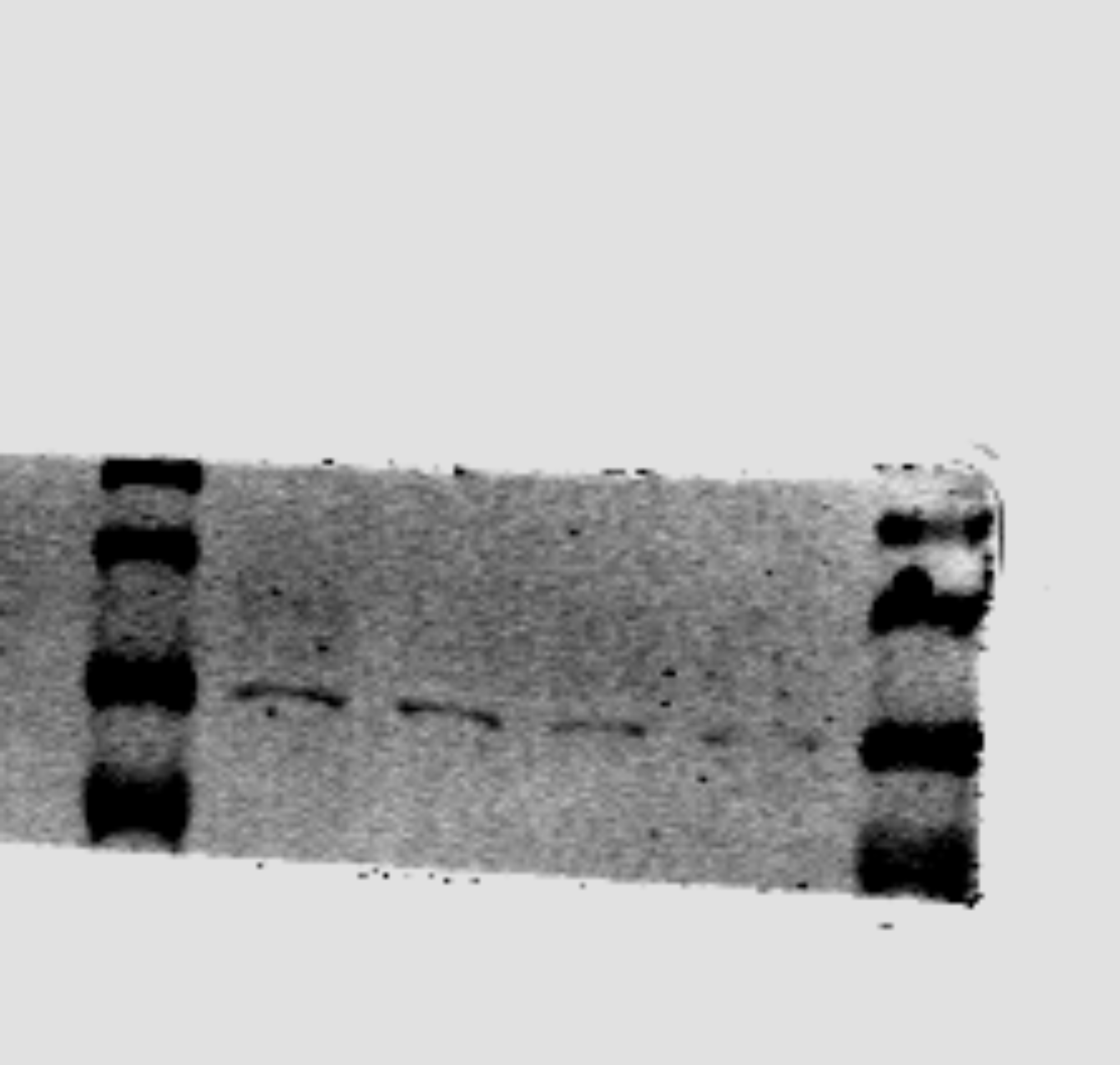

Supplement: Supplementary file 2 — Supplementary Information 2. [file 41598_2023_50476_MOESM2_ESM.zip › protein/2 repeat/5.pathway/T24/PI3K.tif]

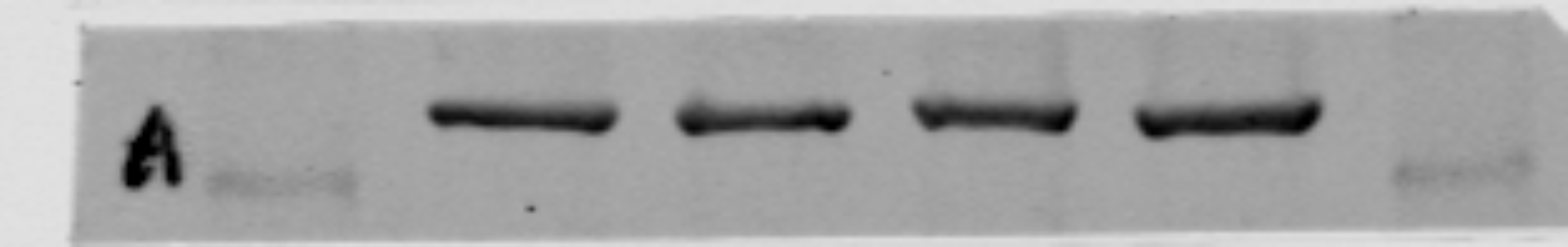

Supplement: Supplementary file 2 — Supplementary Information 2. [file 41598_2023_50476_MOESM2_ESM.zip › protein/3 repeat/1.magration/5637/24.tif]

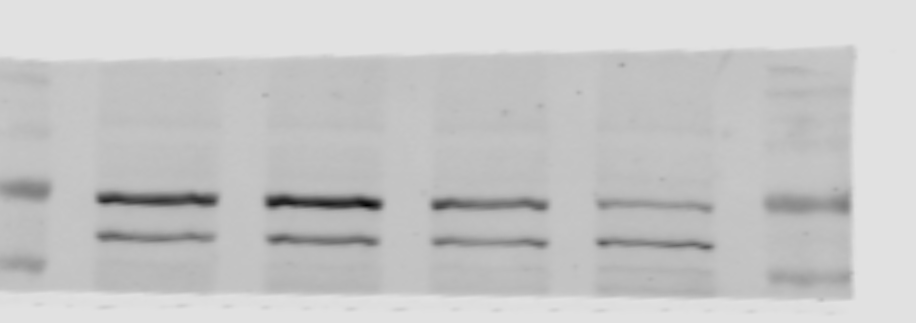

Supplement: Supplementary file 2 — Supplementary Information 2. [file 41598_2023_50476_MOESM2_ESM.zip › protein/3 repeat/1.magration/5637/MMP2Cut.png]

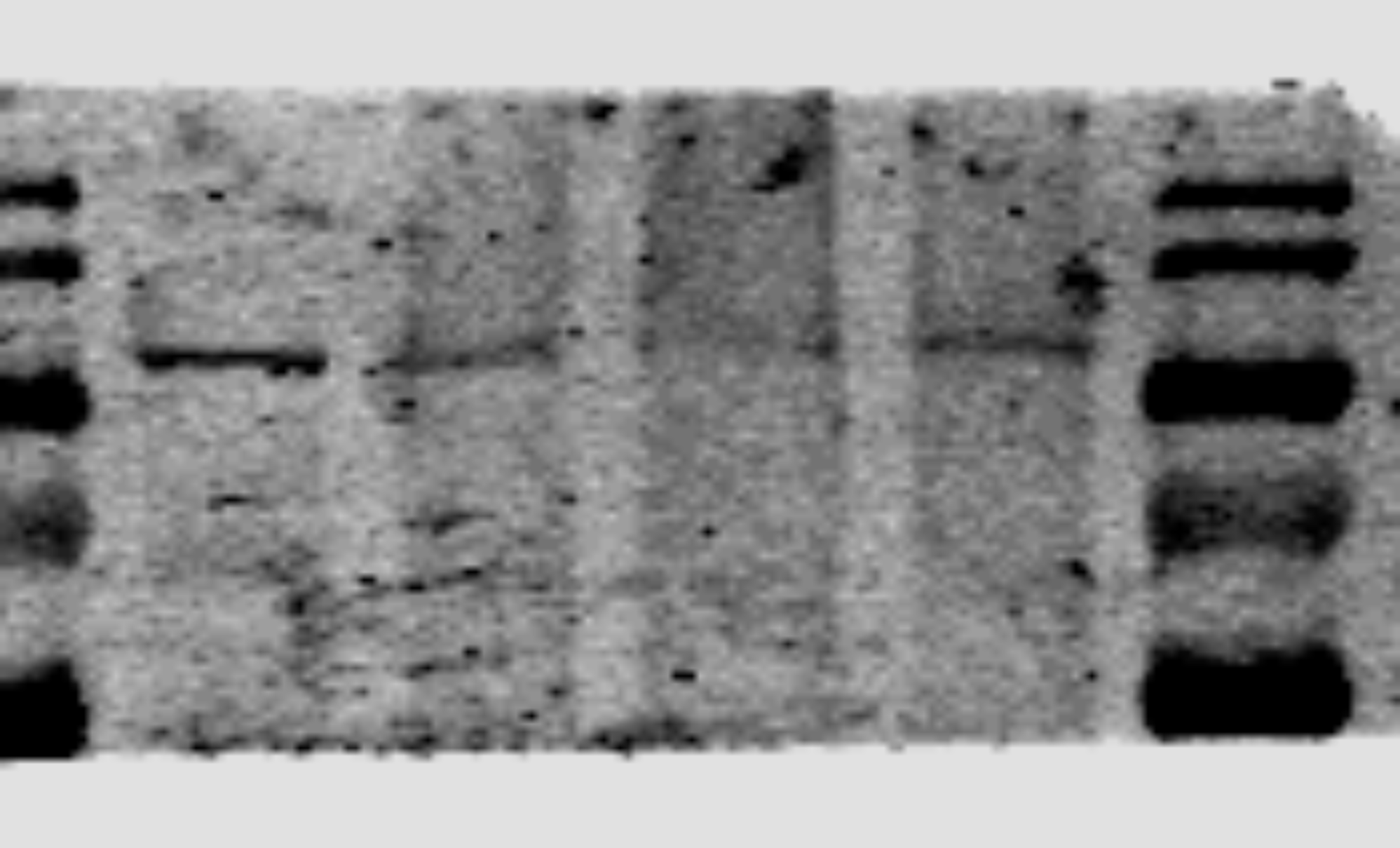

Supplement: Supplementary file 2 — Supplementary Information 2. [file 41598_2023_50476_MOESM2_ESM.zip › protein/3 repeat/1.magration/5637/MMP9.tif]

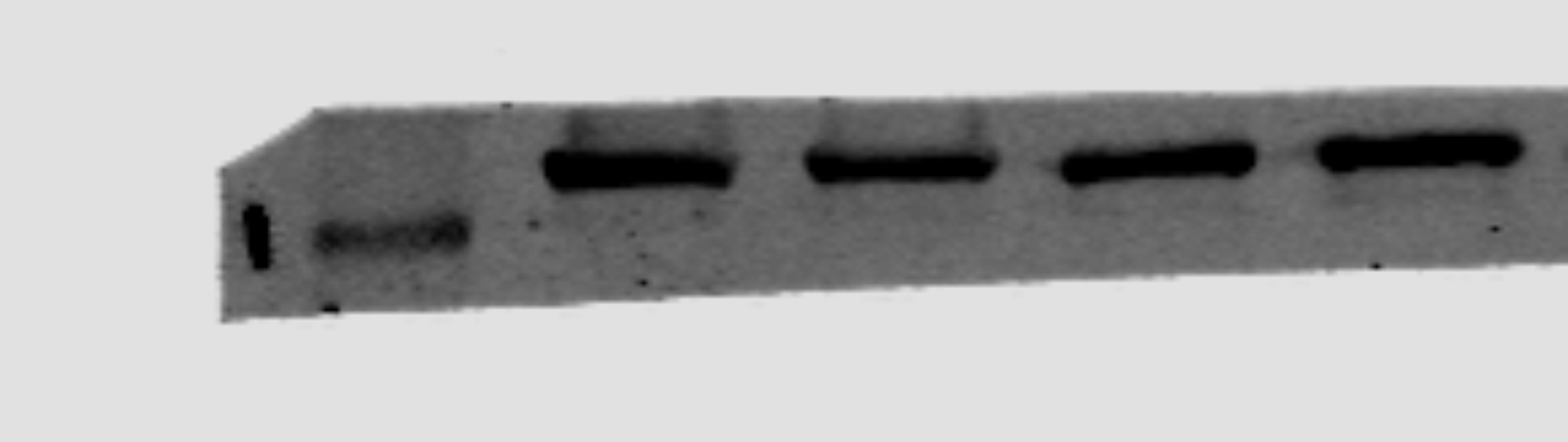

Supplement: Supplementary file 2 — Supplementary Information 2. [file 41598_2023_50476_MOESM2_ESM.zip › protein/3 repeat/1.magration/T24/23.tif]

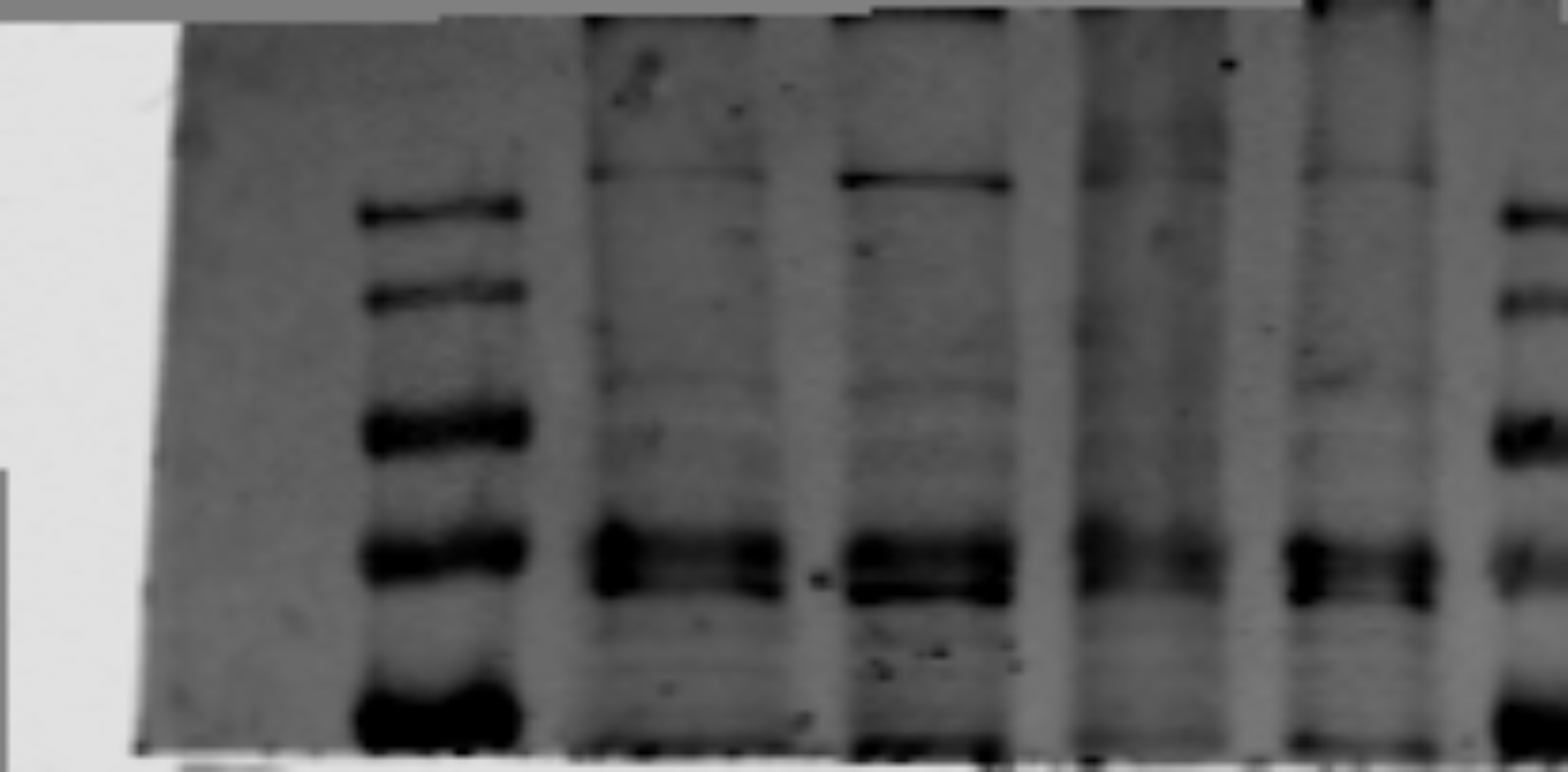

Supplement: Supplementary file 2 — Supplementary Information 2. [file 41598_2023_50476_MOESM2_ESM.zip › protein/3 repeat/1.magration/T24/MMP2.tif]

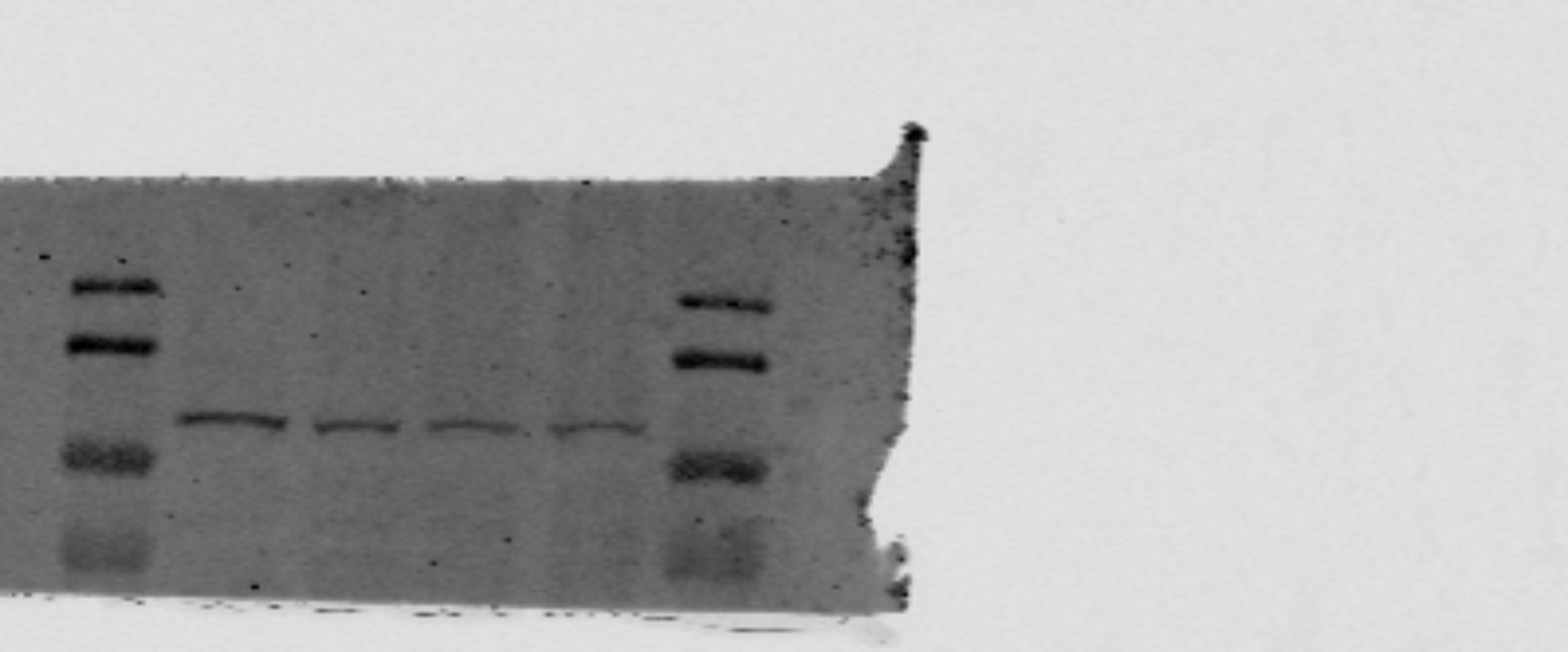

Supplement: Supplementary file 2 — Supplementary Information 2. [file 41598_2023_50476_MOESM2_ESM.zip › protein/3 repeat/1.magration/T24/MMP9.tif]

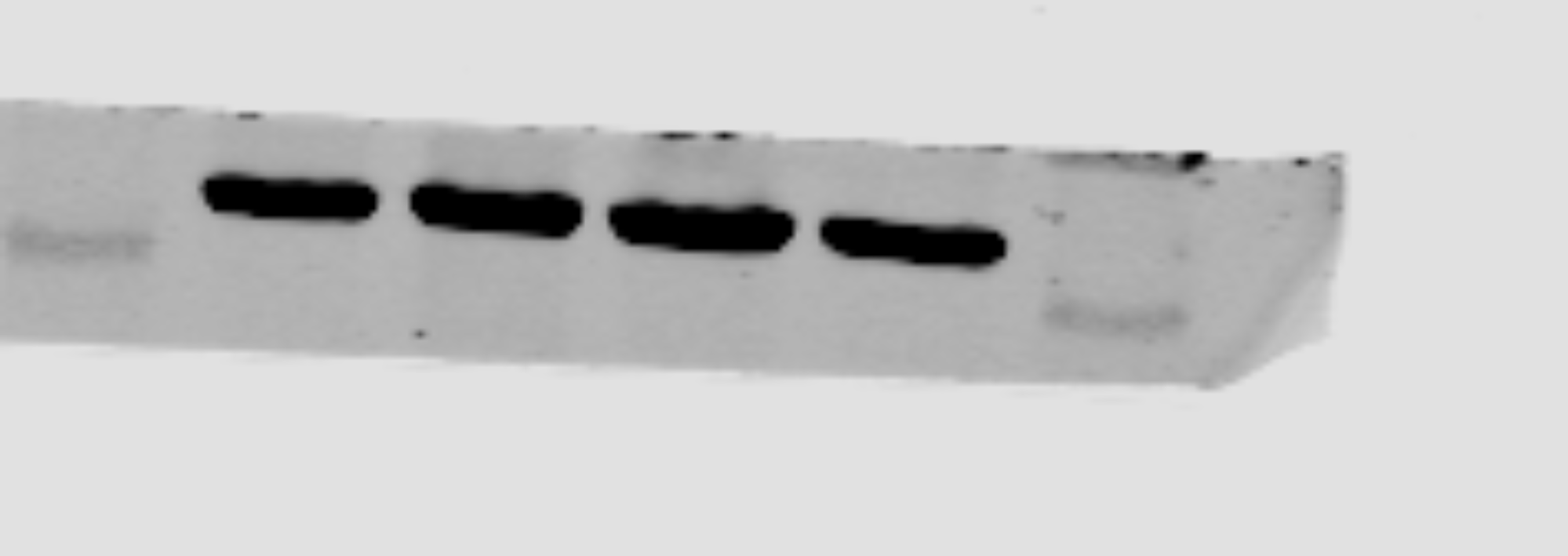

Supplement: Supplementary file 2 — Supplementary Information 2. [file 41598_2023_50476_MOESM2_ESM.zip › protein/3 repeat/2.apoptosis/5637/ACTIN.tif]

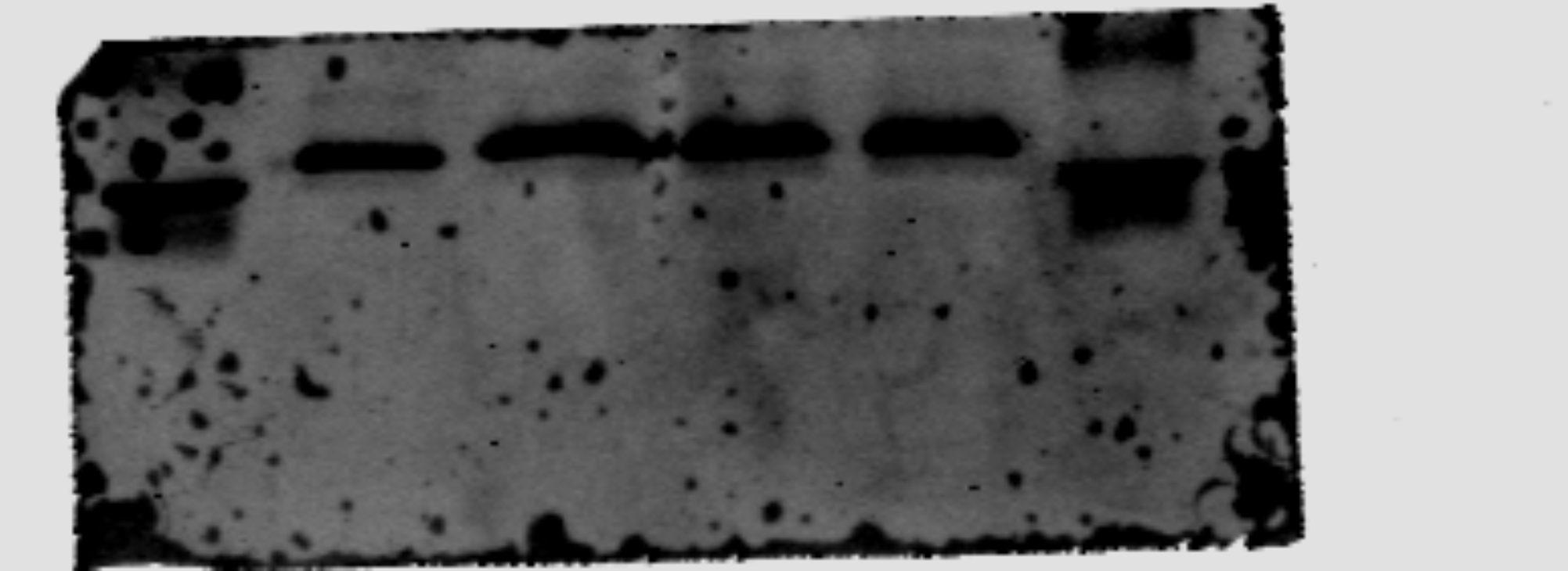

Supplement: Supplementary file 2 — Supplementary Information 2. [file 41598_2023_50476_MOESM2_ESM.zip › protein/3 repeat/2.apoptosis/5637/BAX.tif]

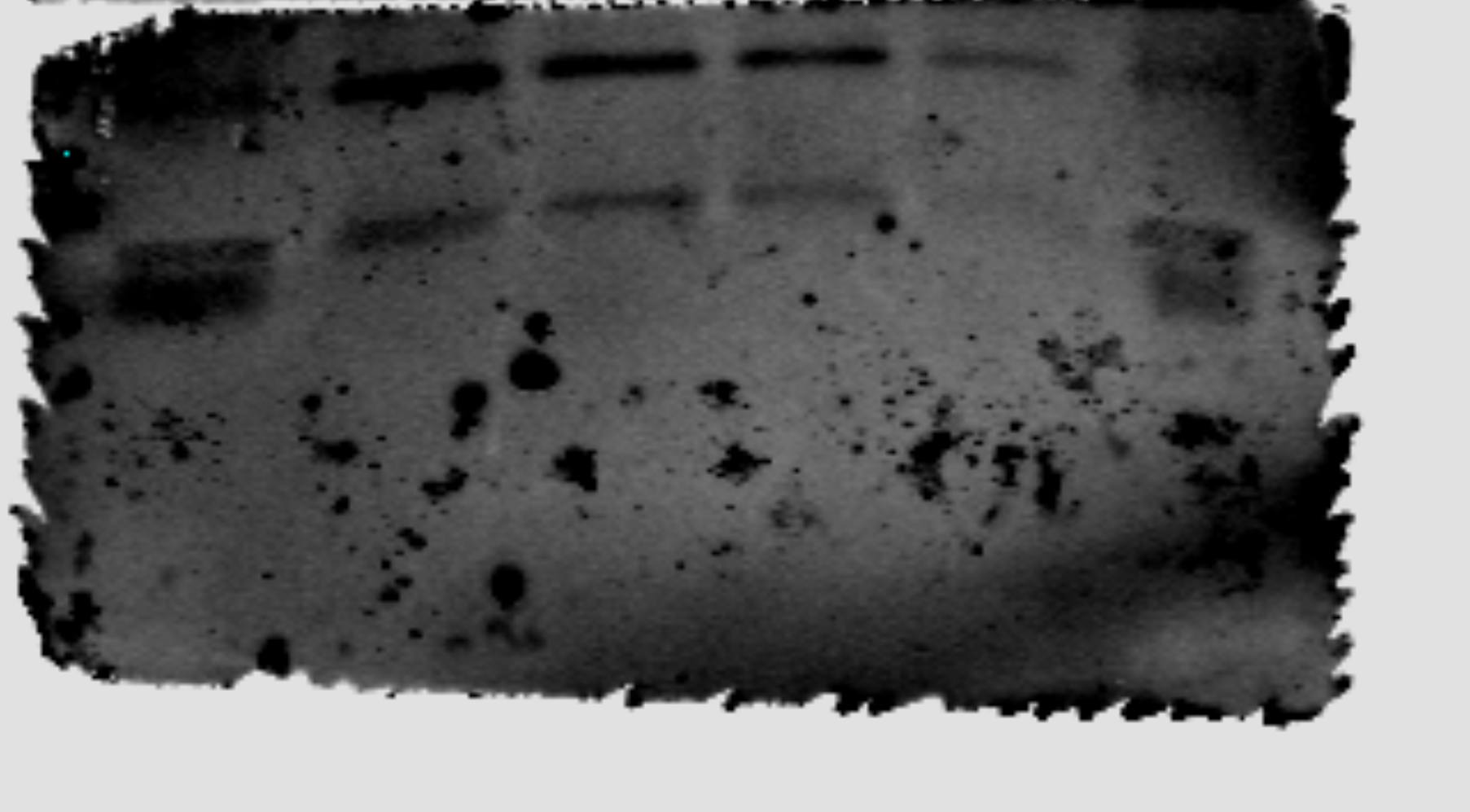

Supplement: Supplementary file 2 — Supplementary Information 2. [file 41598_2023_50476_MOESM2_ESM.zip › protein/3 repeat/2.apoptosis/5637/BCL2.tif]

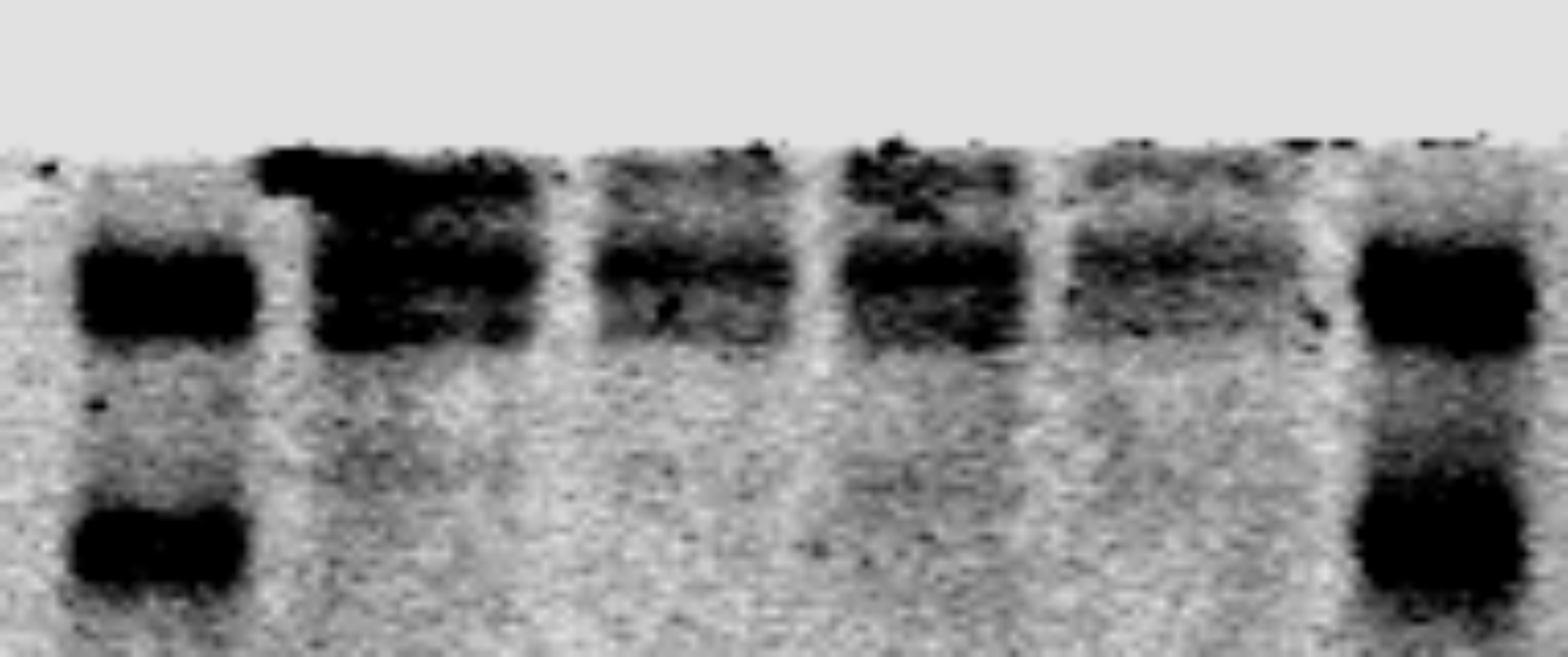

Supplement: Supplementary file 2 — Supplementary Information 2. [file 41598_2023_50476_MOESM2_ESM.zip › protein/3 repeat/2.apoptosis/5637/CASPASE3.tif]

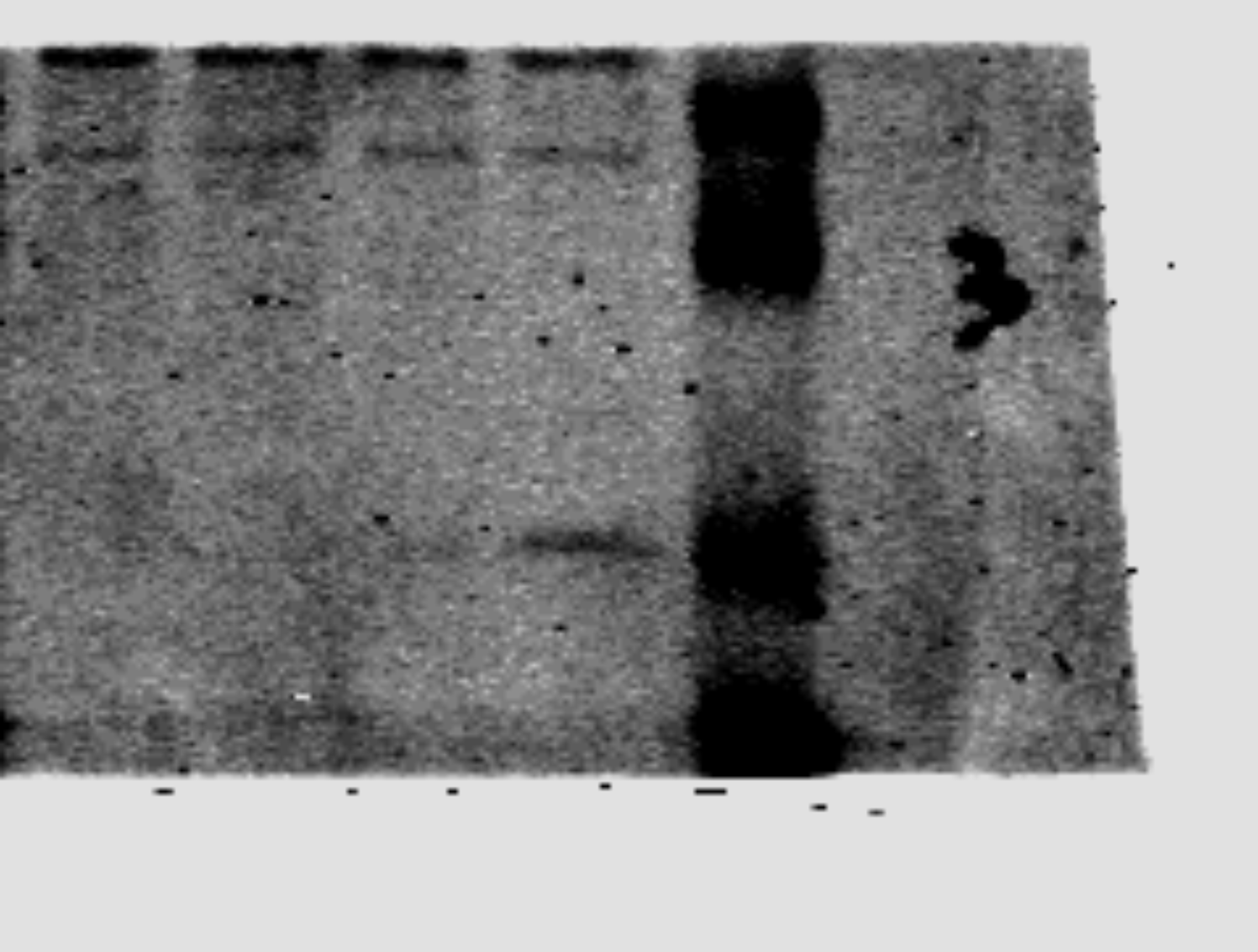

Supplement: Supplementary file 2 — Supplementary Information 2. [file 41598_2023_50476_MOESM2_ESM.zip › protein/3 repeat/2.apoptosis/5637/cleaved caspase3.tif]

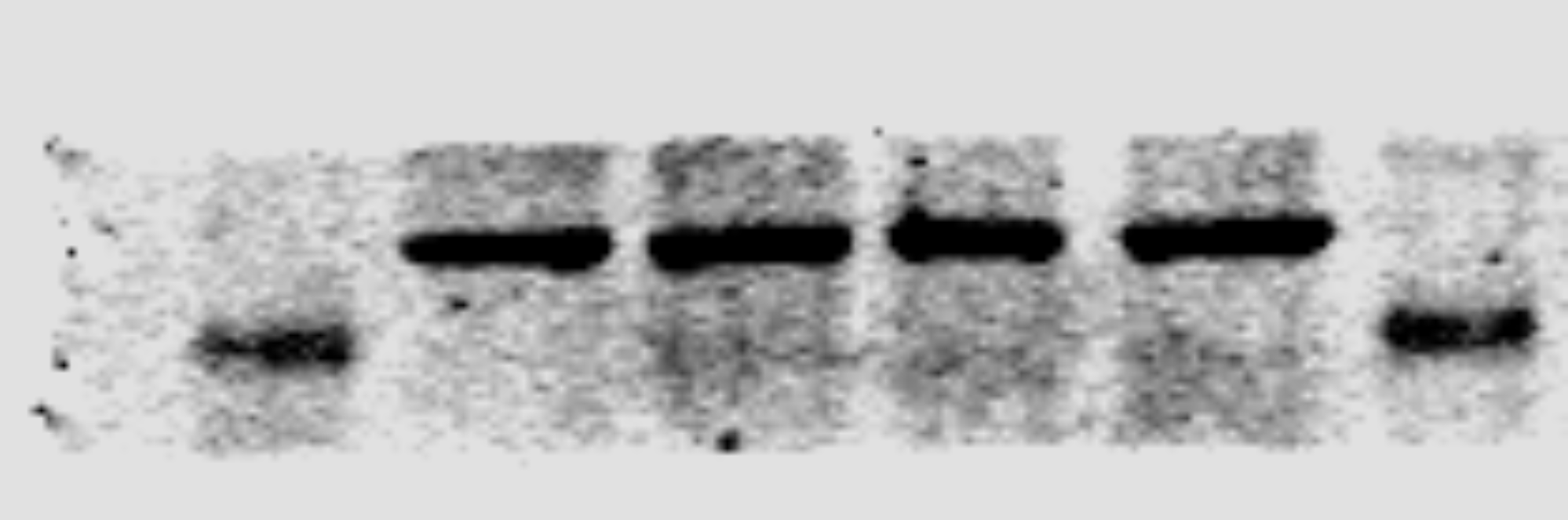

Supplement: Supplementary file 2 — Supplementary Information 2. [file 41598_2023_50476_MOESM2_ESM.zip › protein/3 repeat/2.apoptosis/T24/ACTIN.tif]

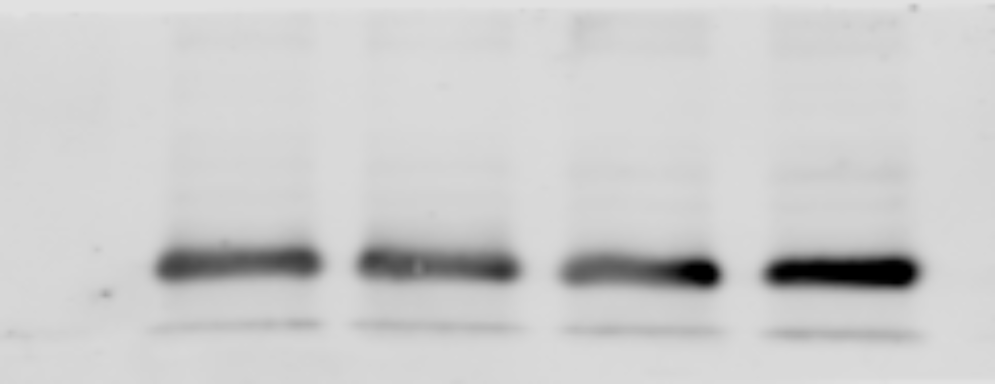

Supplement: Supplementary file 2 — Supplementary Information 2. [file 41598_2023_50476_MOESM2_ESM.zip › protein/3 repeat/2.apoptosis/T24/BAX.png]

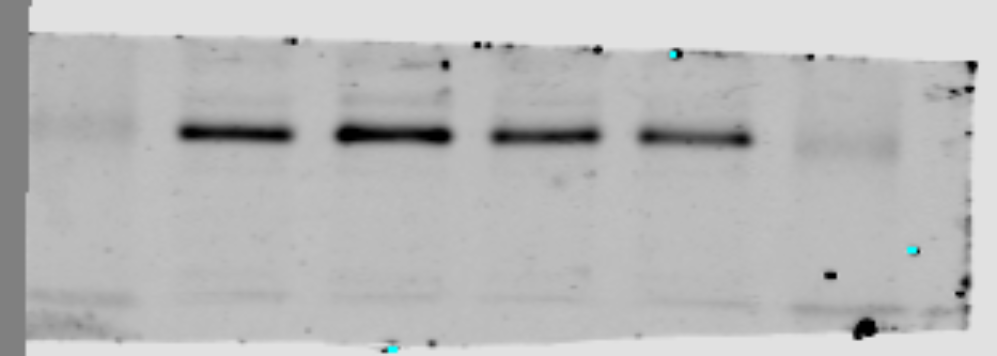

Supplement: Supplementary file 2 — Supplementary Information 2. [file 41598_2023_50476_MOESM2_ESM.zip › protein/3 repeat/2.apoptosis/T24/BCL-2.png]

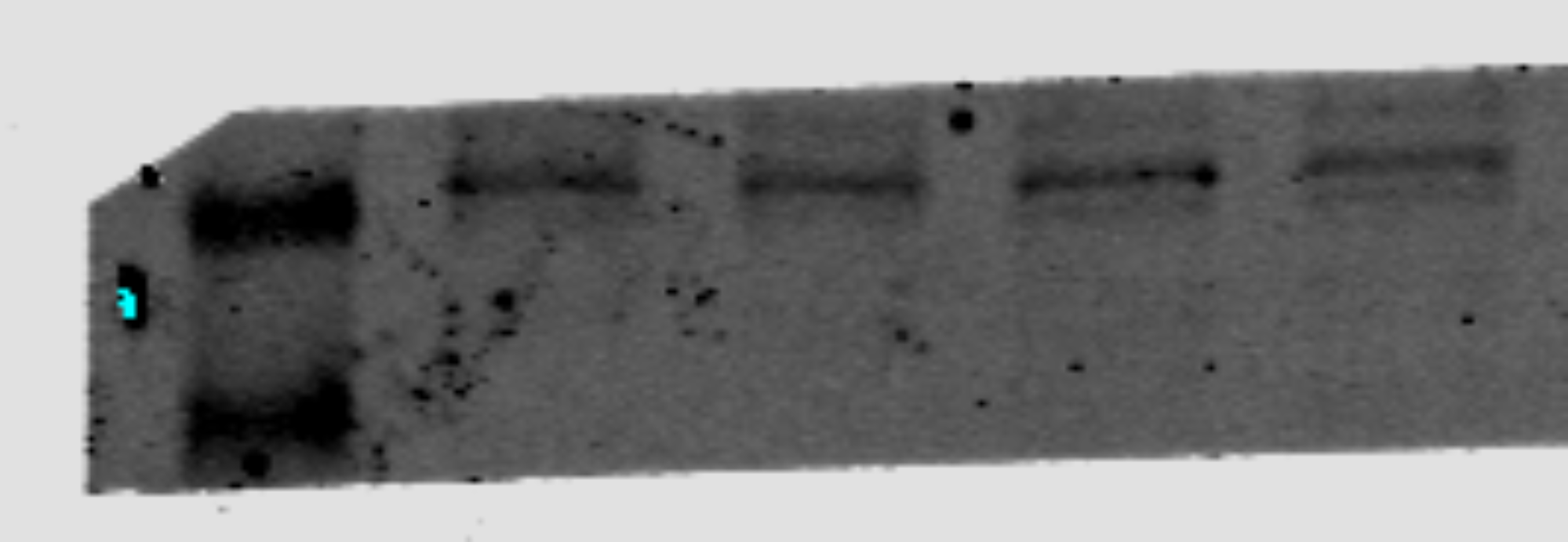

Supplement: Supplementary file 2 — Supplementary Information 2. [file 41598_2023_50476_MOESM2_ESM.zip › protein/3 repeat/2.apoptosis/T24/caspase3.tif]

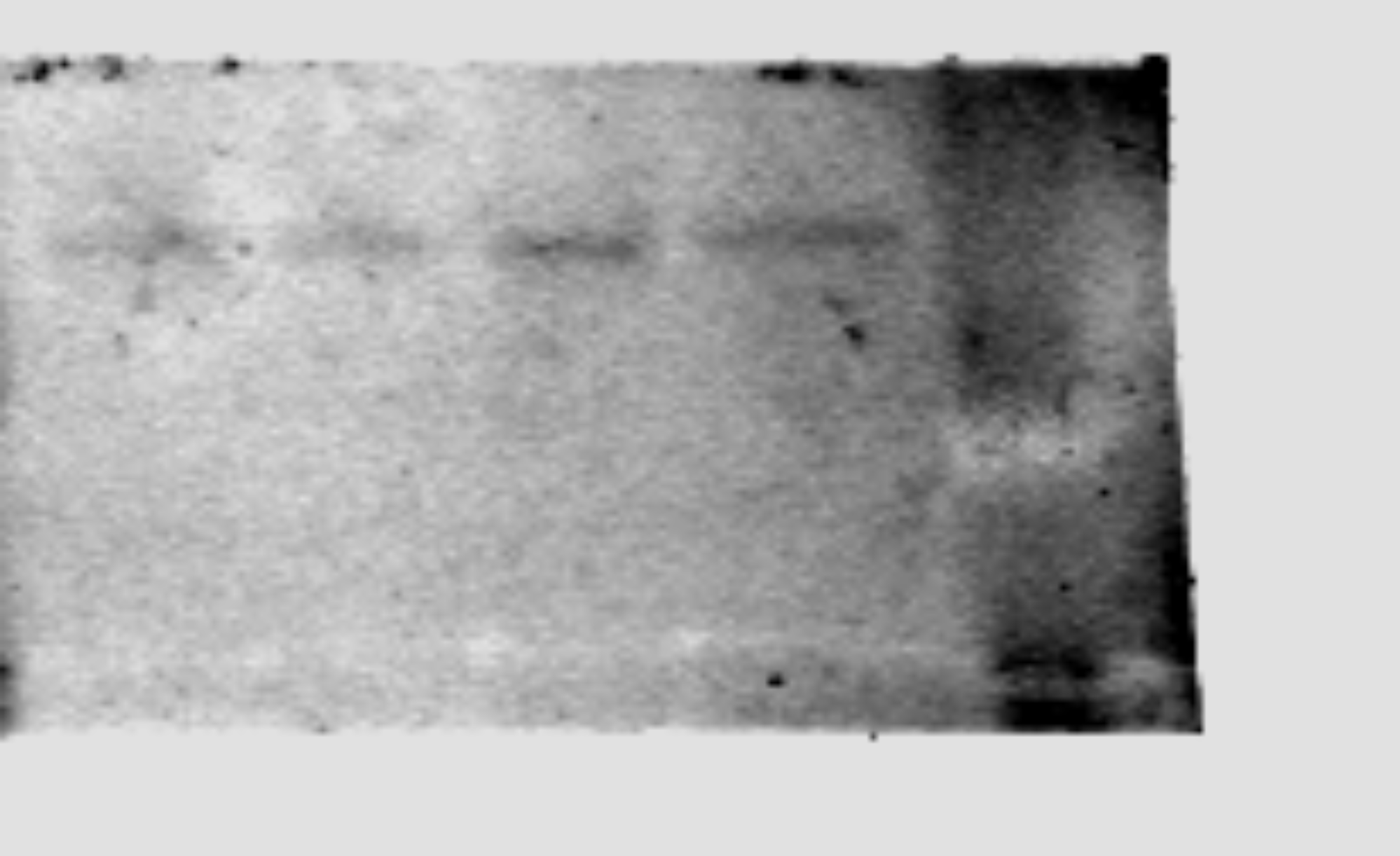

Supplement: Supplementary file 2 — Supplementary Information 2. [file 41598_2023_50476_MOESM2_ESM.zip › protein/3 repeat/2.apoptosis/T24/cleaved caspase3.tif]

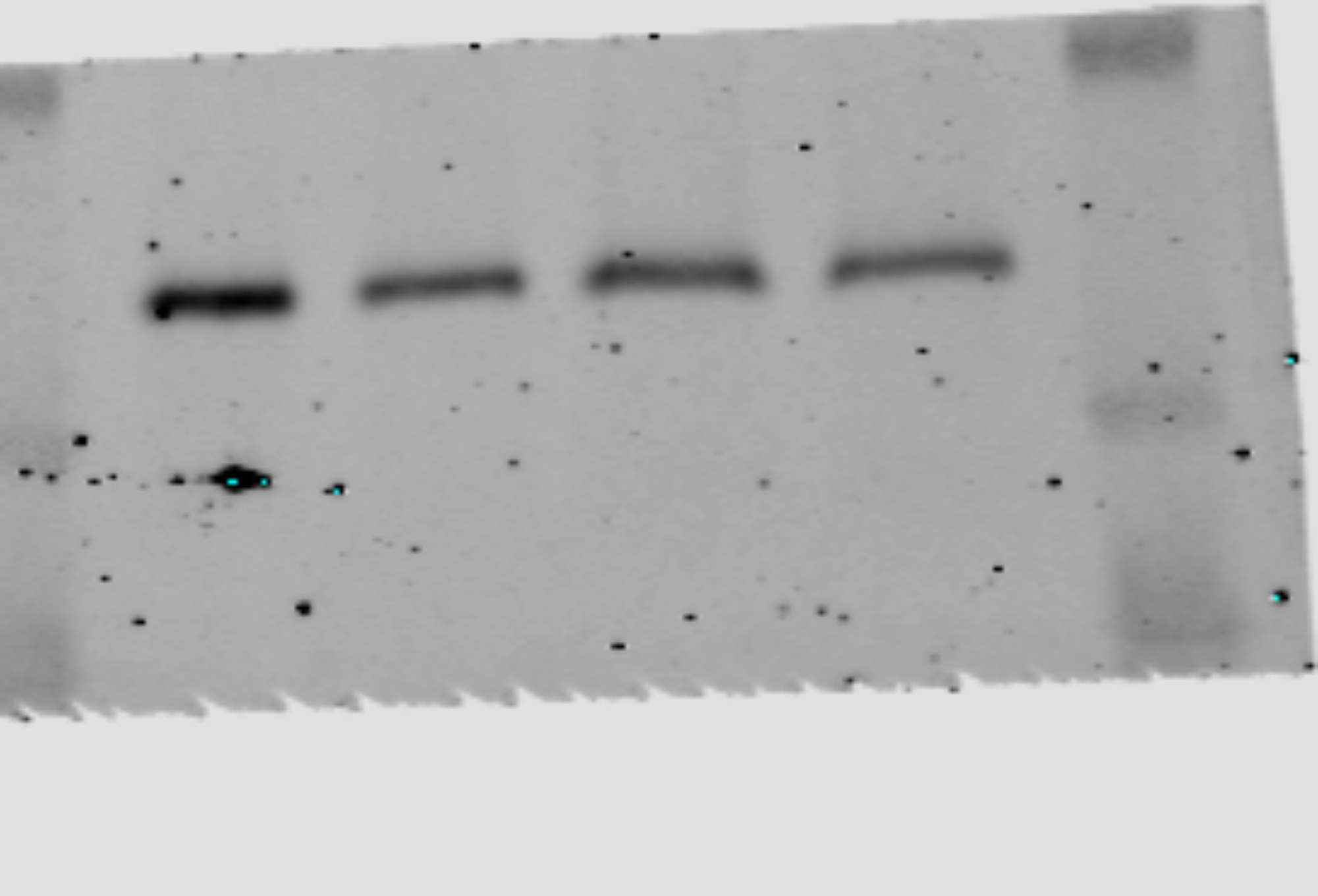

Supplement: Supplementary file 2 — Supplementary Information 2. [file 41598_2023_50476_MOESM2_ESM.zip › protein/3 repeat/3.ferroptosis/5637/11.13.tif]

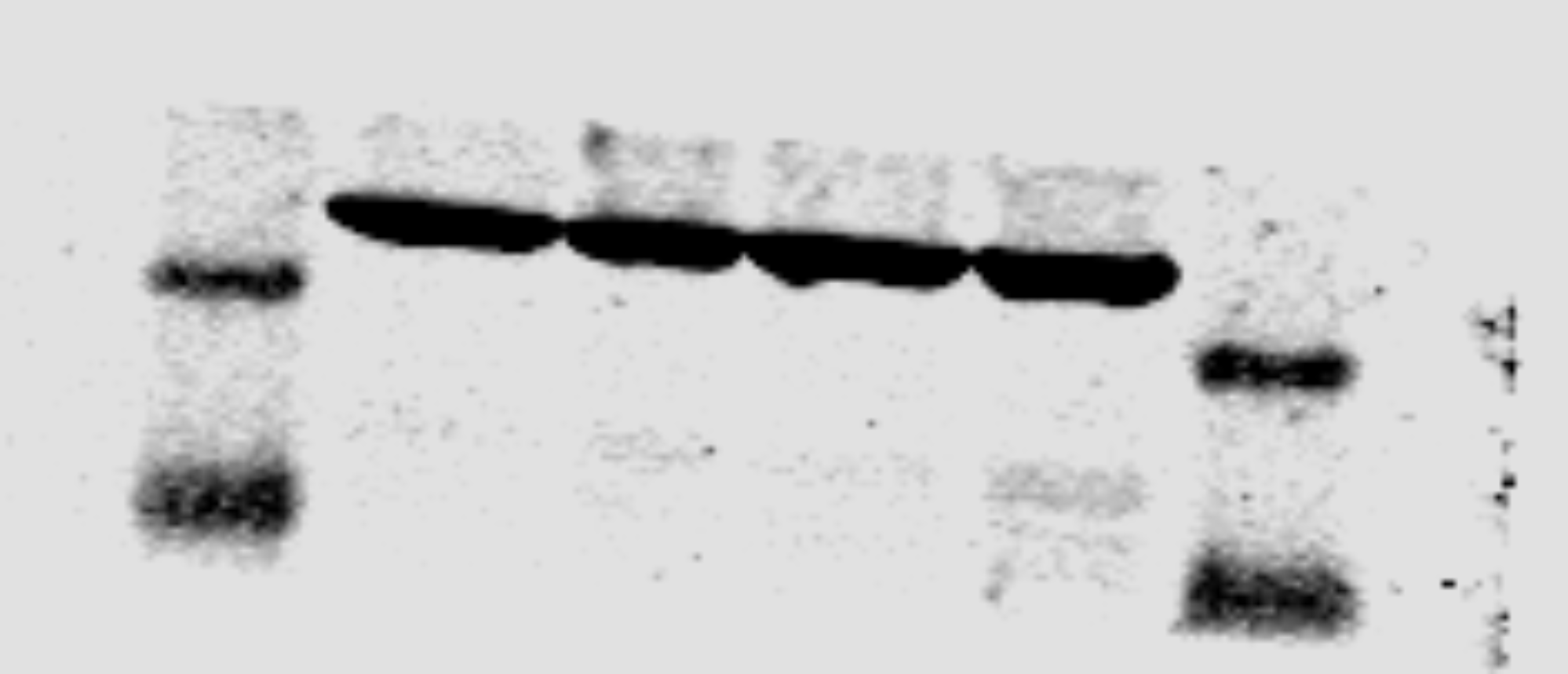

Supplement: Supplementary file 2 — Supplementary Information 2. [file 41598_2023_50476_MOESM2_ESM.zip › protein/3 repeat/3.ferroptosis/5637/ACTIN.tif]

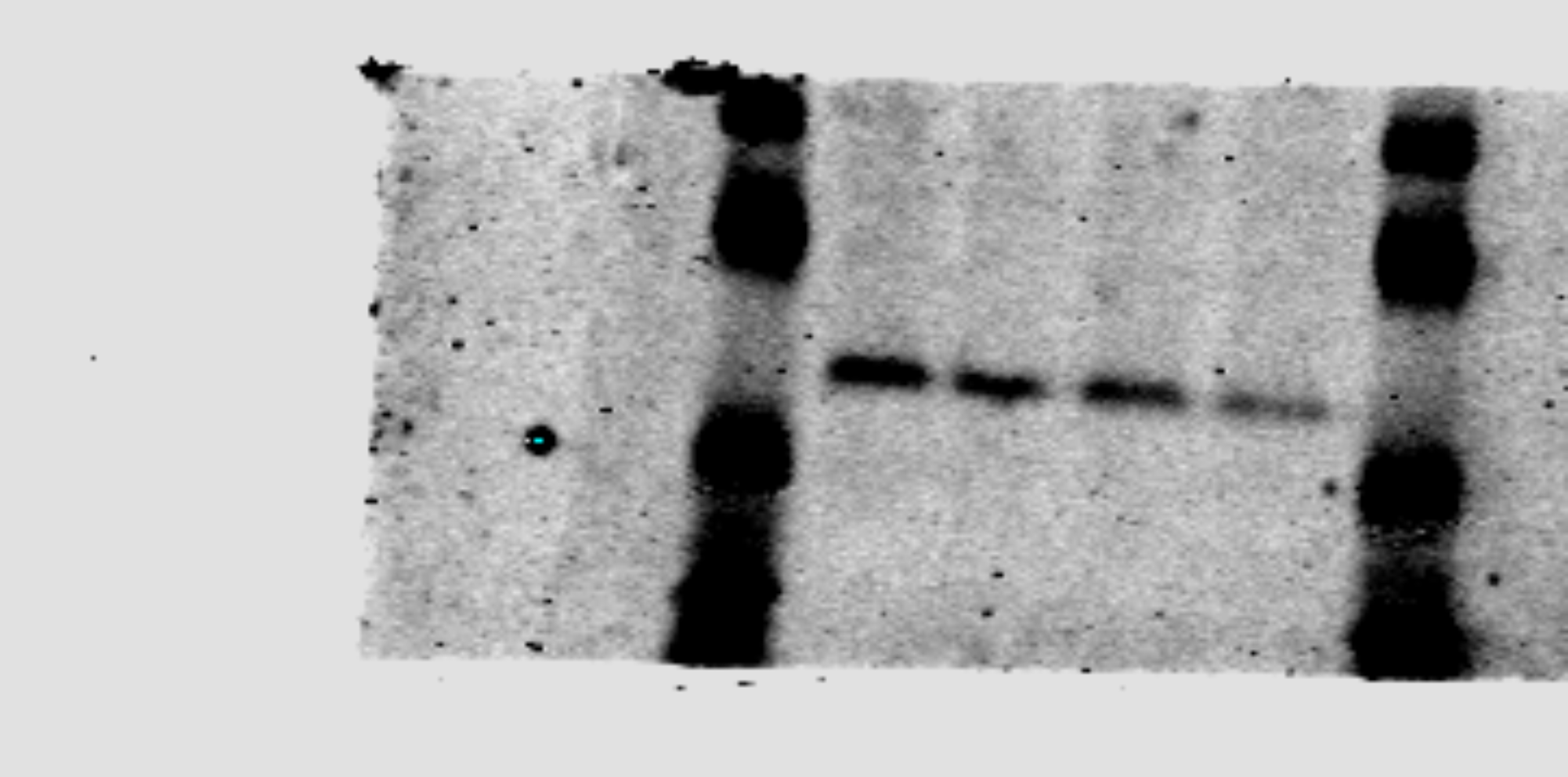

Supplement: Supplementary file 2 — Supplementary Information 2. [file 41598_2023_50476_MOESM2_ESM.zip › protein/3 repeat/3.ferroptosis/T24/2.242.tif]

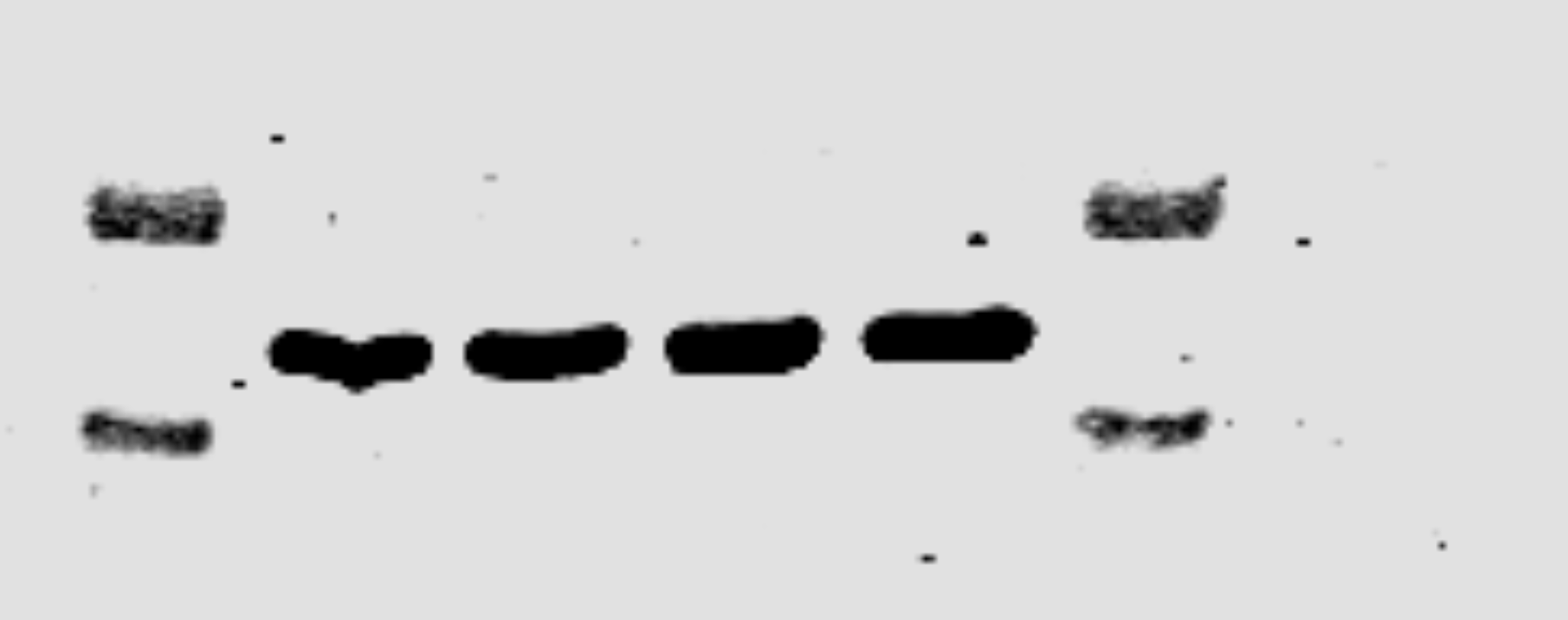

Supplement: Supplementary file 2 — Supplementary Information 2. [file 41598_2023_50476_MOESM2_ESM.zip › protein/3 repeat/3.ferroptosis/T24/20.tif]

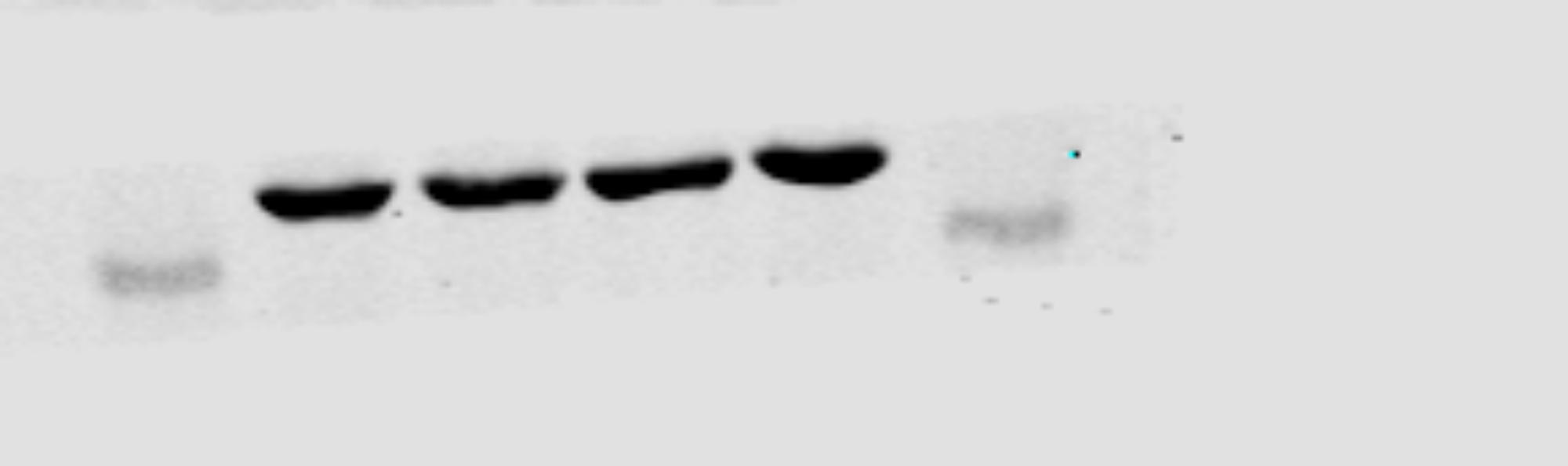

Supplement: Supplementary file 2 — Supplementary Information 2. [file 41598_2023_50476_MOESM2_ESM.zip › protein/3 repeat/4.targets/5637/ACTIN.tif]

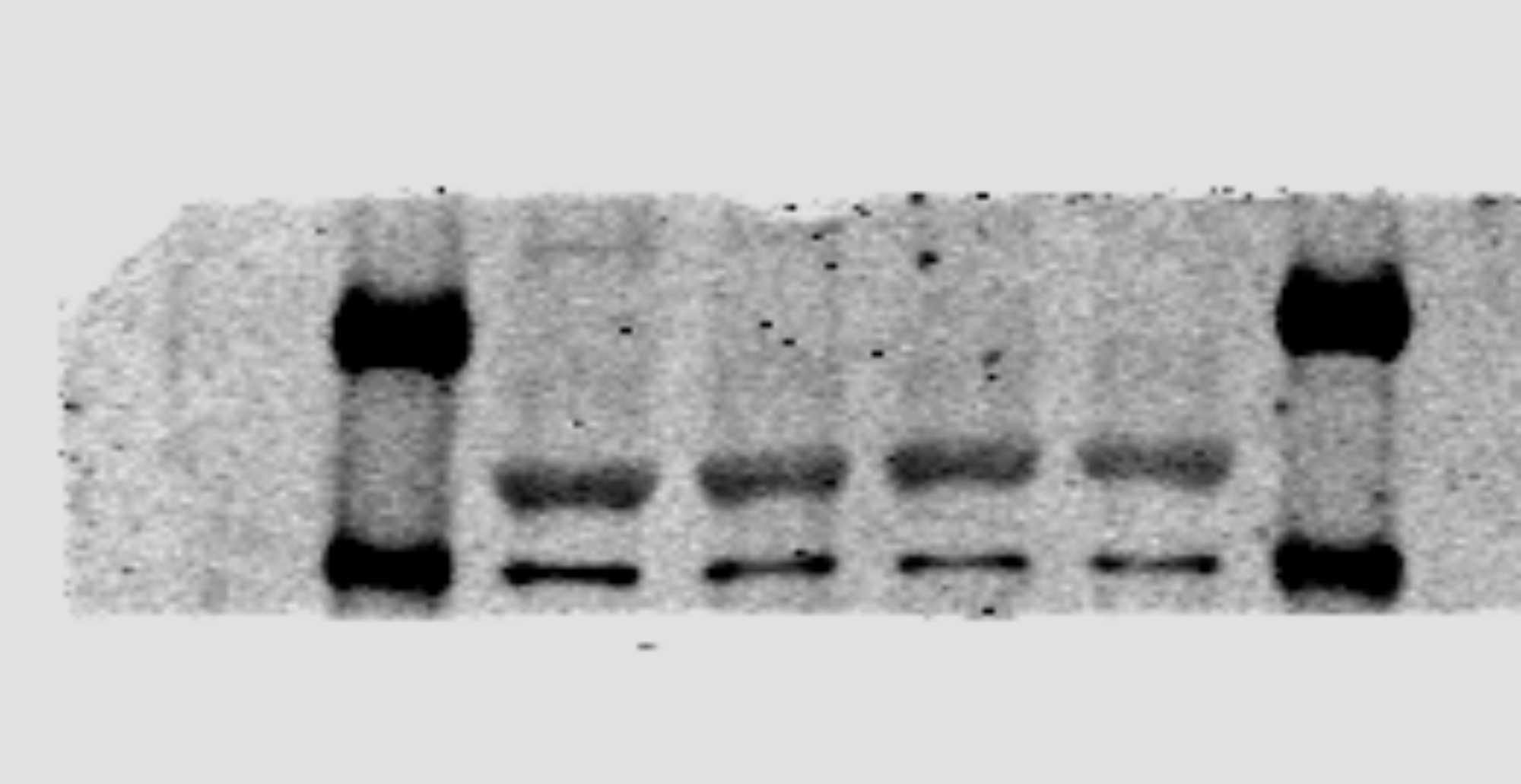

Supplement: Supplementary file 2 — Supplementary Information 2. [file 41598_2023_50476_MOESM2_ESM.zip › protein/3 repeat/4.targets/5637/MAPK1.tif]

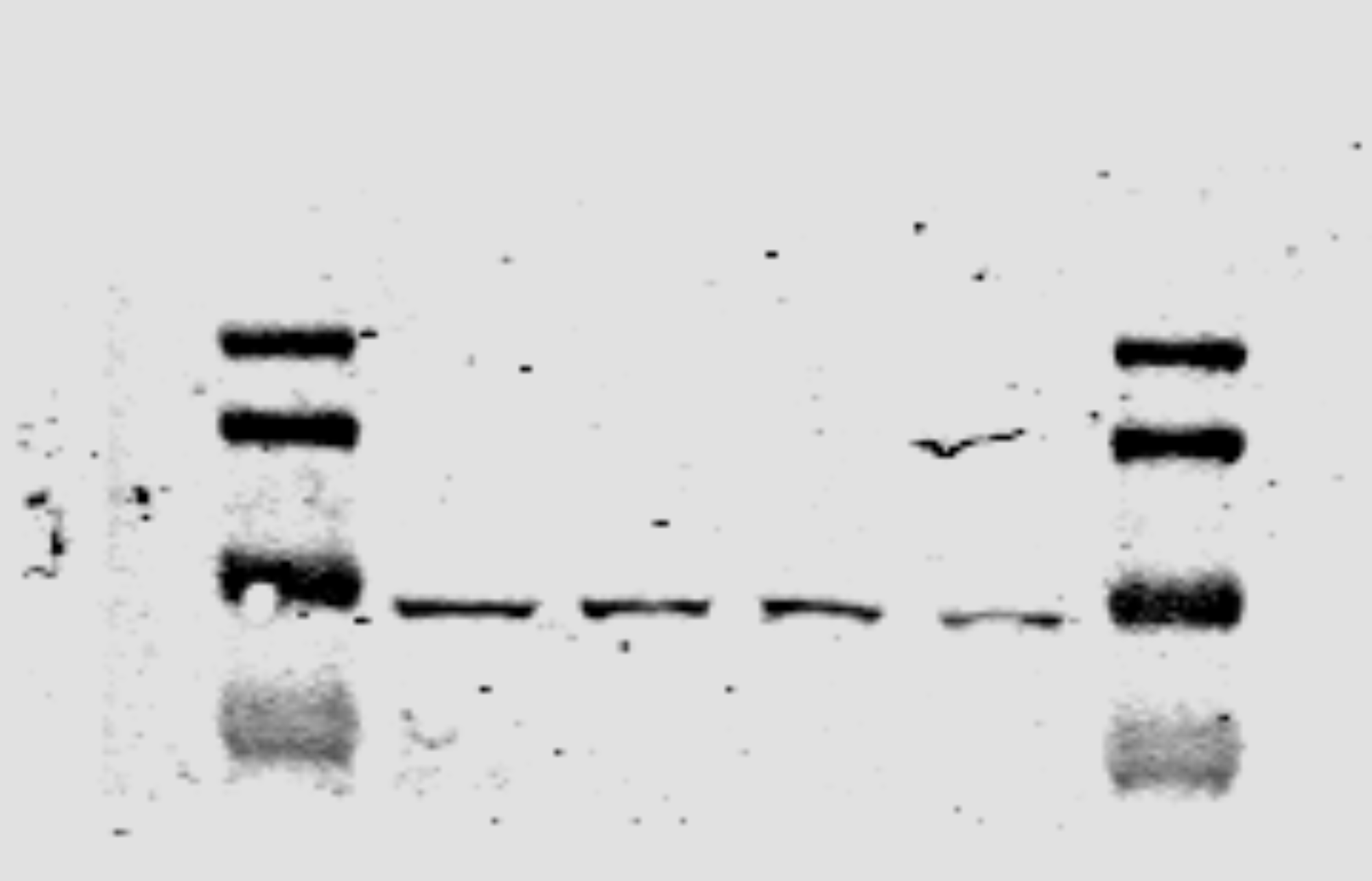

Supplement: Supplementary file 2 — Supplementary Information 2. [file 41598_2023_50476_MOESM2_ESM.zip › protein/3 repeat/4.targets/5637/P85.tif]

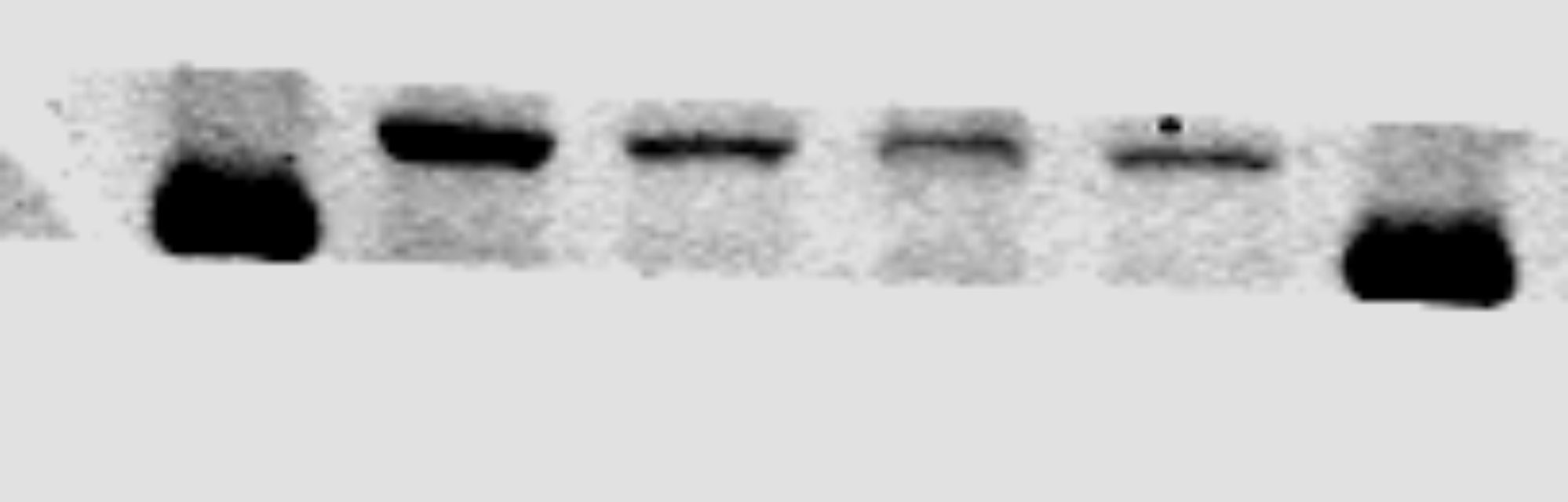

Supplement: Supplementary file 2 — Supplementary Information 2. [file 41598_2023_50476_MOESM2_ESM.zip › protein/3 repeat/4.targets/5637/SRC.tif]

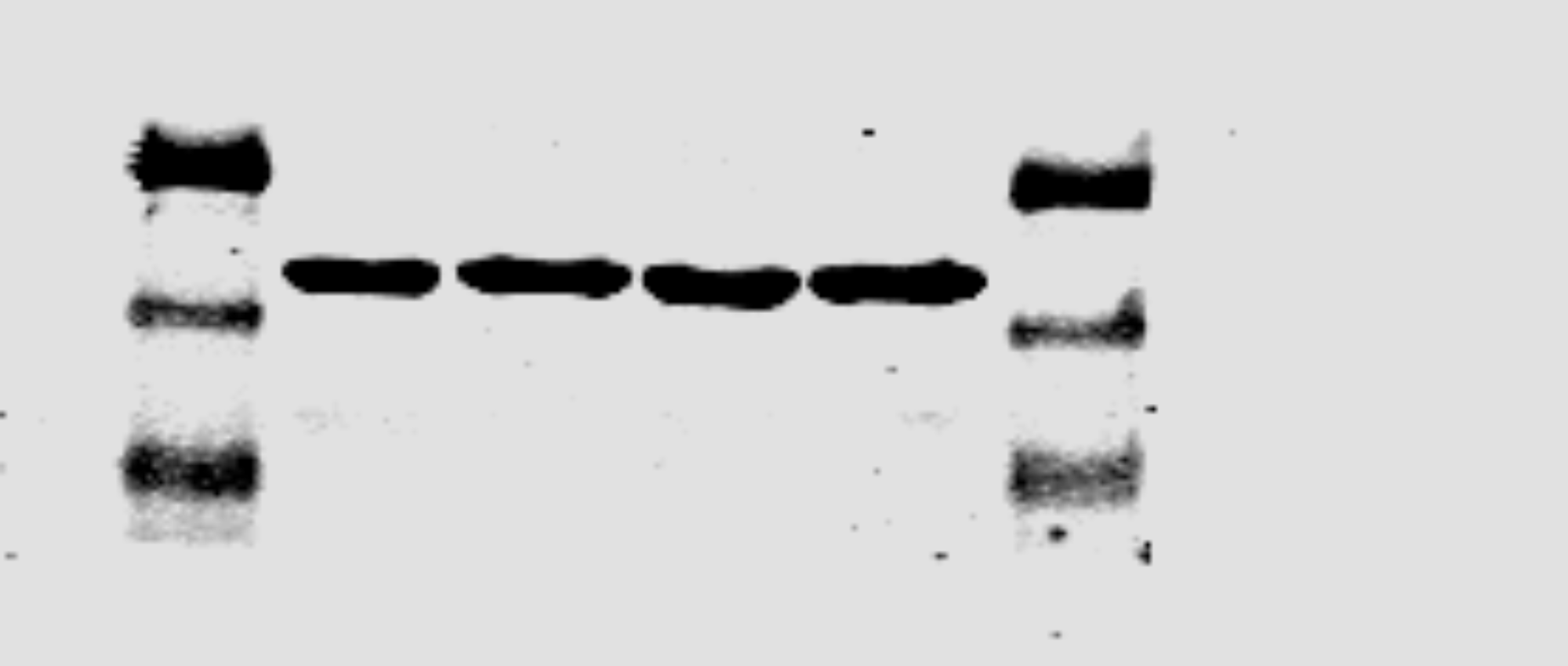

Supplement: Supplementary file 2 — Supplementary Information 2. [file 41598_2023_50476_MOESM2_ESM.zip › protein/3 repeat/4.targets/T24/ACTIN.tif]

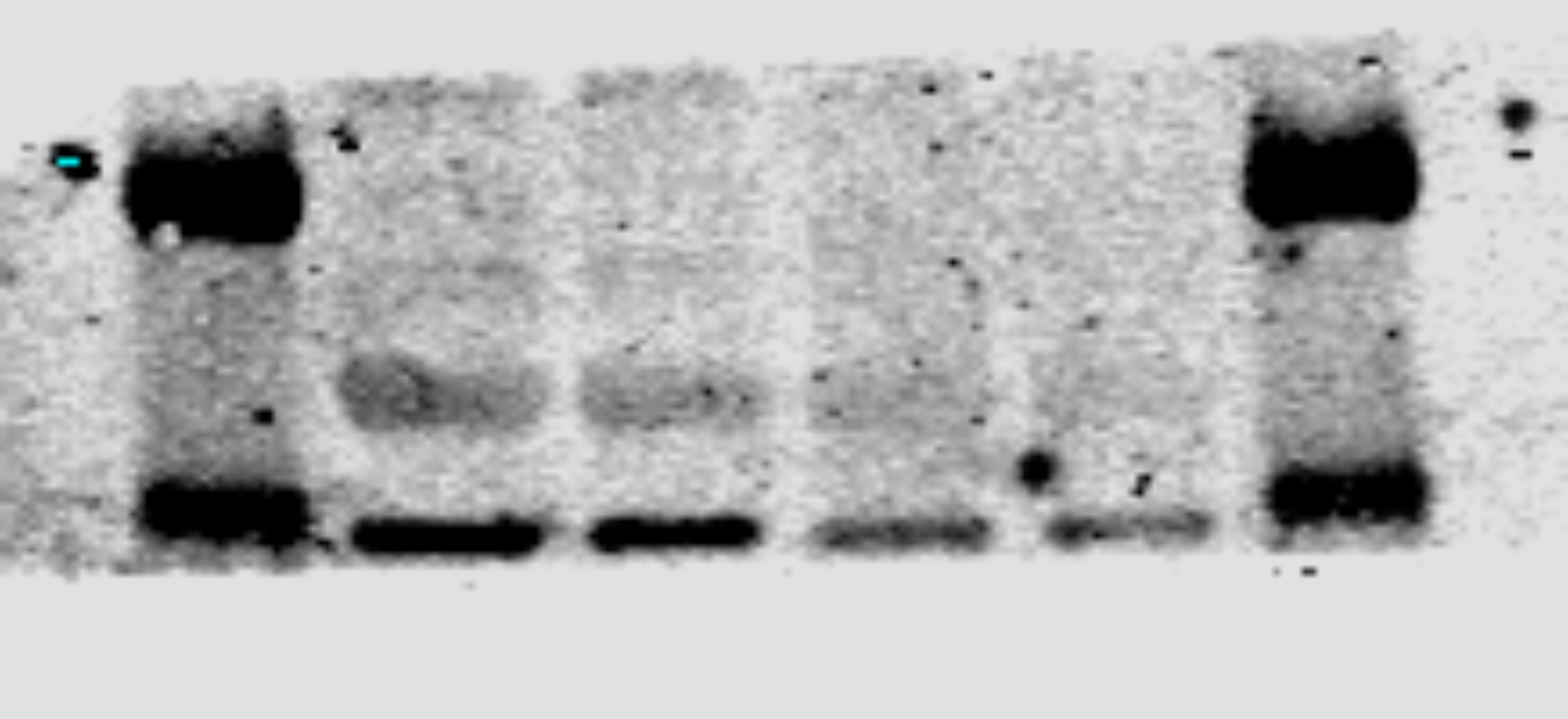

Supplement: Supplementary file 2 — Supplementary Information 2. [file 41598_2023_50476_MOESM2_ESM.zip › protein/3 repeat/4.targets/T24/MAPK1.tif]

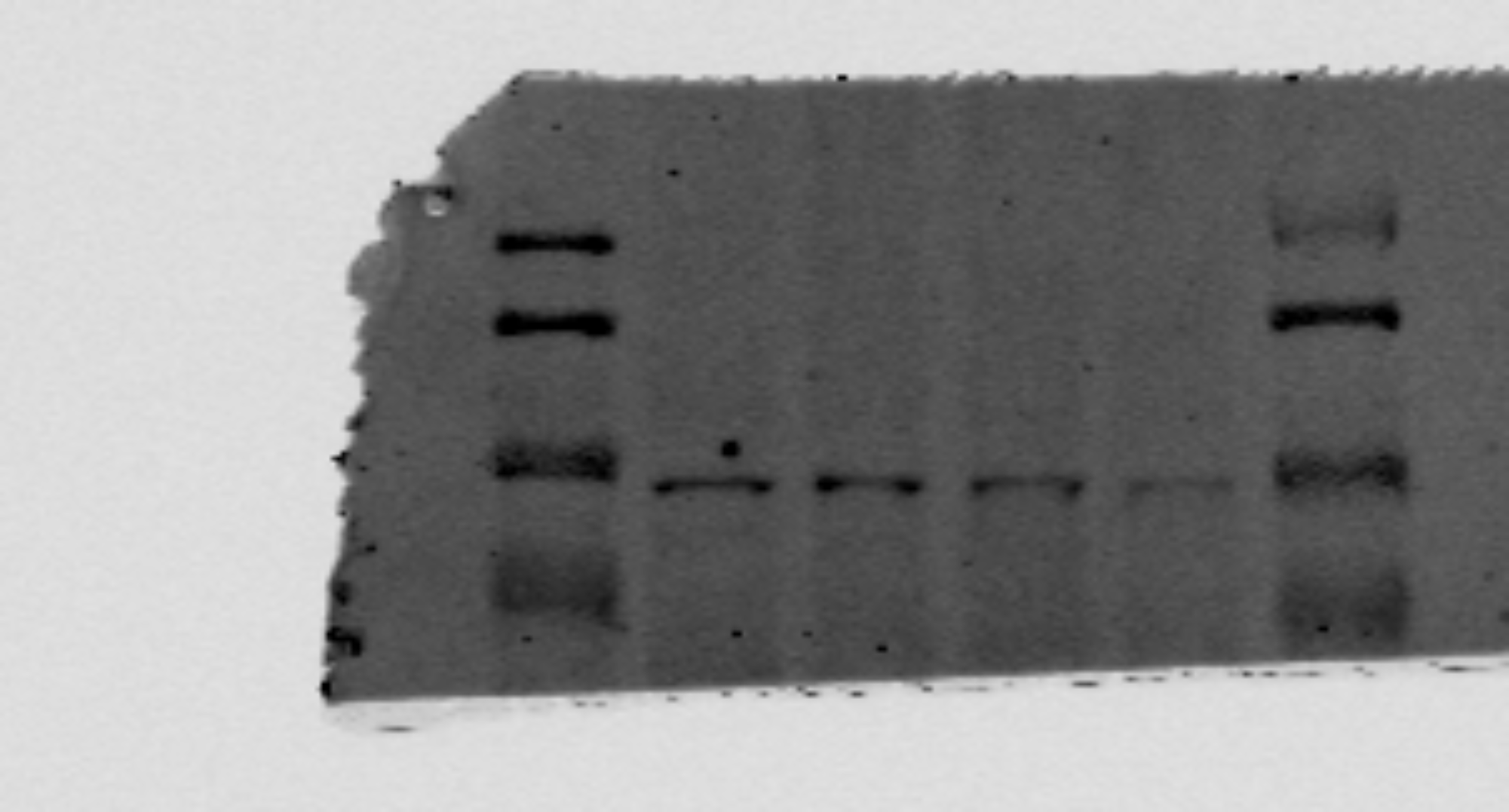

Supplement: Supplementary file 2 — Supplementary Information 2. [file 41598_2023_50476_MOESM2_ESM.zip › protein/3 repeat/4.targets/T24/P85.tif]

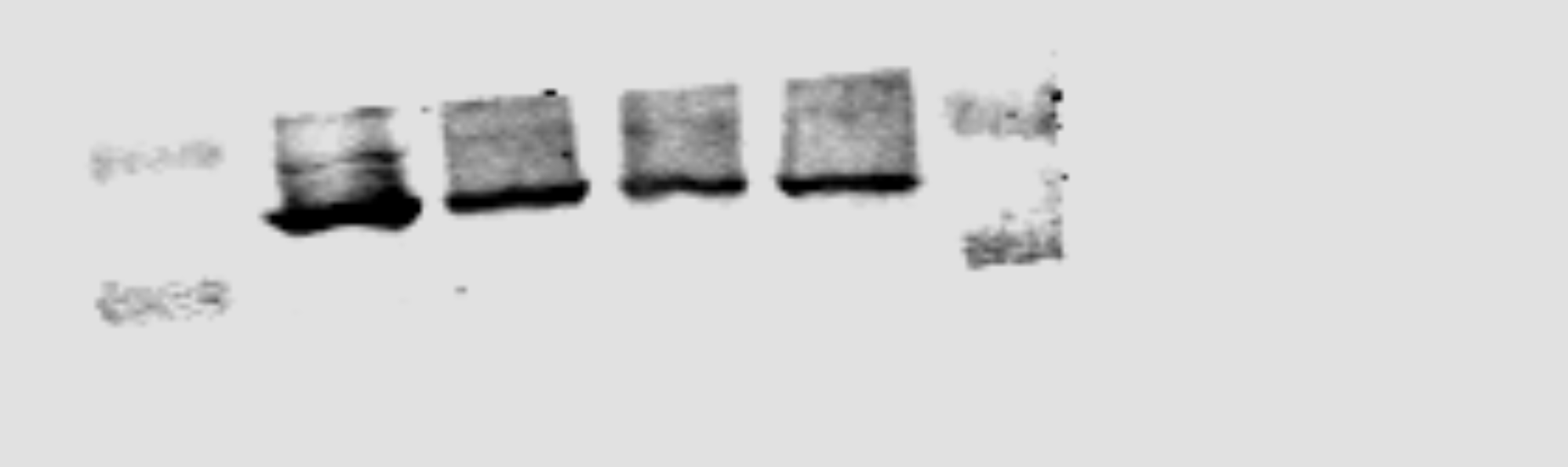

Supplement: Supplementary file 2 — Supplementary Information 2. [file 41598_2023_50476_MOESM2_ESM.zip › protein/3 repeat/4.targets/T24/SRC.tif]

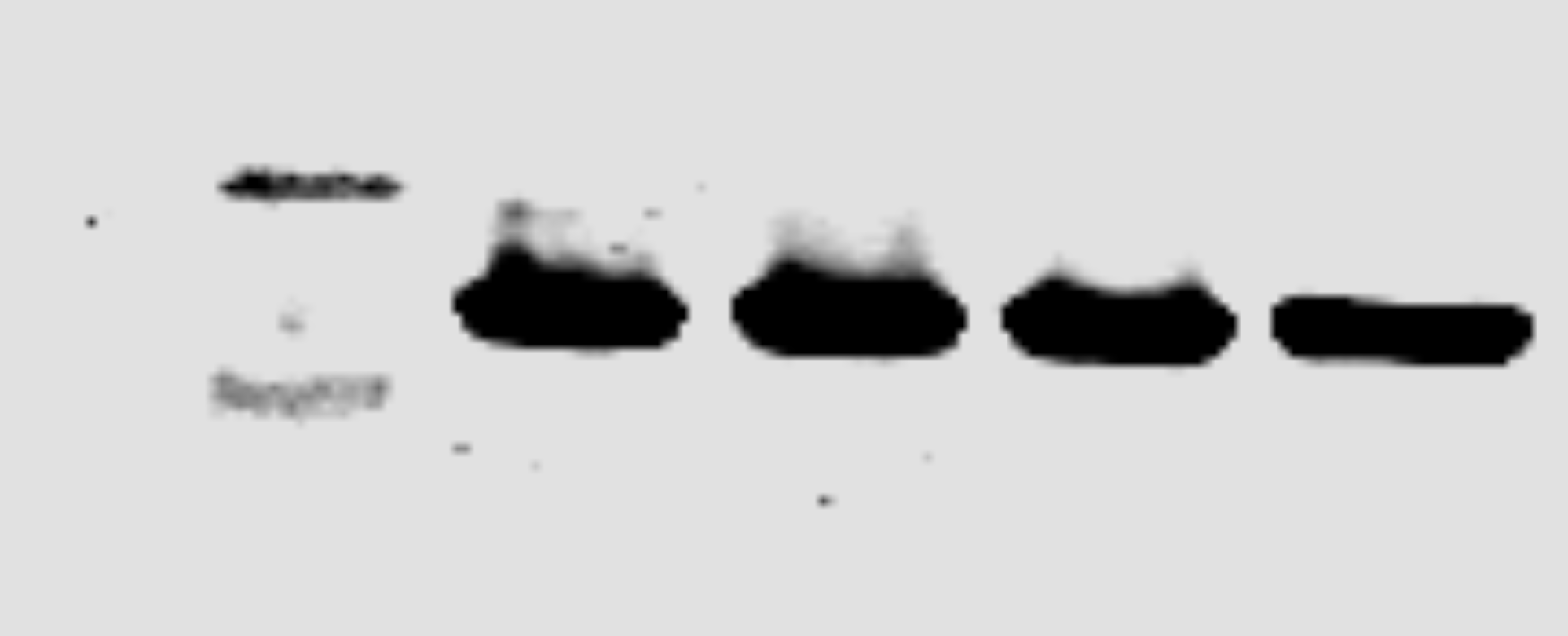

Supplement: Supplementary file 2 — Supplementary Information 2. [file 41598_2023_50476_MOESM2_ESM.zip › protein/3 repeat/5.pathway/5637/ACTIN.tif]

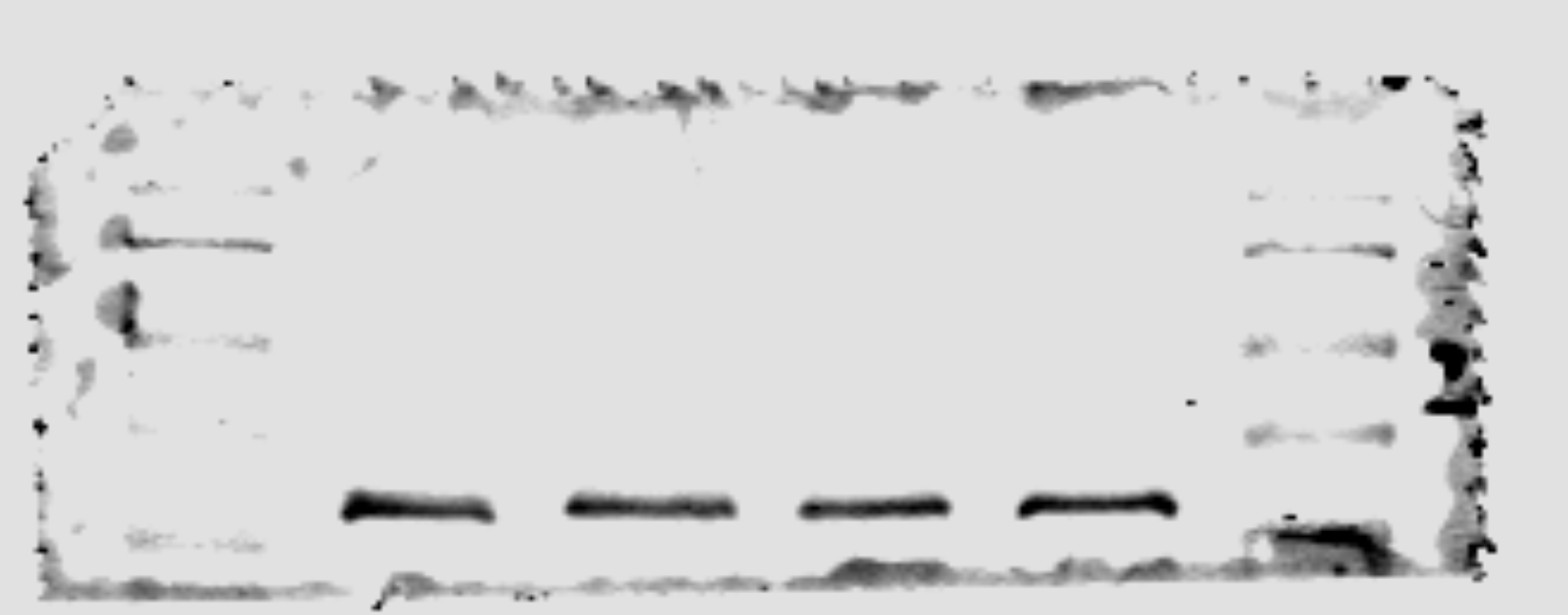

Supplement: Supplementary file 2 — Supplementary Information 2. [file 41598_2023_50476_MOESM2_ESM.zip › protein/3 repeat/5.pathway/5637/AKT.png]

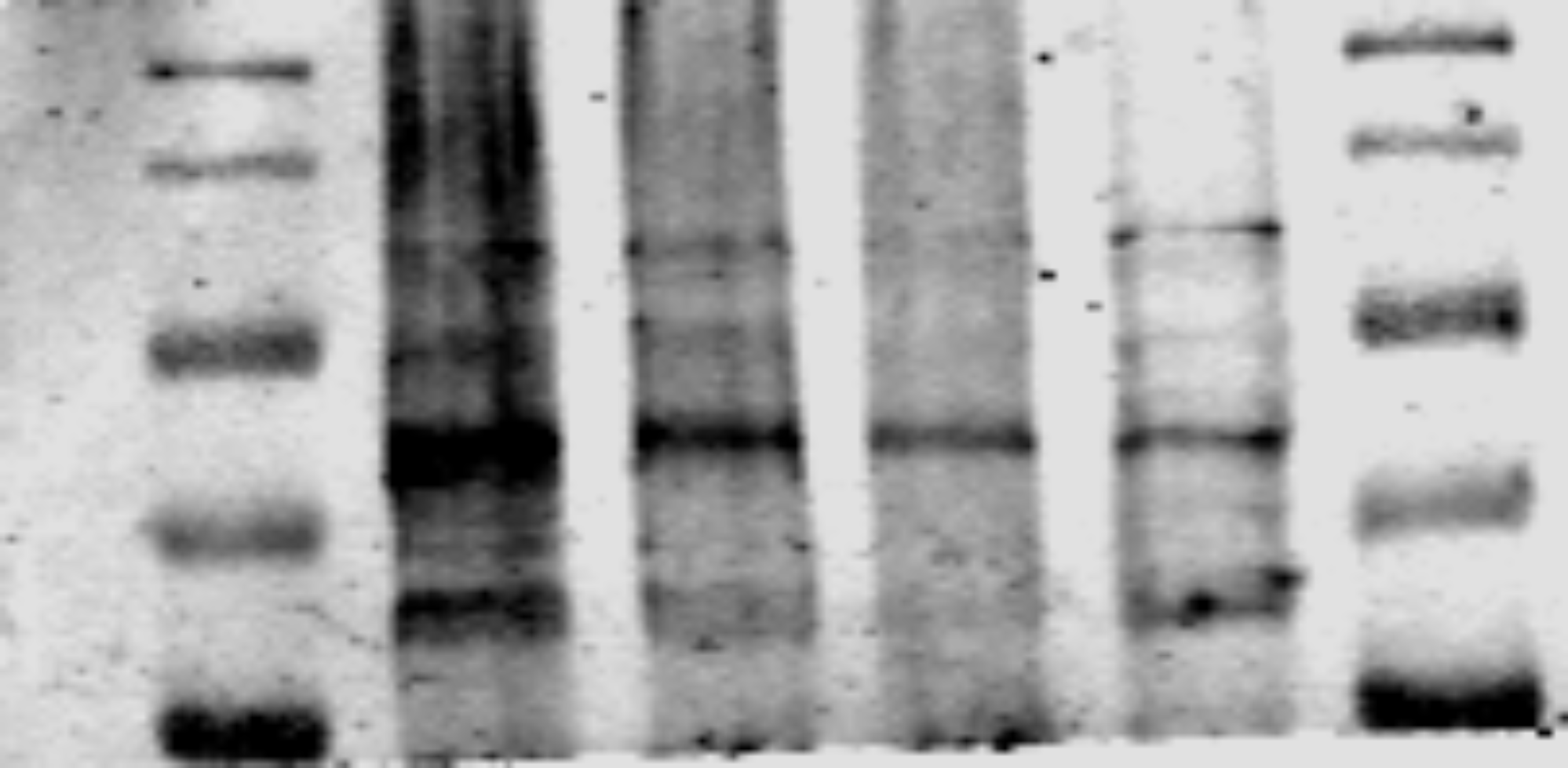

Supplement: Supplementary file 2 — Supplementary Information 2. [file 41598_2023_50476_MOESM2_ESM.zip › protein/3 repeat/5.pathway/5637/PAKT.tif]

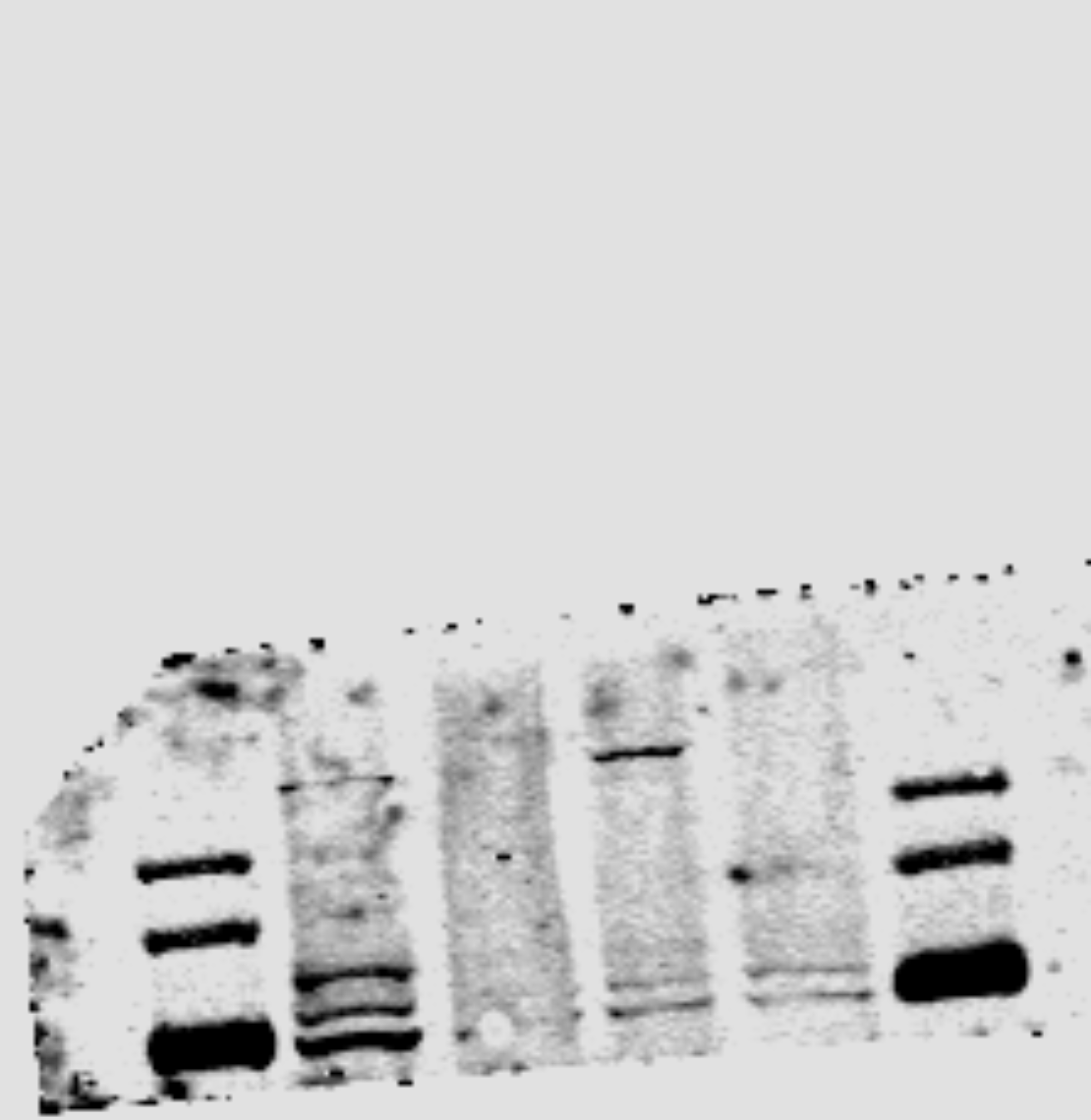

Supplement: Supplementary file 2 — Supplementary Information 2. [file 41598_2023_50476_MOESM2_ESM.zip › protein/3 repeat/5.pathway/5637/PI3K.tif]

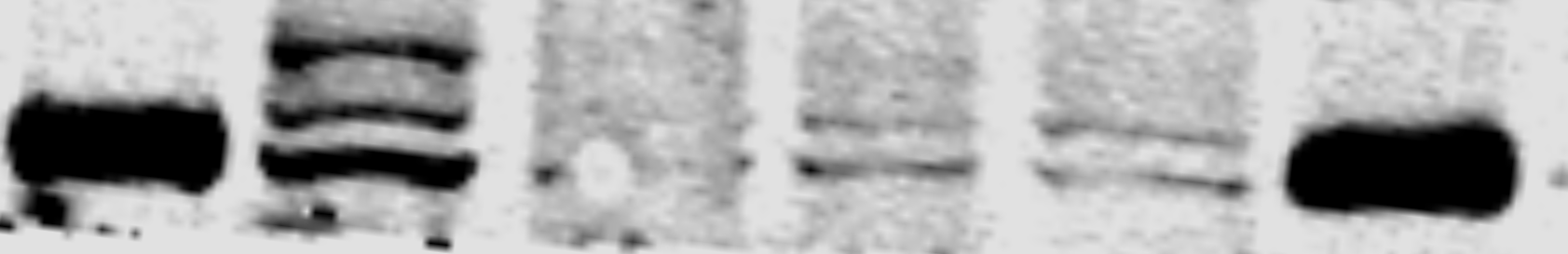

Supplement: Supplementary file 2 — Supplementary Information 2. [file 41598_2023_50476_MOESM2_ESM.zip › protein/3 repeat/5.pathway/5637/PI3KCUT.tif]

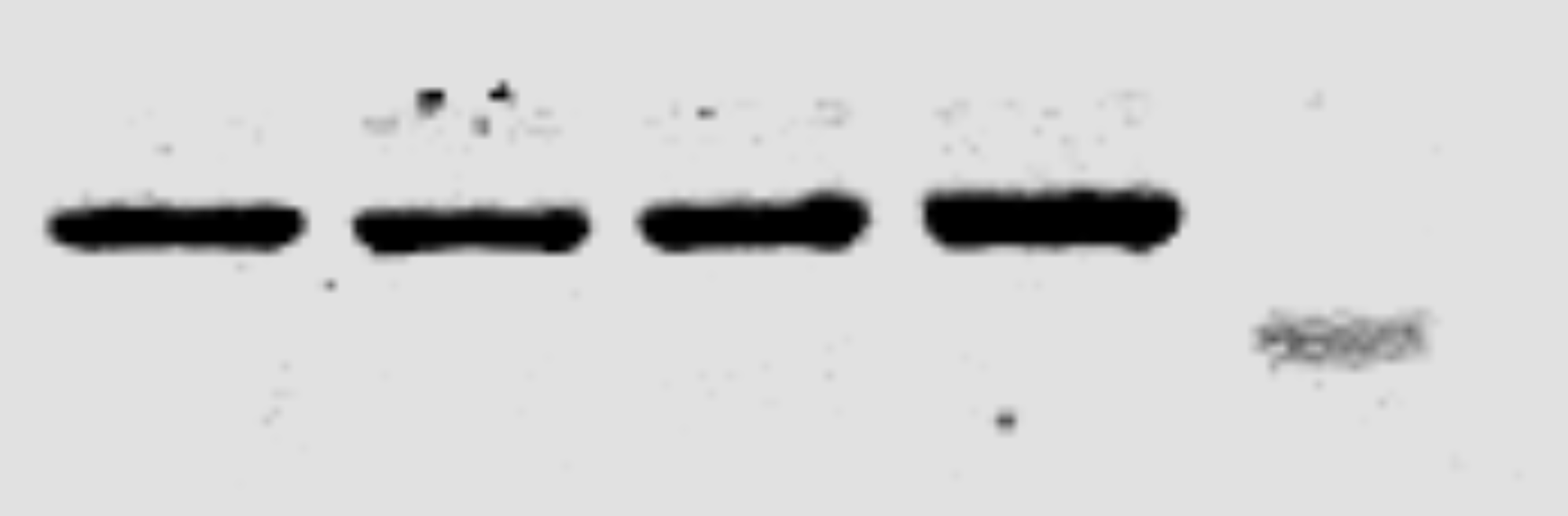

Supplement: Supplementary file 2 — Supplementary Information 2. [file 41598_2023_50476_MOESM2_ESM.zip › protein/3 repeat/5.pathway/T24/ACTIN.tif]

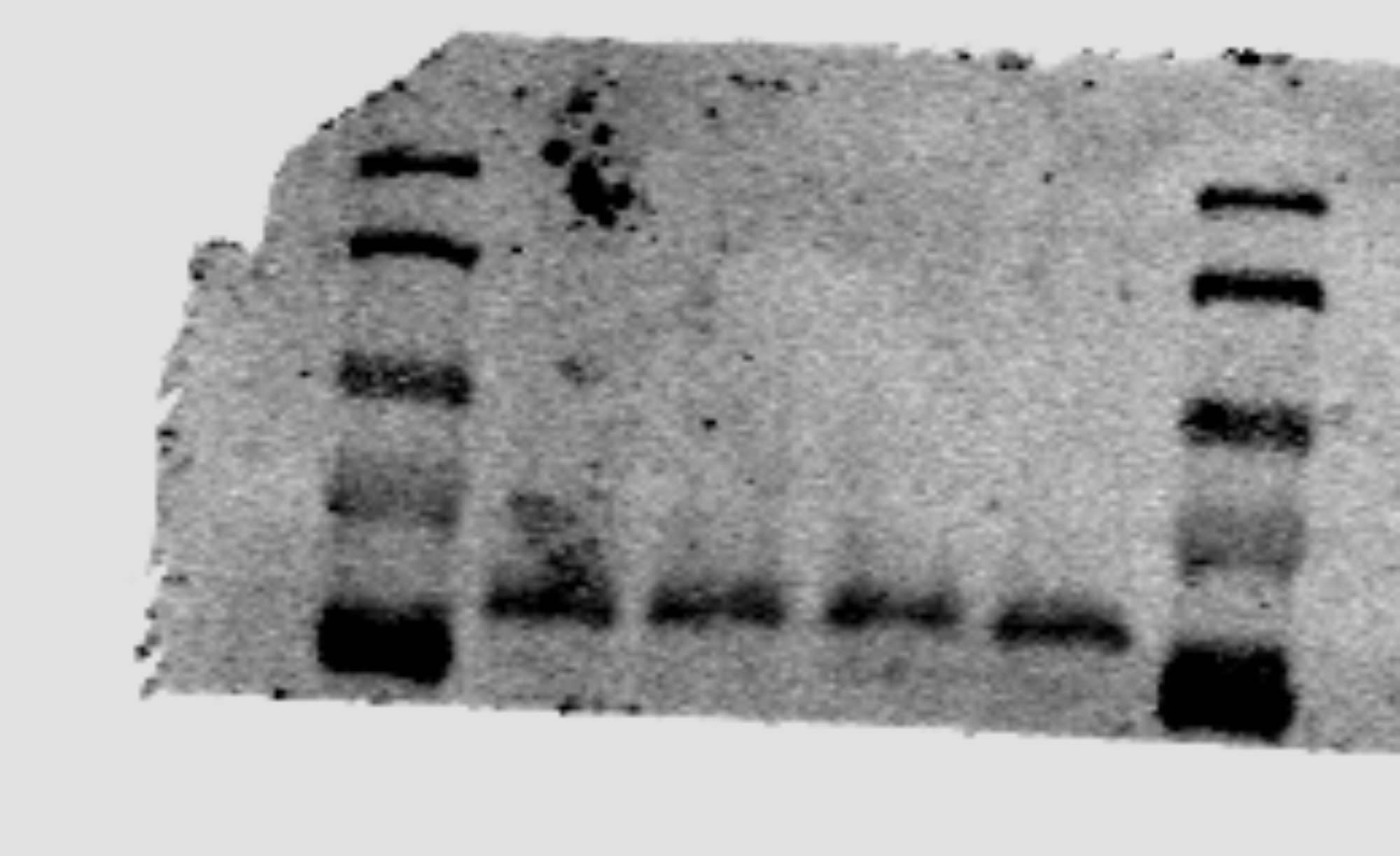

Supplement: Supplementary file 2 — Supplementary Information 2. [file 41598_2023_50476_MOESM2_ESM.zip › protein/3 repeat/5.pathway/T24/AKT.tif]

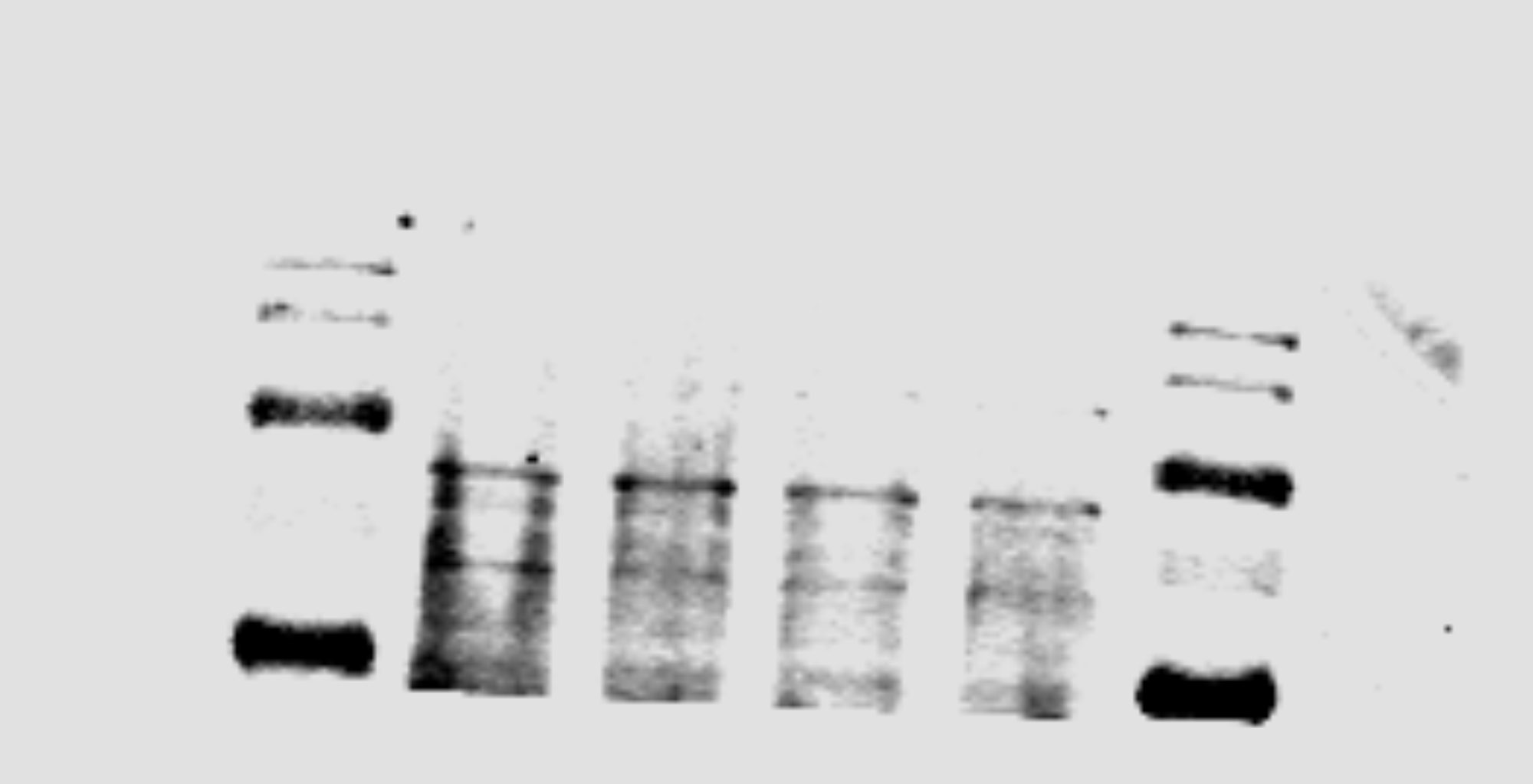

Supplement: Supplementary file 2 — Supplementary Information 2. [file 41598_2023_50476_MOESM2_ESM.zip › protein/3 repeat/5.pathway/T24/PAKT.tif]

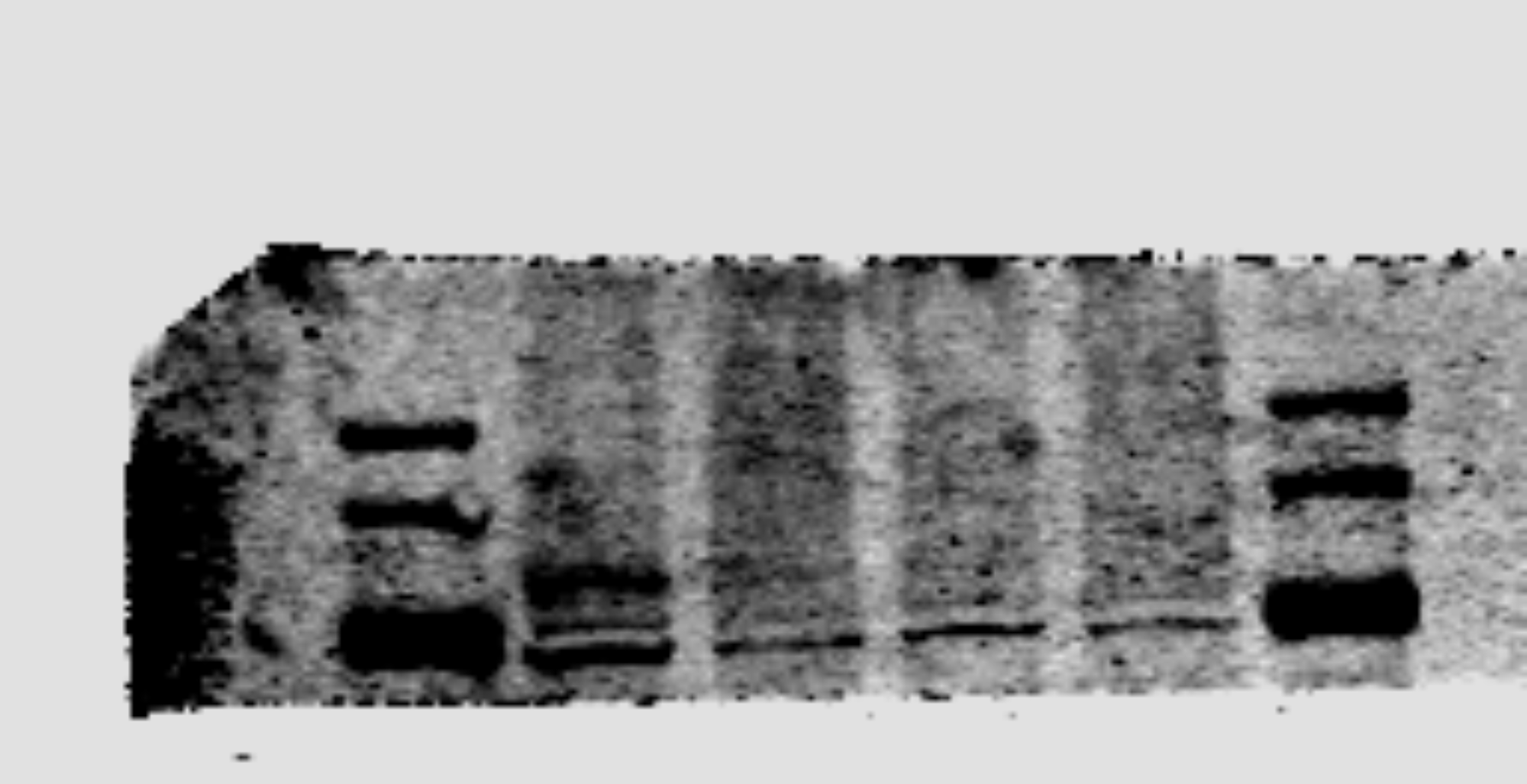

Supplement: Supplementary file 2 — Supplementary Information 2. [file 41598_2023_50476_MOESM2_ESM.zip › protein/3 repeat/5.pathway/T24/PI3K.tif]

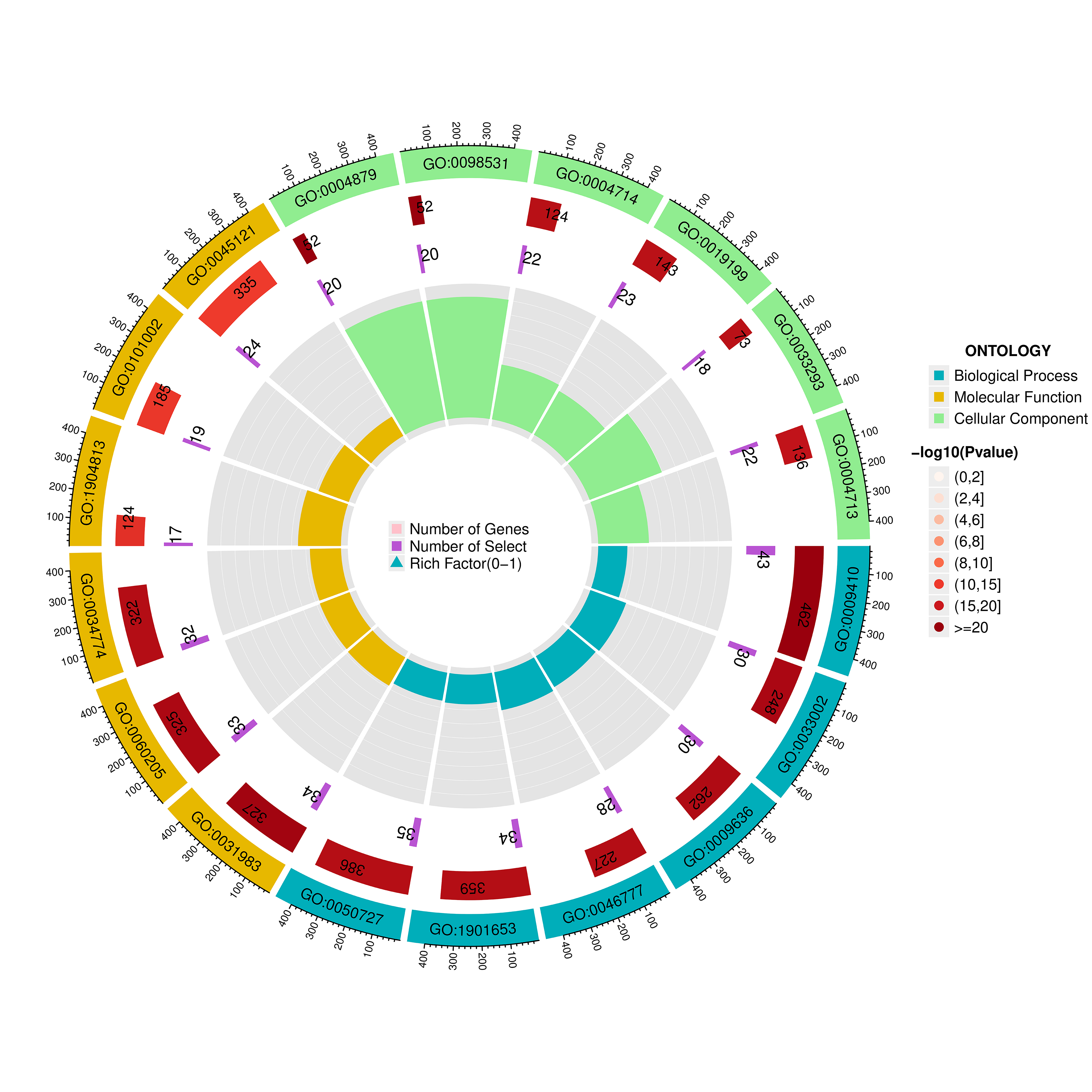

Supplement: Supplementary file 4 — Supplementary Figure 1. [file 41598_2023_50476_MOESM4_ESM.tif]

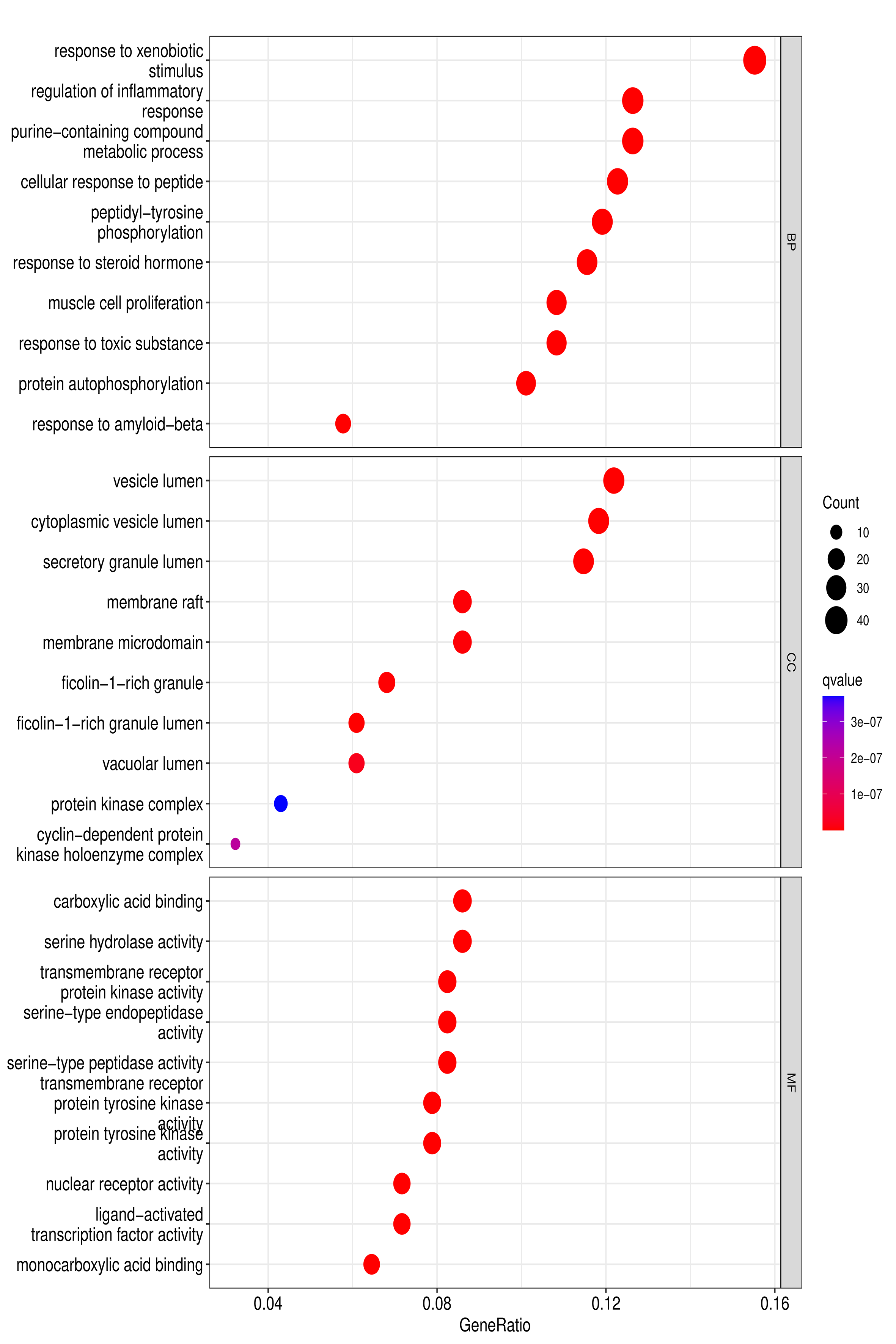

Supplement: Supplementary file 5 — Supplementary Figure 2. [file 41598_2023_50476_MOESM5_ESM.tif]

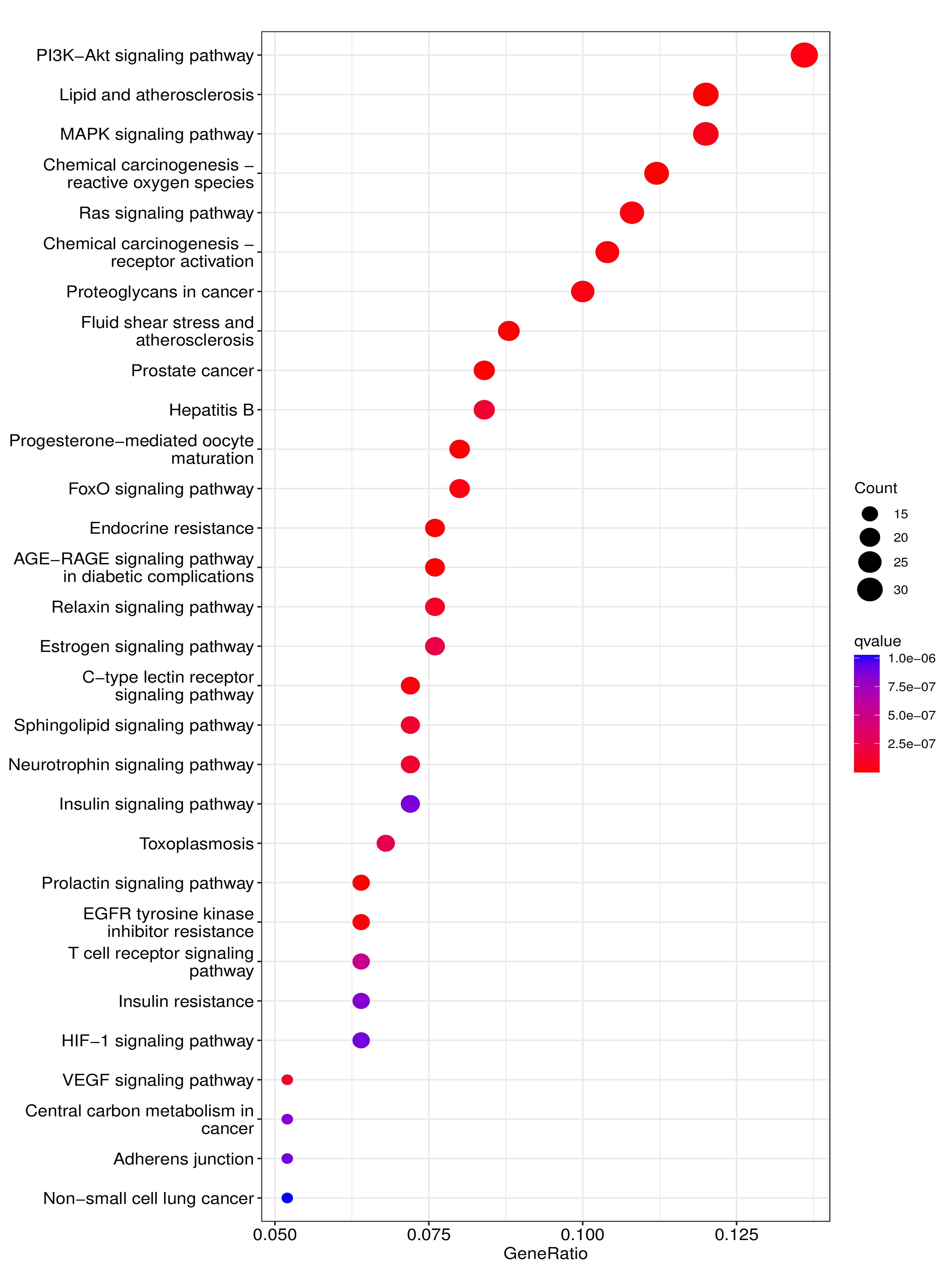

Supplement: Supplementary file 6 — Supplementary Figure 3. [file 41598_2023_50476_MOESM6_ESM.tif]
