# Supplementary figures and images for: Hesperetin promotes bladder cancer cells death via the PI3K/AKT pathway by network pharmacology and molecular docking (part 1 of 2)
Source: Sci Rep. 2024 Jan 10;14:1009. doi: 10.1038/s41598-023-50476-8 (PMC10781778; doi:10.1038/s41598-023-50476-8)

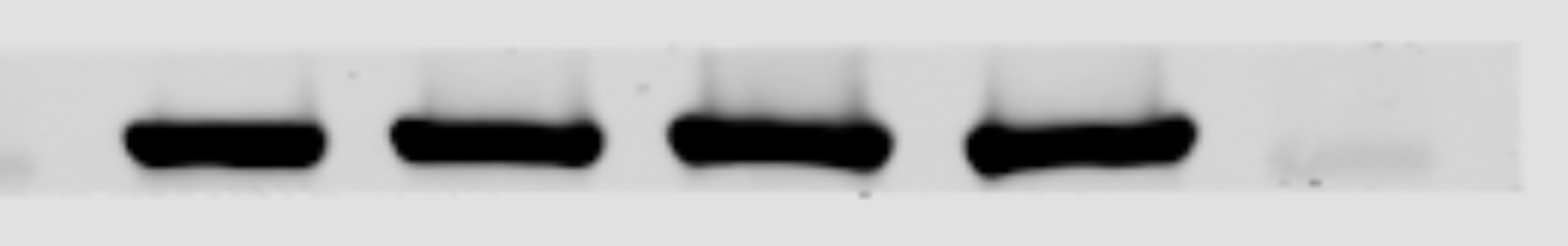

Supplement: Supplementary file 2 — Supplementary Information 2. [file 41598_2023_50476_MOESM2_ESM.zip › protein/1article/1.magration Figure6/5637/ACTIN .tif]

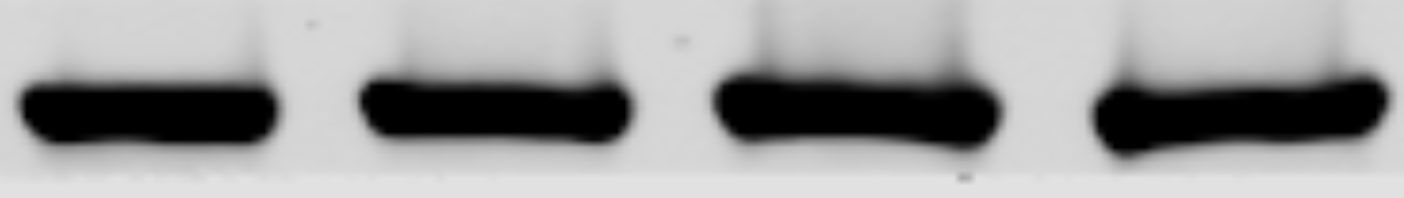

Supplement: Supplementary file 2 — Supplementary Information 2. [file 41598_2023_50476_MOESM2_ESM.zip › protein/1article/1.magration Figure6/5637/ACTIN Cut.tif]

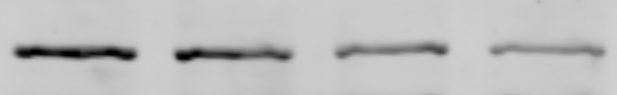

Supplement: Supplementary file 2 — Supplementary Information 2. [file 41598_2023_50476_MOESM2_ESM.zip › protein/1article/1.magration Figure6/5637/MMP2 Cut.png]

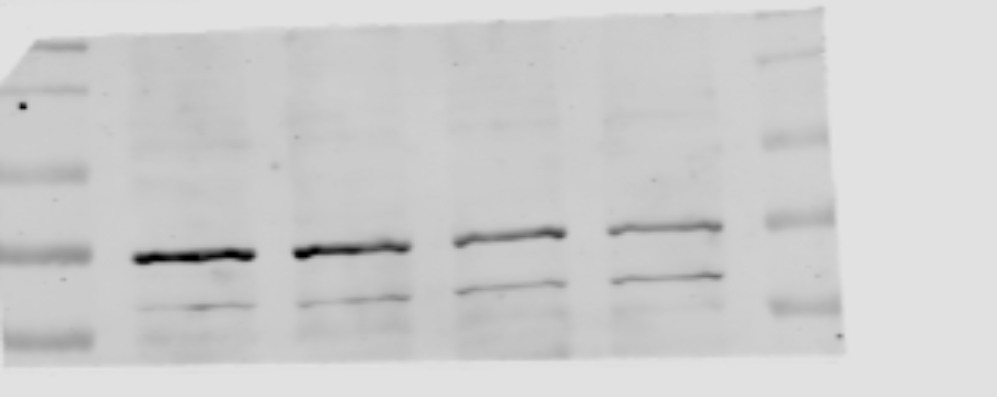

Supplement: Supplementary file 2 — Supplementary Information 2. [file 41598_2023_50476_MOESM2_ESM.zip › protein/1article/1.magration Figure6/5637/MMP2.png]

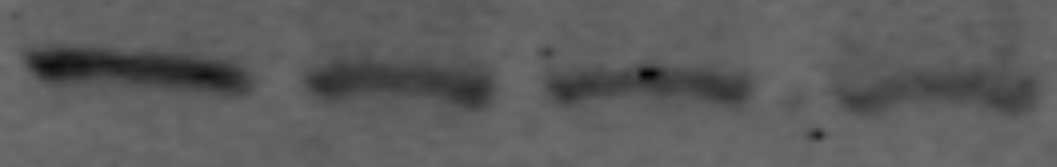

Supplement: Supplementary file 2 — Supplementary Information 2. [file 41598_2023_50476_MOESM2_ESM.zip › protein/1article/1.magration Figure6/5637/MMP9 Cut.tif]

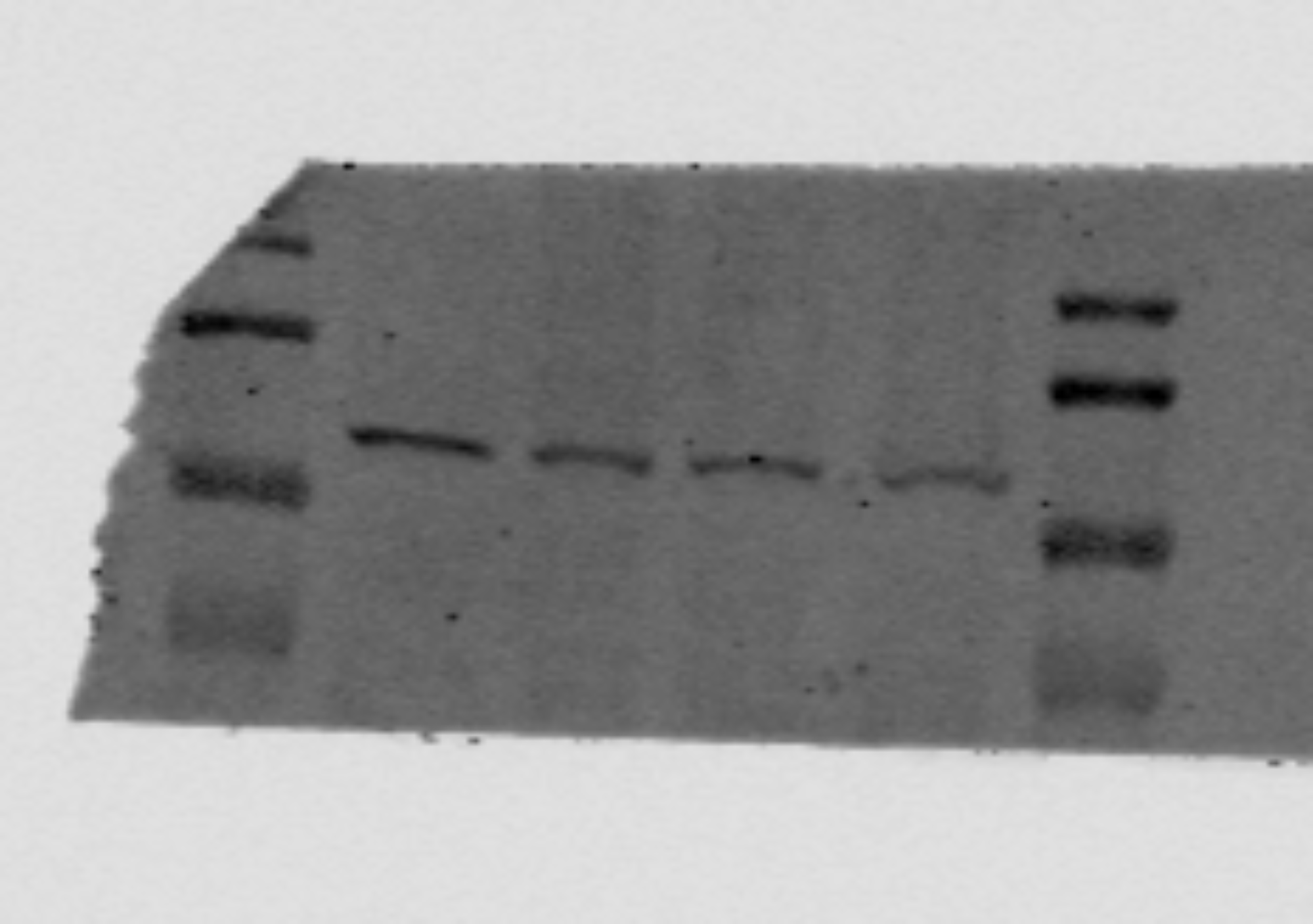

Supplement: Supplementary file 2 — Supplementary Information 2. [file 41598_2023_50476_MOESM2_ESM.zip › protein/1article/1.magration Figure6/5637/MMP9.tif]

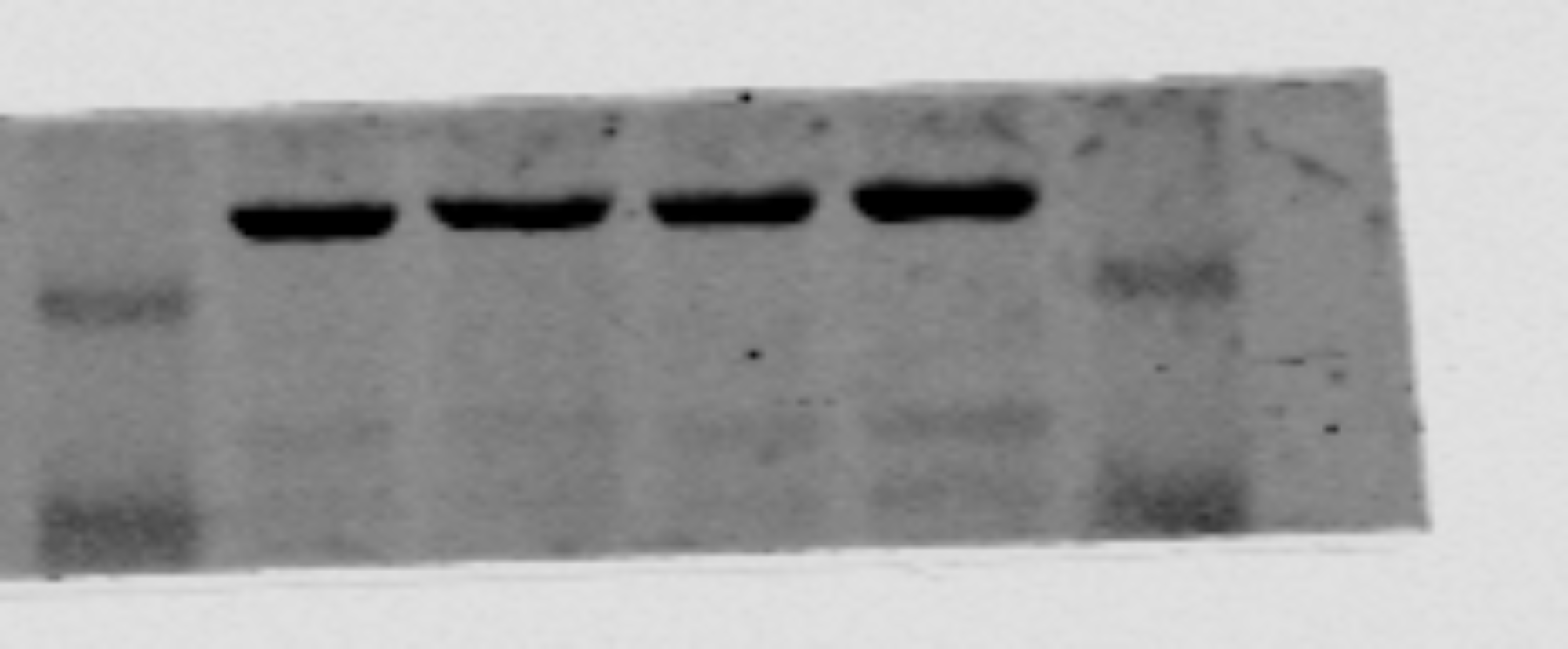

Supplement: Supplementary file 2 — Supplementary Information 2. [file 41598_2023_50476_MOESM2_ESM.zip › protein/1article/1.magration Figure6/T24/ACTIN (1).tif]

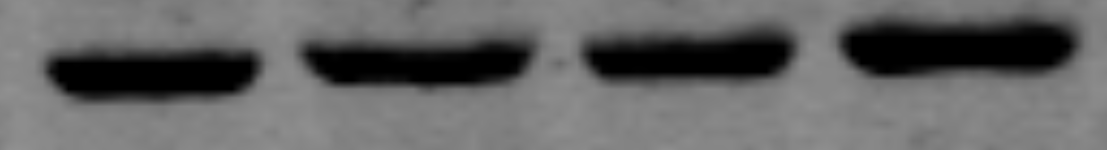

Supplement: Supplementary file 2 — Supplementary Information 2. [file 41598_2023_50476_MOESM2_ESM.zip › protein/1article/1.magration Figure6/T24/ACTIN Cut.tif]

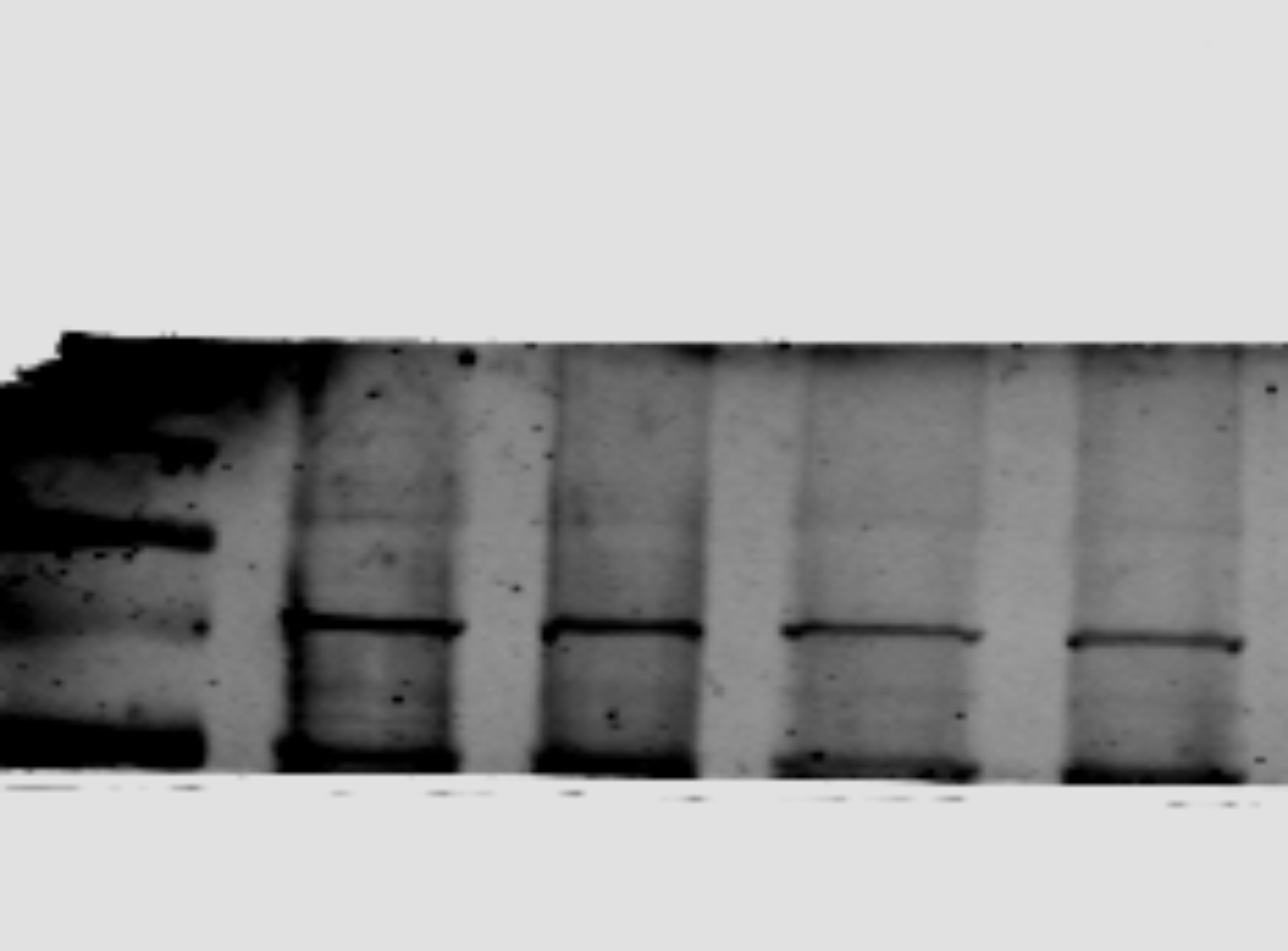

Supplement: Supplementary file 2 — Supplementary Information 2. [file 41598_2023_50476_MOESM2_ESM.zip › protein/1article/1.magration Figure6/T24/MMP2 .tif]

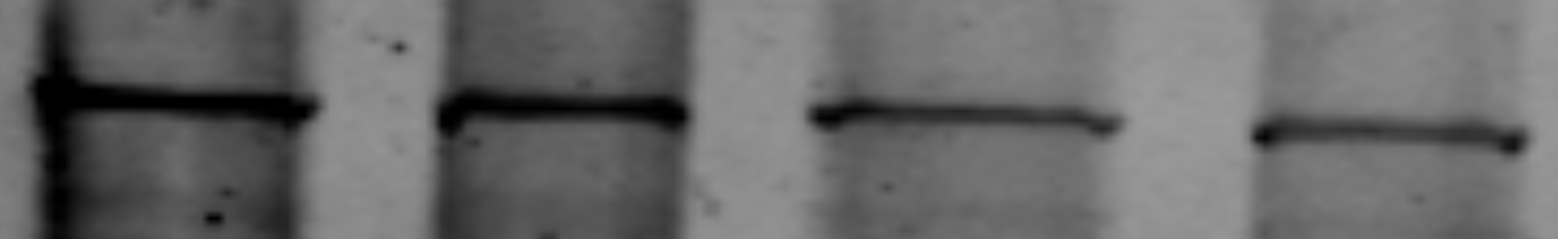

Supplement: Supplementary file 2 — Supplementary Information 2. [file 41598_2023_50476_MOESM2_ESM.zip › protein/1article/1.magration Figure6/T24/MMP2 Cut.tif]

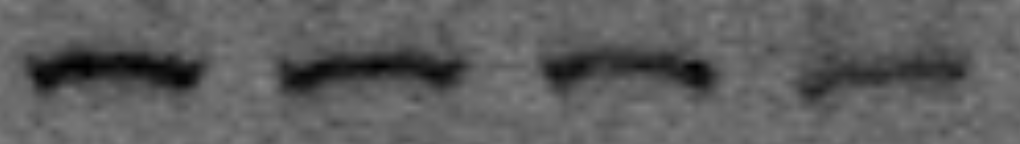

Supplement: Supplementary file 2 — Supplementary Information 2. [file 41598_2023_50476_MOESM2_ESM.zip › protein/1article/1.magration Figure6/T24/MMP9 Cut.tif]

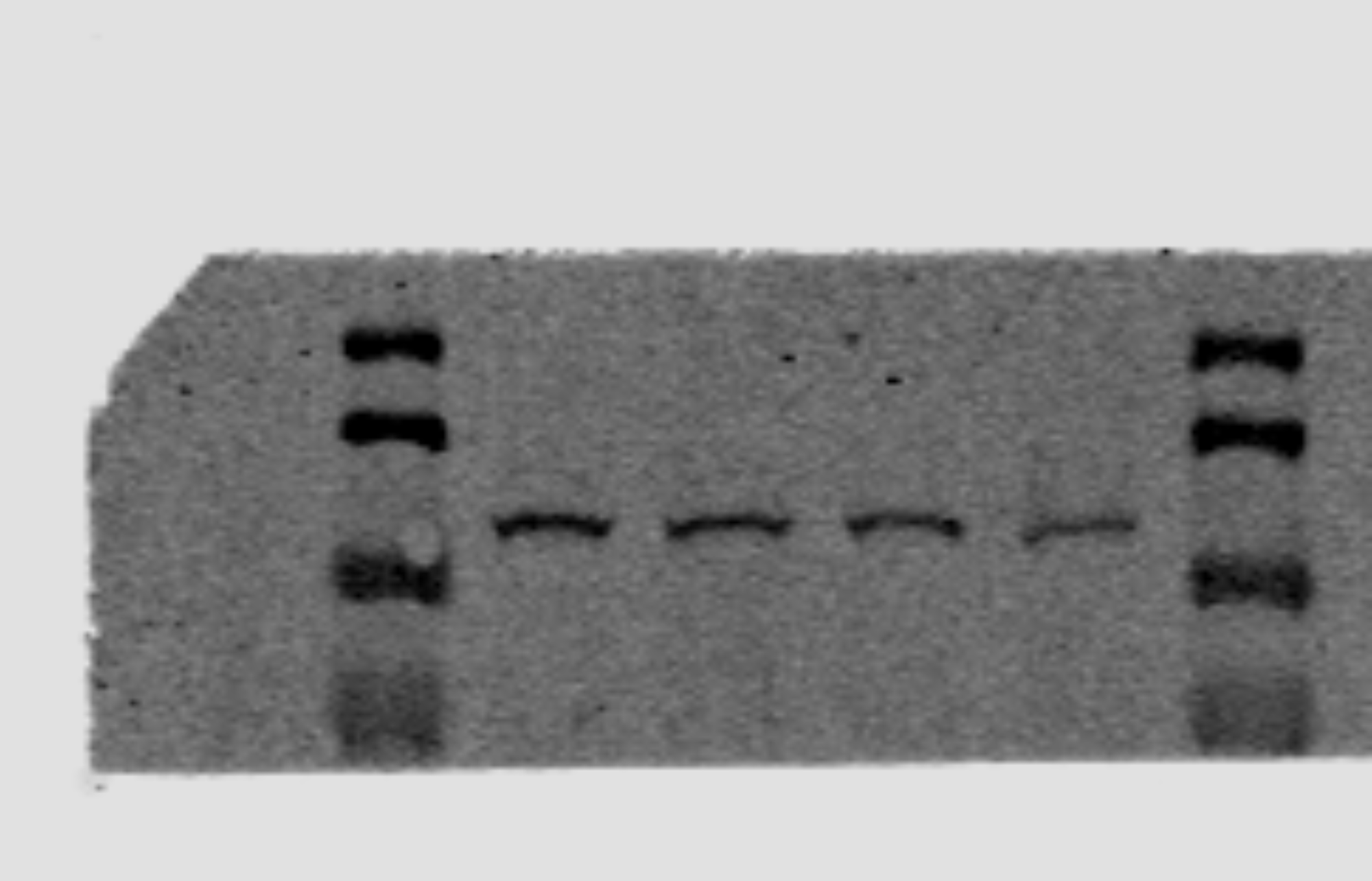

Supplement: Supplementary file 2 — Supplementary Information 2. [file 41598_2023_50476_MOESM2_ESM.zip › protein/1article/1.magration Figure6/T24/MMP9.tif]

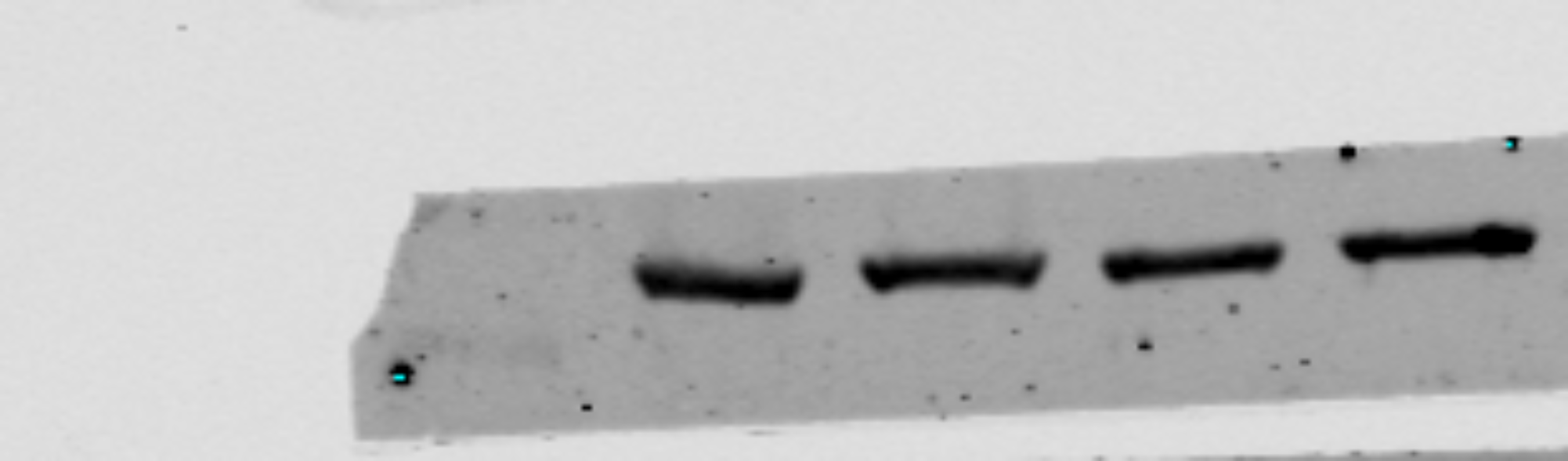

Supplement: Supplementary file 2 — Supplementary Information 2. [file 41598_2023_50476_MOESM2_ESM.zip › protein/1article/2.apoptosis Figure7/5637/ACTIN.tif]

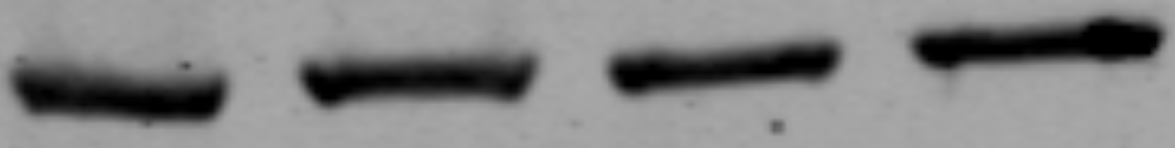

Supplement: Supplementary file 2 — Supplementary Information 2. [file 41598_2023_50476_MOESM2_ESM.zip › protein/1article/2.apoptosis Figure7/5637/ACTINCUT.tif]

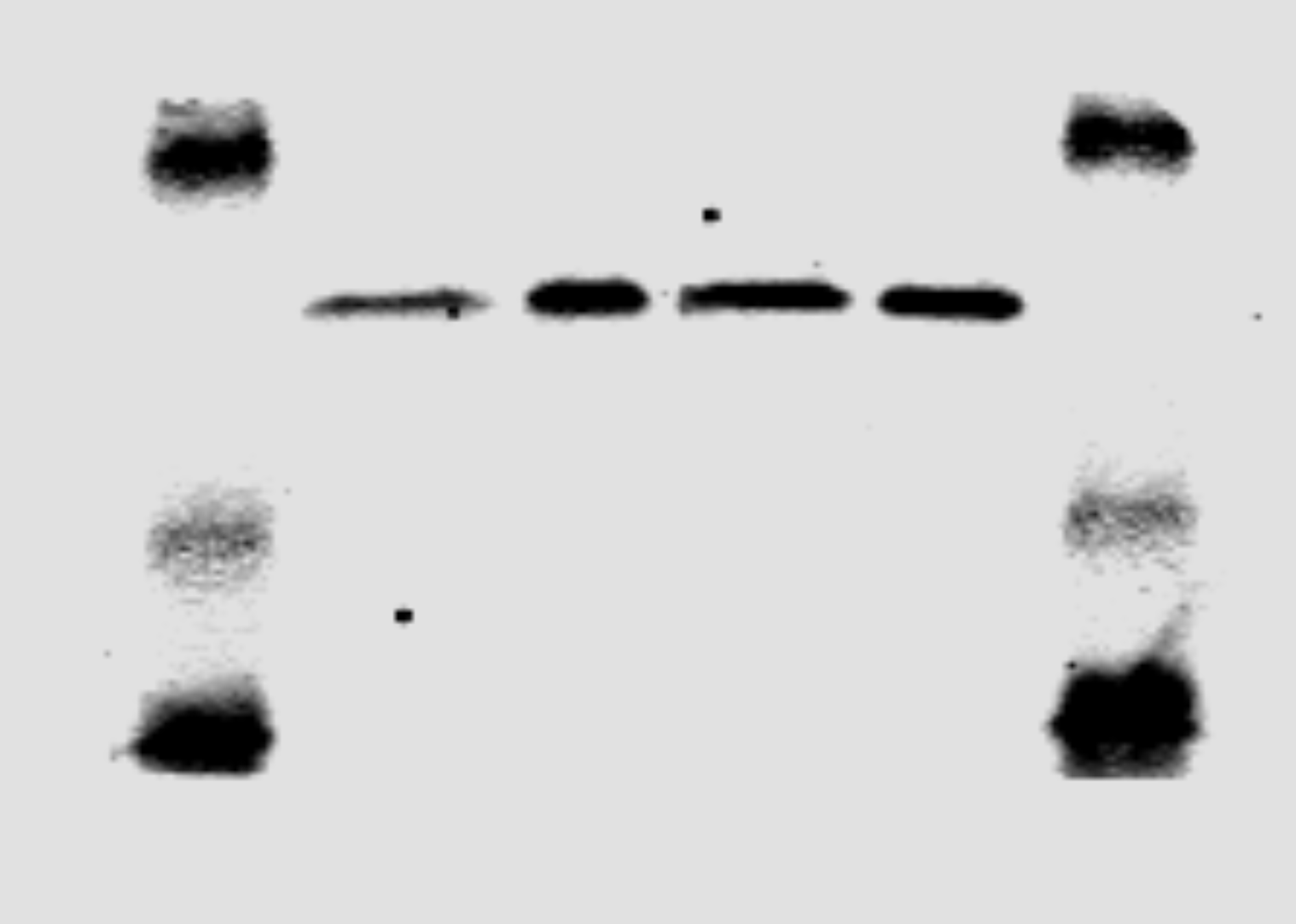

Supplement: Supplementary file 2 — Supplementary Information 2. [file 41598_2023_50476_MOESM2_ESM.zip › protein/1article/2.apoptosis Figure7/5637/BAX.tif]

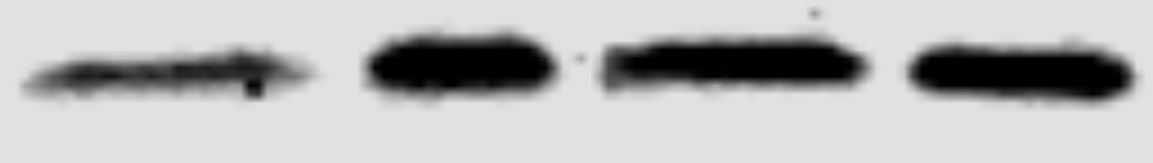

Supplement: Supplementary file 2 — Supplementary Information 2. [file 41598_2023_50476_MOESM2_ESM.zip › protein/1article/2.apoptosis Figure7/5637/BAXCUT.tif]

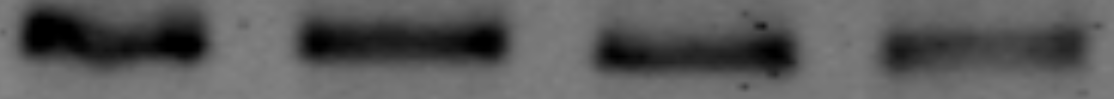

Supplement: Supplementary file 2 — Supplementary Information 2. [file 41598_2023_50476_MOESM2_ESM.zip › protein/1article/2.apoptosis Figure7/5637/BCL-2 cut.tif]

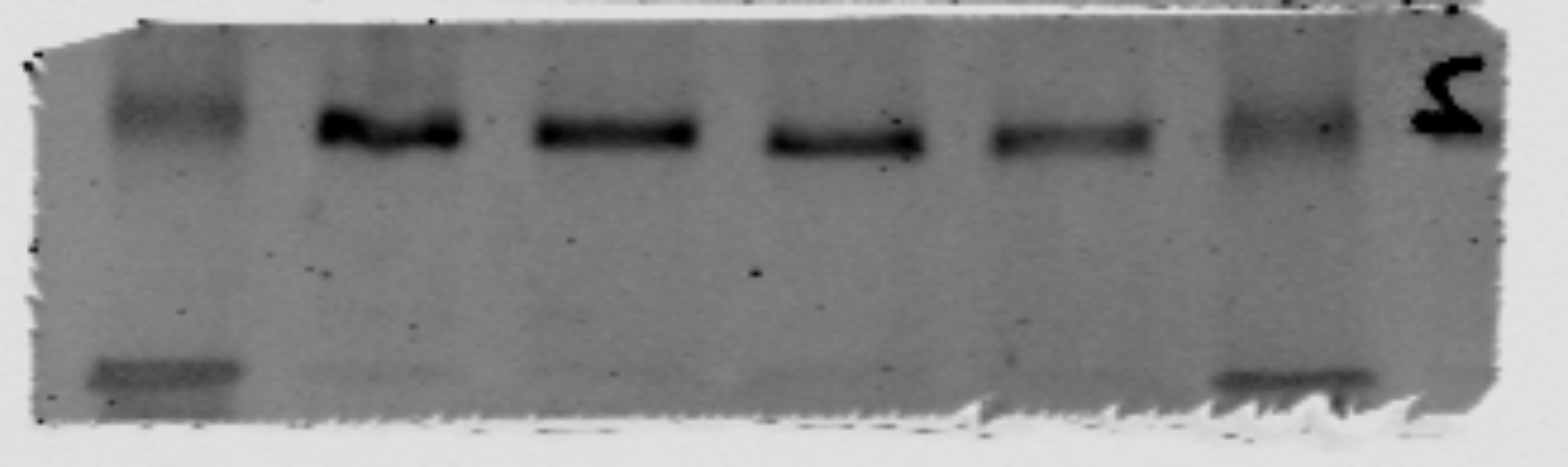

Supplement: Supplementary file 2 — Supplementary Information 2. [file 41598_2023_50476_MOESM2_ESM.zip › protein/1article/2.apoptosis Figure7/5637/BCL-2.tif]

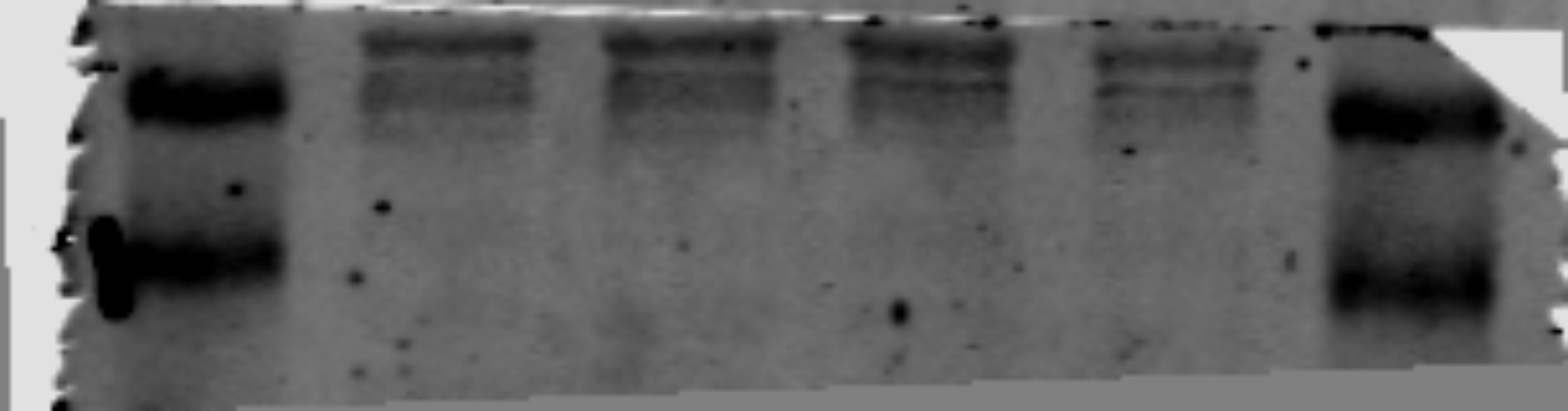

Supplement: Supplementary file 2 — Supplementary Information 2. [file 41598_2023_50476_MOESM2_ESM.zip › protein/1article/2.apoptosis Figure7/5637/caspase-3.tif]

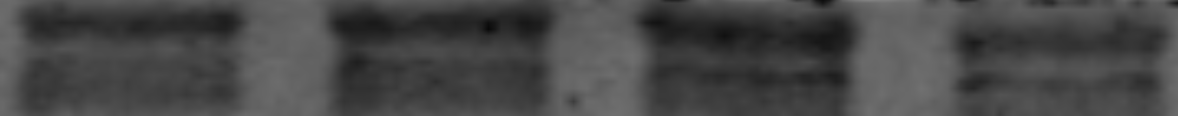

Supplement: Supplementary file 2 — Supplementary Information 2. [file 41598_2023_50476_MOESM2_ESM.zip › protein/1article/2.apoptosis Figure7/5637/caspase-3Cut.tif]

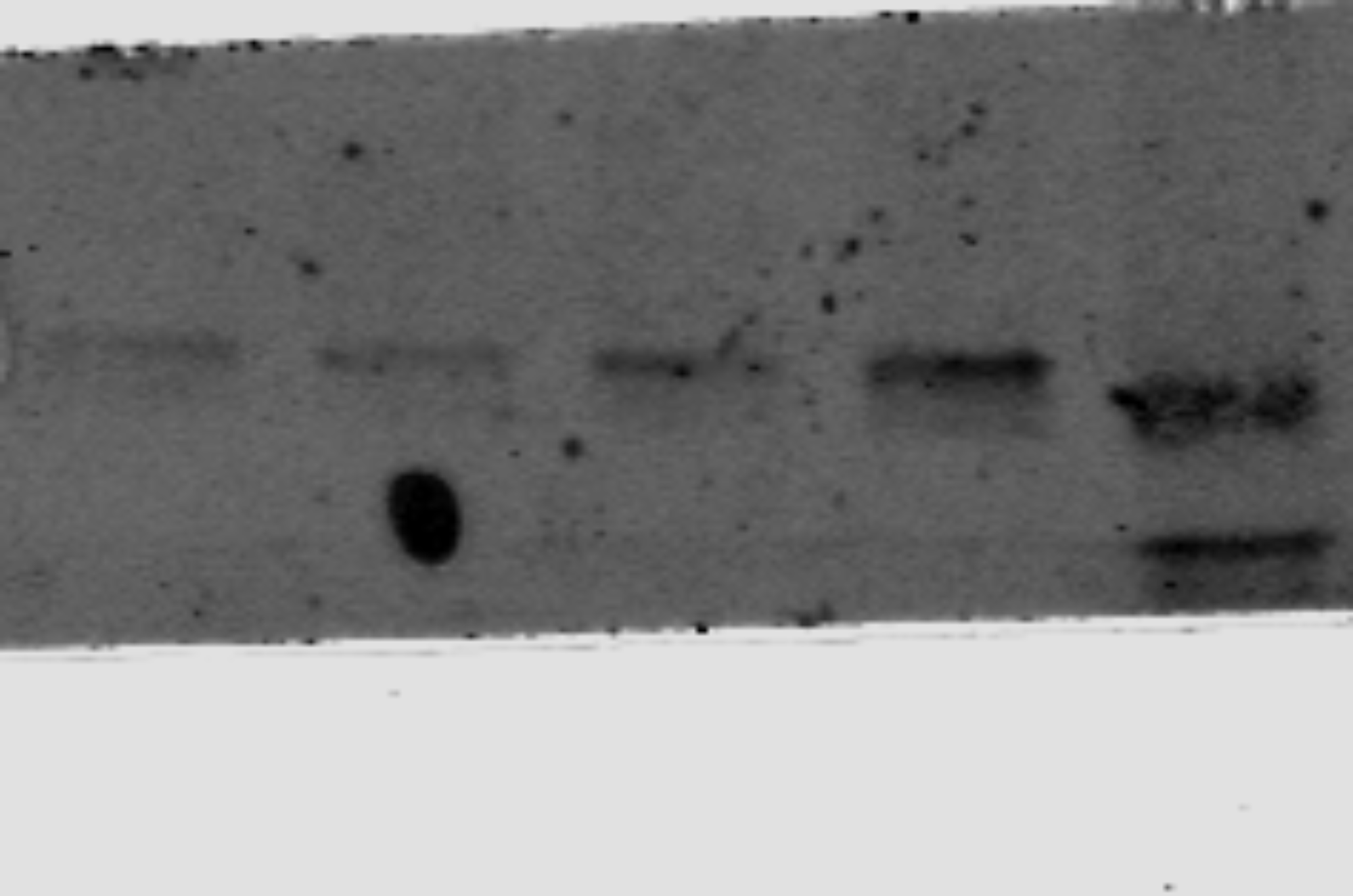

Supplement: Supplementary file 2 — Supplementary Information 2. [file 41598_2023_50476_MOESM2_ESM.zip › protein/1article/2.apoptosis Figure7/5637/cleaved caspase-3.png]

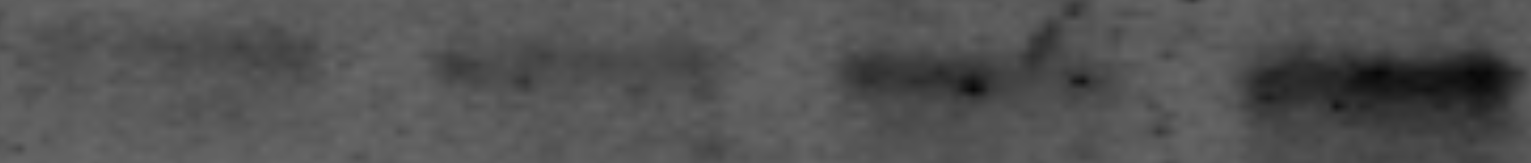

Supplement: Supplementary file 2 — Supplementary Information 2. [file 41598_2023_50476_MOESM2_ESM.zip › protein/1article/2.apoptosis Figure7/5637/cleaved caspase-3cut.png]

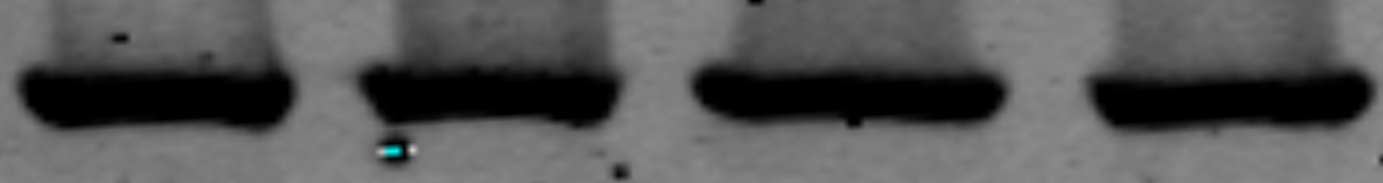

Supplement: Supplementary file 2 — Supplementary Information 2. [file 41598_2023_50476_MOESM2_ESM.zip › protein/1article/2.apoptosis Figure7/T24/ACTIN Cut.tif]

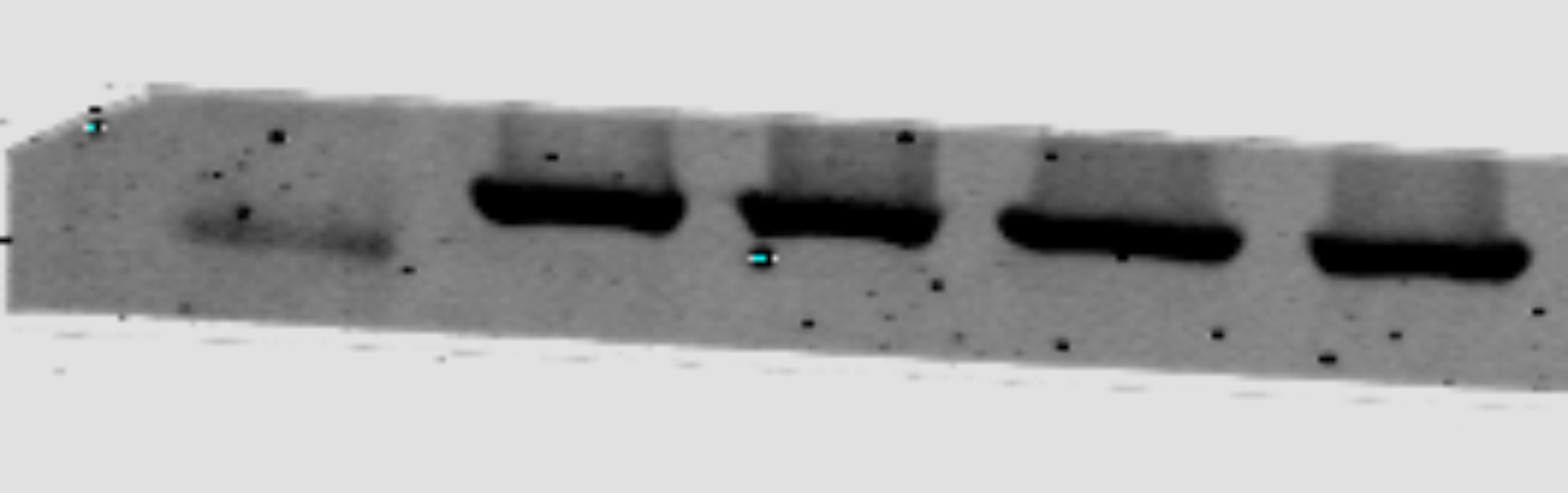

Supplement: Supplementary file 2 — Supplementary Information 2. [file 41598_2023_50476_MOESM2_ESM.zip › protein/1article/2.apoptosis Figure7/T24/ACTIN.tif]

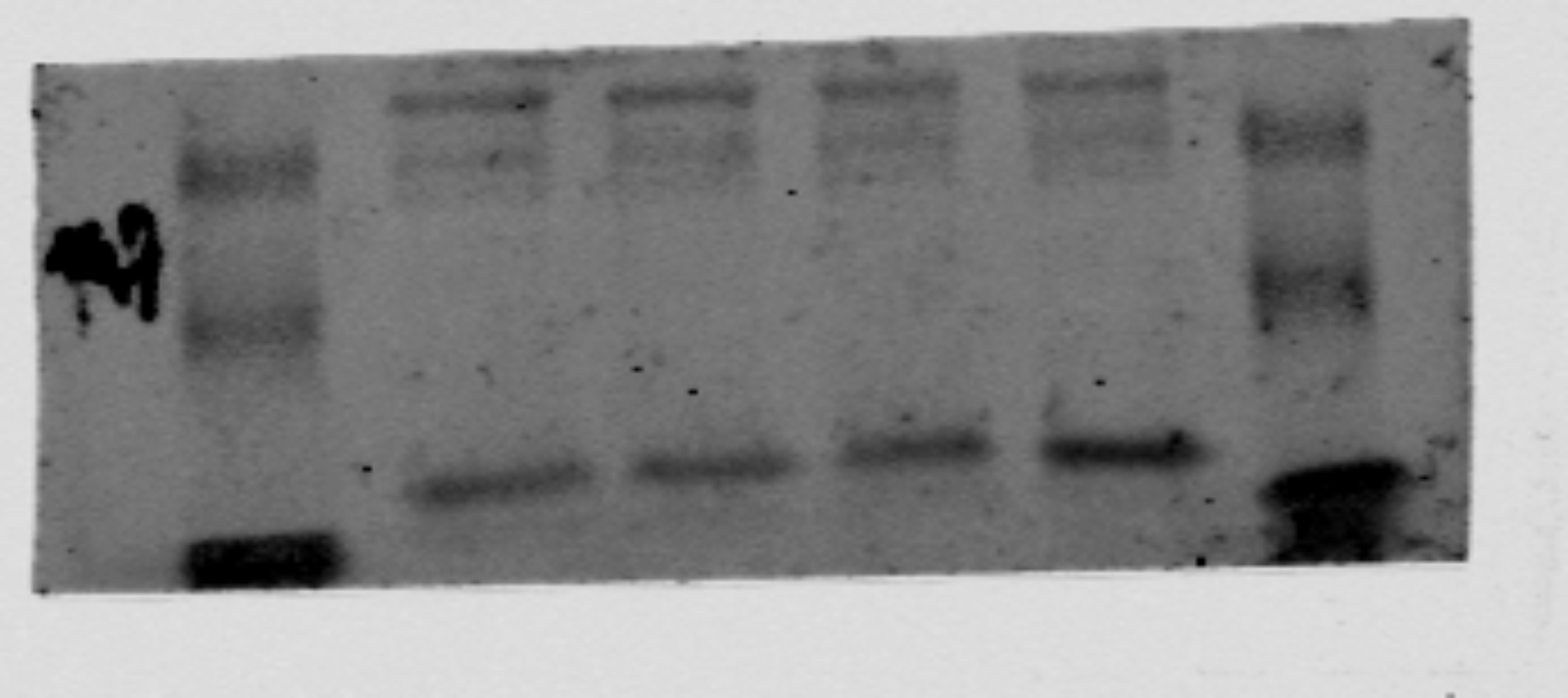

Supplement: Supplementary file 2 — Supplementary Information 2. [file 41598_2023_50476_MOESM2_ESM.zip › protein/1article/2.apoptosis Figure7/T24/BAX.tif]

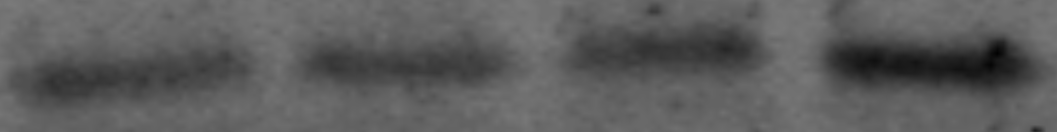

Supplement: Supplementary file 2 — Supplementary Information 2. [file 41598_2023_50476_MOESM2_ESM.zip › protein/1article/2.apoptosis Figure7/T24/BAXCUTtif.tif]

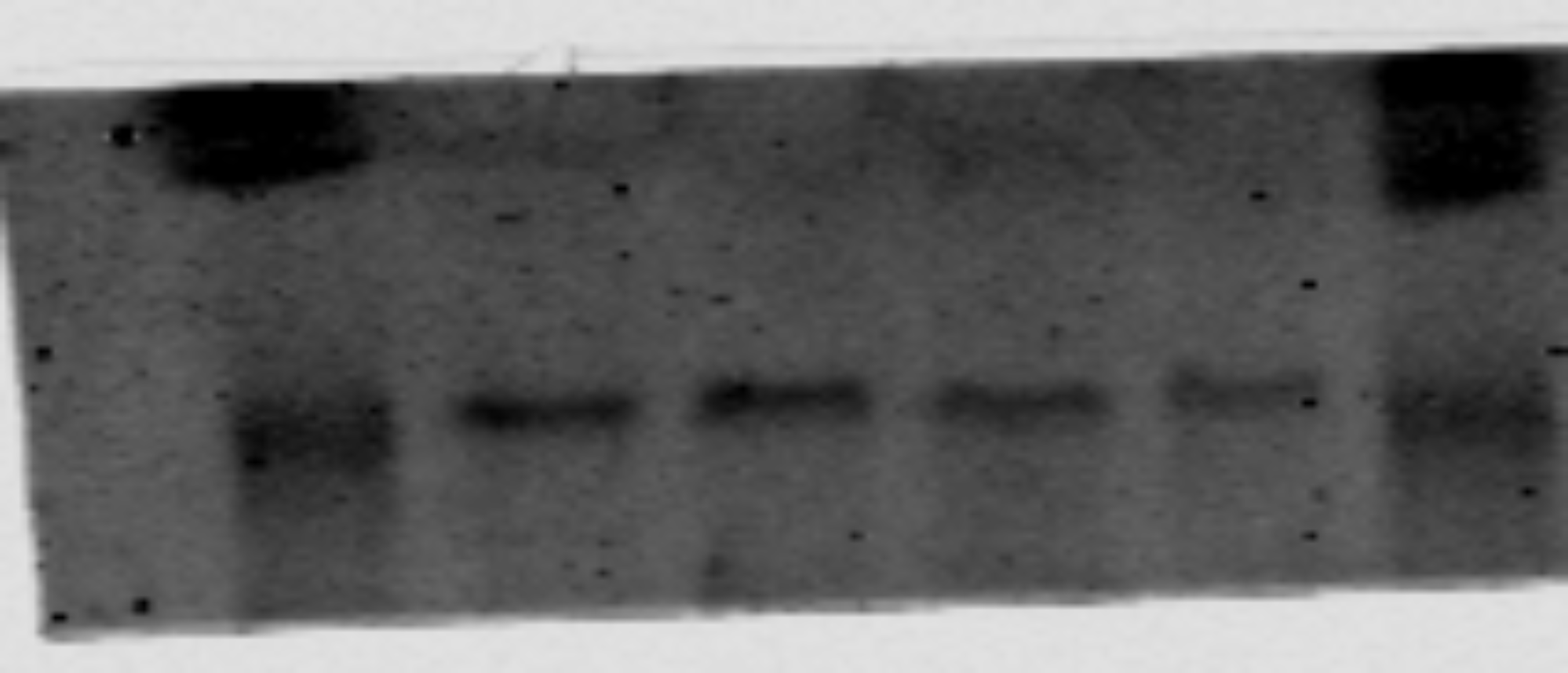

Supplement: Supplementary file 2 — Supplementary Information 2. [file 41598_2023_50476_MOESM2_ESM.zip › protein/1article/2.apoptosis Figure7/T24/BCL-2.tif]

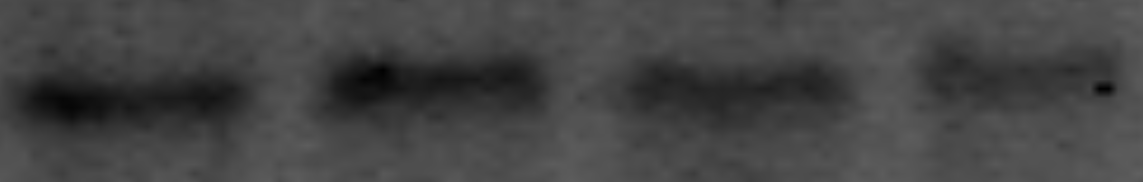

Supplement: Supplementary file 2 — Supplementary Information 2. [file 41598_2023_50476_MOESM2_ESM.zip › protein/1article/2.apoptosis Figure7/T24/BCL-2Cut.tif]

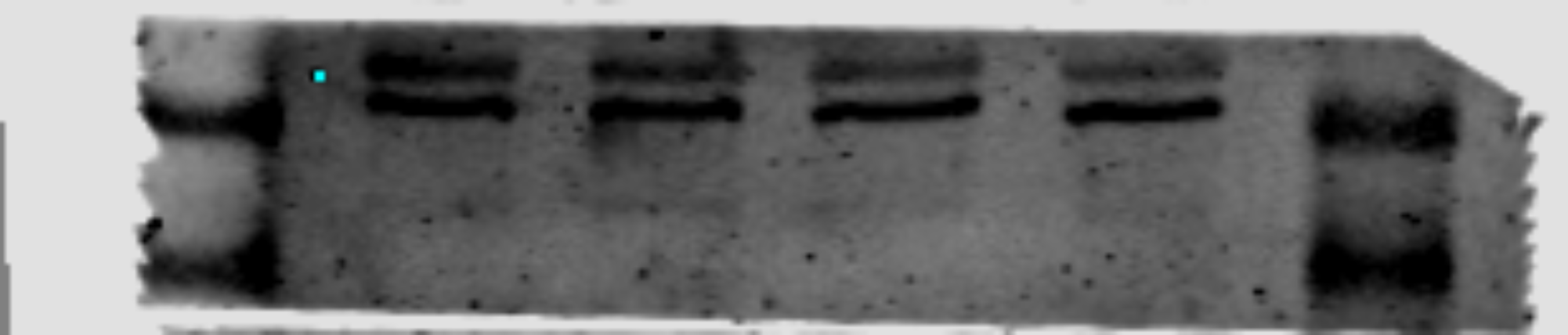

Supplement: Supplementary file 2 — Supplementary Information 2. [file 41598_2023_50476_MOESM2_ESM.zip › protein/1article/2.apoptosis Figure7/T24/capase-3.tif]

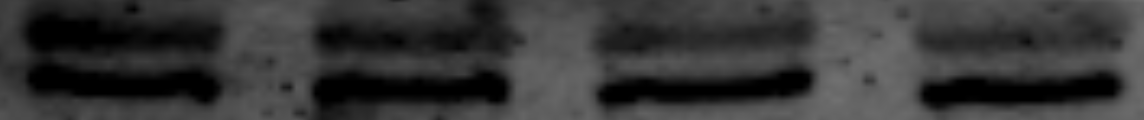

Supplement: Supplementary file 2 — Supplementary Information 2. [file 41598_2023_50476_MOESM2_ESM.zip › protein/1article/2.apoptosis Figure7/T24/capase-3Cut.tif]

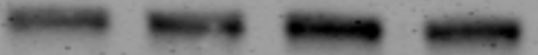

Supplement: Supplementary file 2 — Supplementary Information 2. [file 41598_2023_50476_MOESM2_ESM.zip › protein/1article/2.apoptosis Figure7/T24/cleaved caspase-3 cut.tif]

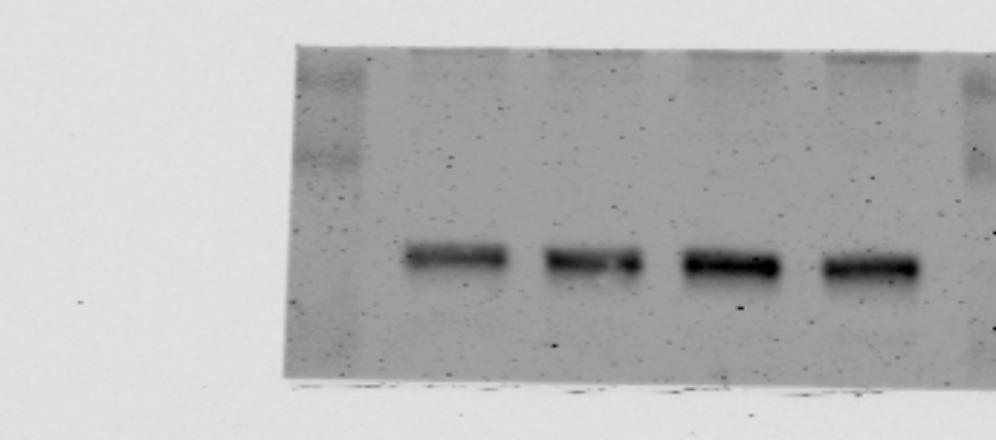

Supplement: Supplementary file 2 — Supplementary Information 2. [file 41598_2023_50476_MOESM2_ESM.zip › protein/1article/2.apoptosis Figure7/T24/cleaved caspase-3.tif]

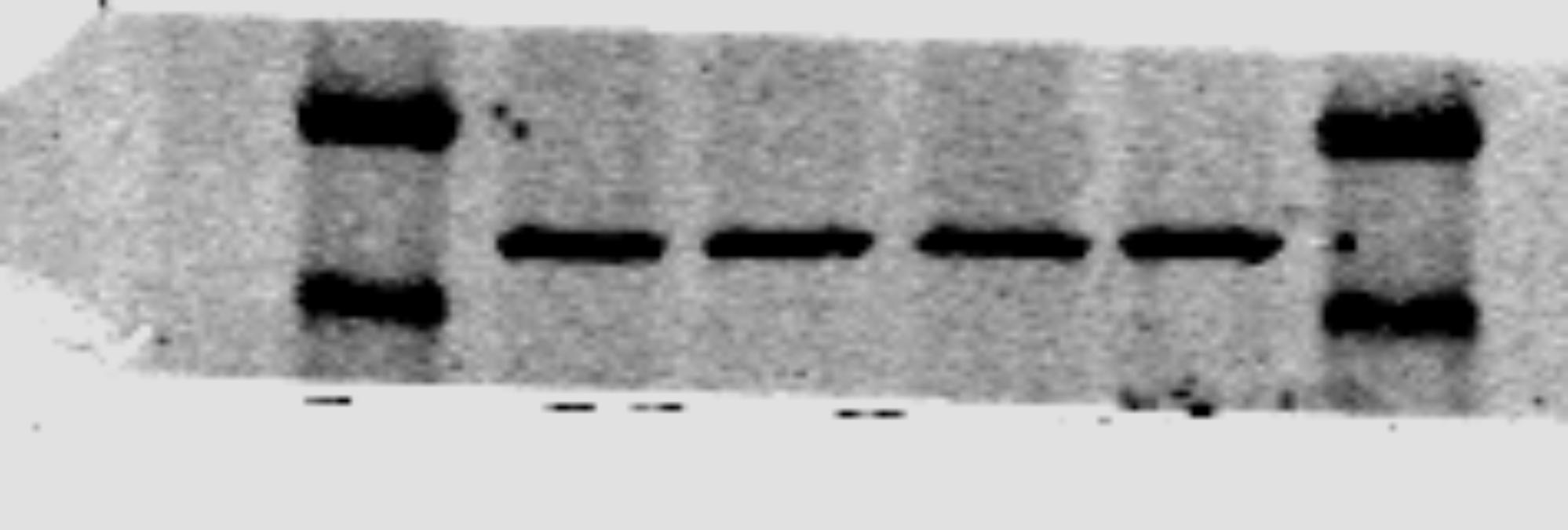

Supplement: Supplementary file 2 — Supplementary Information 2. [file 41598_2023_50476_MOESM2_ESM.zip › protein/1article/3.ferroptosis Figure8/5637/ACTIN.tif]

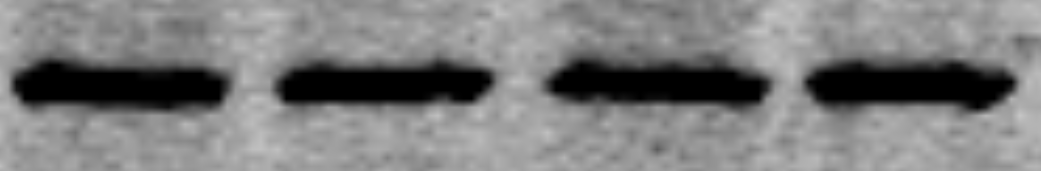

Supplement: Supplementary file 2 — Supplementary Information 2. [file 41598_2023_50476_MOESM2_ESM.zip › protein/1article/3.ferroptosis Figure8/5637/ACTINcut.tif]

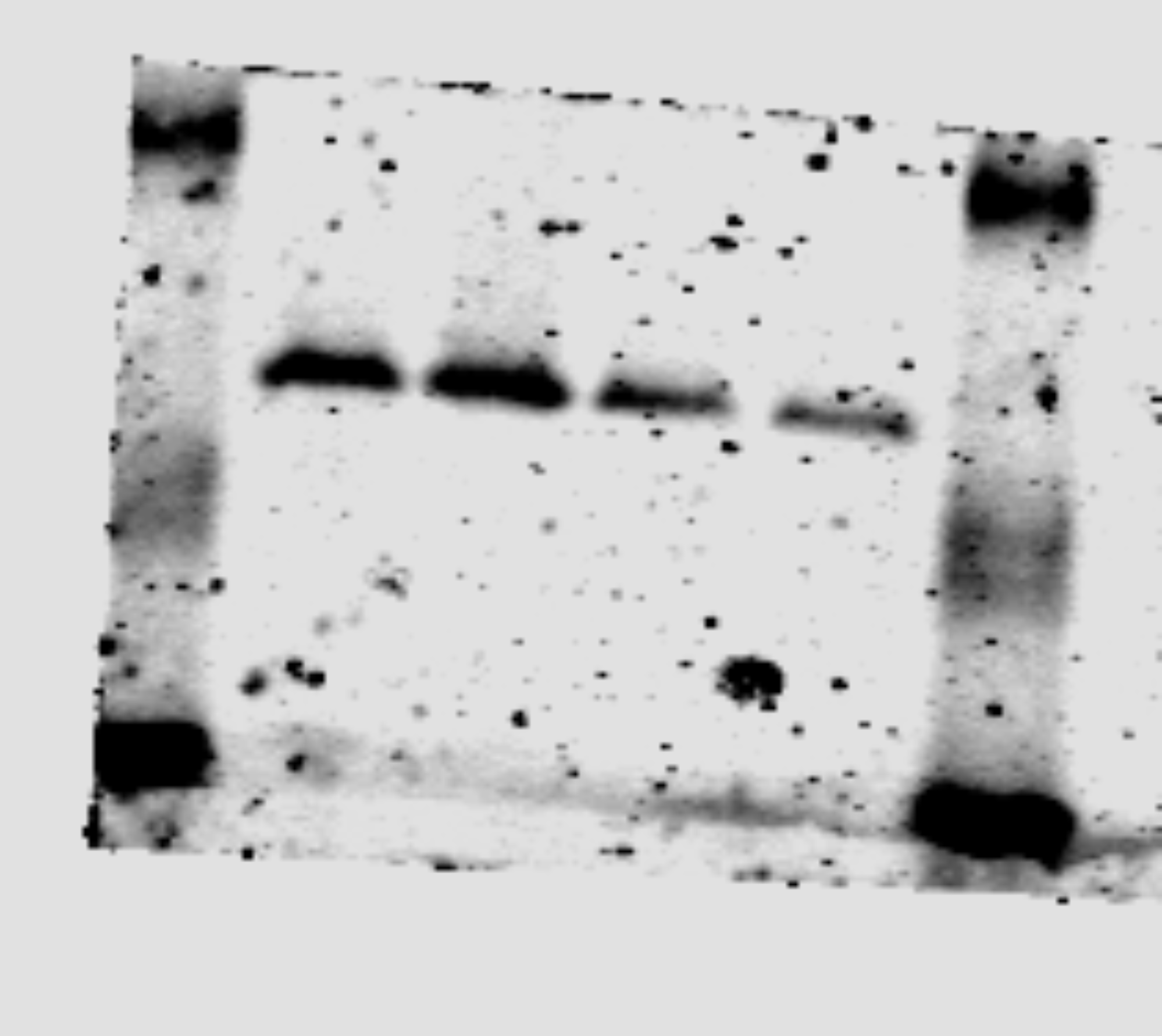

Supplement: Supplementary file 2 — Supplementary Information 2. [file 41598_2023_50476_MOESM2_ESM.zip › protein/1article/3.ferroptosis Figure8/5637/GPX4.tif]

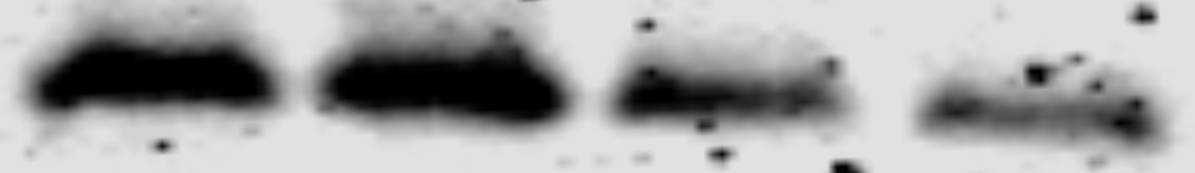

Supplement: Supplementary file 2 — Supplementary Information 2. [file 41598_2023_50476_MOESM2_ESM.zip › protein/1article/3.ferroptosis Figure8/5637/GPX4cut.tif]

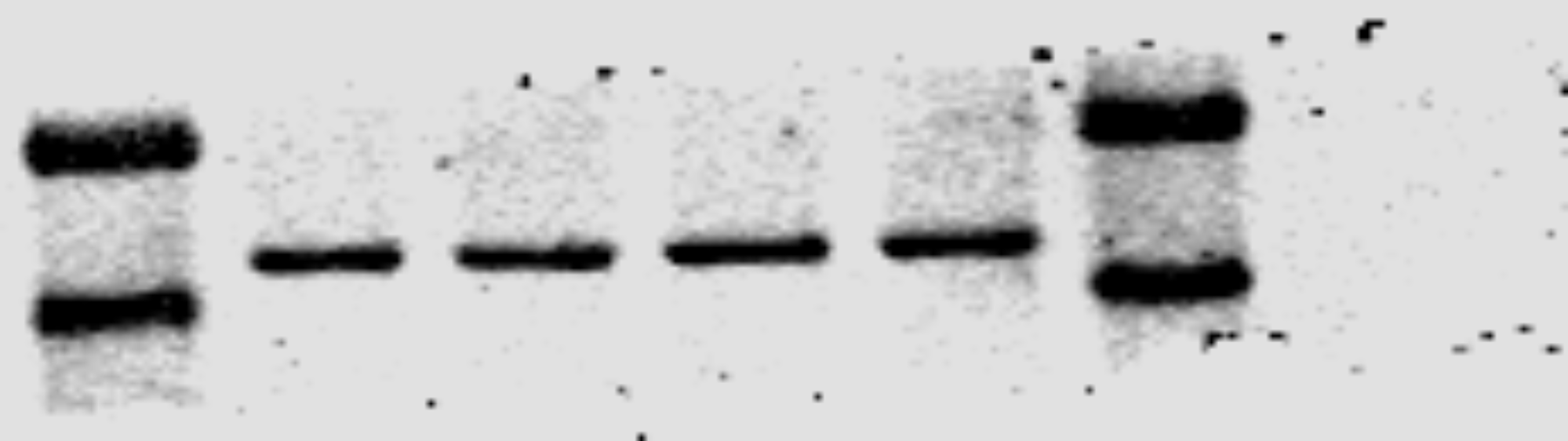

Supplement: Supplementary file 2 — Supplementary Information 2. [file 41598_2023_50476_MOESM2_ESM.zip › protein/1article/3.ferroptosis Figure8/T24/ACTIN.tif]

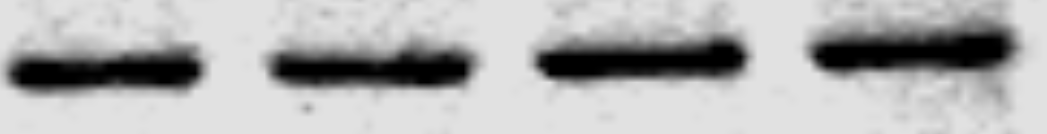

Supplement: Supplementary file 2 — Supplementary Information 2. [file 41598_2023_50476_MOESM2_ESM.zip › protein/1article/3.ferroptosis Figure8/T24/ACTINcut.tif]

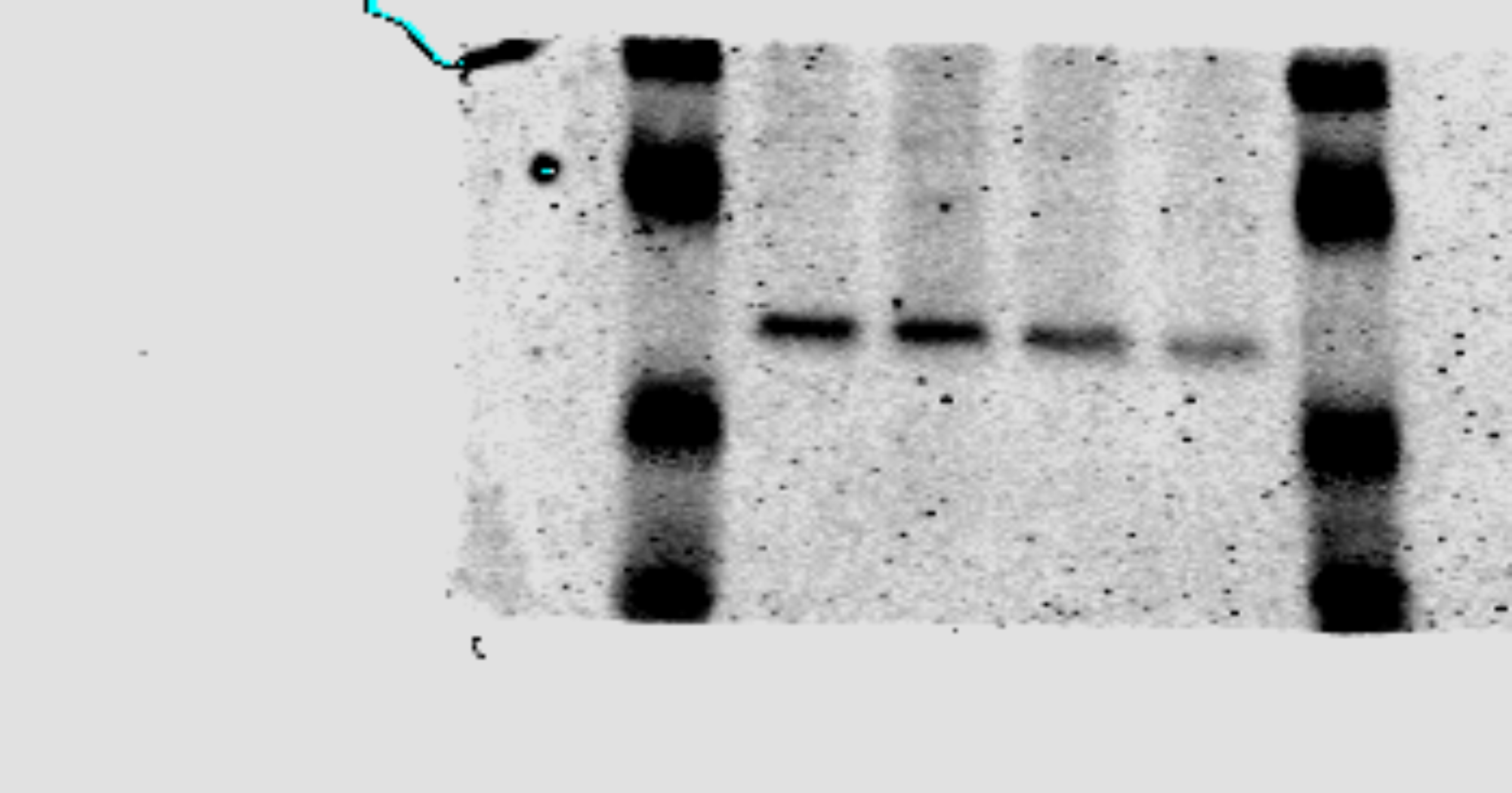

Supplement: Supplementary file 2 — Supplementary Information 2. [file 41598_2023_50476_MOESM2_ESM.zip › protein/1article/3.ferroptosis Figure8/T24/GPX4.tif]

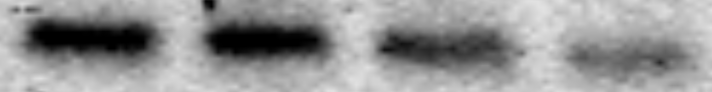

Supplement: Supplementary file 2 — Supplementary Information 2. [file 41598_2023_50476_MOESM2_ESM.zip › protein/1article/3.ferroptosis Figure8/T24/GPX4cut.tif]

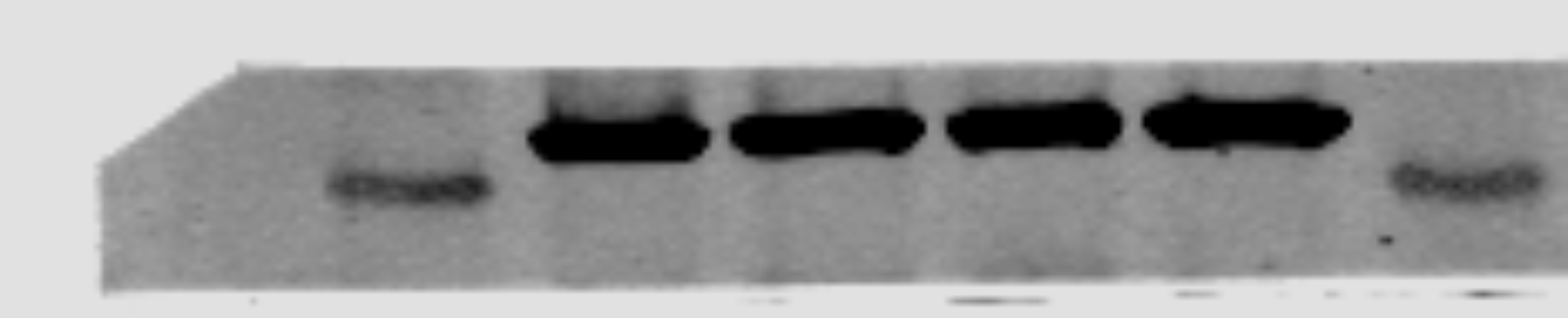

Supplement: Supplementary file 2 — Supplementary Information 2. [file 41598_2023_50476_MOESM2_ESM.zip › protein/1article/4.targets Figure9/5637/ACTIN.tif]

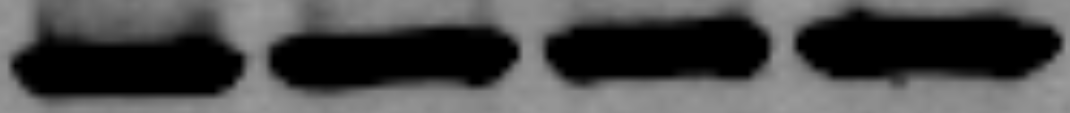

Supplement: Supplementary file 2 — Supplementary Information 2. [file 41598_2023_50476_MOESM2_ESM.zip › protein/1article/4.targets Figure9/5637/ACTINCUT.tif]

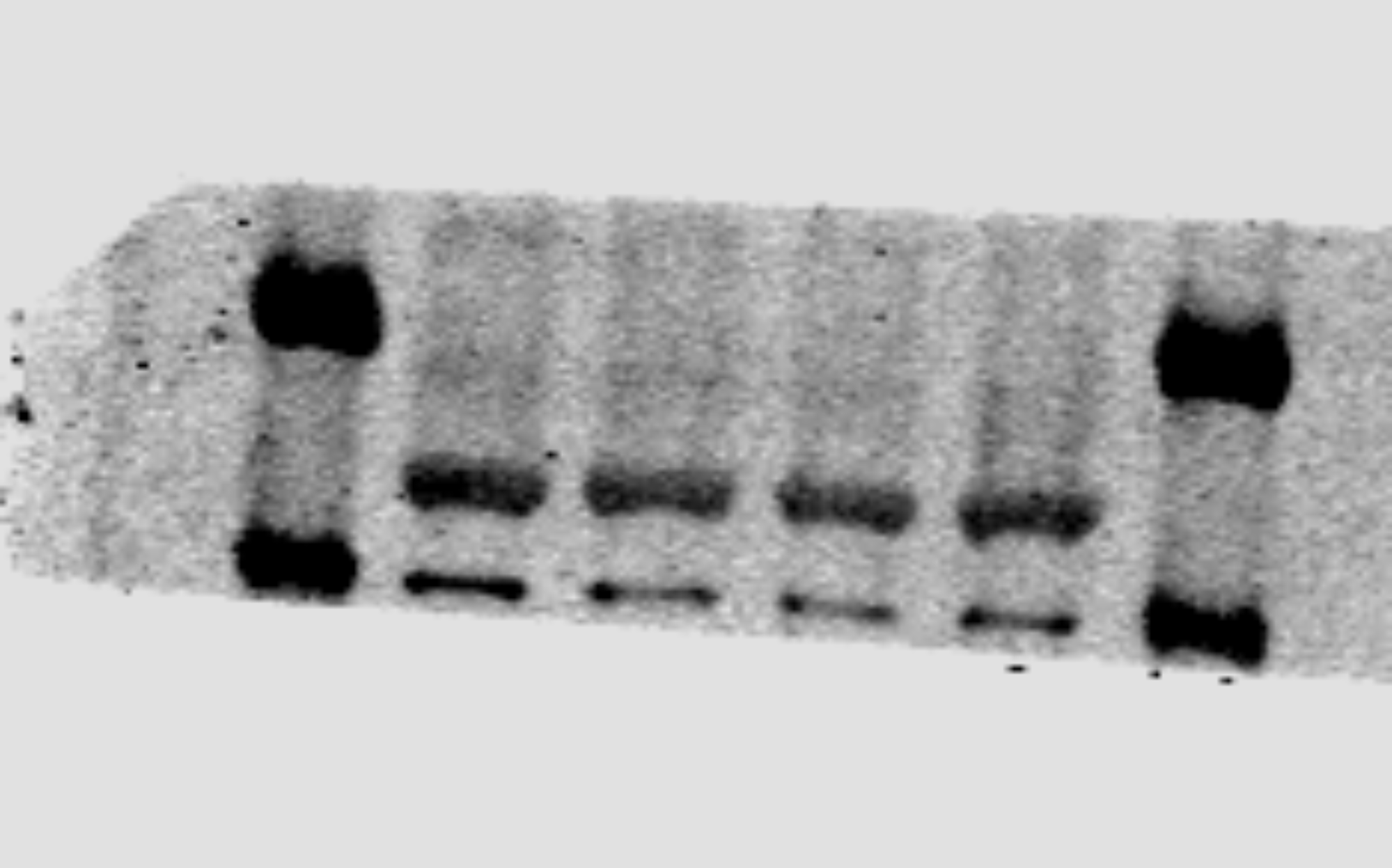

Supplement: Supplementary file 2 — Supplementary Information 2. [file 41598_2023_50476_MOESM2_ESM.zip › protein/1article/4.targets Figure9/5637/MAPK1.tif]

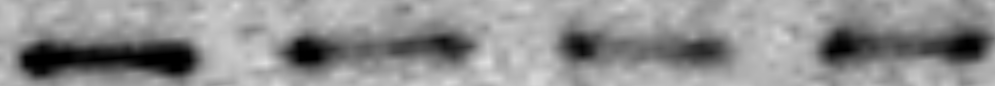

Supplement: Supplementary file 2 — Supplementary Information 2. [file 41598_2023_50476_MOESM2_ESM.zip › protein/1article/4.targets Figure9/5637/MAPK1CUT.tif]

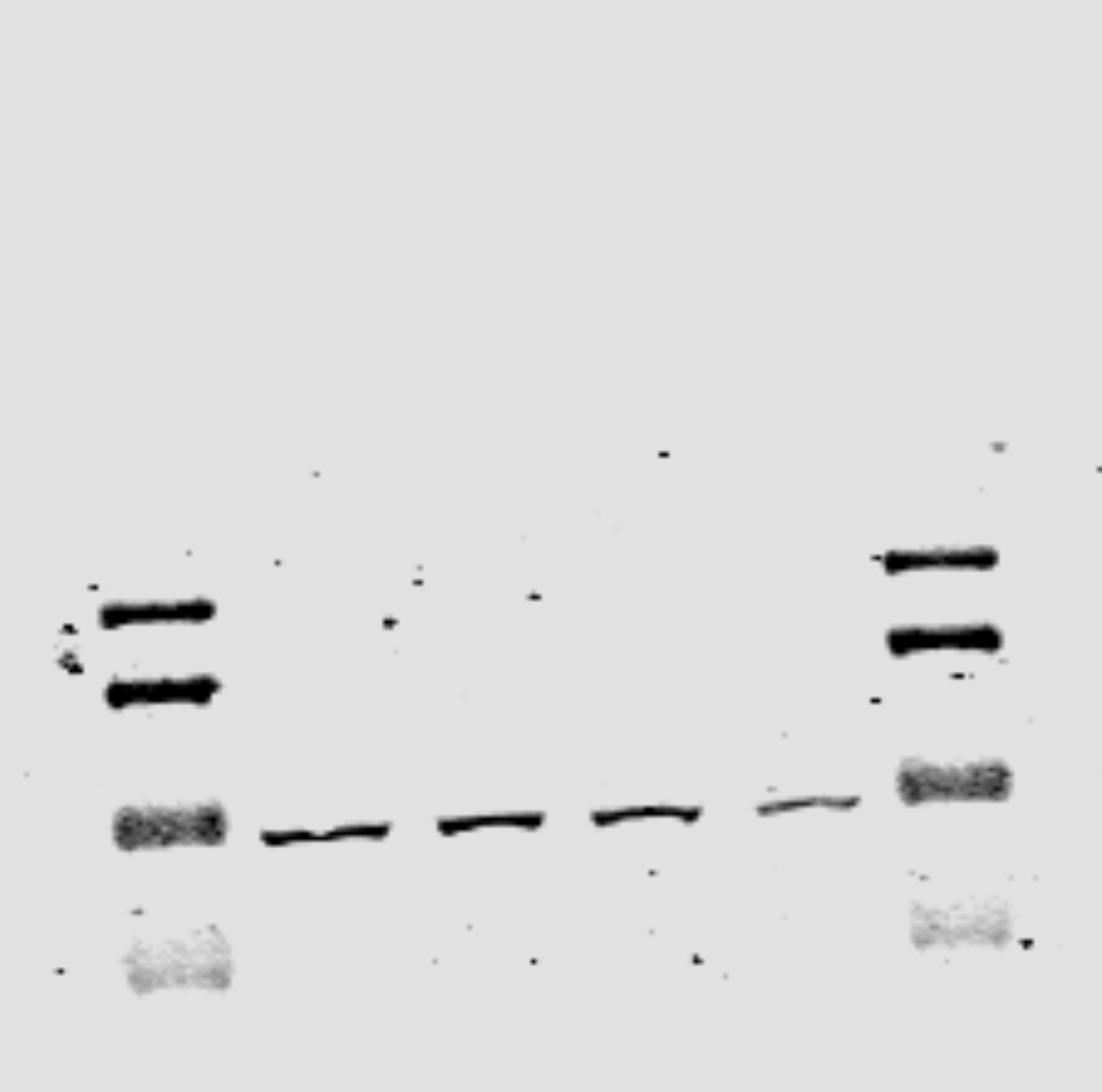

Supplement: Supplementary file 2 — Supplementary Information 2. [file 41598_2023_50476_MOESM2_ESM.zip › protein/1article/4.targets Figure9/5637/P85.tif]

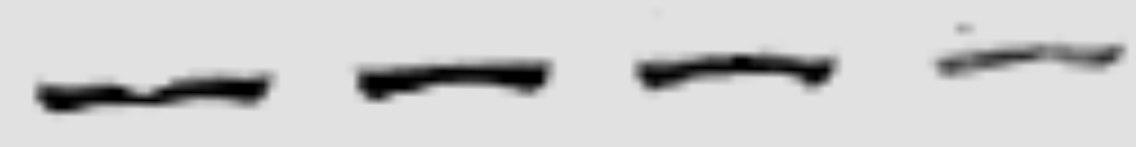

Supplement: Supplementary file 2 — Supplementary Information 2. [file 41598_2023_50476_MOESM2_ESM.zip › protein/1article/4.targets Figure9/5637/P85CUT.tif]

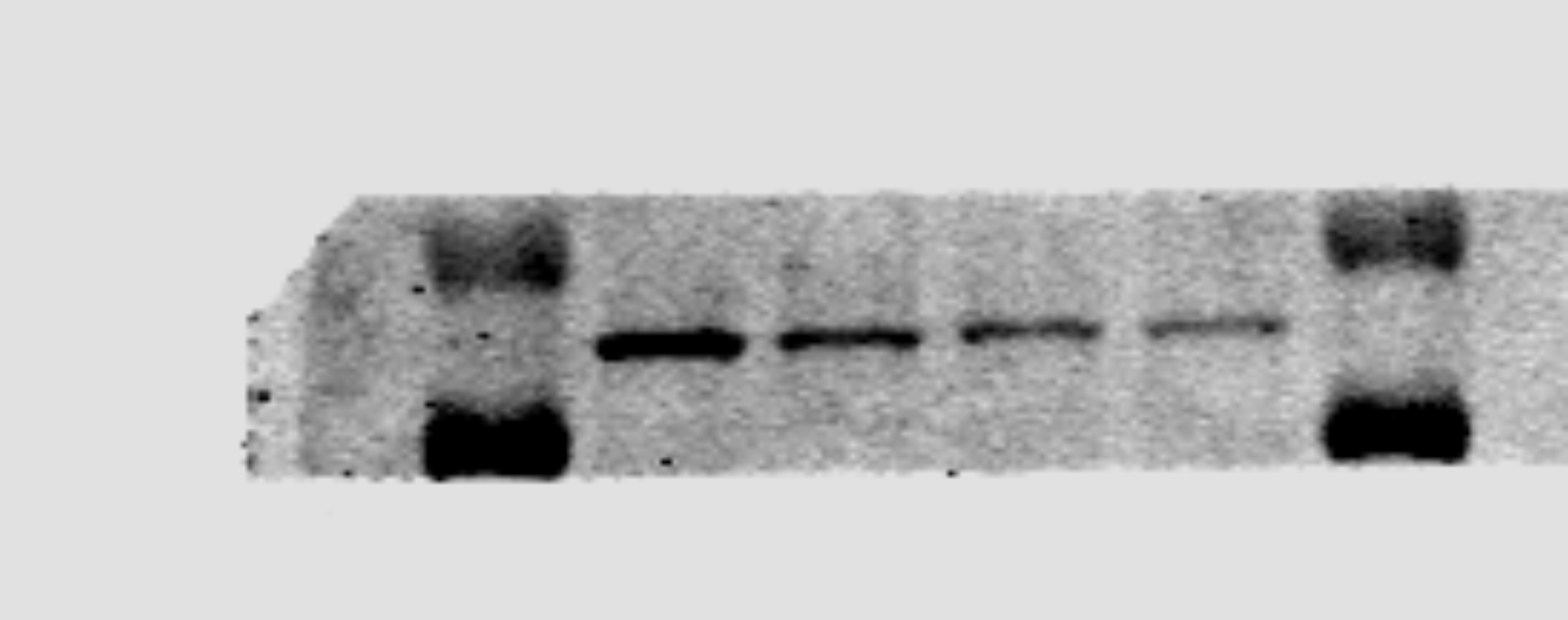

Supplement: Supplementary file 2 — Supplementary Information 2. [file 41598_2023_50476_MOESM2_ESM.zip › protein/1article/4.targets Figure9/5637/SRC.tif]

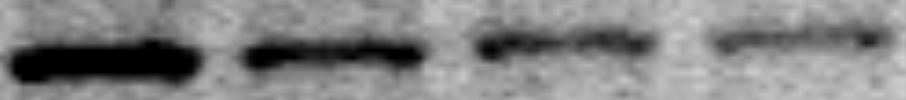

Supplement: Supplementary file 2 — Supplementary Information 2. [file 41598_2023_50476_MOESM2_ESM.zip › protein/1article/4.targets Figure9/5637/SRCCUT.tif]

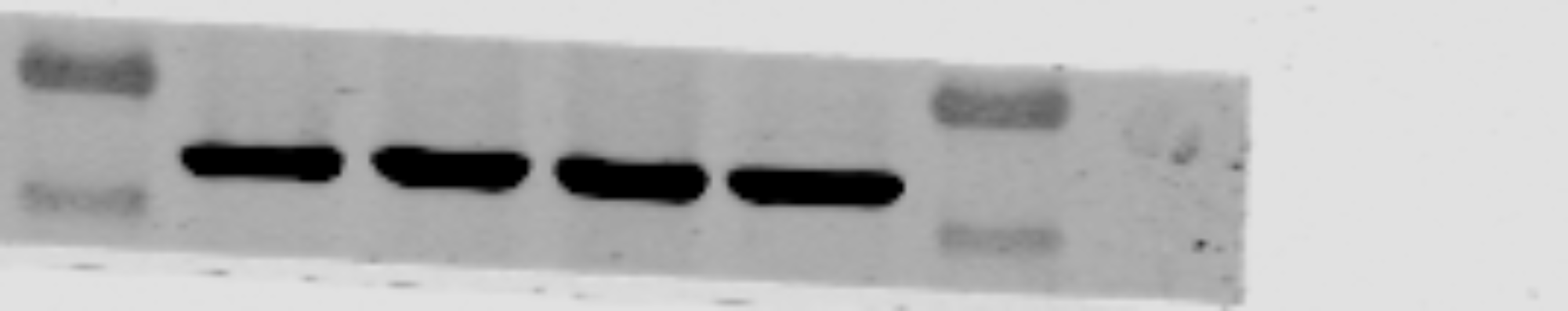

Supplement: Supplementary file 2 — Supplementary Information 2. [file 41598_2023_50476_MOESM2_ESM.zip › protein/1article/4.targets Figure9/T24/ACTIN.tif]

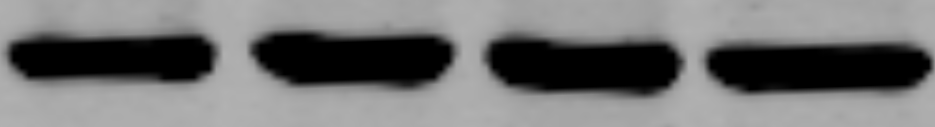

Supplement: Supplementary file 2 — Supplementary Information 2. [file 41598_2023_50476_MOESM2_ESM.zip › protein/1article/4.targets Figure9/T24/ACTINCUT.tif]

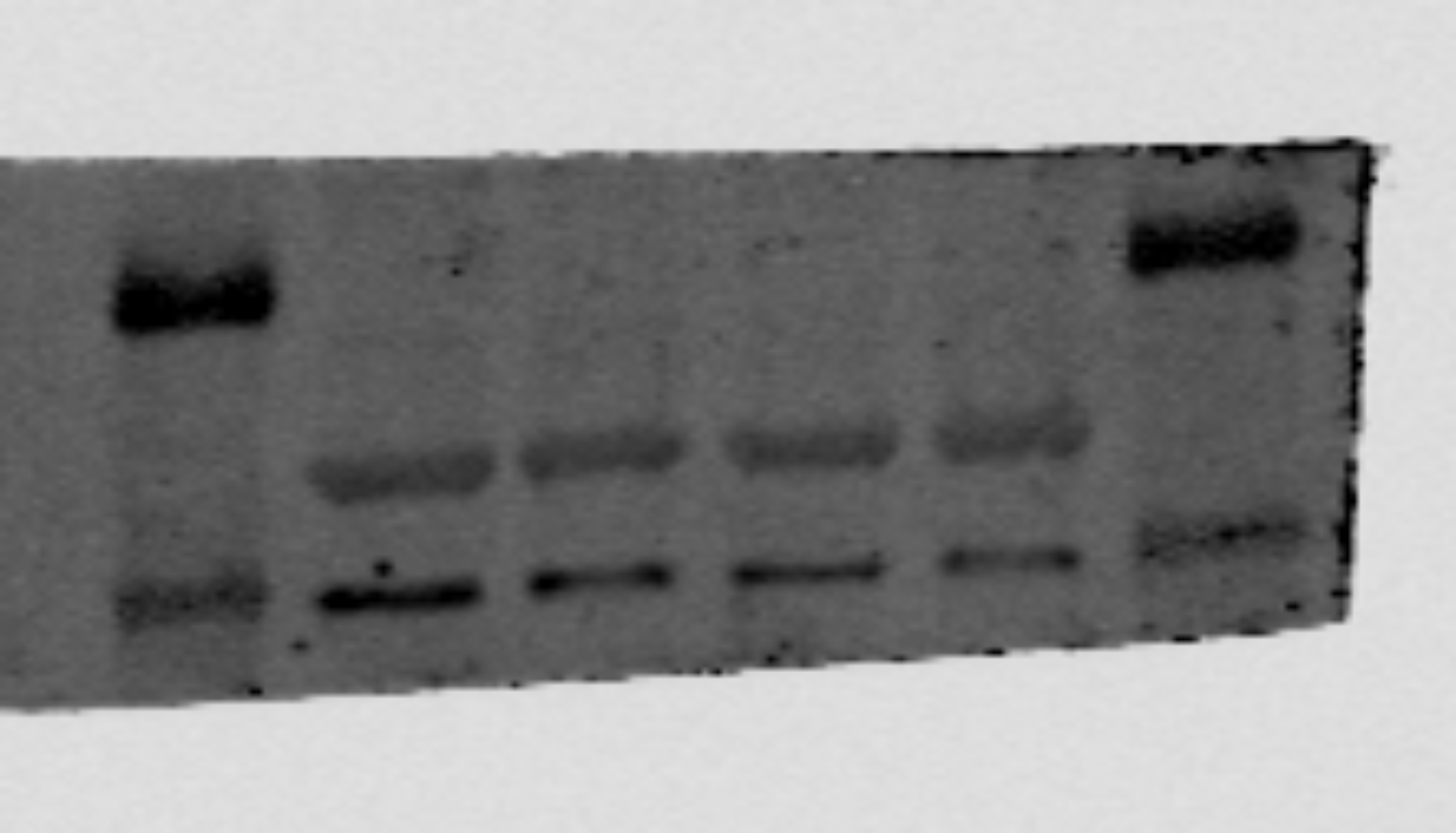

Supplement: Supplementary file 2 — Supplementary Information 2. [file 41598_2023_50476_MOESM2_ESM.zip › protein/1article/4.targets Figure9/T24/MAPK1.tif]

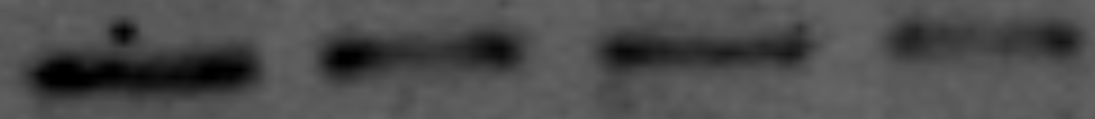

Supplement: Supplementary file 2 — Supplementary Information 2. [file 41598_2023_50476_MOESM2_ESM.zip › protein/1article/4.targets Figure9/T24/MAPK1CUT.tif]

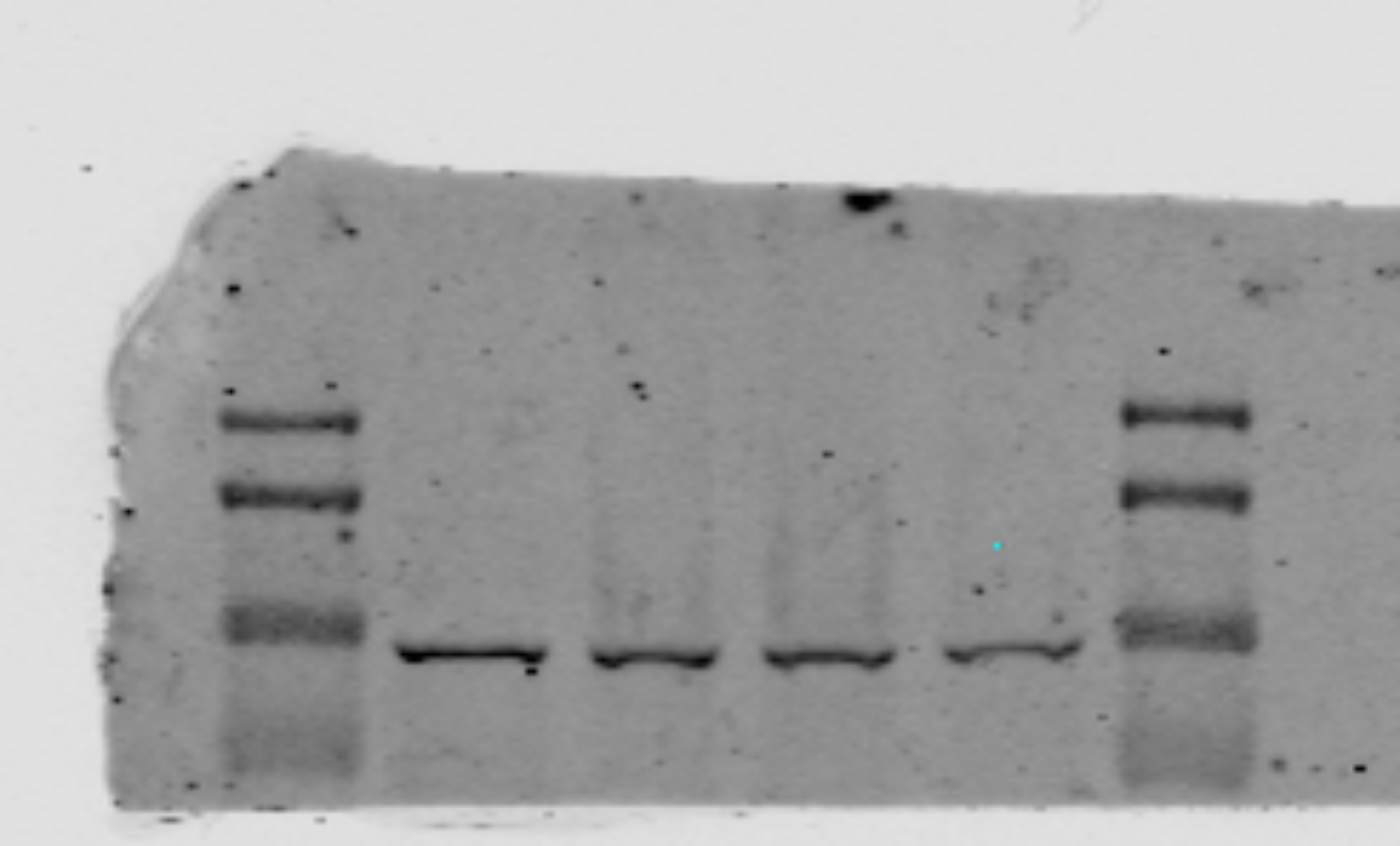

Supplement: Supplementary file 2 — Supplementary Information 2. [file 41598_2023_50476_MOESM2_ESM.zip › protein/1article/4.targets Figure9/T24/P85.tif]

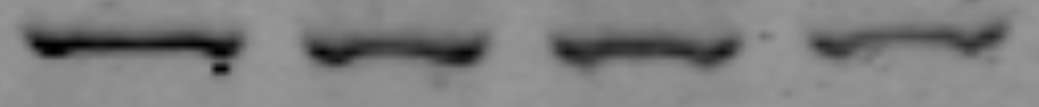

Supplement: Supplementary file 2 — Supplementary Information 2. [file 41598_2023_50476_MOESM2_ESM.zip › protein/1article/4.targets Figure9/T24/P85CUT.tif]

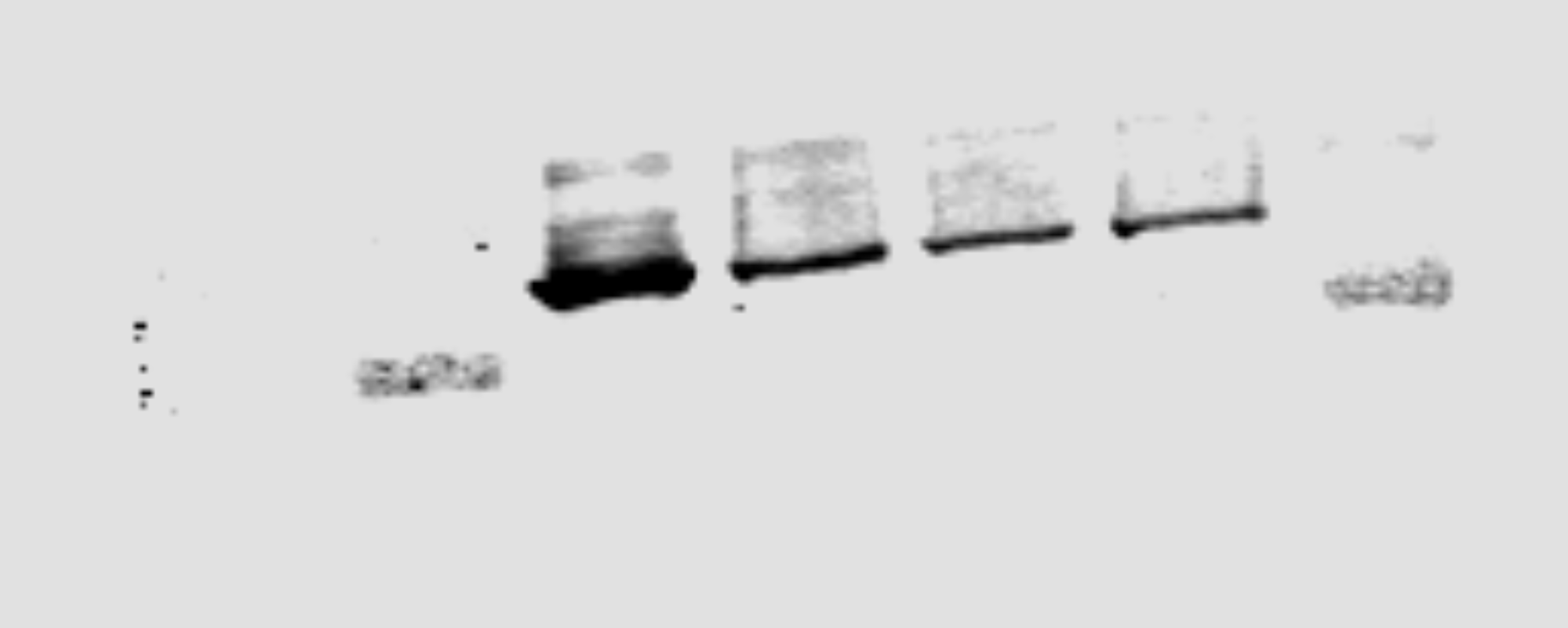

Supplement: Supplementary file 2 — Supplementary Information 2. [file 41598_2023_50476_MOESM2_ESM.zip › protein/1article/4.targets Figure9/T24/SRC.tif]

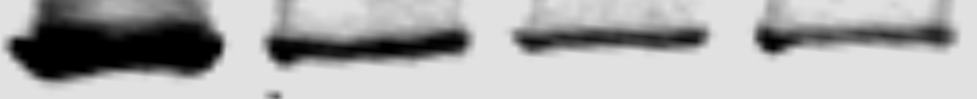

Supplement: Supplementary file 2 — Supplementary Information 2. [file 41598_2023_50476_MOESM2_ESM.zip › protein/1article/4.targets Figure9/T24/SRCCUT.tif]

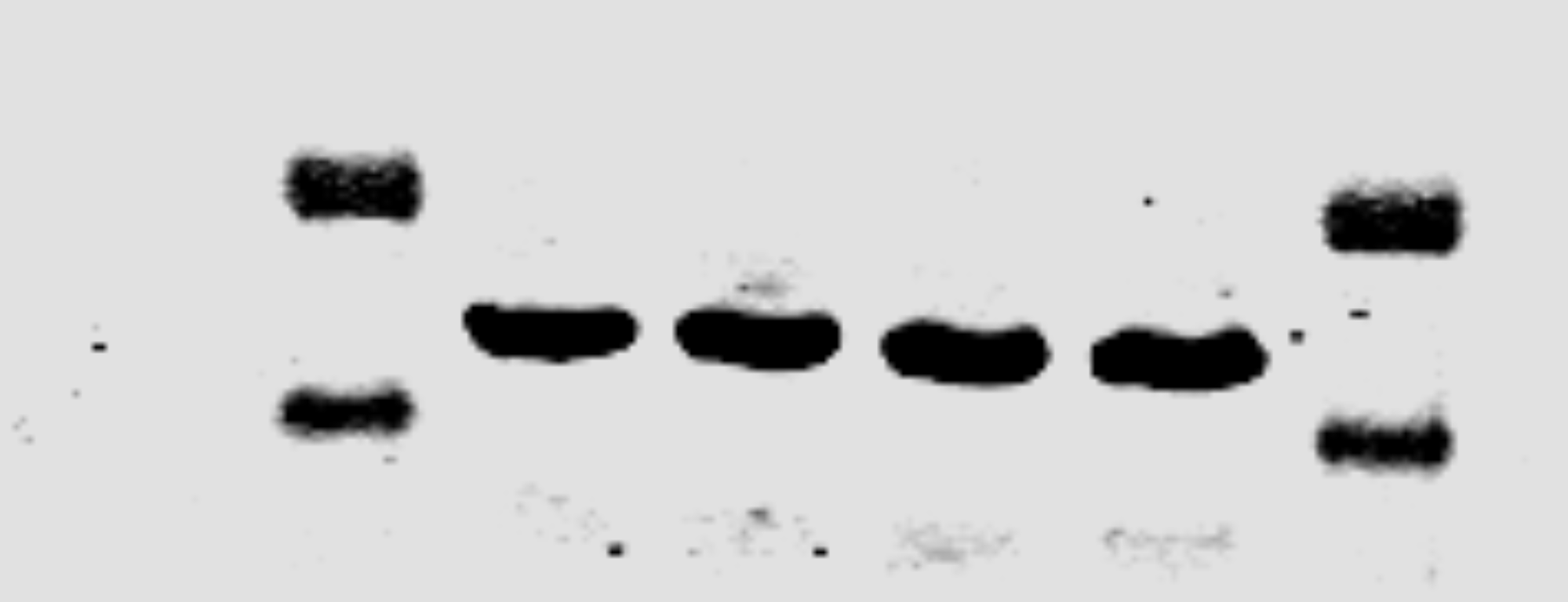

Supplement: Supplementary file 2 — Supplementary Information 2. [file 41598_2023_50476_MOESM2_ESM.zip › protein/1article/5.pathway Figure10/5637/ACTIN.tif]

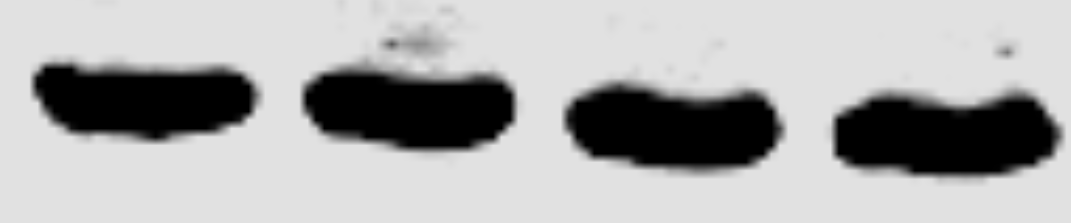

Supplement: Supplementary file 2 — Supplementary Information 2. [file 41598_2023_50476_MOESM2_ESM.zip › protein/1article/5.pathway Figure10/5637/ACTINCUT.tif]

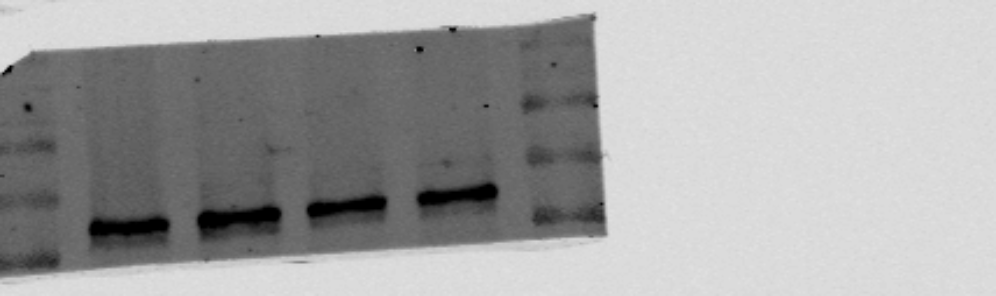

Supplement: Supplementary file 2 — Supplementary Information 2. [file 41598_2023_50476_MOESM2_ESM.zip › protein/1article/5.pathway Figure10/5637/AKT.png]

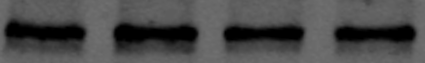

Supplement: Supplementary file 2 — Supplementary Information 2. [file 41598_2023_50476_MOESM2_ESM.zip › protein/1article/5.pathway Figure10/5637/AKTCUT.png]

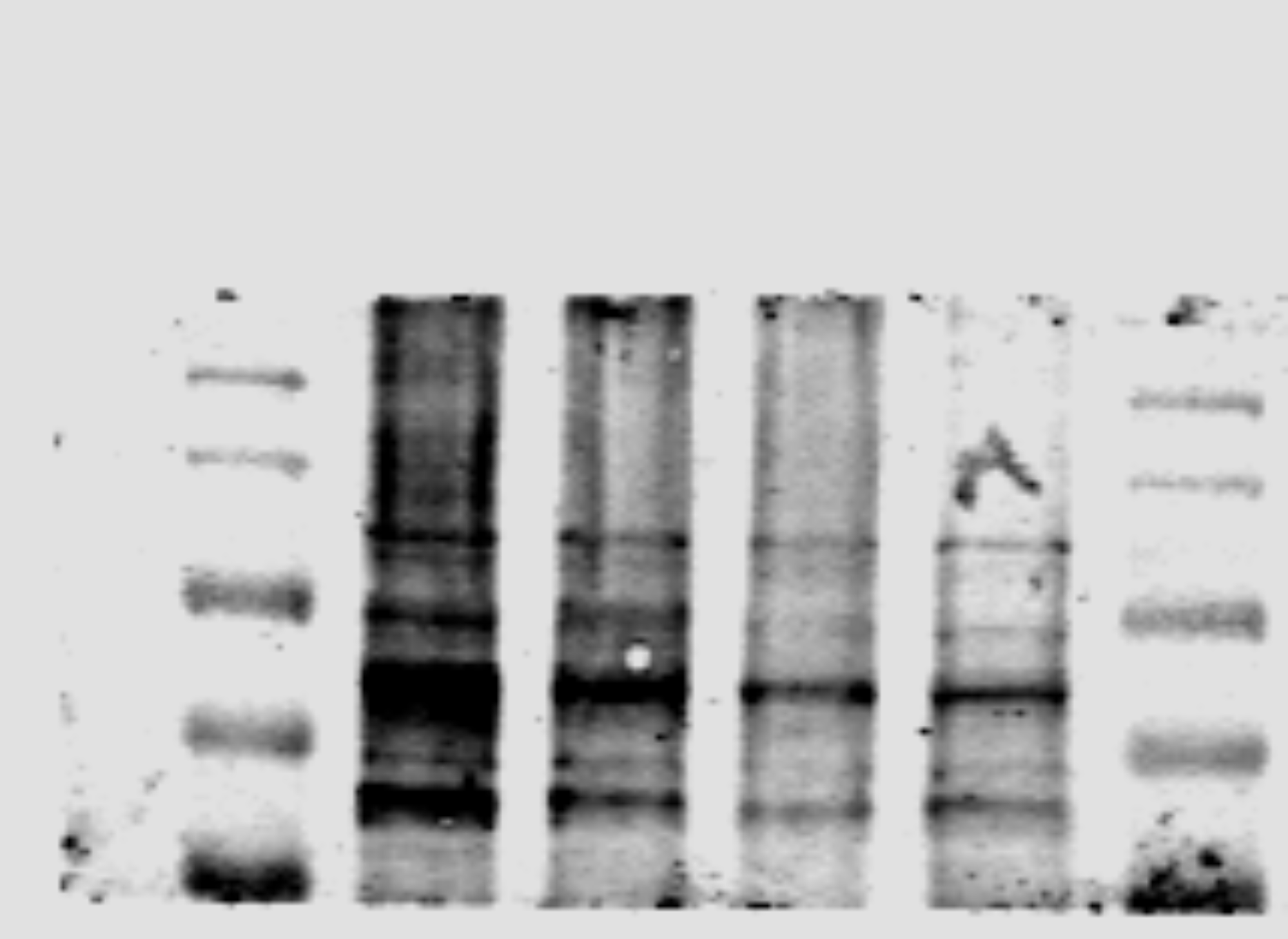

Supplement: Supplementary file 2 — Supplementary Information 2. [file 41598_2023_50476_MOESM2_ESM.zip › protein/1article/5.pathway Figure10/5637/PAKT.tif]

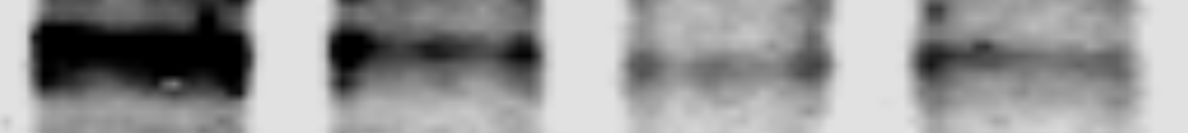

Supplement: Supplementary file 2 — Supplementary Information 2. [file 41598_2023_50476_MOESM2_ESM.zip › protein/1article/5.pathway Figure10/5637/PAKTCUT.tif]

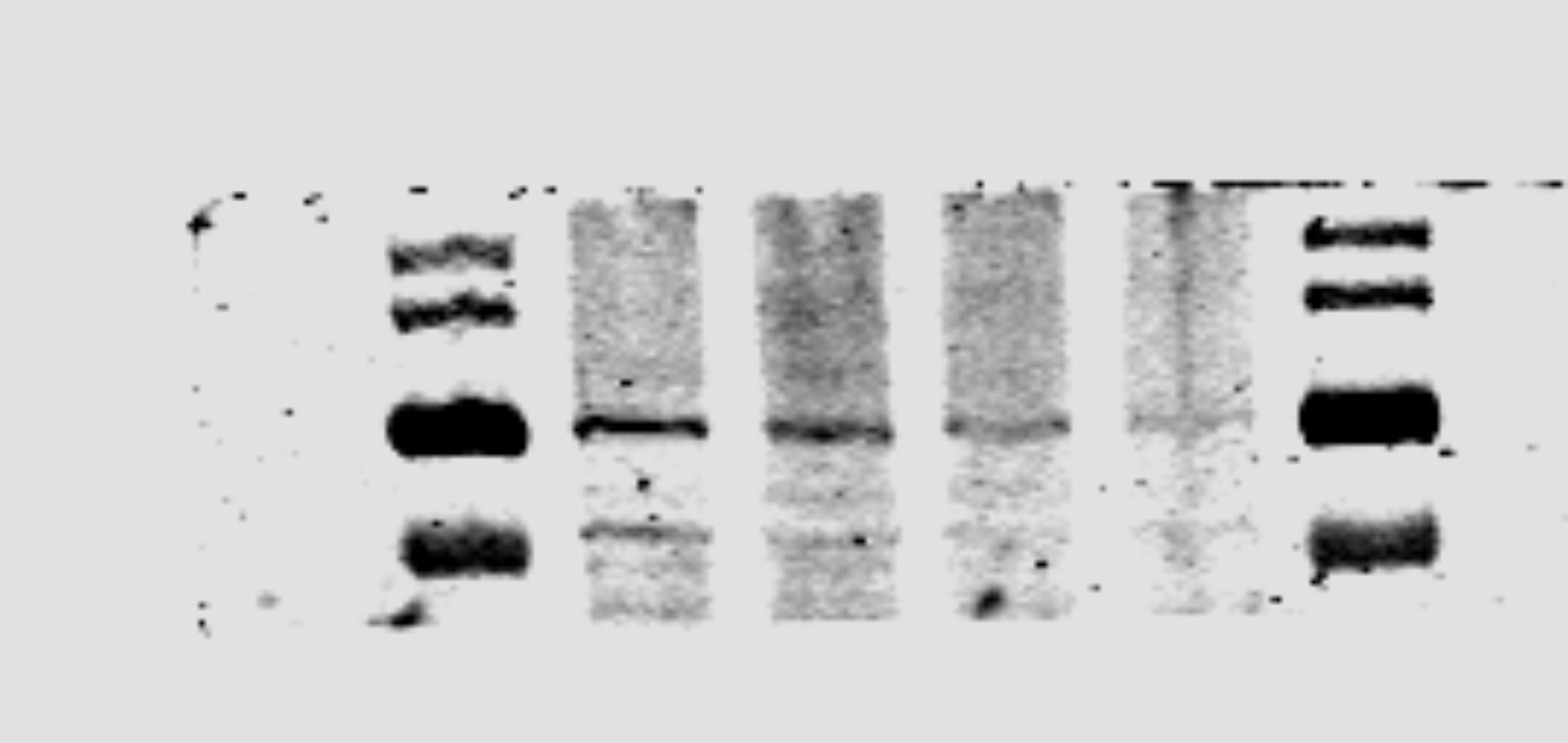

Supplement: Supplementary file 2 — Supplementary Information 2. [file 41598_2023_50476_MOESM2_ESM.zip › protein/1article/5.pathway Figure10/5637/PI3K.tif]

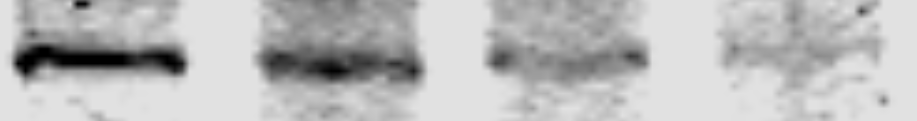

Supplement: Supplementary file 2 — Supplementary Information 2. [file 41598_2023_50476_MOESM2_ESM.zip › protein/1article/5.pathway Figure10/5637/PI3KCUT.tif]

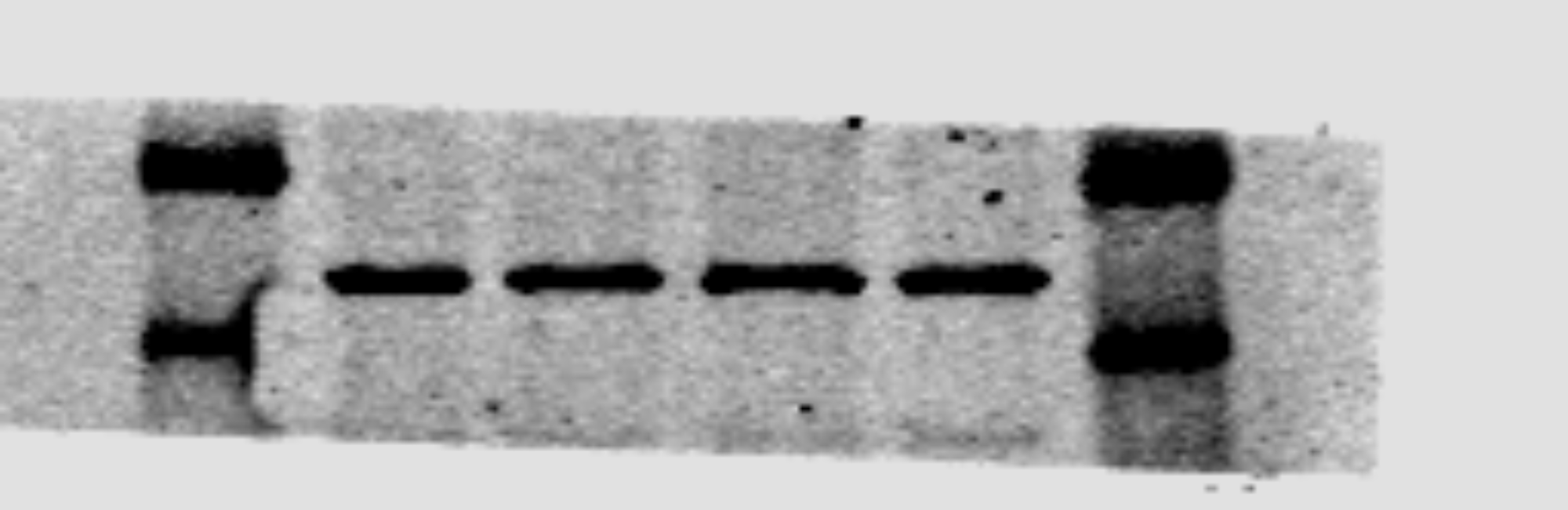

Supplement: Supplementary file 2 — Supplementary Information 2. [file 41598_2023_50476_MOESM2_ESM.zip › protein/1article/5.pathway Figure10/T24/ACTIN.tif]

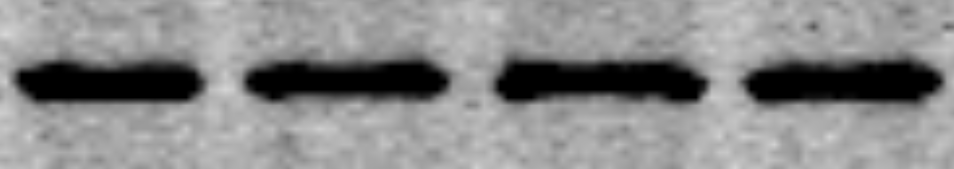

Supplement: Supplementary file 2 — Supplementary Information 2. [file 41598_2023_50476_MOESM2_ESM.zip › protein/1article/5.pathway Figure10/T24/ACTINCUT.tif]

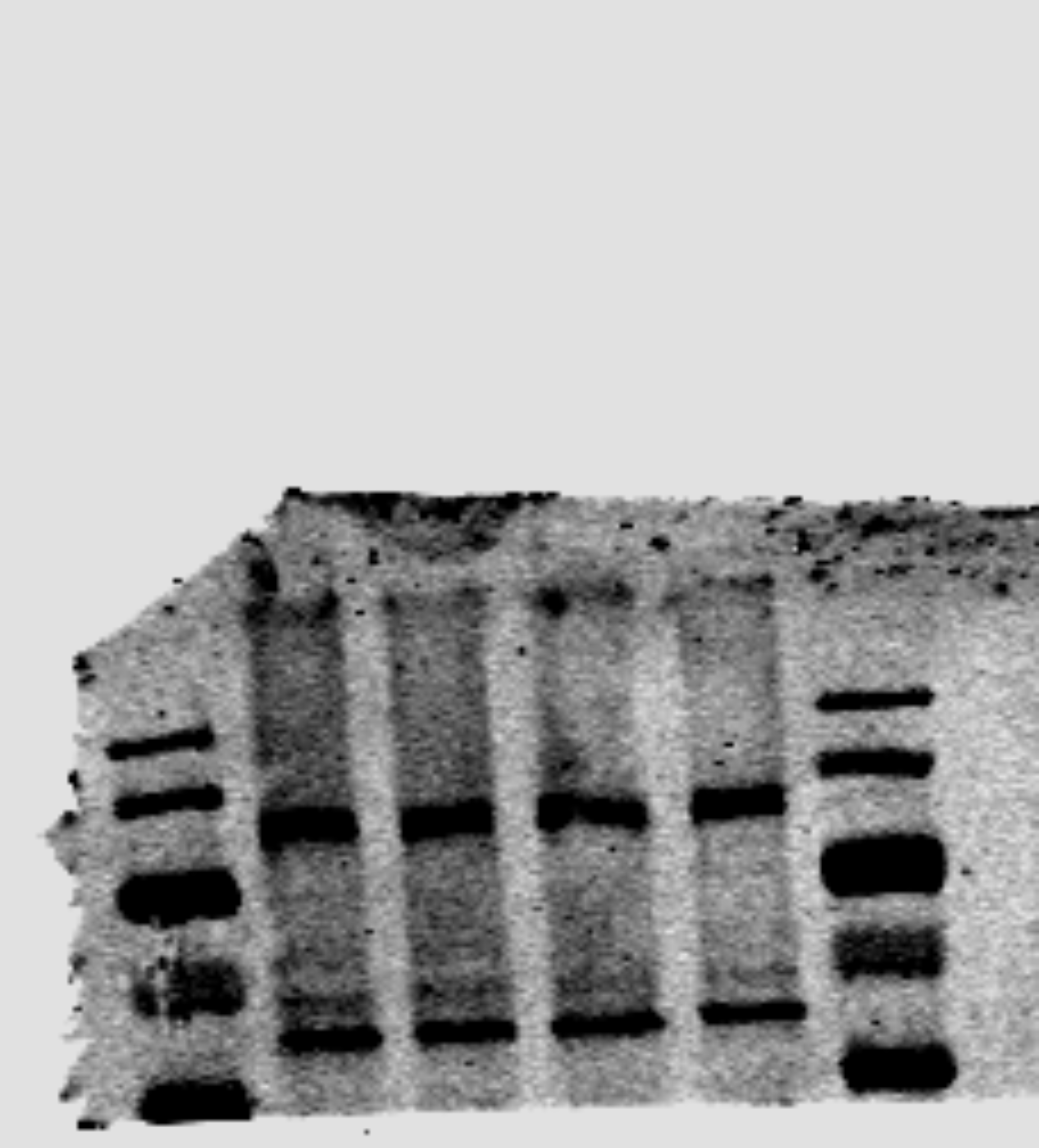

Supplement: Supplementary file 2 — Supplementary Information 2. [file 41598_2023_50476_MOESM2_ESM.zip › protein/1article/5.pathway Figure10/T24/AKT.tif]

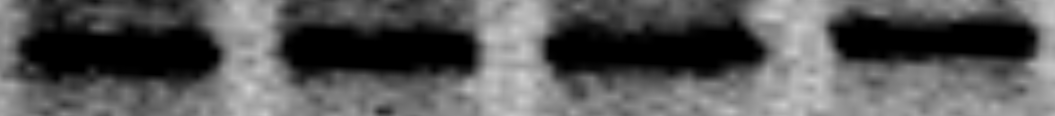

Supplement: Supplementary file 2 — Supplementary Information 2. [file 41598_2023_50476_MOESM2_ESM.zip › protein/1article/5.pathway Figure10/T24/AKTCUT.tif]

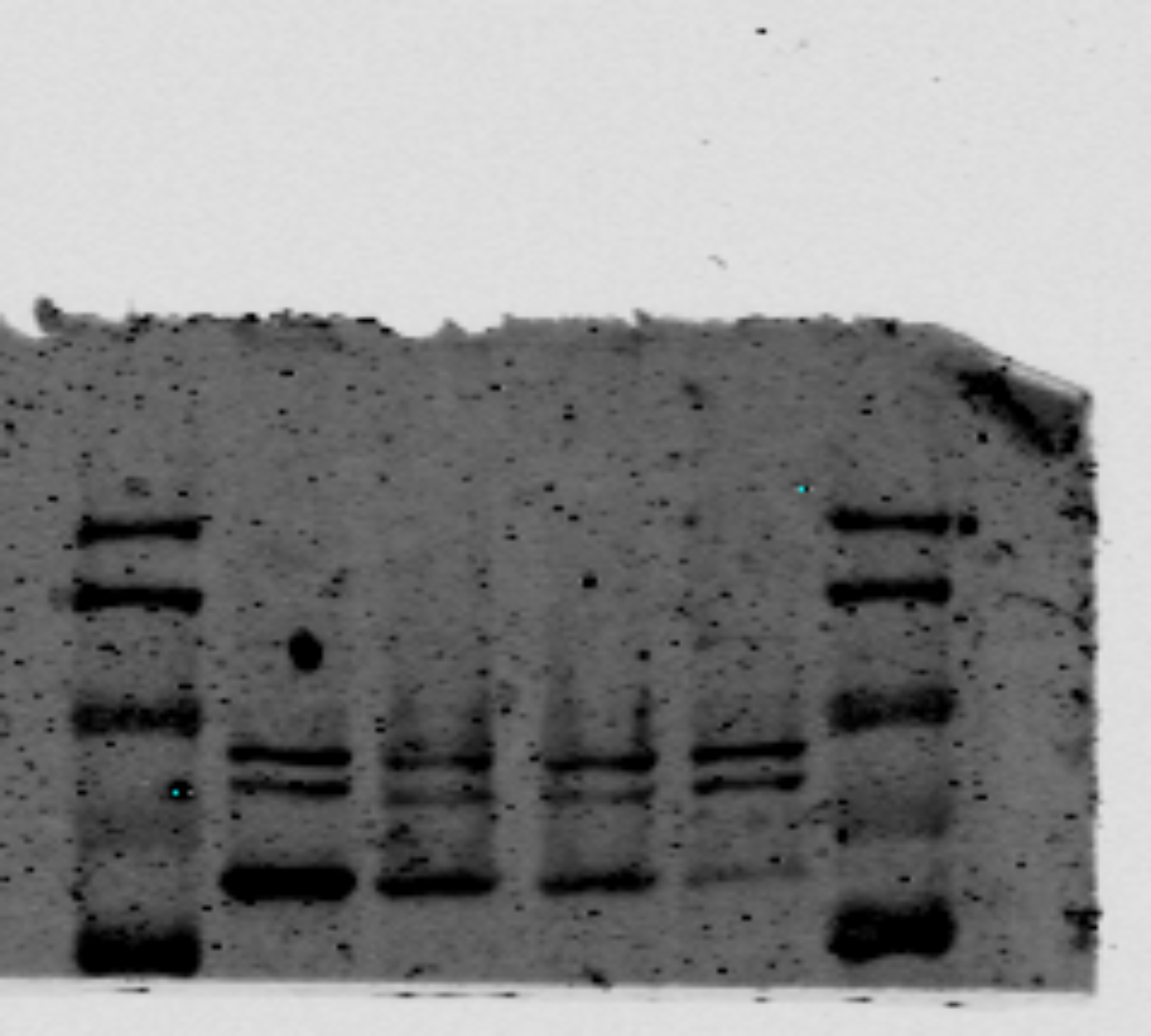

Supplement: Supplementary file 2 — Supplementary Information 2. [file 41598_2023_50476_MOESM2_ESM.zip › protein/1article/5.pathway Figure10/T24/PAKT.tif]

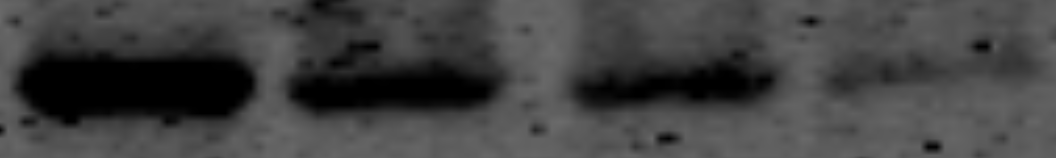

Supplement: Supplementary file 2 — Supplementary Information 2. [file 41598_2023_50476_MOESM2_ESM.zip › protein/1article/5.pathway Figure10/T24/PAKTCUT.tif]

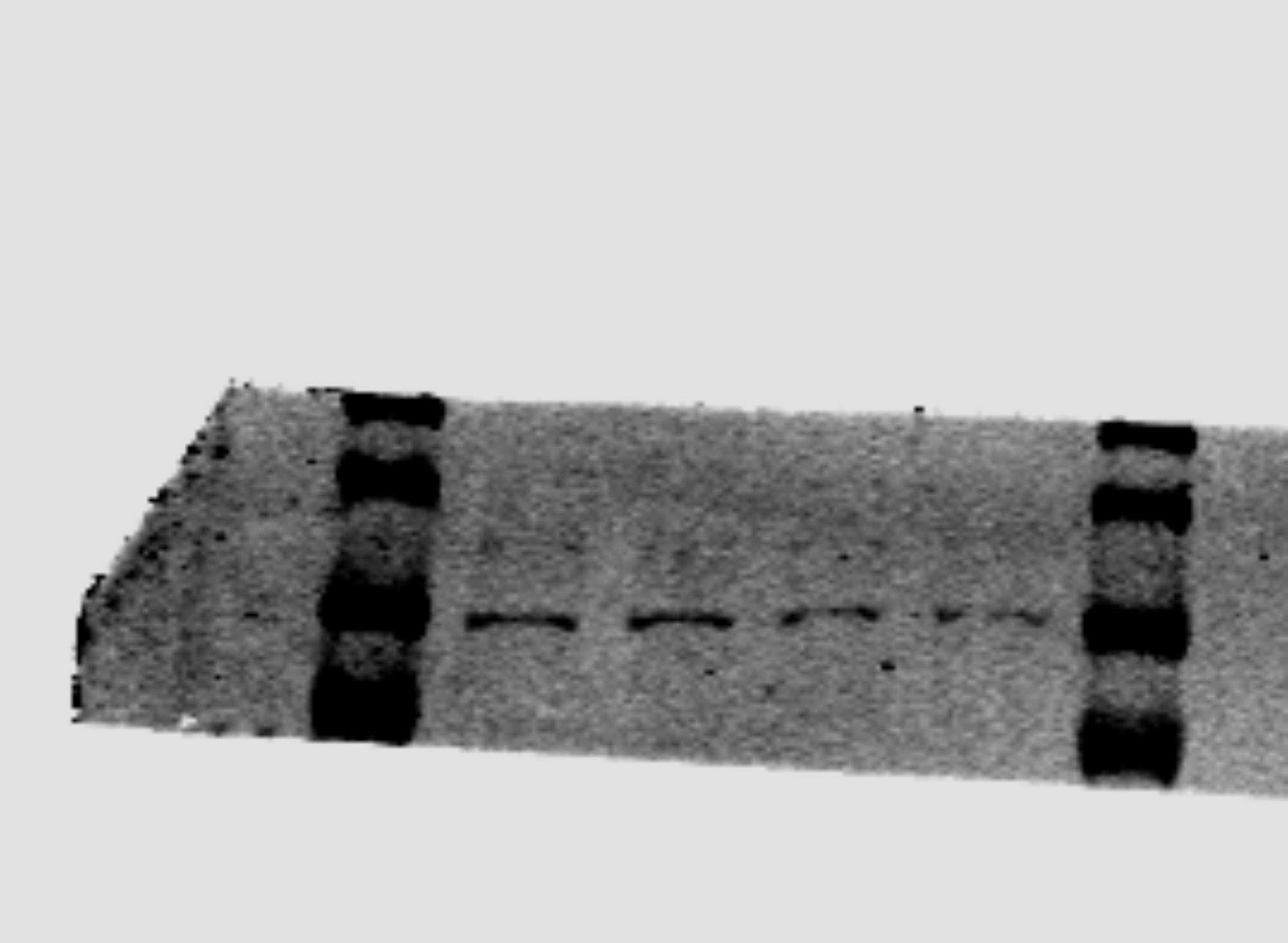

Supplement: Supplementary file 2 — Supplementary Information 2. [file 41598_2023_50476_MOESM2_ESM.zip › protein/1article/5.pathway Figure10/T24/PI3K.tif]

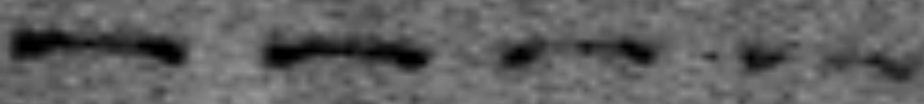

Supplement: Supplementary file 2 — Supplementary Information 2. [file 41598_2023_50476_MOESM2_ESM.zip › protein/1article/5.pathway Figure10/T24/PI3KCUT.tif]

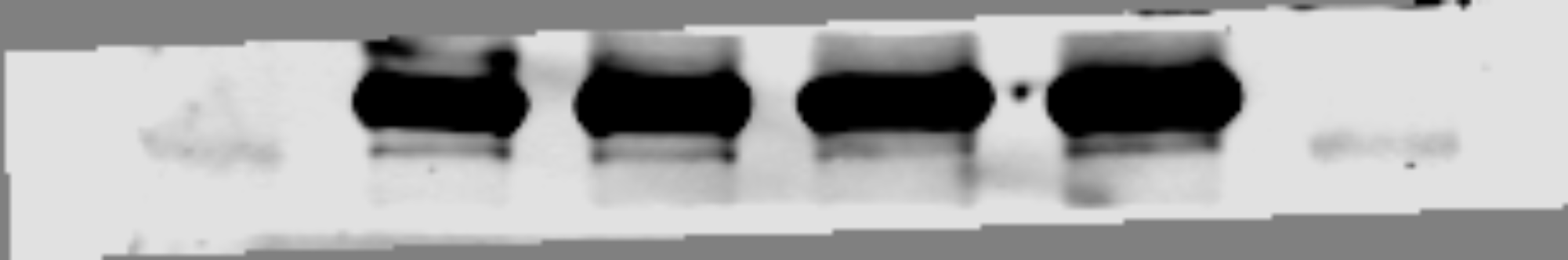

Supplement: Supplementary file 2 — Supplementary Information 2. [file 41598_2023_50476_MOESM2_ESM.zip › protein/2 repeat/1.magration/5637/ACTIN.tif]

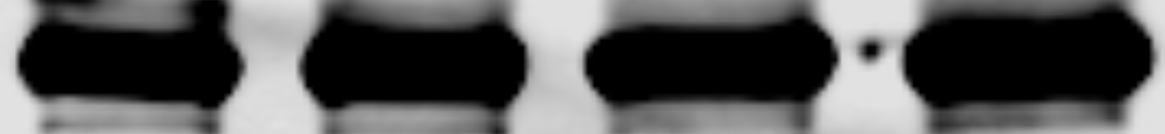

Supplement: Supplementary file 2 — Supplementary Information 2. [file 41598_2023_50476_MOESM2_ESM.zip › protein/2 repeat/1.magration/5637/ACTINcut.tif]

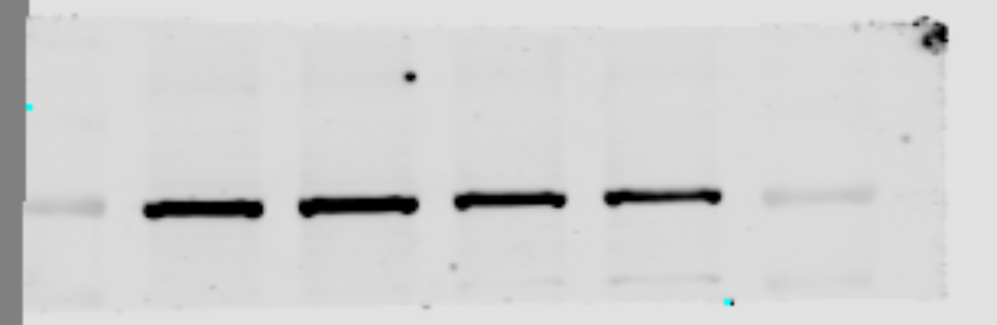

Supplement: Supplementary file 2 — Supplementary Information 2. [file 41598_2023_50476_MOESM2_ESM.zip › protein/2 repeat/1.magration/5637/MMP2.png]

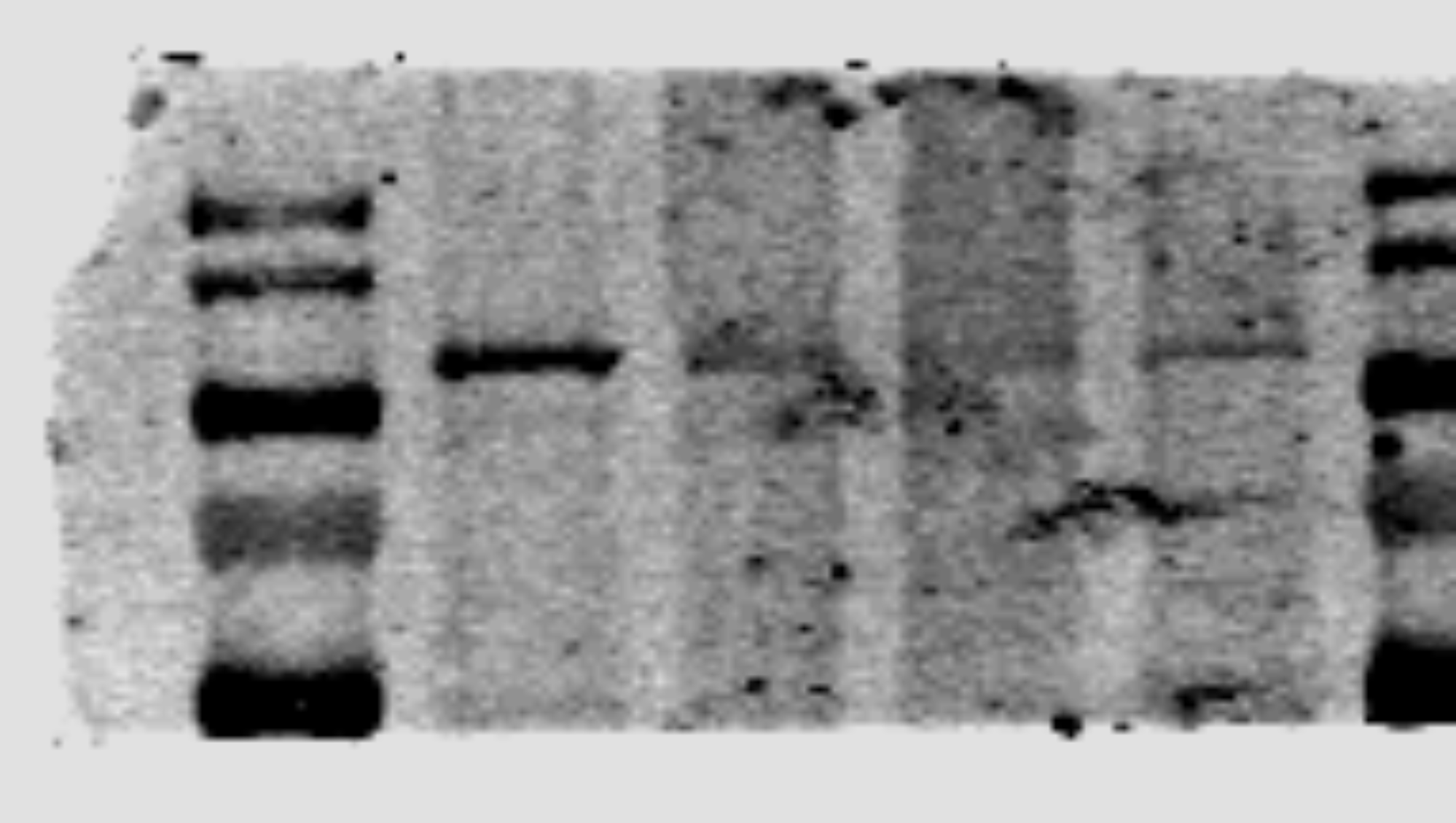

Supplement: Supplementary file 2 — Supplementary Information 2. [file 41598_2023_50476_MOESM2_ESM.zip › protein/2 repeat/1.magration/5637/MMP9.tif]

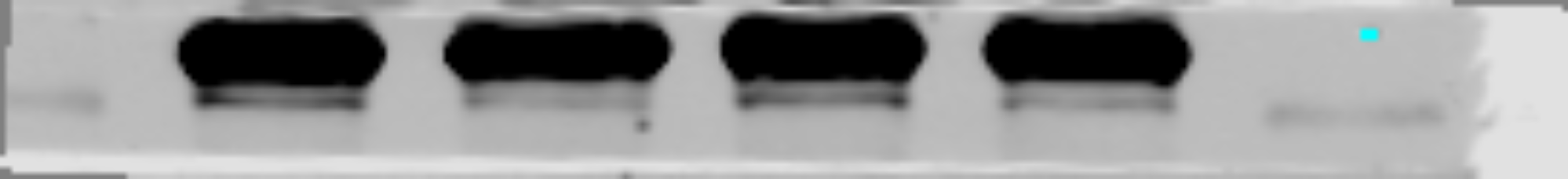

Supplement: Supplementary file 2 — Supplementary Information 2. [file 41598_2023_50476_MOESM2_ESM.zip › protein/2 repeat/1.magration/T24/ACTIN.tif]

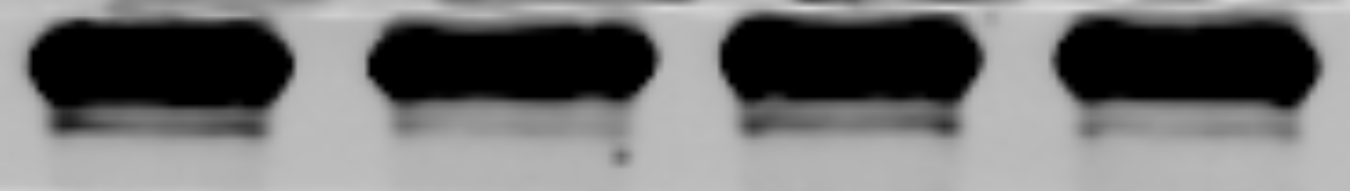

Supplement: Supplementary file 2 — Supplementary Information 2. [file 41598_2023_50476_MOESM2_ESM.zip › protein/2 repeat/1.magration/T24/ACTINCut.tif]

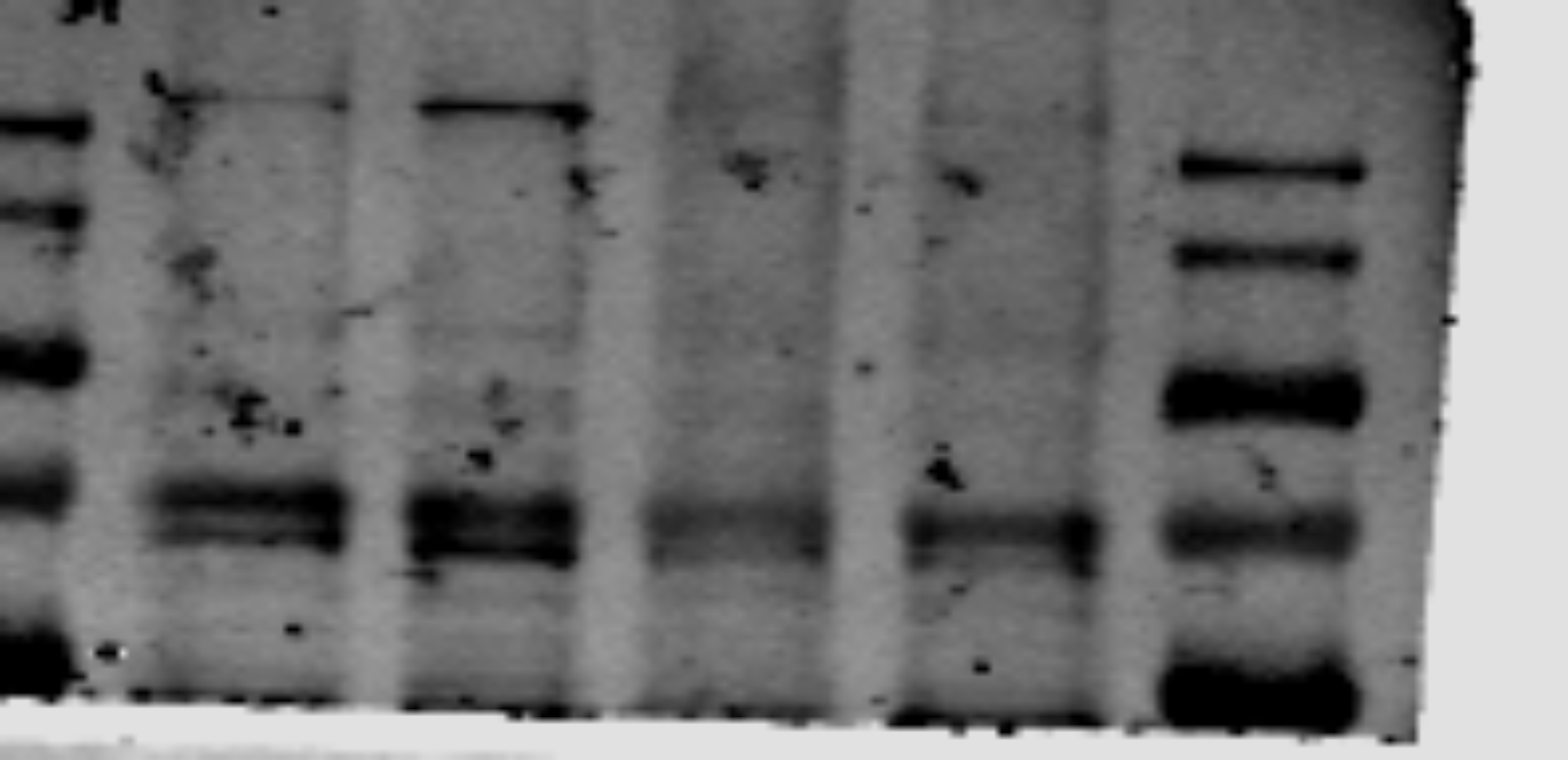

Supplement: Supplementary file 2 — Supplementary Information 2. [file 41598_2023_50476_MOESM2_ESM.zip › protein/2 repeat/1.magration/T24/MMP2.tif]

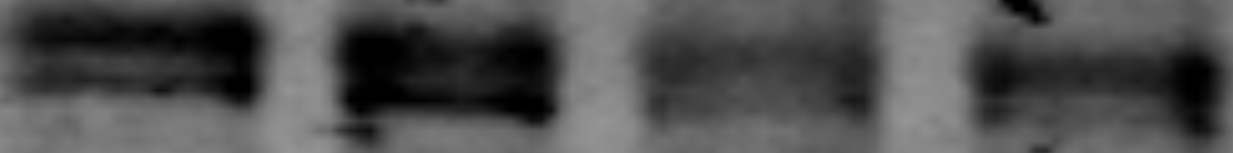

Supplement: Supplementary file 2 — Supplementary Information 2. [file 41598_2023_50476_MOESM2_ESM.zip › protein/2 repeat/1.magration/T24/MMP2JIE.tif]

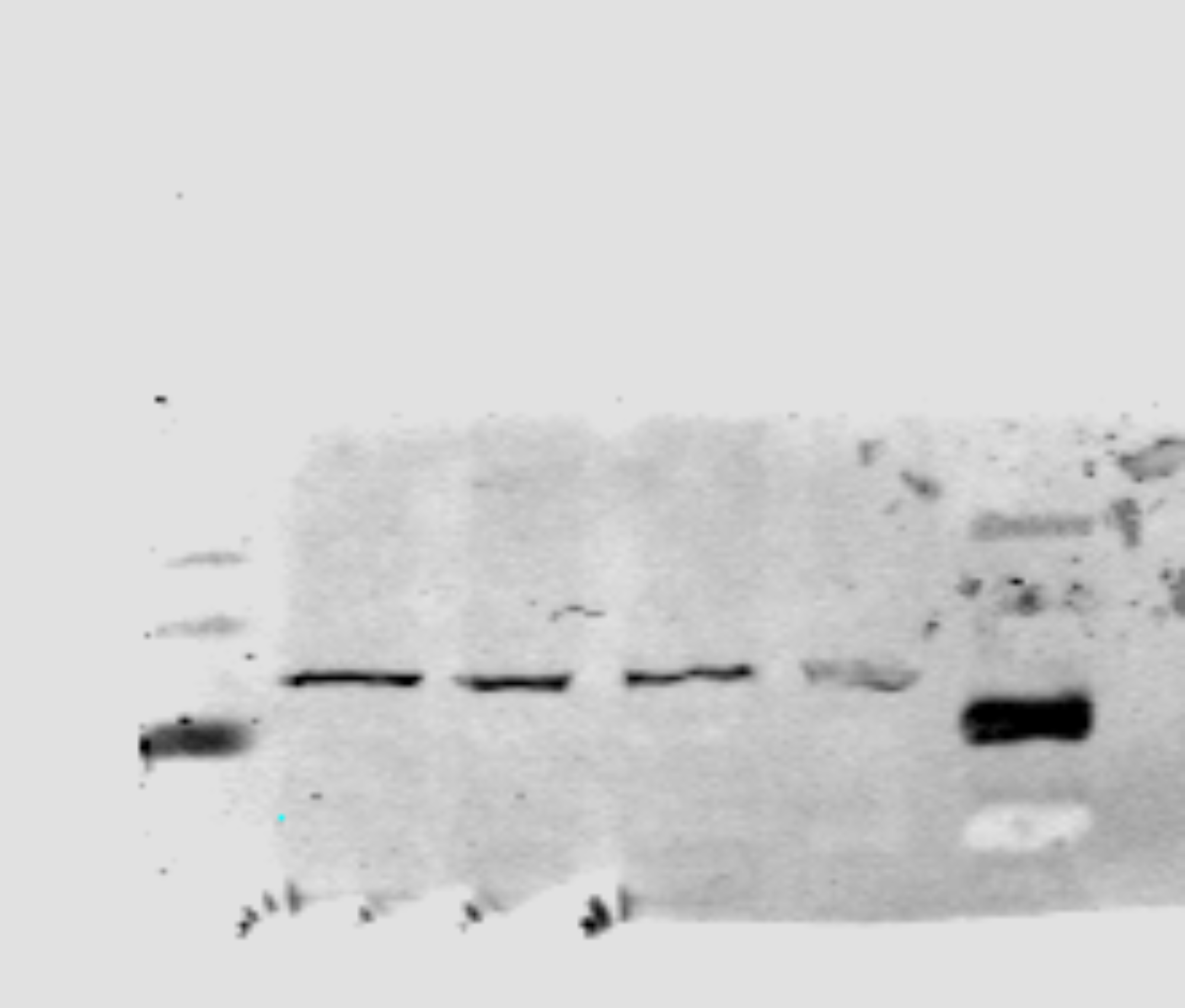

Supplement: Supplementary file 2 — Supplementary Information 2. [file 41598_2023_50476_MOESM2_ESM.zip › protein/2 repeat/1.magration/T24/MMP9.tif]

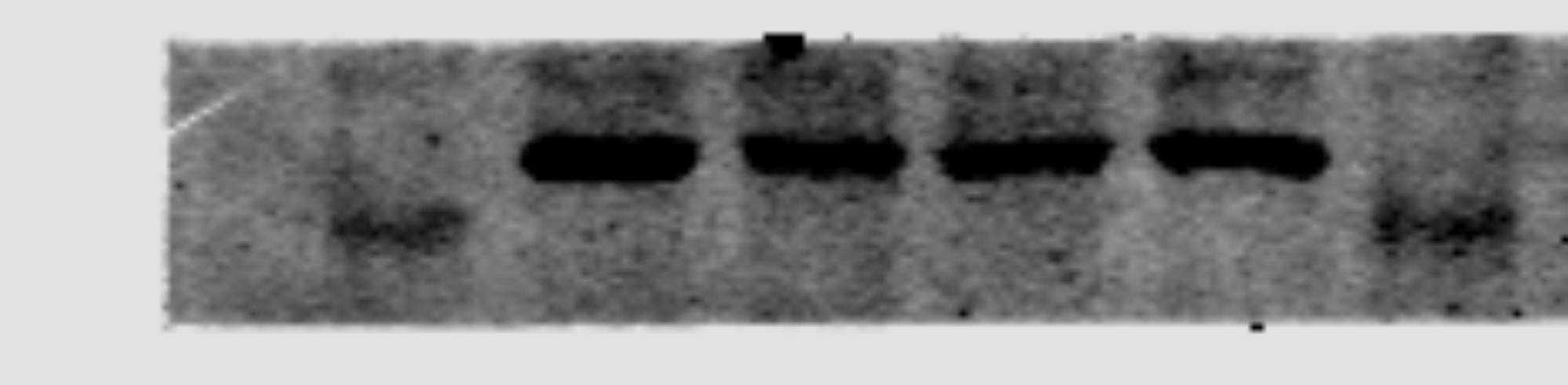

Supplement: Supplementary file 2 — Supplementary Information 2. [file 41598_2023_50476_MOESM2_ESM.zip › protein/2 repeat/2.apoptosis/5637/ACTIN.tif]

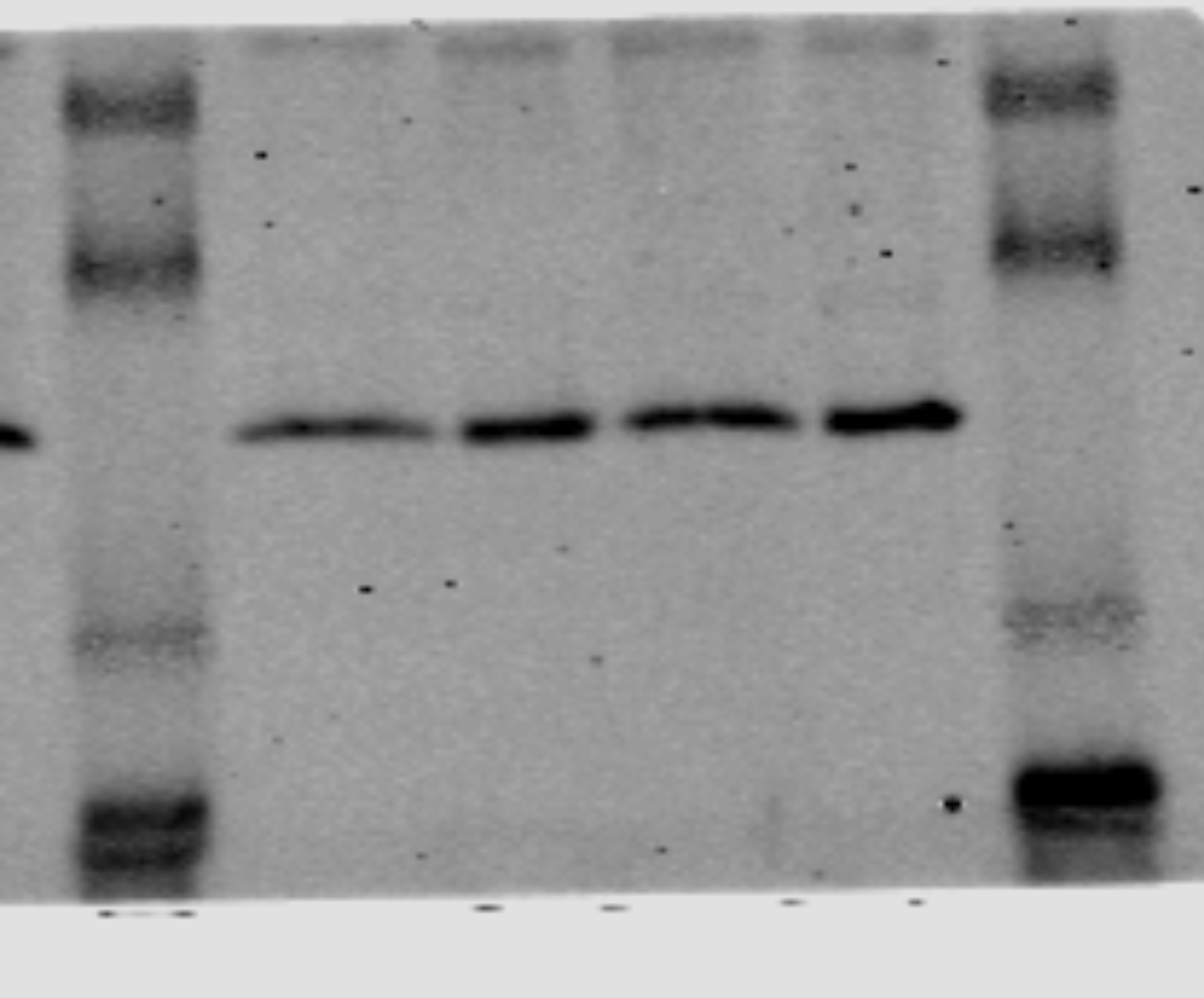

Supplement: Supplementary file 2 — Supplementary Information 2. [file 41598_2023_50476_MOESM2_ESM.zip › protein/2 repeat/2.apoptosis/5637/BAX.tif]

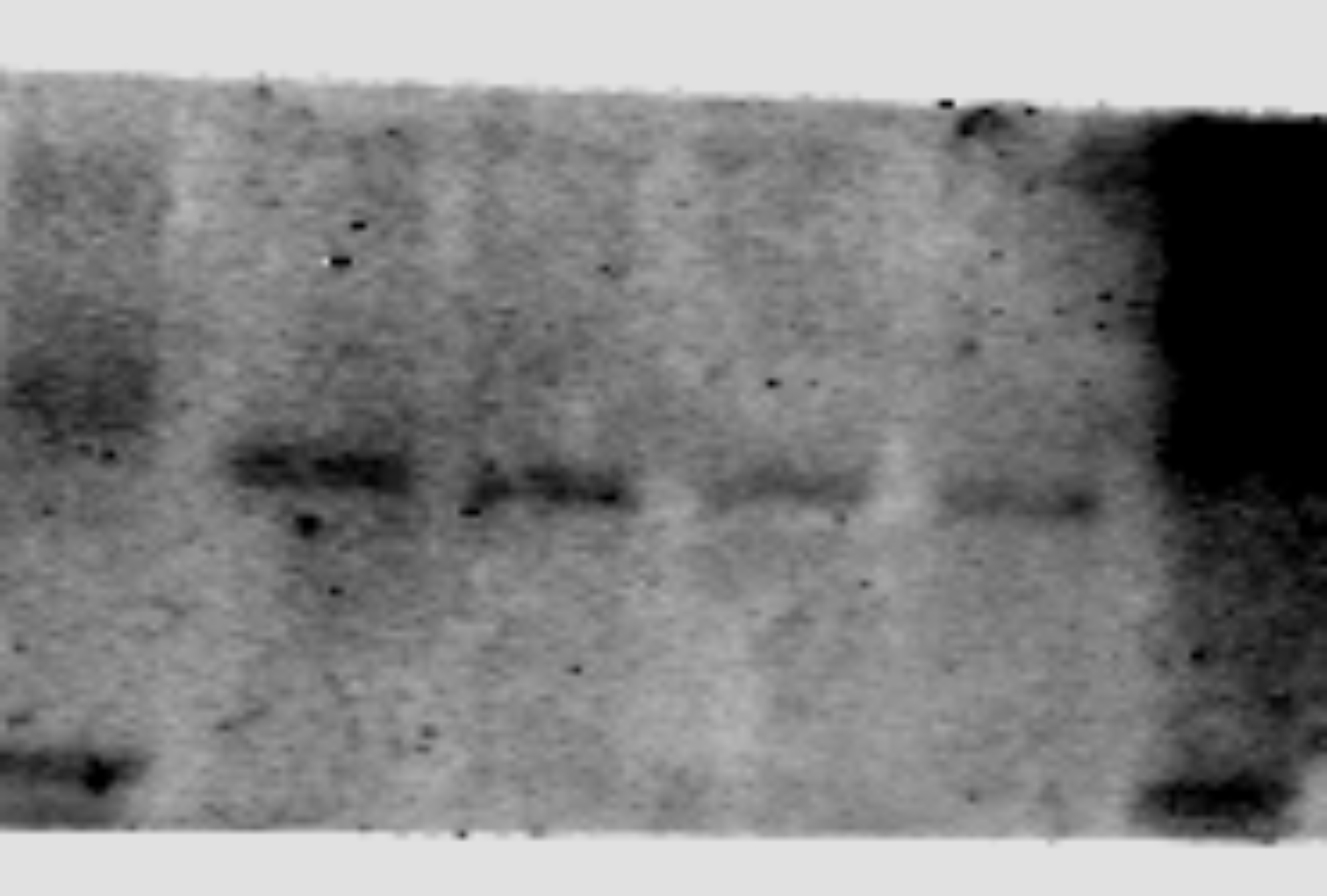

Supplement: Supplementary file 2 — Supplementary Information 2. [file 41598_2023_50476_MOESM2_ESM.zip › protein/2 repeat/2.apoptosis/5637/BCL-2.tif]

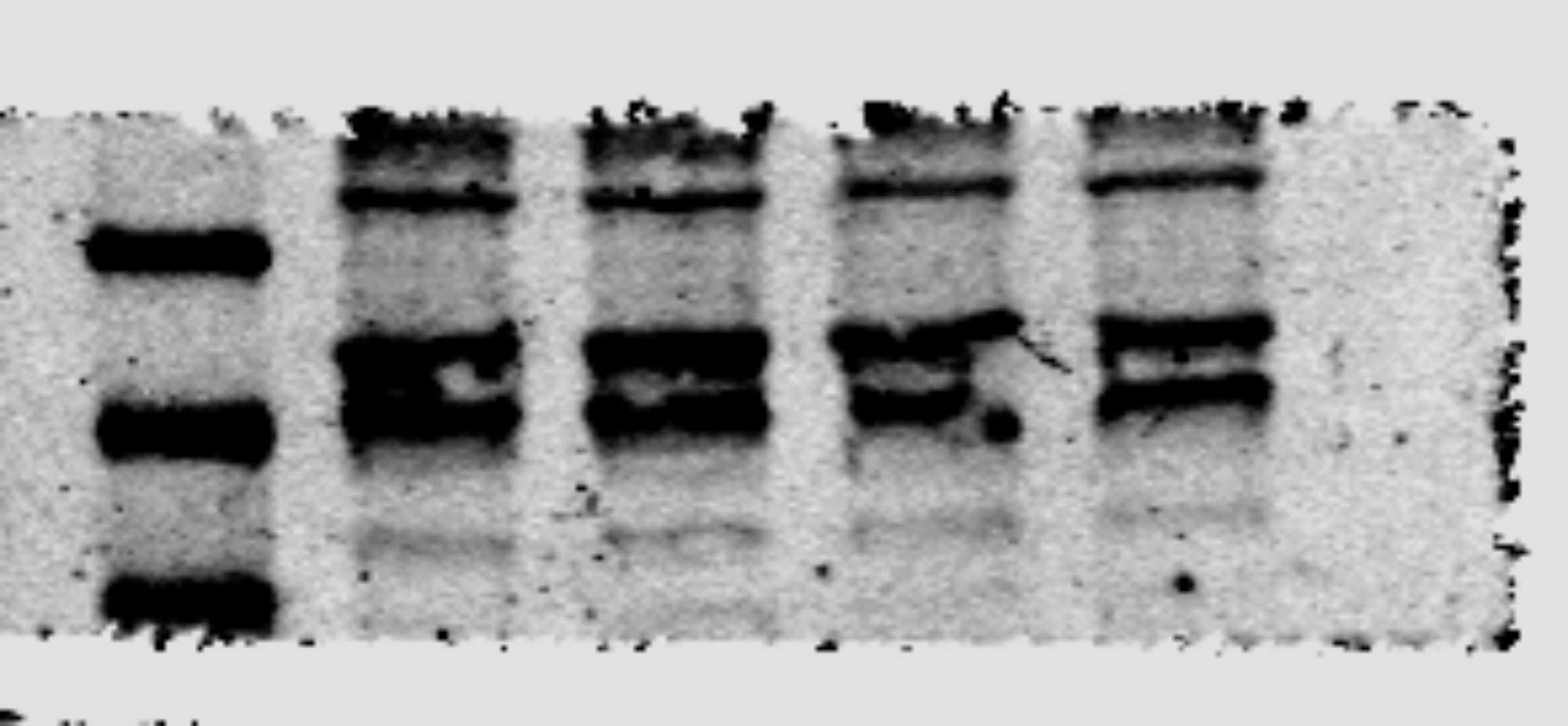

Supplement: Supplementary file 2 — Supplementary Information 2. [file 41598_2023_50476_MOESM2_ESM.zip › protein/2 repeat/2.apoptosis/5637/CASPASE3.tif]

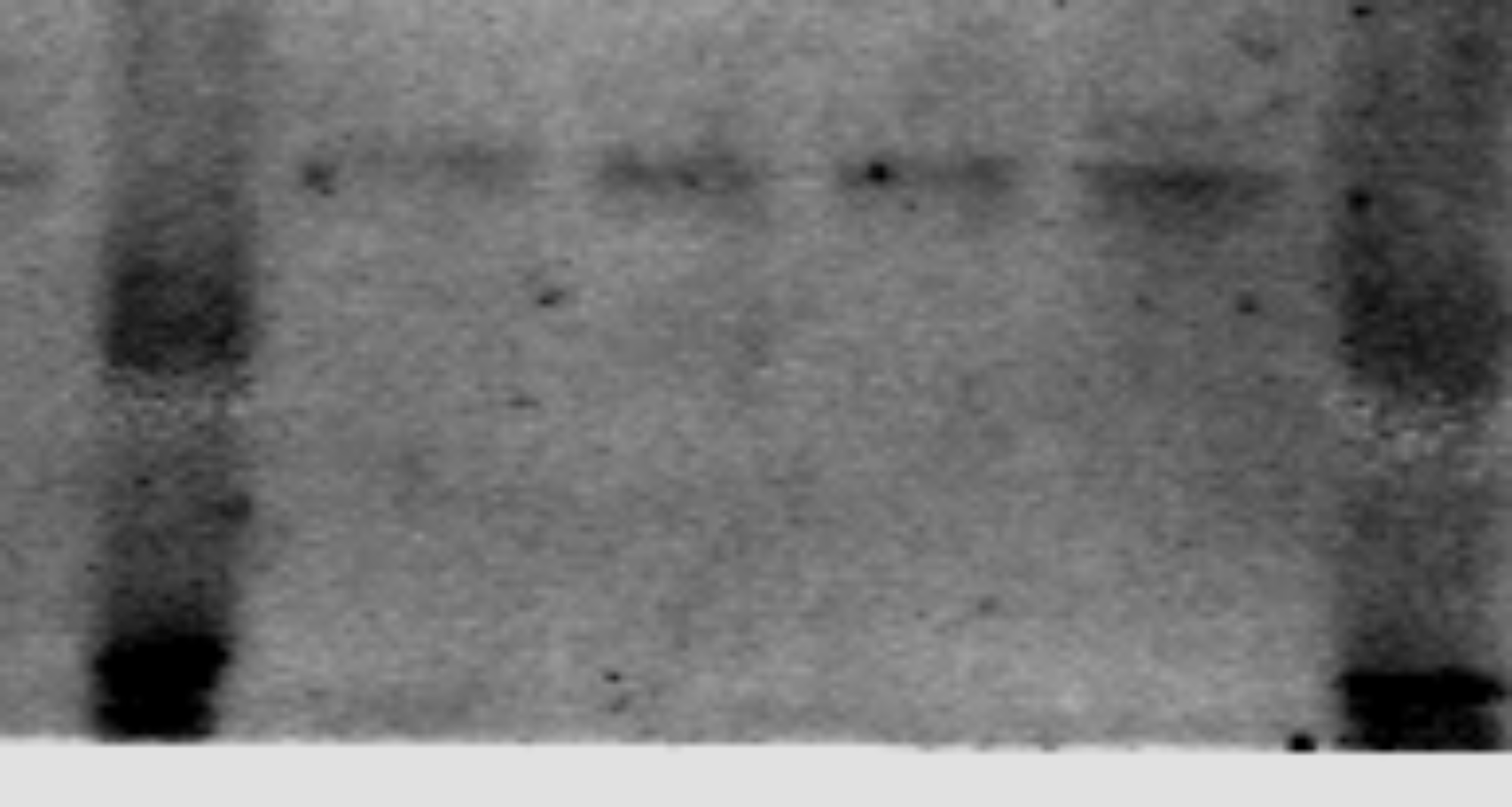

Supplement: Supplementary file 2 — Supplementary Information 2. [file 41598_2023_50476_MOESM2_ESM.zip › protein/2 repeat/2.apoptosis/5637/CLEAVED CASPASE3.tif]

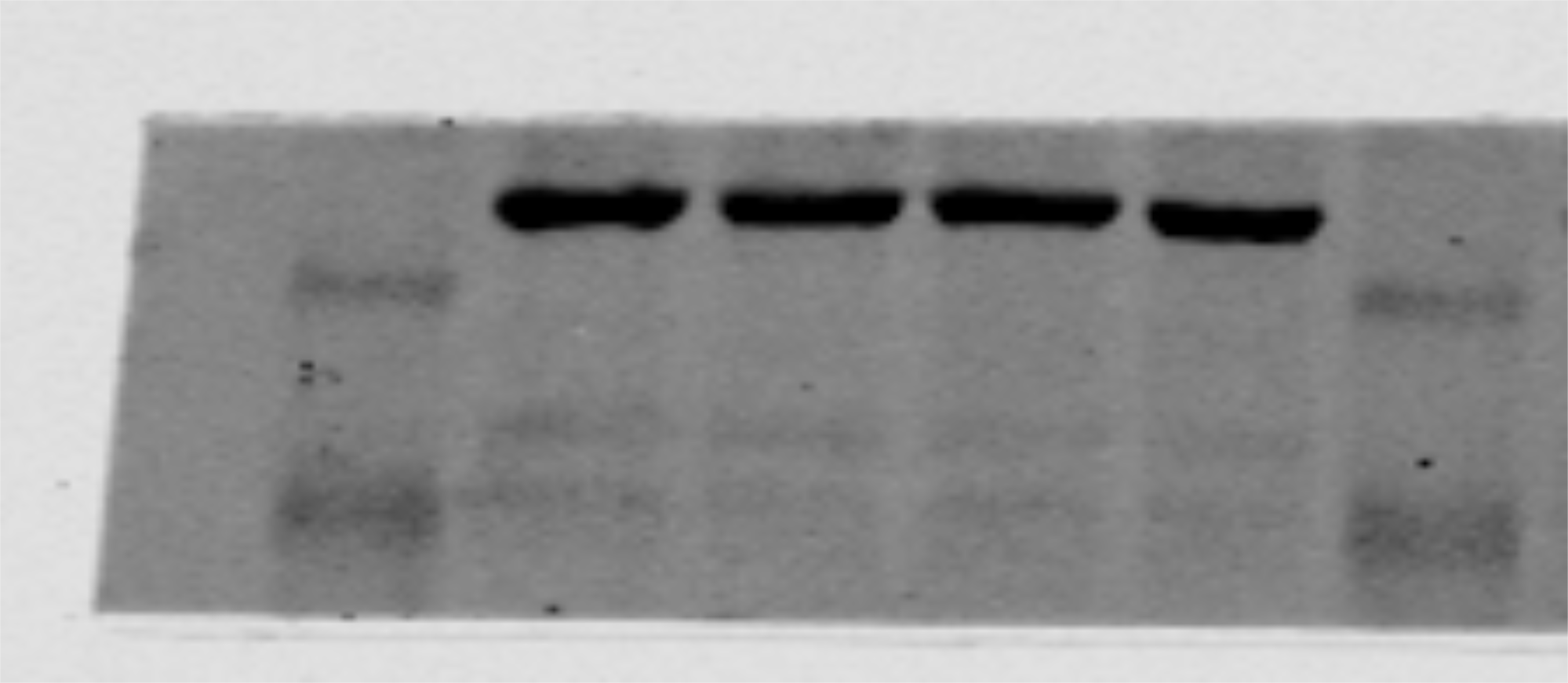

Supplement: Supplementary file 2 — Supplementary Information 2. [file 41598_2023_50476_MOESM2_ESM.zip › protein/2 repeat/2.apoptosis/T24/ACTIN.png]

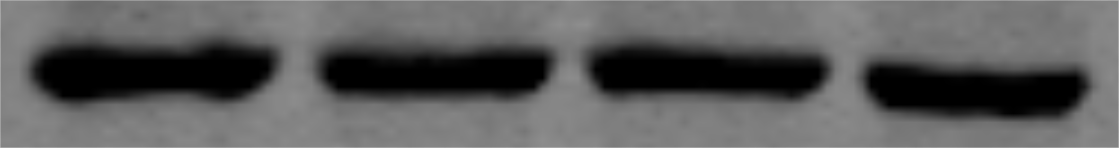

Supplement: Supplementary file 2 — Supplementary Information 2. [file 41598_2023_50476_MOESM2_ESM.zip › protein/2 repeat/2.apoptosis/T24/ACTINCUT.png]

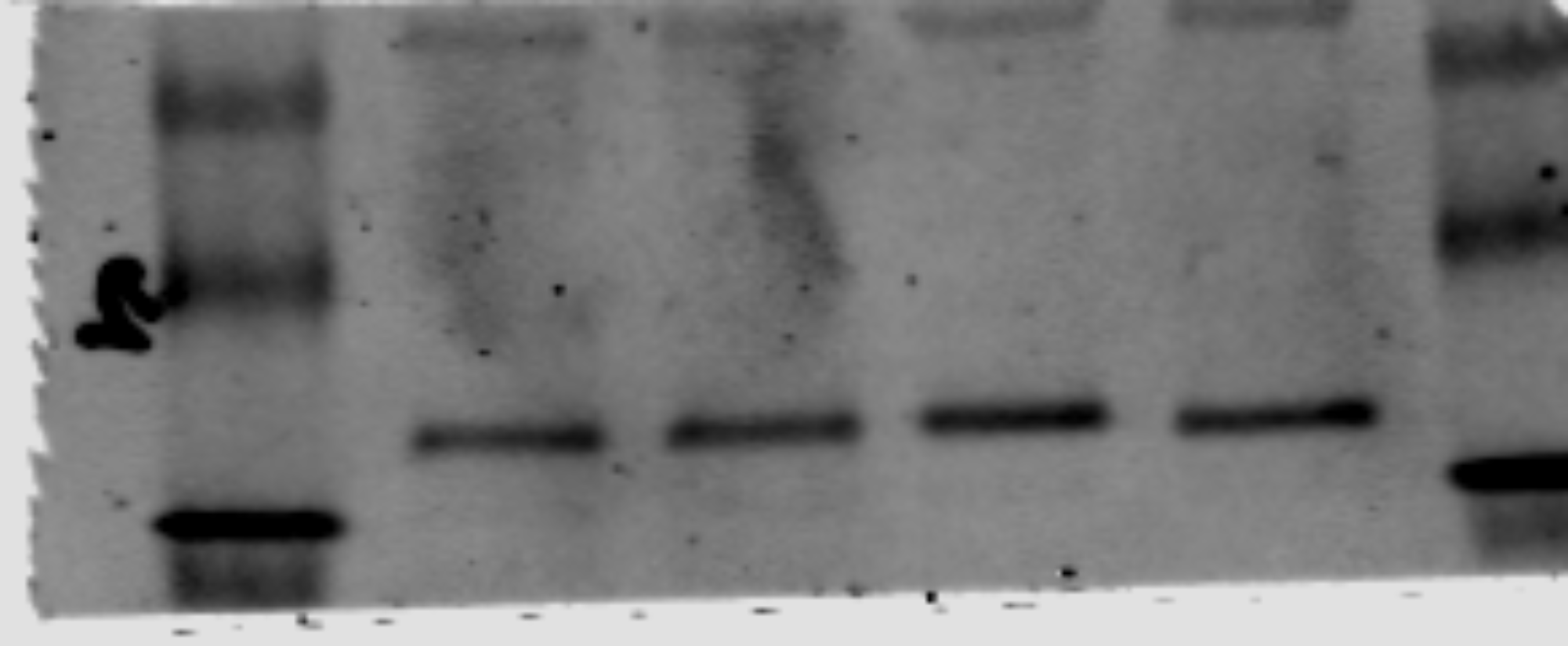

Supplement: Supplementary file 2 — Supplementary Information 2. [file 41598_2023_50476_MOESM2_ESM.zip › protein/2 repeat/2.apoptosis/T24/BAX.png]

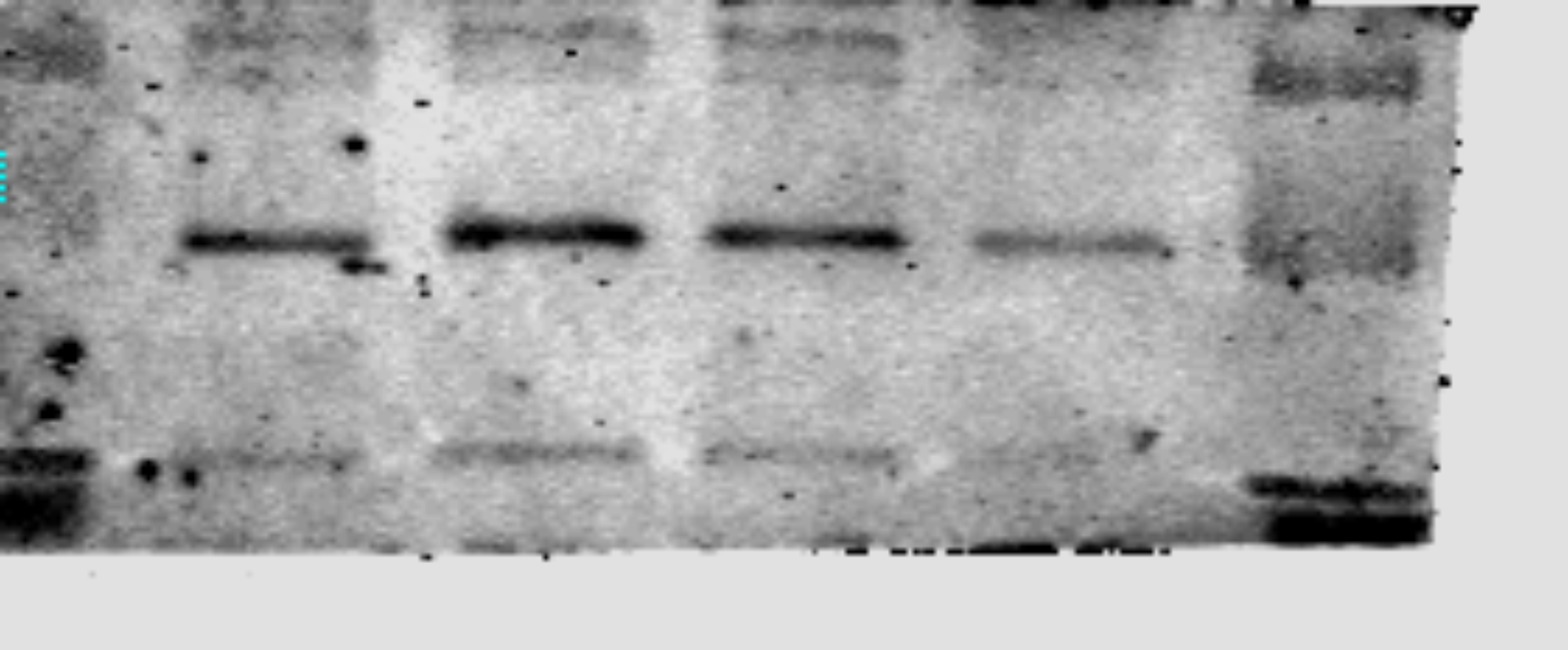

Supplement: Supplementary file 2 — Supplementary Information 2. [file 41598_2023_50476_MOESM2_ESM.zip › protein/2 repeat/2.apoptosis/T24/BCL-2.png]

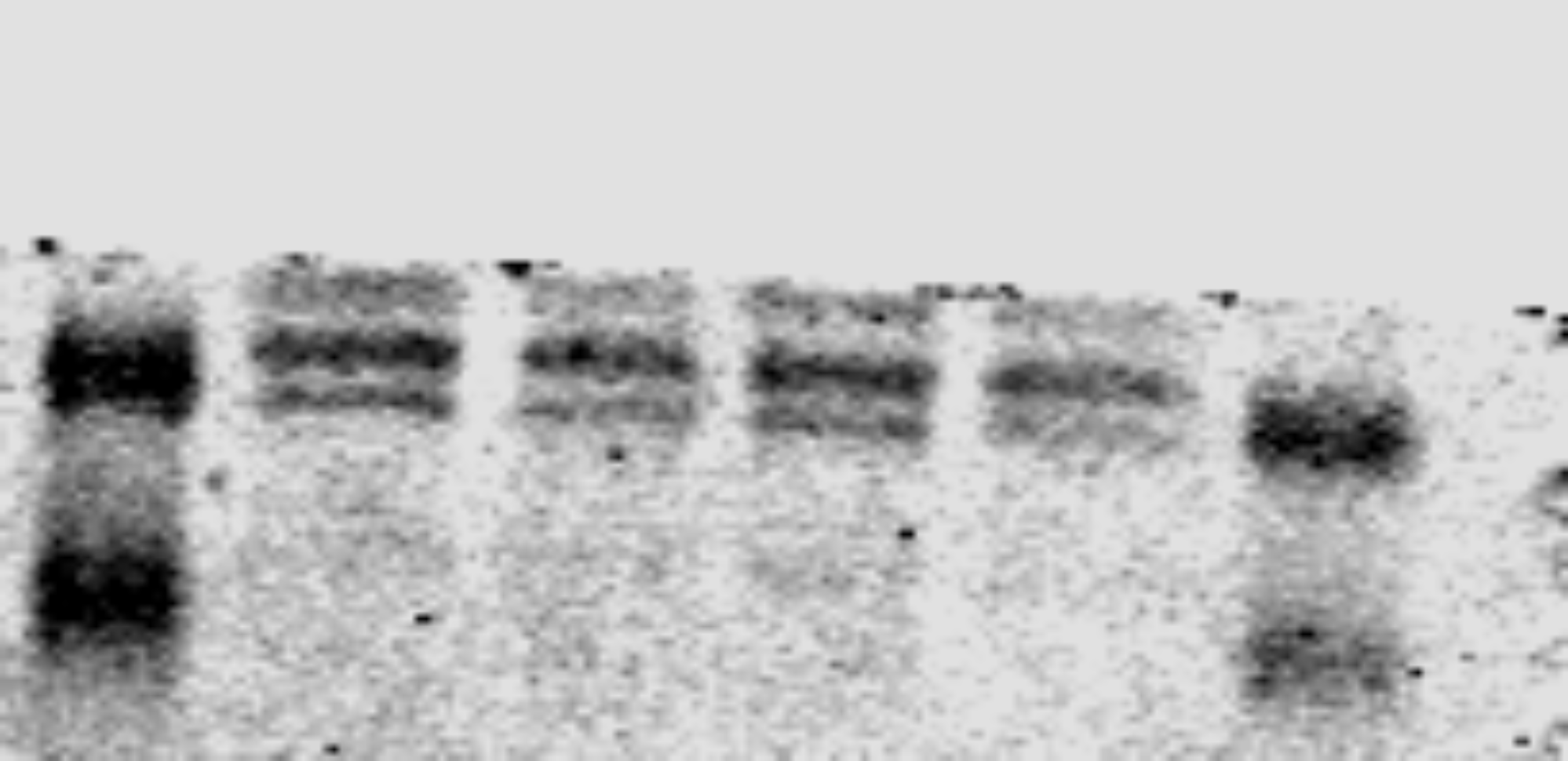

Supplement: Supplementary file 2 — Supplementary Information 2. [file 41598_2023_50476_MOESM2_ESM.zip › protein/2 repeat/2.apoptosis/T24/CASPASE3.tif]

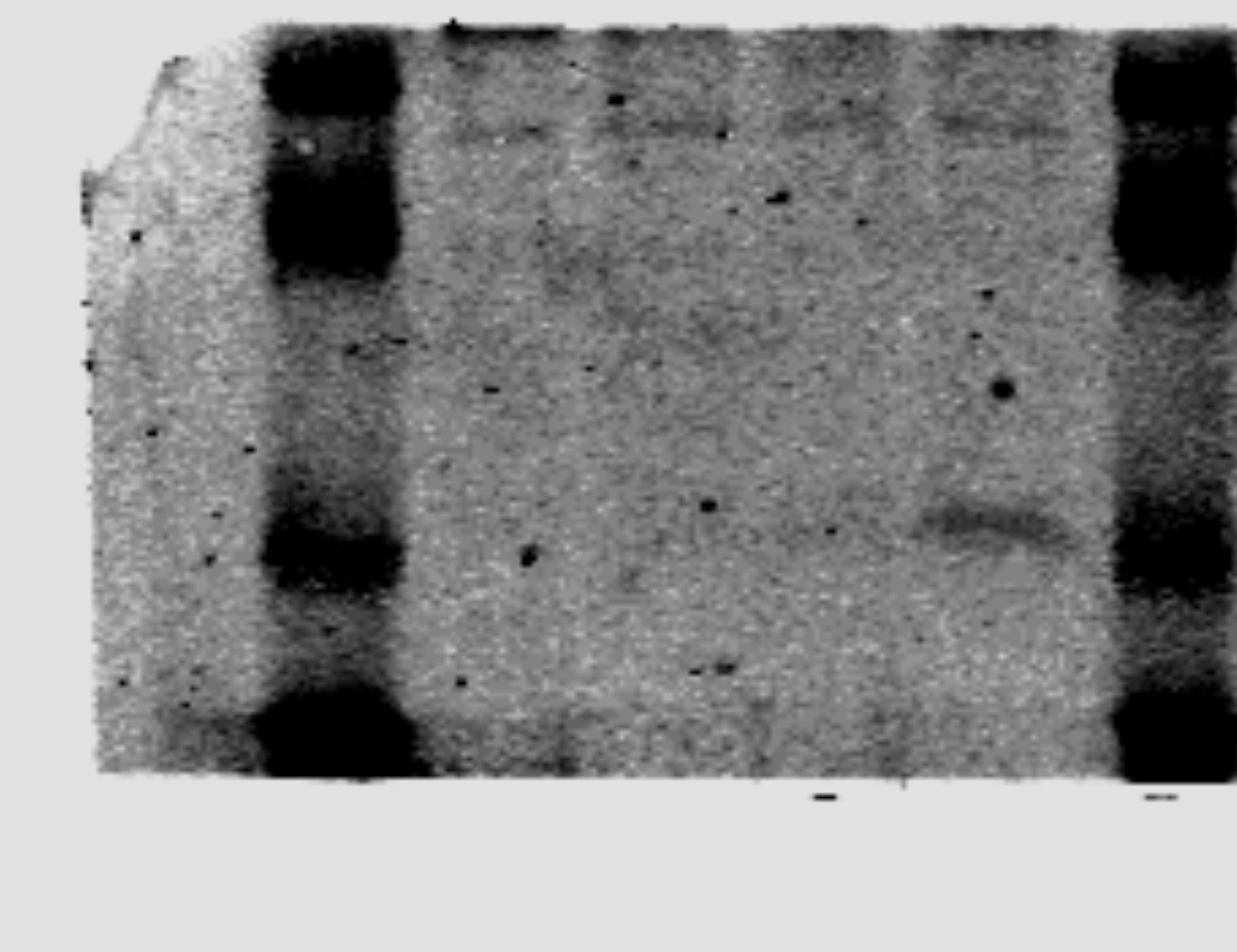

Supplement: Supplementary file 2 — Supplementary Information 2. [file 41598_2023_50476_MOESM2_ESM.zip › protein/2 repeat/2.apoptosis/T24/CLEAVED CASPASE3.tif]

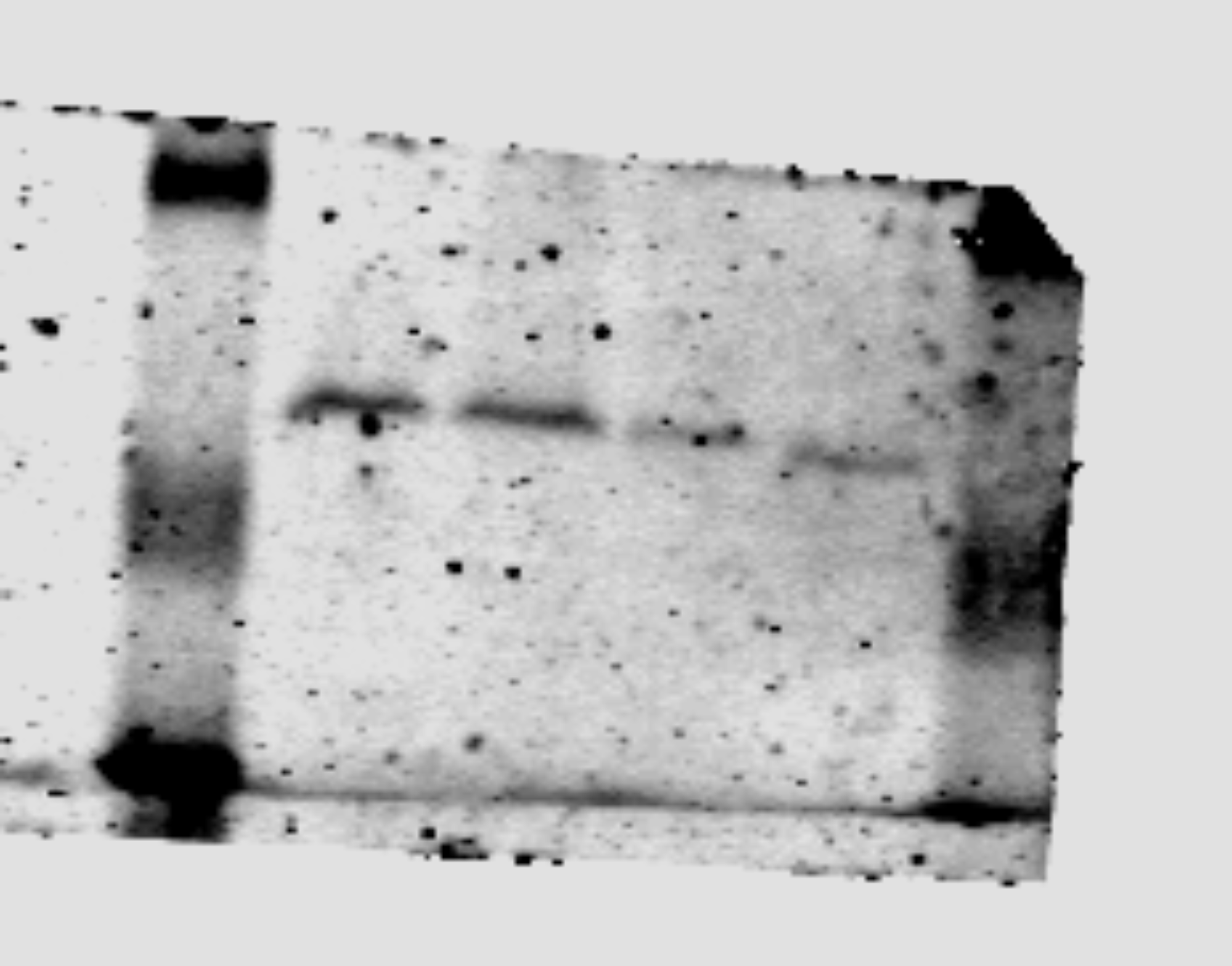

Supplement: Supplementary file 2 — Supplementary Information 2. [file 41598_2023_50476_MOESM2_ESM.zip › protein/2 repeat/3.ferroptosis/5637/1.241.tif]

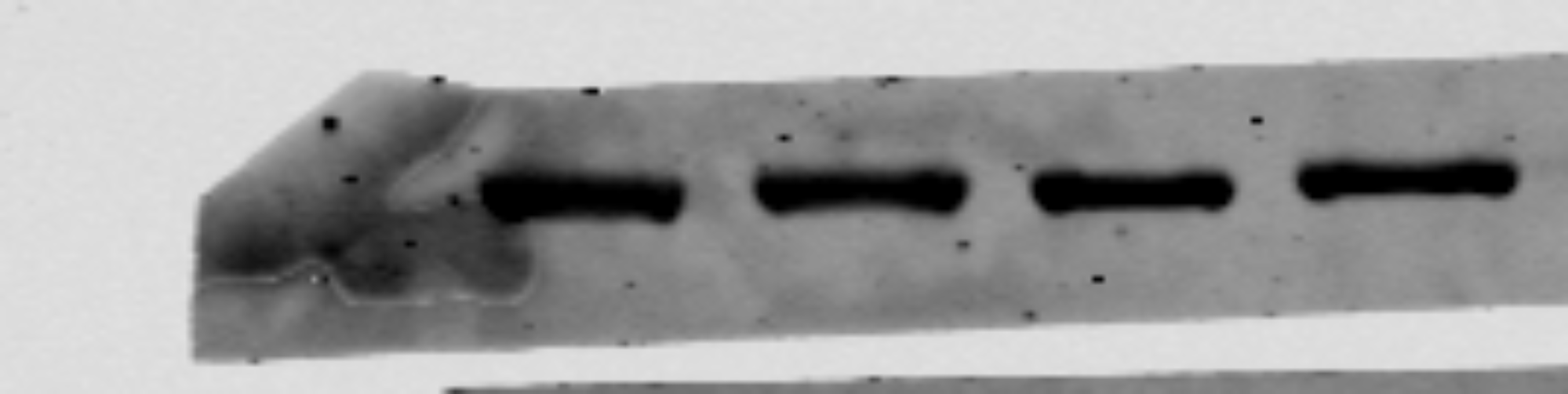

Supplement: Supplementary file 2 — Supplementary Information 2. [file 41598_2023_50476_MOESM2_ESM.zip › protein/2 repeat/3.ferroptosis/5637/26.tif]

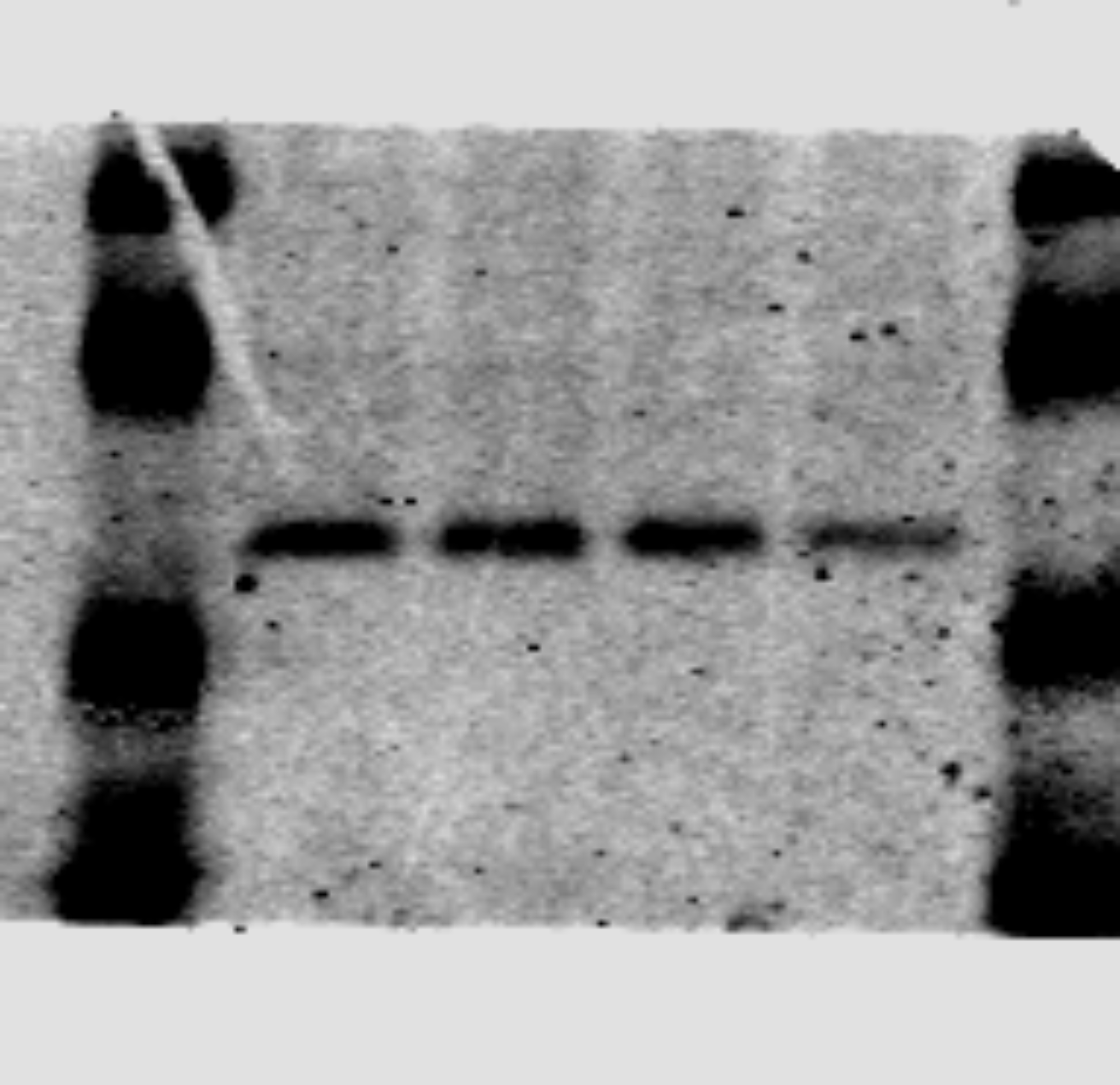

Supplement: Supplementary file 2 — Supplementary Information 2. [file 41598_2023_50476_MOESM2_ESM.zip › protein/2 repeat/3.ferroptosis/T24/2.241.tif]

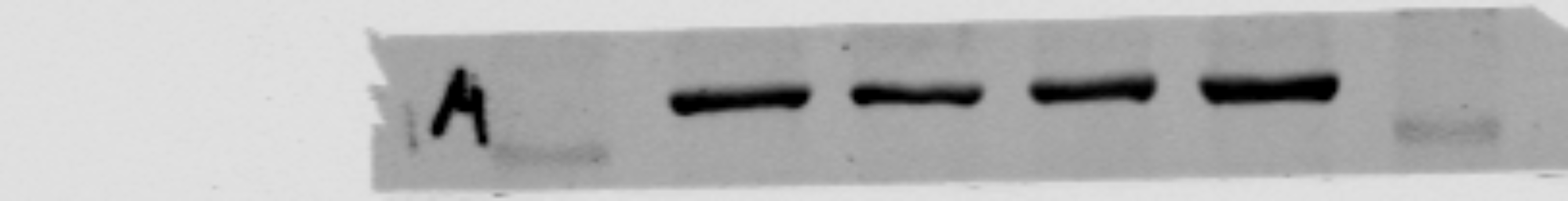

Supplement: Supplementary file 2 — Supplementary Information 2. [file 41598_2023_50476_MOESM2_ESM.zip › protein/2 repeat/3.ferroptosis/T24/25.tif]

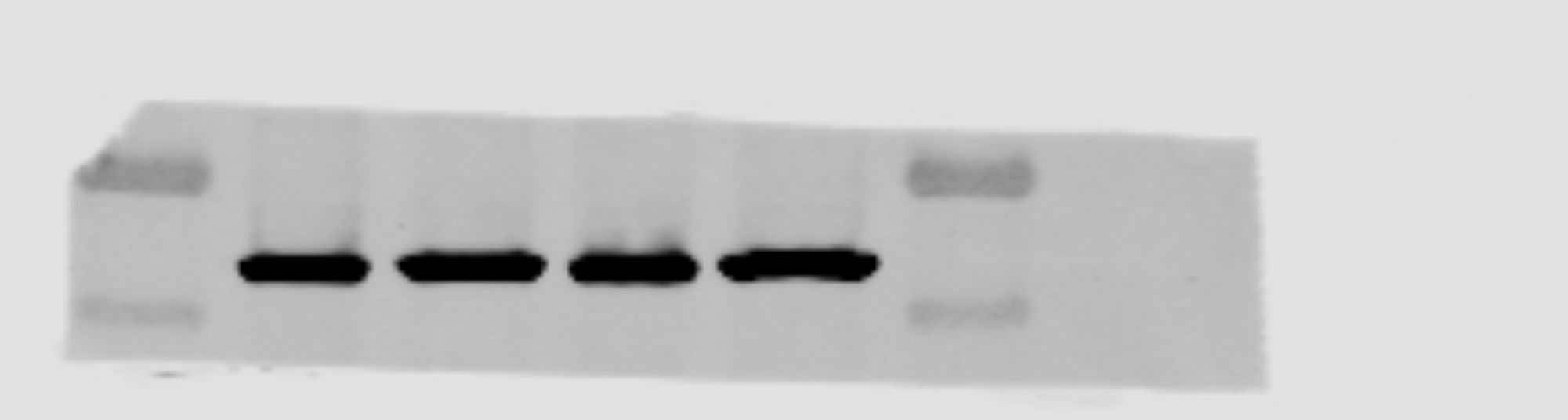

Supplement: Supplementary file 2 — Supplementary Information 2. [file 41598_2023_50476_MOESM2_ESM.zip › protein/2 repeat/4.targets/5637/ACTIN.tif]

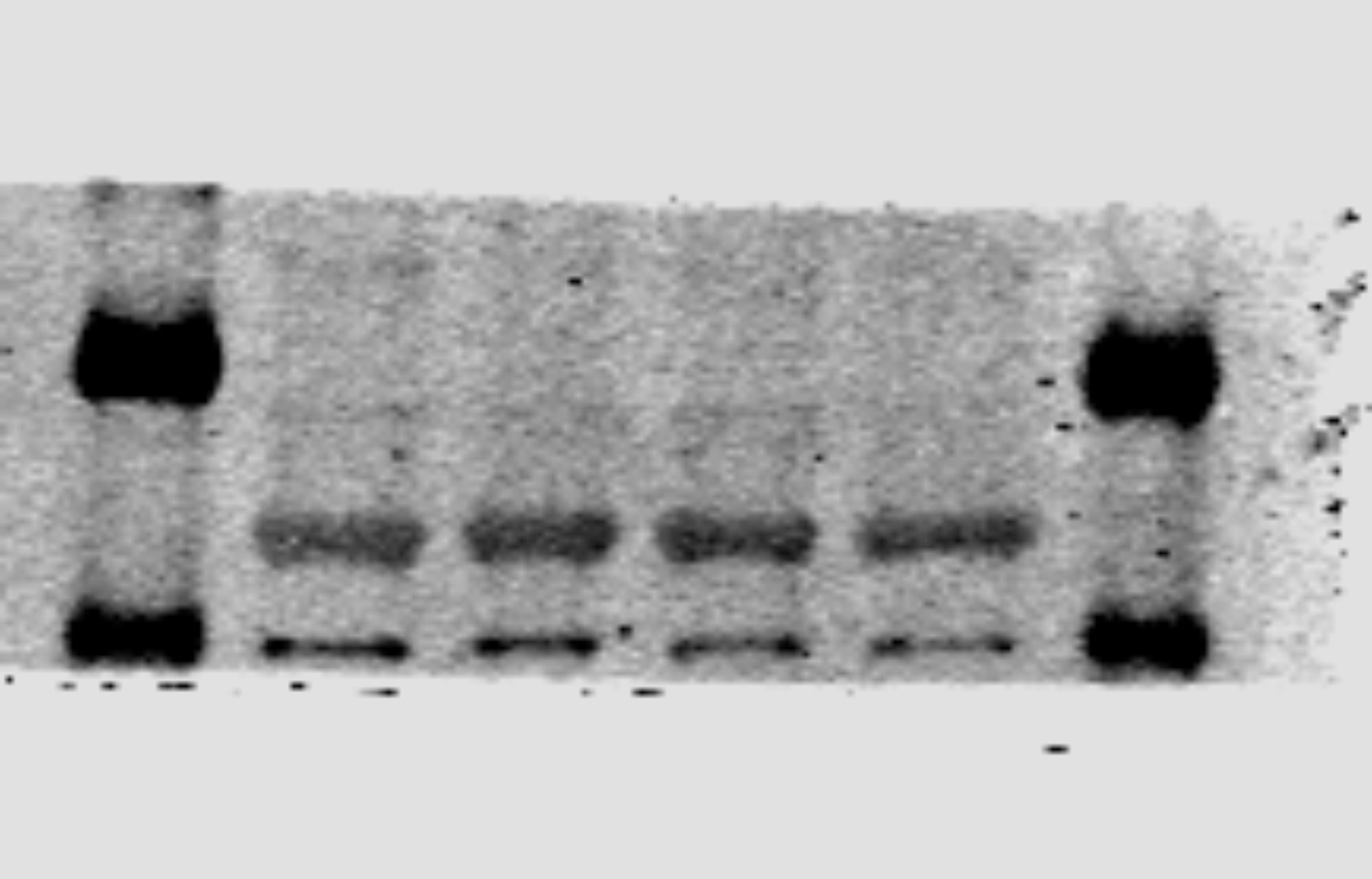

Supplement: Supplementary file 2 — Supplementary Information 2. [file 41598_2023_50476_MOESM2_ESM.zip › protein/2 repeat/4.targets/5637/MAPK1.tif]

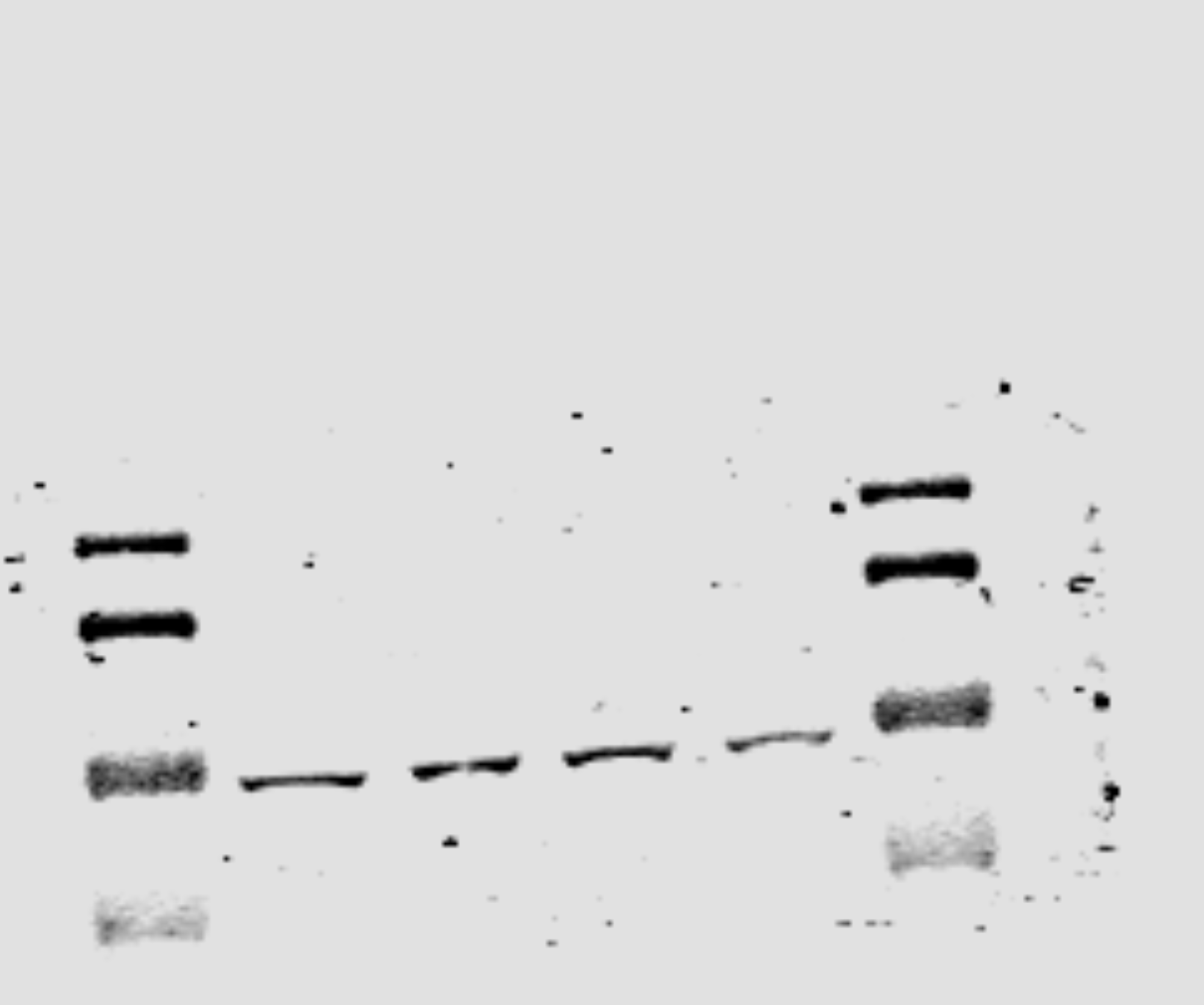

Supplement: Supplementary file 2 — Supplementary Information 2. [file 41598_2023_50476_MOESM2_ESM.zip › protein/2 repeat/4.targets/5637/P85.tif]

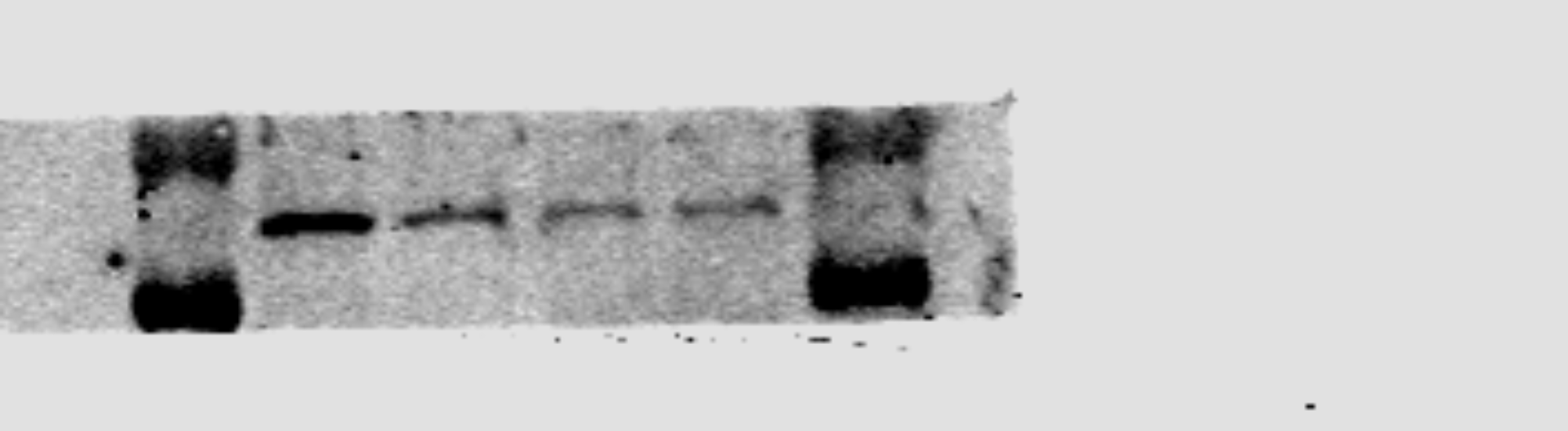

Supplement: Supplementary file 2 — Supplementary Information 2. [file 41598_2023_50476_MOESM2_ESM.zip › protein/2 repeat/4.targets/5637/SRC.tif]
