# Supplementary material for: Hesperetin promotes bladder cancer cells death via the PI3K/AKT pathway by network pharmacology and molecular docking
Source: Sci Rep. 2024 Jan 10;14:1009. doi: 10.1038/s41598-023-50476-8 (PMC10781778; doi:10.1038/s41598-023-50476-8)
Supplement: Supplementary file 3 — Supplementary Legends. [file 41598_2023_50476_MOESM3_ESM.docx]

Appendix figure legends ：

Appendix Figure1：The results of GO analysis were visualized by a circle diagram. Appendix Figure2: The results of GO analysis.

Appendix Figure3: The results of KEGG analysis.
