# Supplementary material for: Hesperetin promotes bladder cancer cells death via the PI3K/AKT pathway by network pharmacology and molecular docking
Source: Sci Rep. 2024 Jan 10;14:1009. doi: 10.1038/s41598-023-50476-8 (PMC10781778; doi:10.1038/s41598-023-50476-8)
Supplement: Supplementary file 1 — Supplementary Information 1. [file 41598_2023_50476_MOESM1_ESM.docx]

Dear editor, the membrane was cropped before we incubated the antibodies so that we could incubate the different antibodies. So we provide the original bands for each protein molecule.And because we develop multiple bands at once. They stick close to each other, so in the post-collation time for a close-up of the image clipping. This allows each band to be displayed independently. But we still managed to keep the edges of each band for review.

一：Article：1.magration Figure6

2. apoptosis Figure7

3. ferroptosis Figure8

4. targets	Figure9

5. pathway	Figure10

二repeat1：

1. magration Figure6

2. apoptosis Figure7
3.

4. ferroptosis Figure8

5. targets	Figure9

5，pathway	Figure10

二repeat2：

1. magration Figure6

2. apoptosis Figure7

3. ferroptosis Figure8

4. targets	Figure9

5，pathway	Figure10
